# Supplementary material for: Easy access to medium-sized lactones through metal carbene migratory insertion enabled 1,4-palladium shift
Source: Nat Commun. 2020 Jan 23;11:461. doi: 10.1038/s41467-019-14101-5 (PMC6978448; doi:10.1038/s41467-019-14101-5)
Supplement: Supplementary file 2 — Supplementary Information [file 41467_2019_14101_MOESM2_ESM.pdf]

Supplementary Information

Easy access to medium-sized lactones through metal carbene migratory insertion enabled 1,4-palladium shift

Yinghua Yu,†[a] Pushkin Chakraborty,†[a] Jinshuai Song,†[b,c] Lei Zhu,[a] Chunsen Li*[b] and Xueliang Huang*[a,d]

[a] Key Laboratory of Coal to Ethylene Glycol and Its Related Technology, Center for Excellence in Molecular Synthesis, Fujian Institute of Research on the Structure of Matter, Chinese Academy of Sciences, Fuzhou, Fujian 350002, China. *e-mail: huangxl@fjirsm.ac.cn

[b] State Key Laboratory of Structural Chemistry, Fujian Institute of Research on the Structure of Matter, Chinese Academy of Sciences, Fuzhou, Fujian 350002, China. *e-mail: chunsen.li@fjirsm.ac.cn

[c] College of Chemistry, and Institute of Green Catalysis, Zhengzhou University, Zhengzhou 450001, China.

[d] State Key Laboratory of Elemento-Organic Chemistry, Nankai University, Tianjin 300071, China

Table of Contents

General information **2**

Optimization of the reaction conditions **3**

Gram-scale synthesis **12**

Substrates involved in the manuscript **13**

Procedures for the preparation of substrates **15**

General procedure for the synthesis of lactones **24**

Deuterium-labeling experiments **62**

Representative synthetic application **64**

Computational details **68**

Crystal data and structure refinement for 66 .... **70**

Supplementary References **185**

Supplementary Methods

General information

Chemicals were purchased from commercial suppliers and used without further purification unless otherwise stated. Solvents were dried by Innovative Technology Solvent Purification System. Reaction progress was monitored by thin layer chromatography (TLC) and components were visualized by observation under UV light at 254nm. Flash column chromatography was performed using silica gel 60 (200-300 mesh). ^{1}H, ^{13}C and ^{19}F NMR spectra were recorded on Bruker-BioSpin AVANCE III HD and JNM-ECZ600S spectrometer. Chemical shifts are reported parts per million (ppm) referenced to $CDCl_3$ (δ 7.26 ppm), tetramethylsilane (TMS, δ 0.00 ppm), DMSO-d_6 (δ 2.50 ppm), CD_3CN (δ 1.94 ppm) for ^{1}H NMR; $CDCl_3$ (δ 77.00 ppm), DMSO-d_6 (δ 40.00 ppm), CD_3CN (δ 118.0 ppm) for ^{13}C NMR. Data for ^{1}H NMR are recorded as follows: chemical shift (δ, ppm), multiplicity (s = singlet; d = doublet; t = triplet; q = quartet; p = pentet; m = multiplet; br = broad), coupling constant (Hz), integration. Data for ^{13}C NMR are reported in terms of chemical shift (δ, ppm). HRMS were obtained on Thermo Scientific LTQ Orbitrap XL and Bruker Impact II UHR-TOF.

Optimization of the reaction conditions

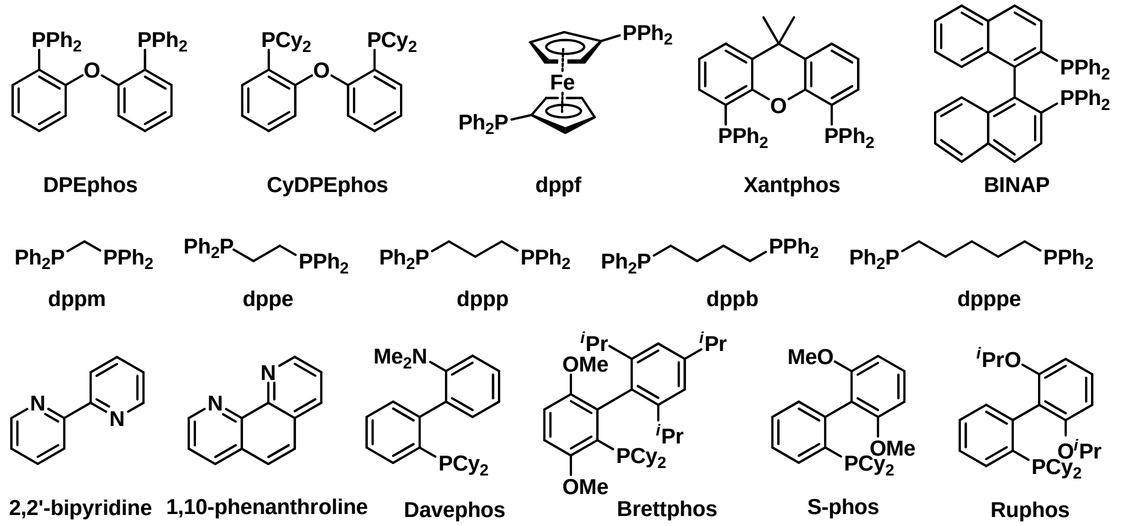

Optimization of the reaction conditions for seven-membered lactone in terms of 2-bromobenzaldehyde.

An oven-dried reaction tube containing a stirring bar was charged with Pd precatalyst (**x** mol%), ligand (**y** mol%), base (**z** equiv.) and **2a** (0.4 mmol, 116 mg). After evacuating and back filling with dry argon, the procedure was repeated for three times, anhydrous solvent (2.0 mL) and **1a** (0.2 mmol, 37 mg) were added via syringe. The mixture was stirred at 80 ^{o}C for 24 h. The crude mixture was cooled to room temperature. EtOAc was added to the mixture. The mixture was filtered through celite. The solvents were evaporated and the crude products were determined via ^{1}H NMR using mesitylene as internal standard.

Supplementary Table 1. Screening of ligands[a]

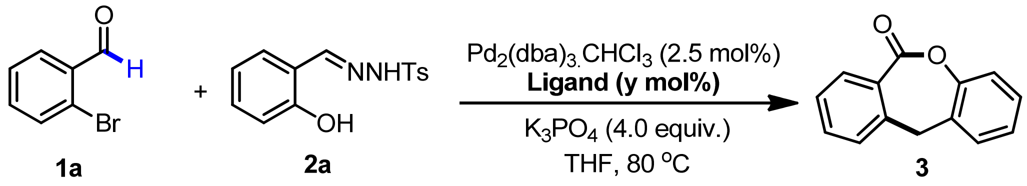

Entry	Ligand (y mol%)	3 (%)[b]
1	DPEphos (7.5)	5
2	dppe (7.5)	52[c]
3	dppf (7.5)	49[c]
4	Tri-2-furylphosphine (15)	0
5	Tri-*o*-tolylphosphine (15)	0
**6**	**Xantphos (7.5)**	**77**
7	dppp (7.5)	67
8	dpppe (7.5)	35
9	BINAP (7.5)	2
10	dppb (7.5)	57
11	dppm (7.5)	0
12	CyDPEphos (7.5)	0

[a] Reaction condition: **1a** (0.2 mmol), **2a** (0.4 mmol), $Pd_2(dba)_3.CHCl_3$ (2.5 mol%), **Ligand (y mol%)**, K_3PO_4 (4.0 equiv) in THF (2.0 mL), stirring under atmosphere of Argon at 80 °C for 24h.
[b] NMR yields were determined using mesitylene as internal standard. [c] Isolated yield.

Supplementary Table 2. Screening of solvents[a]

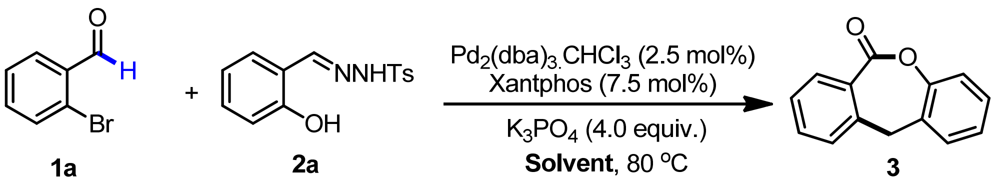

Entry	Solvent	3 (%)[b]
1	MeCN	1
2	Toluene	0.2
3	DMF	trace
4	Dioxane	77
5	DCE	NP
6	PhCl	NP
7	MTBE	NP
8	DME	NP

[a] Reaction condition: **1a** (0.2 mmol), **2a** (0.4 mmol), $Pd_2(dba)_3.CHCl_3$ (2.5 mol%), Xantphos (7.5 mol%), K_3PO_4 (4.0 equiv) in **solvent** (2.0 mL), stirring under atmosphere of Argon at 80 °C for 24h.
[b] NMR yields were determined using mesitylene as internal standard.

Supplementary Table 3. Screening of bases[a]

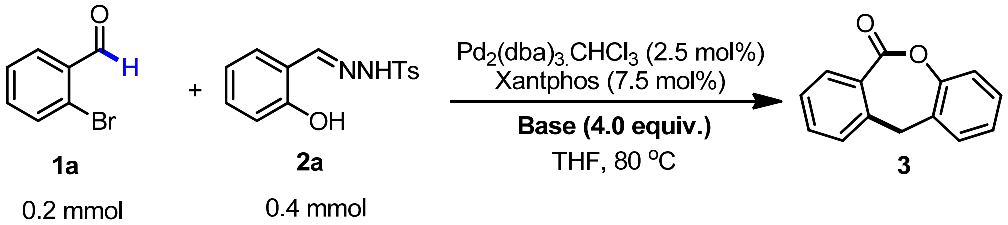

Entry	Base (4.0 equiv.)	**3** (%)[b]
1	Cs_2CO_3	0
2	KOAc	5
3	LiO^{t}Bu	0
4	NaOMe	5
5	Na_2CO_3	0
6	$NaHCO_3$	0
7	NaOAc	0
**8**	**K_2CO_3**	**80 (74)[c,d]**

[a] Reaction condition: **1a** (0.2 mmol), **2a** (0.4 mmol), $Pd_2(dba)_3.CHCl_3$ (2.5 mol%), Xantphos (7.5 mol%), **base (4.0 equiv)** in THF (2.0 mL), stirring under atmosphere of Argon at 80 °C for 24h.
[b] NMR yields were determined using mesitylene as internal standard. [c] Isolated yield. [d] 12h.

Supplementary Table 4. Screening of palladium sources[a]

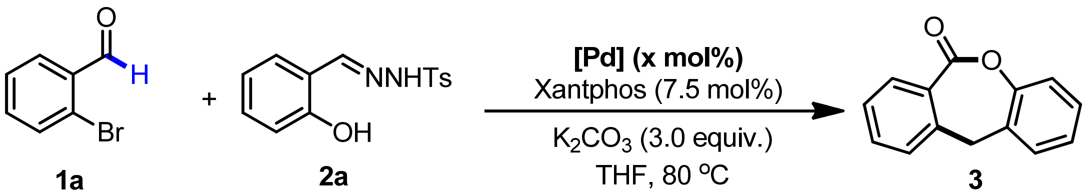

Entry	[Pd] (x mol%)	**3** (%)[b]
**1**	**$Pd(OAc)_2$ (5)**	**76 (76)[c]**
2	$PdCl_2$ (5)	73 (73)[c]
3	(η_3-$C_3H_5)_2Pd_2Cl_2$ (2.5)	84 (75)[c]
4	$Pd(MeCN)_2Cl_2$ (5)	(69)[c]
5	$Pd(SMe_2)_2Cl_2$ (5)	(61)[c]

[a] Reaction condition: **1a** (0.2 mmol), **2a** (0.4 mmol), **[Pd] (x mol%)**, Xantphos (7.5 mol%), K_2CO_3 (3.0 equiv.) in THF (2.0 mL), stirring under atmosphere of Argon at 80 °C for 12h.
[b] NMR yields were determined using mesitylene as internal standard. [c] Isolated yield.

Optimization of the reaction conditions for seven-membered lactone in terms of 2-formylphenyl trifluoromethanesulfonate.

An oven-dried reaction tube containing a stirring bar was charged with Pd precatalyst (**x** mol%), ligand (7.5 mol%), base (3.0 equiv.) and **2a** (0.4 mmol, 116 mg). After evacuating and back filling with dry argon, the procedure was repeated for three times, anhydrous solvent (2.0 mL) and **1c** (0.2 mmol, 51 mg) were added via syringe. The mixture was stirred at 80 °C for 12 h. The crude mixture was cooled to room temperature. Decane (0.2 mmol, 1.0 equiv., 39 μL) was added via microsyringe followed by 5 mL of EtOAc. An aliquot was filtered through a plug of silica and celite and analyzed by GC.

Supplementary Table 5. Screening of solvents[a]

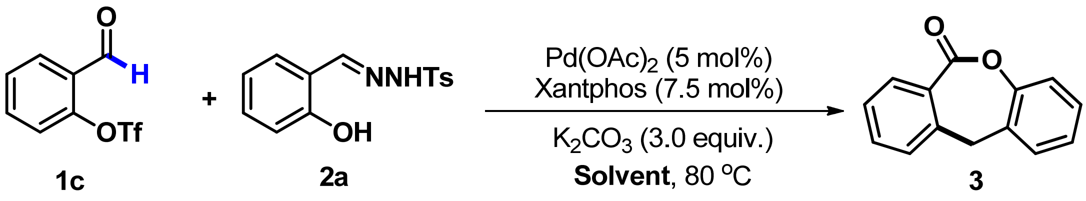

Entry	Solvent	3 (%)[b]
1	THF	53
2	Dioxane	63

[a]Reaction condition: **1c** (0.2 mmol), **2a** (0.4 mmol), Pd(OAc) (5 mol%), Xantphos (7.5 mol%), K_2CO_3 (3.0 equiv) in **solvent** (2.0 mL), stirring under atmosphere of Argon at 80 °C for 12h.
[b] GC yields were determined using decane as internal standard.

Supplementary Table 6. Screening of ligands[a]

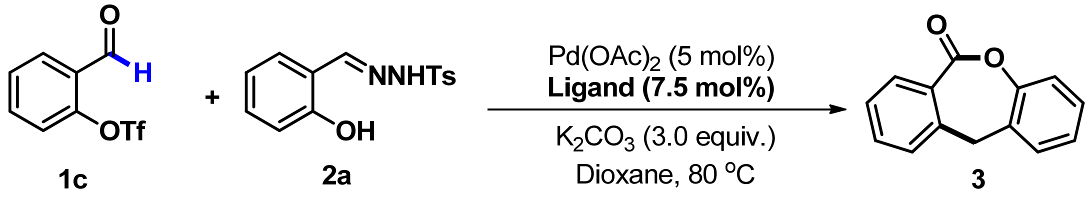

Entry	Ligand	3 (%)[b]
1	Xantphos	63
2	dppm	trace
3	dppe	18
4	dppp	29
5	dppb	15
6	dpppe	15
7	dppf	22
8	BINAP	trace
9	DPEphos	trace
10	CyDPEphos	trace

[a] Reaction condition: **1c** (0.2 mmol), **2a** (0.4 mmol), Pd(OAc) (5 mol%), **Ligand** (7.5 mol%), K_2CO_3 (3.0 equiv) in dioxane (2.0 mL), stirring under atmosphere of Argon at 80 °C for 12h.
[b] GC yields were determined using decane as internal standard.

Supplementary Table 7. Screening of bases[a]

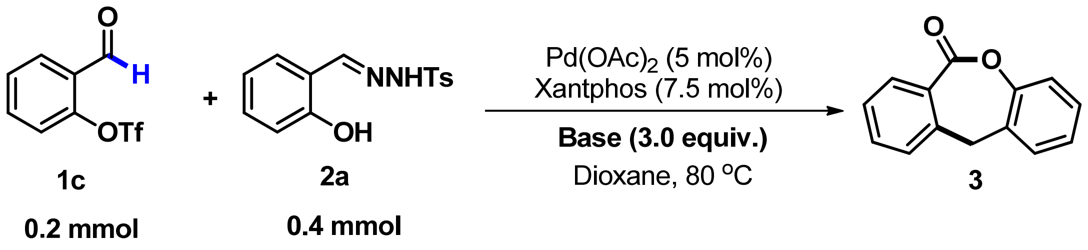

Entry	Base	3 (%)[b]
1	K_3PO_4	53
2	Cs_2CO_3	15
3	LiO^tBu	5
4	KOAc	trace
5	NaOMe	trace
6	K_2CO_3	63

[a] Reaction condition: **1c** (0.2 mmol), **2a** (0.4 mmol), Pd(OAc) (5 mol%), Xantphos (7.5 mol%), **base** (3.0 equiv) in dioxane (2.0 mL), stirring under atmosphere of Argon at 80 °C for 12h.
[b] GC yields were determined using decane as internal standard.

Supplementary Table 8. Screening of palladium sources[a]

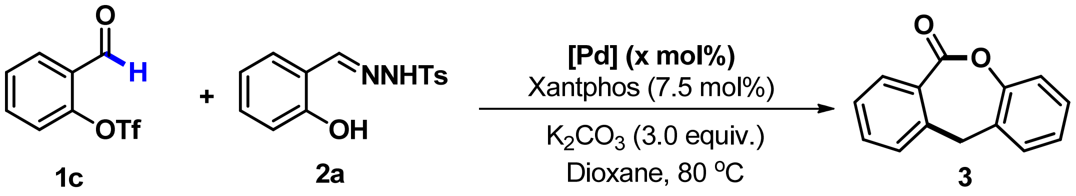

Entry	[Pd] (x mol%)	3 (%)[b]
1	$Pd(OAc)_2$ (5)	63
2	$PdCl_2$ (5)	52
3	$Pd(TFA)_2$ (5)	76
**4**	**(η -$C_3H_5)_2Pd_2Cl_2$ (2.5)**	**85(80)[c]**
5	$Pd(MeCN)_2Cl_2$ (5)	85(79)[c]
6	$Pd_2(dba)_3.CHCl_3$ (2.5)	78

[a] Reaction condition: **1c** (0.2 mmol), **2a** (0.4 mmol), **[Pd] (x mol%)**, Ligand (7.5 mol%), K_2CO_3 (3.0 equiv) in dioxane (2.0 mL), stirring under atmosphere of Argon at 80 °C for 12h. [b] GC yields were determined using decane as internal standard. [c] isolated yield.

Optimization of the reaction conditions for eight-membered lactone.

An oven-dried reaction tube containing a stirring bar was charged with Pd precatalyst (5 mol%), ligand (7.5 mol%), base (3.0 equiv.) and **S2w** (0.4 mmol). After evacuating and back filling with dry argon, the procedure was repeated for three times, anhydrous solvent (2.0 mL) and **1a/ 1b/ 1c** (0.2 mmol) were added via syringe. The mixture was stirred at 80 °C for 10 h. The crude mixture was cooled to room temperature. EtOAc was added to the mixture. The mixture was filtered through celite. The solvents were evaporated and the crude products were determined via ^{1}H NMR using dibromomethane as internal standard.

Supplementary Table 9. Screening of ligands[a]

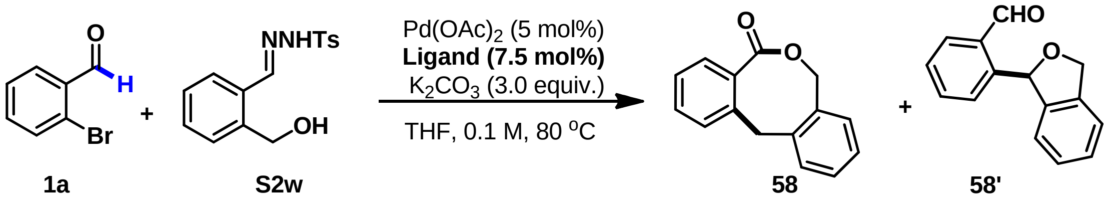

Entry	Ligand (7.5 mol%)	58 (%)[b]	58' (%)[b]
1	Xantphos	15	trace
2	DPEphos	27	8
3	P-(2-furyl)$_3$	-	-
4	Ruphos	trace	-
5	S-phos	trace	-
**6**	**dppb**	**47(30)[c]**	**7**
7	dppf	-	-
8	PPh_3	12	trace
9	BINAP	-	-
10	Brettphos	14	-
11	Davephos	12	-
12	P-(*o*-tolyl)$_3$	19	-
13	P-(*p*-$C_6H_5CF_3$)$_3$	14	-
14	P(Cy)$_3$.HBF_4	10	-
12	2,2'-bipyridine	-	-
13	1,10-phenanthroline	-	-

[a] Reaction condition: **1a** (0.2 mmol), **S2w** (0.4 mmol), $Pd(OAc)_2$ (5 mol%), **Ligand** (7.5 mol%), K_2CO_3 (3.0 equiv.) in THF (2.0 mL), stirring under atmosphere of Argon at 80 °C for 10 h. [b] NMR yields were determined using dibromomethane as internal standard. [c] Isolated yield.

Supplementary Table 10. Screening of solvents[a]

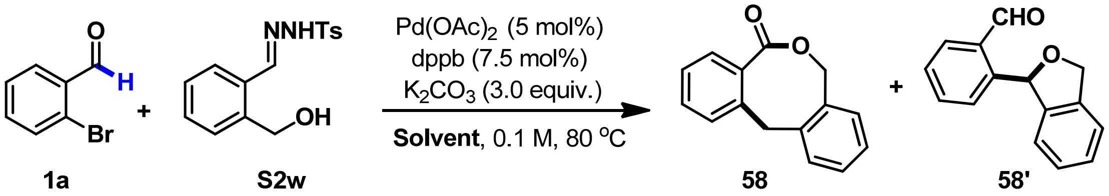

Entry	Solvent	58 (%)[b]	58' (%)[b]
1	Toluene	45	8
2	DMF	-	-
3	Dioxane	23	-
4	Acetonitrile	trace	-
5	DME	44	7
6	$PhCF_3$	46	9
**7**	**THF**	**47**	**7**

[a] Reaction condition: **1a** (0.2 mmol), **S2w** (0.4 mmol), $Pd(OAc)_2$ (5 mol%), dppb (7.5 mol%), K_2CO_3 (3.0 equiv.) in **solvent** (2.0 mL), stirring under atmosphere of Argon at 80 °C for 10 h. [b] NMR yields were determined using dibromomethane as internal standard.

Supplementary Table 11. Screening of bases[a]

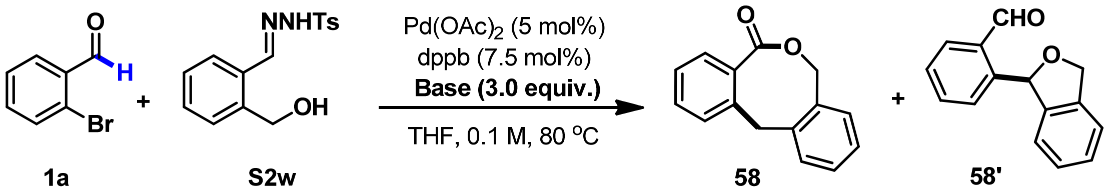

Entry	Base	58 (%)[b]	58' (%)[b]
1	$NaHCO_3$	trace	-
2	Na_2CO_3	trace	-
**3**	**K_3PO_4**	**56 (44)[c]**	**10**
4	Cs_2CO_3	36	6
5	LiO^tBu	-	-
6	KOH	37	9
7	KH_2PO_4	trace	-
8	K_3PO_4 (4.0 equiv.)	52	10
9	KOAc	31	14

[a] Reaction condition: **1a** (0.2 mmol), **S2w** (0.4 mmol), $Pd(OAc)_2$ (5 mol%), dppb (7.5 mol%), **Base (3.0 equiv.)** in THF (2.0 mL), stirring under atmosphere of Argon at 80 °C for 10 h.
[b] NMR yields were determined using dibromomethane as internal standard. [c] Isolated yield.

Supplementary Table 12. Screening of palladium sources[a]

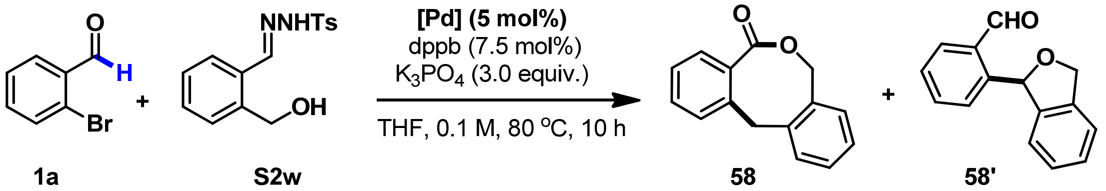

Entry	[Pd] (5 mol%)	58 (%)[b]	58' (%)[b]
1	$Pd(PPh_3)_2Cl_2$	38	8
2	$PdCl_2$	38	9
**3**	**$[(\eta\text{-}C_3H_6)PdCl]_2$**	**72 (64)[c]**	**6**
4	$Pd(TFA)_2$	-	-
5	$Pd(OAc)_2$	56 (44)[c]	9
6	$Pd(dba)_2$	44	12

[a] Reaction condition: **1a** (0.2 mmol), **S2w** (0.4 mmol), **[Pd] (5 mol%)**, dppb (7.5 mol%), K_3PO_4 (3.0 equiv.) in THF (2.0 mL), stirring under atmosphere of Argon at 80 °C for 10 h.
[b] NMR yields were determined using dibromomethane as internal standard. [c] Isolated yield.

Supplementary Table 13. Final screening: achieving optimal conditions[a]

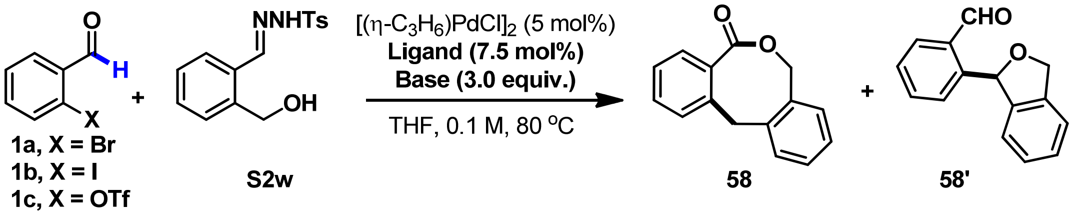

Entry	X	Ligand	Base	58 (%)[b]	58' (%)[b]
1	Br	dppb	K_2CO_3	74 (64)[c]	8
2	Br	dppb	Cs_2CO_3	23	5
3	I	dppb	K_3PO_4	trace	-
4	Cl	dppb	K_3PO_4	-	-
5	OTf	dppb	K_3PO_4	75 (71)[c]	7
**6**	**OTf**	**dppb**	**K_2CO_3**	**85 (76)[c]**	**7 (5)[c]**
7	OTf	dppm	K_2CO_3	40	10
8	OTf	dppe	K_2CO_3	40	9
9	OTf	dppp	K_2CO_3	72	10
10	OTf	dpppe	K_2CO_3	54	9
11[d]	OTf	dppb	K_2CO_3	78 (71)[c]	15 (11)[c]

[a] Reaction condition: **1a/ 1b/ 1c** (0.2 mmol), **S2w** (0.4 mmol), [(η-C_3H_6)PdCl]$_2$ (5 mol%), **Ligand (7.5 mol%)**, **Base(3.0 equiv.)** in THF (2.0 mL), stirring under atmosphere of Argon at 80 °C for 10 h. [b] NMR yields were determined using dibromomethane as internal standard. [c] Isolated yield.
[d] [(η-C_3H_6)PdCl]$_2$ (2.5 mol%), dppb (7.5 mol%) for 16 h.

Gram-scale synthesis

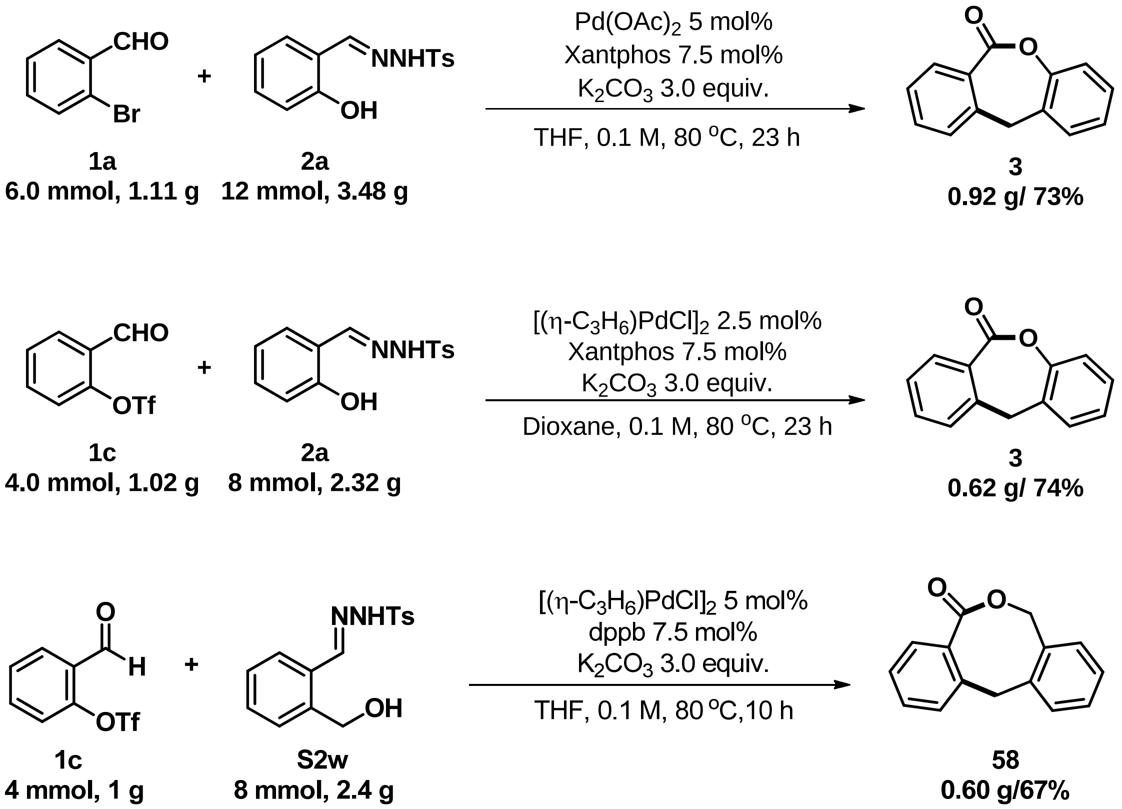

Supplementary Figure 1. Gram-scale synthesis. Synthesis of lactones under optimal reaction conditions.

Substrates involved in the manuscript

Aldehyde derivatives:

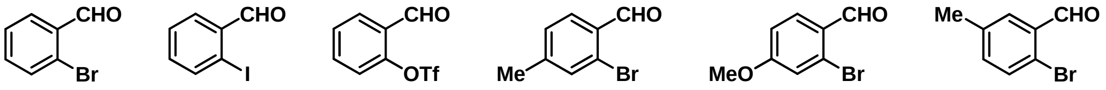

1a 1b 1c S1a S1b S1c

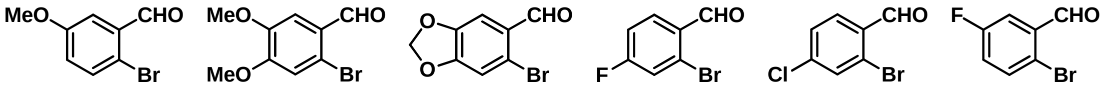

S1d S1e S1f S1g S1h S1i

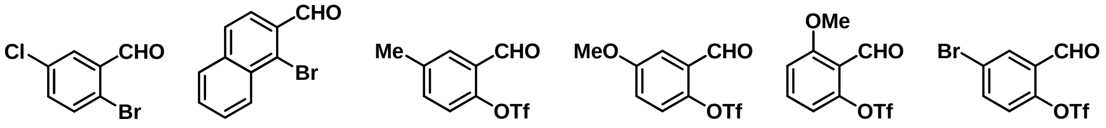

S1j S1k S1l S1m S1n S1o

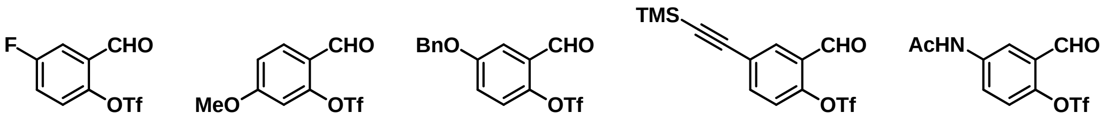

S1p S1q S1r S1s S1t

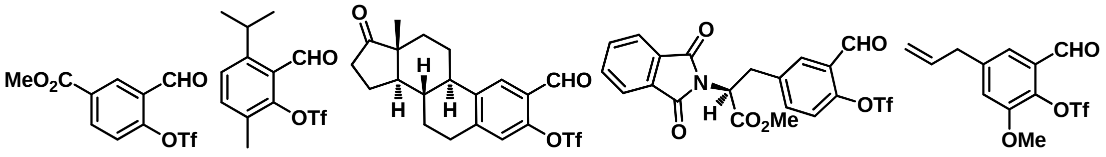

S1u S1v S1w S1x S1y

N-tosylhydrazone:

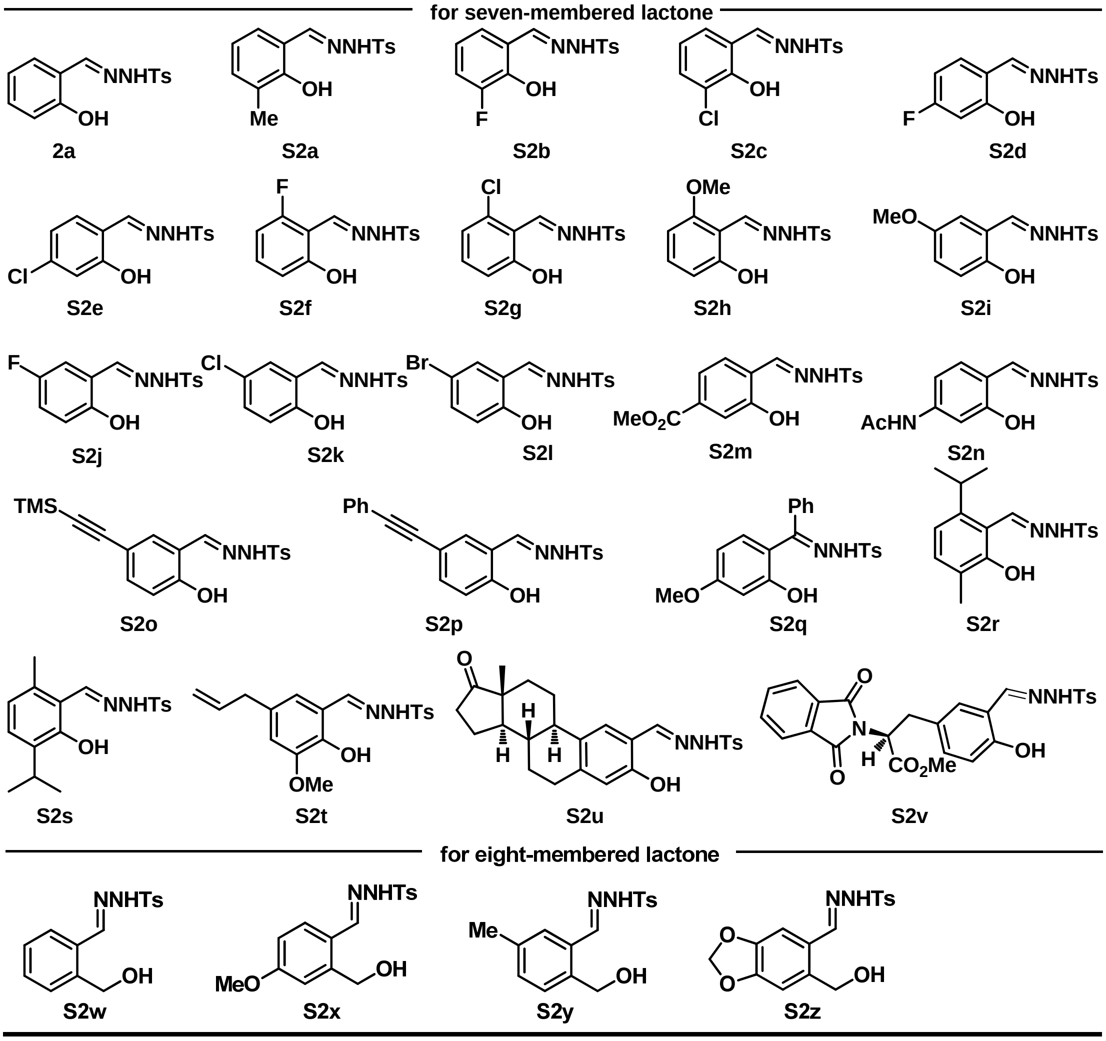

Supplementary Figure 2. Substrates involved in the manuscript.

Procedures for the preparation of substrates

The salicylaldehyde analogues with substituents on the aromatic ring **S1s-a**[1], **S1t-a** (paracitamol)[2], **S1u-a** (methylparaben)[3,4], **S1v-a** (thymol)[3,4], **S1w-a** (estrone)[5], **S1x-a** (methyl N-Phth-L-tyrosinate)[5] were prepared according to the reference. The other salicylaldehyde analogues were commercial available.

5.1 General procedure for the synthesis S1[6]

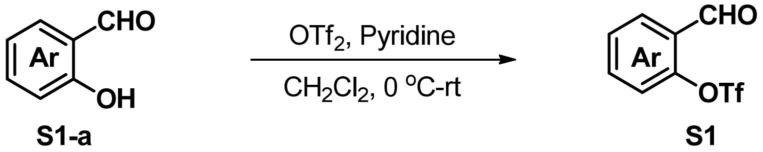

To a solution of salicylaldehyde (2.44 g, 20 mmol) in CH_2Cl_2 (60 mL) were successively added pyridine (4.8 mL, 60 mmol) and Tf_2O (5.0 mL, 30 mmol) at 0 °C. After being stirred for 3 h at room temperature, the reaction was stopped by adding saturated aqueous $NaHCO_3$ at 0 °C. The crude products were extracted with CH_2Cl_2 (x 4) and the combined organic extracts were washed with 1 M aqueous HCl (x1), brine, dried (Na_2SO_4), and concentrated in vacuo. The residue was purified by column chromatography (silica gel, PE/EtOAc = 10/1) to give **1c** (4.63 g, 91%) as a colorless oil.

4-Acetamido-2-formylphenyl trifluoromethanesulfonate (S1t)

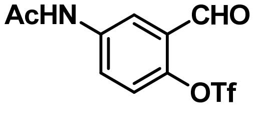

Yield 70%, slightly yellow solid; R_f = 0.30 (petroleum ether : ethyl acetate = 2 : 1); **^{1}H NMR (600 MHz, DMSO-d_6)** δ 10.48 (s, 1H), 10.04 (s, 1H), 8.28 (d, J = 2.4 Hz, 1H), 7.95 (dd, J_1 = 9.0 Hz, J_2 = 3.0 Hz, 1H),

7.54 (d, J = 9.0 Hz, 1H), 2.09 (s, 3H); **^{13}C NMR (151 MHz, DMSO-d_6)** δ 189.2, 169.6, 143.0, 140.6, 128.8, 125.9, 123.9, 122.9, 122.8, 118.7 (q, J = 320.7 Hz), 24.5; **^{19}F NMR (376 MHz, DMSO-d_6)** δ -73.1; **HRMS (ESI)** m/z calcd for $C_{10}H_9F_3NO_5S^+$ $(M+H)^+$ 312.01480, found 312.01489.

(8S,9R,13R,14R)-2-Formyl-13-methyl-17-oxo-7,8,9,11,12,13,14,15,16,17-decahydro-6H-cyclopenta[a]phenanthren-3-yl trifluoromethanesulfonate (S1w)

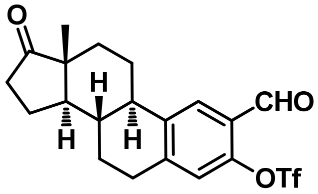

Yield 80%, White solid; R_f = 0.40 (petroleum ether : ethyl acetate = 5 : 1); **^{1}H NMR (600 MHz, CDCl$_3$)** δ 10.11 (s, 1H), 7.85 (s, 1H), 7.07 (s, 1H), 3.03-2.93 (m, 2H), 2.47 (q, J = 9.0 Hz, 2H), 2.30 (dd, J_1 = 10.8 Hz, J_2 = 3.6 Hz, 1H), 2.15-2.02 (m, 3H), 1.96 (d, J = 13.8 Hz, 1H), 1.65-1.44 (m, 6H), 0.88 (s, 3H); **^{13}C NMR (151 MHz, CDCl$_3$)** δ 219.9, 186.4, 147.4, 146.5, 141.1, 128.1, 125.8, 122.2, 118.4 (q, J = 320.9 Hz), 50.1, 47.6, 43.7, 37.3, 35.6, 31.2, 29.7, 25.6, 25.4, 21.4, 13.6; **^{19}F NMR (376 MHz, CDCl$_3$)** δ -72.9; **HRMS (ESI)** m/z calcd for $C_{20}H_{22}F_3O_5S^+$ $(M+H)^+$ 431.11346, found 431.11340.

(S)-Methyl 2-(1,3-dioxoisoindolin-2-yl)-3-(3-formyl-4-(((trifluoromethyl)sulfonyl)oxy)phenyl)propanoate (S1x)

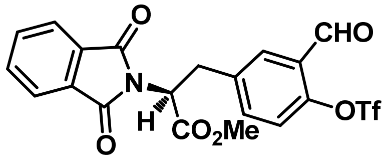

Yield 81%, White solid; R_f = 0.40 (petroleum ether : ethyl acetate = 5 : 1); **^{1}H NMR (600 MHz, CDCl$_3$)** δ 10.09 (s, 1H), 7.79-7.76 (m, 3H), 7.70-7.69 (m, 2H), 7.53 (dd, J_1 = 8.4 Hz, J_2 = 1.8 Hz, 1H), 7.24 (d, J = 8.4 Hz, 1H), 5.15 (q, J = 5.4 Hz, 1H), 3.74 (s, 3H), 3.68 (dd, J_1 =

14.4 Hz, J_2 = 5.4 Hz, 1H), 3.58 (dd, J_1 = 14.4 Hz, J_2 = 10.8 Hz, 1H); **^{13}C NMR (151 MHz, $CDCl_3$)** δ 186.2, 168.6, 167.2, 148.4, 138.4, 136.1, 134.4, 131.3, 131.2, 128.2, 123.6, 122.5, 118.4 (q, J = 327.4 Hz,), 53.0, 52.3, 34.0; **^{19}F NMR (376 MHz, $CDCl_3$)** δ -72.9; **HRMS (ESI)** m/z calcd for $C_{20}H_{15}F_3NO_8S^+$ $(M+H)^+$ 486.04650, found 486.04681.

4-Allyl-2-formyl-6-methoxyphenyl trifluoromethanesulfonate (S1y)

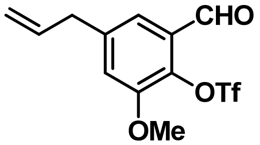

Yield 87%, White solid; R_f = 0.50 (petroleum ether : ethyl acetate = 20 : 1); **^{1}H NMR (600 MHz, $CDCl_3$)** δ 10.15 (s, 1H), 7.30 (d, J = 2.4 Hz, 1H), 7.12 (d, J = 2.4 Hz, 1H), 5.94-5.87 (m, 1H), 5.15-5.10 (m, 2H), 3.92 (s, 3H), 3.42 (d, J = 7.2 Hz, 2H); **^{13}C NMR (151 MHz, $CDCl_3$)** δ 186.7, 151.4, 141.9, 137.3, 135.3, 129.0, 120.9, 118.7, 118.5 (q, J = 318.9 Hz), 117.4, 56.3, 39.6; **^{19}F NMR (376 MHz, $CDCl_3$)** δ -72.9; **HRMS (ESI)** m/z calcd for $C_{12}H_{12}F_3O_5S^+$ $(M+H)^+$ 325.03521, found 325.03506.

5.2 General procedure for the synthesis of *N*-tosylhydrazones used for seven-membered lactones[7]

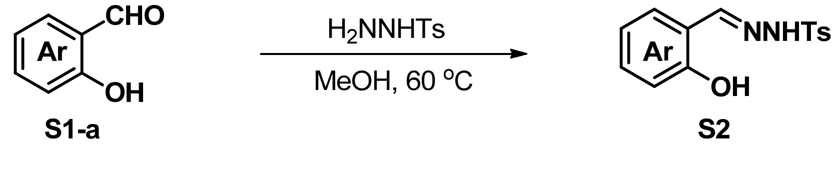

Salicylaldehyde (4.8 mL, 44 mmol) was dissolved in methanol (30 mL), then $TsNHNH_2$ (7.44 g, 40 mmol) was added to the reaction mixture. The resulting mixture was heated at 60 °C for 2 h. After cooling to room temperature, the precipitates were filtered and washed by petroleum ether, and then kept in desiccator under vacuum to

afford pure product **2a** (10.4 g, 90%).

N'-(2-hydroxy-5-((trimethylsilyl)ethynyl)benzylidene)-4-methylbenzenesulfonohydrazide (S2o)

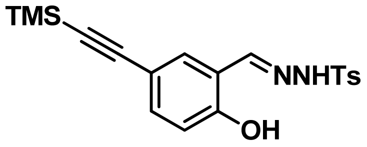

Yield 87%, white solid; R_f = 0.40 (petroleum ether : ethyl acetate = 5 : 1); **^{1}H NMR (400 MHz, $CDCl_3$)** δ 10.33 (s, 1H), 8.36 (s, 1H), 7.88 (s, 1H), 7.84 (d, *J* = 8.0 Hz, 2H), 7.38-7.34 (m, 3H), 7.26-7.25 (m, 1H), 6.86 (d, *J* = 8.8 Hz, 1H), 2.42 (s, 3H), 0.22 (s, 9H); **^{13}C NMR (101 MHz, $CDCl_3$)** δ 158.0, 150.9, 145.1, 135.6, 134.7, 134.1, 130.1, 127.8, 117.2, 116.9, 114.5, 103.9, 93.1, 21.6, -0.1; **HRMS (ESI)** m/z calcd for $C_{19}H_{23}N_2O_3SSi^+$ $(M+H)^+$ 387.11932, found 387.11972.

N'-(2-hydroxy-5-(phenylethynyl)benzylidene)-4-methylbenzenesulfonohydrazide (S2p)

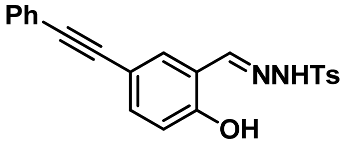

Yield 89%, white solid; R_f= 0.50 (petroleum ether : ethyl acetate = 3 : 1); **^{1}H NMR (400 MHz, $CDCl_3$)** δ 10.35 (s, 1H), 8.32-8.28 (m, 1H), 7.92 (s, 1H), 7.85 (d, *J* = 8.4 Hz, 2H), 7.49-7.47 (m, 2H), 7.43 (dd, J_1 = 8.8 Hz, J_2 = 2.0 Hz, 1H), 7.36-7.32 (m, 6H), 6.92 (d, *J* = 8.8 Hz, 1H), 2.42 (s, 3H); **^{13}C NMR (101 MHz, $CDCl_3$)** δ 158.0, 151.1, 145.1, 135.3, 134.3, 134.1, 131.4, 130.1, 128.4, 128.2, 127.9, 123.1, 117.5, 117.0, 114.6, 88.4, 88.2, 21.7; **HRMS (ESI)** m/z calcd for $C_{22}H_{19}N_2O_3S^+$ $(M+H)^+$ 391.11109, found 391.11127.

Methyl 4-hydroxy-3-((2-tosylhydrazono)methyl)benzoate (S2m)

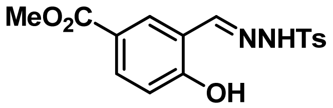

Yield 65%, white solid; R_f = 0.40 (petroleum ether : ethyl acetate = 1 : 1); **^{1}H NMR (400 MHz, DMSO-*d*$_6$)** δ 11.63 (s, 1H), 11.09 (s, 1H), 8.22 (s, 1H), 8.14 (d, *J* = 2.0 Hz, 1H), 7.83 (dd, J_1 = 8.8 Hz, J_2 = 2.0 Hz, 1H), 7.76 (d, *J* = 8.4 Hz, 2H), 7.43 (d, *J* = 8.4 Hz, 2H), 6.97 (d, *J* = 8.8 Hz, 1H), 3.83 (s, 3H), 2.37 (s, 3H); **^{13}C NMR (101 MHz, DMSO-*d*$_6$)** δ 166.2, 160.8, 144.4, 144.2, 136.5, 132.9, 130.3, 128.7, 127.6, 121.3, 120.1, 116.9, 52.4, 21.54; **HRMS (ESI)** m/z calcd for $C_{16}H_{17}N_2O_5S^+$ $(M+H)^+$ 349.08527, found 349.08502.

N-(3-hydroxy-4-((2-tosylhydrazono)methyl)phenyl)acetamide (S2n)

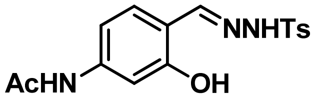

Yield 78%, white solid; R_f = 0.40 (petroleum ether : ethyl acetate = 1 : 3); **^{1}H NMR (400 MHz, DMSO-*d*$_6$)** δ11.43 (s, 1H), 9.92 (s, 1H), 9.81 (s, 1H), 8.14 (s, 1H), 7.74 (d, *J* = 8.0 Hz, 2H), 7.66 (d, *J* = 2.4 Hz, 1H), 7.49 (dd, J_1 = 8.8 Hz, J_2 = 2.4 Hz, 1H), 7.42 (d, *J* = 8.0 Hz, 2H), 6.78 (d, *J* = 8.8 Hz, 1H), 2.36 (s, 3H), 1.99 (s, 3H); **^{13}C NMR (101 MHz, DMSO-*d*$_6$)** δ 168.3, 152.8, 145.5, 144.1, 136.5, 132.0, 130.3, 127.6, 123.6, 119.5, 117.6, 117.0, 24.3, 21.6; **HRMS (ESI)** m/z calcd for $C_{16}H_{18}N_3O_4S^+$ $(M+H)^+$ 348.10125, found 348.10110.

N'-(((8*S*,9*R*,13*R*,14*R*)-3-hydroxy-13-methyl-17-oxo-7,8,9,11,12,13,14,15,16,17-decahydro-6H-cyclopenta[*a*]phenanthren-2-yl)methylene)-4-methylbenzenesulfonohydrazide (S2u)

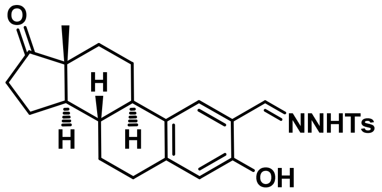

Yield 62%, white solid; R_f = 0.30 (petroleum ether : ethyl acetate = 2 : 1); **^{1}H NMR (400 MHz, CDCl$_3$)** δ 9.99 (s, 1H), 8.34 (s, 1H), 7.97 (s, 1H), 7.82 (d, *J* = 8.4 Hz, 2H), 7.32 (d, *J* = 8.4 Hz, 2H), 7.01 (s, 1H), 6.67 (s, 1H), 2.88-2.85 (m, 2H), 2.52 (dd, J_1 = 19.2 Hz, J_2 = 8.8 Hz, 1H), 2.40 (s, 3H), 2.33-1.93 (m, 6H), 1.66-1.35 (m, 6H), 0.89 (s, 3H); **^{13}C NMR (101 MHz, CDCl$_3$)** δ 221.7, 155.6, 152.4, 144.6, 141.5, 134.4, 131.2, 129.9, 127.9, 127.8, 116.6, 115.0, 50.2, 48.0, 43.4, 38.0, 35.9, 31.4, 29.4, 26.2, 25.8, 21.6, 21.5, 13.7. **HRMS (ESI)** m/z calcd for $C_{26}H_{31}N_2O_4S^+$ $(M+H)^+$ 467.19990, found 467.20020.

(S)-Methyl 2-(1,3-dioxoisoindolin-2-yl)-3-(4-hydroxy-3-((2-tosylhydrazono)methyl)phenyl)propanoate (S2v)

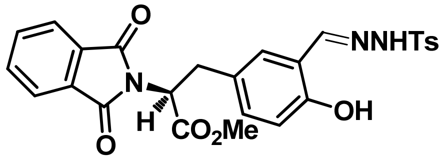

Yield 86%, white solid; R_f = 0.30 (petroleum ether : ethyl acetate = 2 : 1); **^{1}H NMR (400 MHz, DMSO-*d$_6$*)** δ 11.41 (s, 1H), 10.06 (s, 1H), 8.04 (s, 1H), 7.86 (s, 4H), 7.73 (d, *J* = 8.4 Hz, 2H), 7.41 (d, *J* = 8.0 Hz, 2H), 7.32 (d, *J* = 1.2 Hz, 1H), 7.02 (dd, J_1 = 8.4 Hz, J_2 = 1.6 Hz, 1H), 6.67 (d, *J* = 8.4 Hz, 1H), 5.22 (dd, J_1 = 11.2 Hz, J_2 = 4.8 Hz, 1H), 3.70 (s, 3H), 3.40 (d, *J* = 4.8 Hz, 1H), 3.25-3.19 (m, 1H), 2.38 (s, 3H); **^{13}C NMR (101 MHz, DMSO-*d$_6$*)** δ 169.6, 167.5, 155.7, 146.2, 144.1, 136.3, 135.6, 132.4, 131.1, 130.3,

128.2, 128.1, 127.7, 124.1, 119.4, 116.7, 53.3, 53.2, 33.6, 21.6; **HRMS (ESI)** m/z calcd for $C_{26}H_{24}N_3O_7S^+$ $(M+H)^+$ 522.13295, found 522.13330.

5.3 General procedure for the synthesis of *N*-tosylhydrazones used for eight-membered lactones

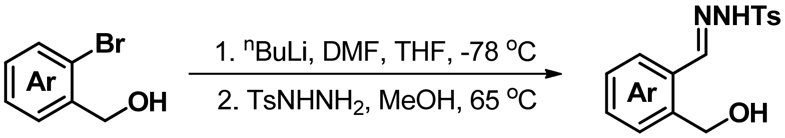

^{n}BuLi (2.5 equiv, 50 mmol) was added slowly to a solution of (2-bromoaryl)methanol (20 mmol) in 20 mL of dry THF at -78 ^{o}C. The resulting mixture was stirred at that temperature for 30 minutes and dry DMF (2.0 equiv) was added to the solution dropwise. After stirring at -78 ^{o}C for another 30 minutes, the reaction flask was taken out from the low-temperature bath and stirred for 10 minutes in an ice-bath. The conversion of starting into product could be checked by TLC. The reaction was then quenched by adding 10 mL of aq. solution of ammonium chloride. 20 mL of ethyl acetate was added and reaction mixture was stirred at room temperature for 15 minutes. It was then filtered via a short pad of celite and the filtrate was extracted by ethyl acetate (3 X 25 mL). The combined ethyl acetate layers were washed with brine, dried over anhydrous Na_2SO_4 and solvent was removed under reduced pressure. The residue thus obtained was dissolved in DCM and passed through a short pad of silica gel using DCM as eluent. The solvent was removed under reduced pressure. The residue was then dissolved in methanol (20 mL), $TsNHNH_2$ was added and the mixture was heated at 65 ^{o}C for 1 h. The reaction flask was then cooled to room

temperature during which white precipitate appeared (In case if precipitate didn't appeare petroleum ether could be added). The precipitate was then filtered, and washed with DCM to get pure *N*-tosylhydrzones.

***N*-(2-(hydroxymethyl)benzylidene)-4-methylbenzenesulfonohydrazide (S2w)**

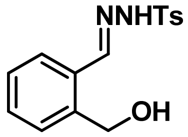

Yield 68%, white solid; R_f = 0.30 (petroleum ether : ethyl acetate = 1 : 1); **^{1}H NMR (400 MHz, DMSO-*d*$_6$)** δ 11.43 (s, 1H), 8.19 (s, 1H), 7.77 (d, *J* = 8.4 Hz, 2H), 7.59 (d, *J* = 7.6 Hz, 1H), 7.45-7.40 (m, 3H), 7.35 (t, *J* = 7.6 Hz, 1H), 7.28 (t, *J* = 7.2 Hz, 1H), 5.21 (t, *J* = 5.2 Hz, 1H), 4.54 (d, *J* = 5.2 Hz, 2H), 2.36 (s, 3H); **^{13}C NMR (101 MHz, DMSO-*d*$_6$)** δ 146.8, 144.0, 141.3, 136.6, 131.4, 130.2, 130.1, 128.4, 127.8, 127.7, 127.3, 61.7, 21.5.

***N*-(2-(hydroxymethyl)-4-methoxybenzylidene)-4-methylbenzenesulfonohydrazide (S2x)**

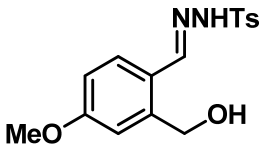

Yield 64%, white solid; R_f = 0.30 (petroleum ether : ethyl acetate = 1 : 2); **^{1}H NMR (400 MHz, DMSO-*d*$_6$)** δ 11.20 (br s, 1H), 8.08 (s, 1H), 7.76 (d, *J* = 8.0 Hz, 2H), 7.51 (d, *J* = 8.8 Hz, 1H), 7.40 (d, *J* = 8.4 Hz, 2H), 7.04 (d, *J* = 2.4 Hz, 1H), 6.84 (dd, J_1 = 8.4 Hz, J_2 = 2.4 Hz, 1H), 5.24 (br s, 1H), 4.53 (d, *J* = 2.8 Hz, 2H), 3.77 (s, 3H), 2.36 (s, 3H); **^{13}C NMR (101 MHz, DMSO-*d*$_6$)** δ 160.9, 147.1, 143.9, 143.5, 136.6, 130.1, 129.8, 127.8, 123.7, 113.3, 112.9, 61.7, 55.7, 21.5; **HRMS (ESI)** m/z calcd for $C_{16}H_{19}N_2O_4S^+$ $(M+H)^+$ 335.10600, found 335.10626.

N-(2-(hydroxymethyl)-5-methylbenzylidene)-4-methylbenzenesulfonohydrazide (S2y)

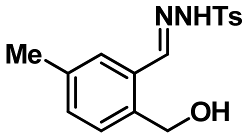

Yield 77%, white solid; R_f = 0.30 (petroleum ether : ethyl acetate = 1 : 1); **^{1}H NMR (600 MHz, DMSO-d_6)** δ 11.43 (s, 1H), 8.15 (s, 1H), 7.76 (d, J = 8.4 Hz, 2H), 7.41 (d, J = 8.4 Hz, 2H), 7.39 (s, 1H), 7.31 (d, J = 7.8 Hz, 1H), 7.16 (dd, J_1 = 7.8 Hz, J_2 = 1.2 Hz, 1H), 5.15 (t, J = 5.4 Hz, 1H), 4.49 (d, J = 5.4 Hz, 2 H), 2.35 (s, 3H), 2.28 (s, 3H); **^{13}C NMR (101 MHz, DMSO-d_6)** δ 146.9, 144.0, 138.5, 136.8, 136.6, 131.3, 130.8, 130.2, 128.6, 127.8, 127.7, 61.6, 21.5, 21.1; **HRMS (ESI)** m/z calcd for $C_{16}H_{19}N_2O_3S^+$ $(M+H)^+$ 319.11109, found 319.11136.

N-((6-(hydroxymethyl)benzo[d][1,3]dioxol-5-yl)methylene)-4-methylbenzenesulfonohydrazide (S2z)

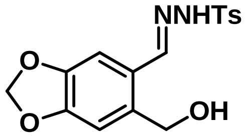

Yield 63%, white solid; R_f = 0.30 (petroleum ether : ethyl acetate = 1 : 2); **^{1}H NMR (600 MHz, DMSO-d_6)** δ 11.28 (s, 1H), 8.11 (s, 1H), 7.76 (d, J = 8.4 Hz, 2H), 7.41 (d, J = 7.8 Hz, 2H), 7.10 (s, 1H), 6.95 (s, 1H), 6.03 (s, 2H), 5.20 (t, J = 5.4 Hz, 1H), 4.44 (d, J = 5.4 Hz, 2 H), 2.36 (s, 3H); **^{13}C NMR (151 MHz, DMSO-d_6)** δ 149.1, 147.0, 146.0, 143.9, 136.9, 136.6, 130.1, 127.8, 125.4, 108.9, 105.5, 101.9, 61.1, 21.5; **HRMS (ESI)** m/z calcd for $C_{16}H_{17}N_2O_5S^+$ $(M+H)^+$ 349.08527, found 349.08563.

General procedure for the synthesis of lactones

General procedure for the synthesis of seven-membered lactones

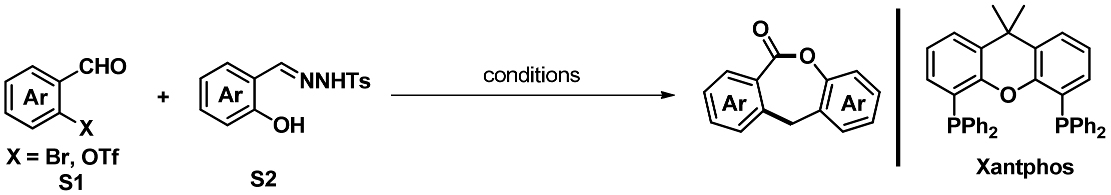

An oven-dried reaction tube containing a stirring bar was charged with $Pd(OAc)_2$ (5 mol%) or $[(\eta\text{-}C_3H_6)PdCl]_2$ (2.5 mol%), Xantphos (7.5 mol%), K_2CO_3 (3.0 equiv.), *N*-tosylhydrazone S**2** (0.4 mmol) and S**1** (if solid) (0.2 mmol). After evacuating and back filling with dry argon, the procedure was repeated for three times, solvent THF or dioxane (2.0 mL) and **S1** (if liquid) (0.2 mmol) were added via syringe. The mixture was stirred at 80 °C. When the reaction was completed, the crude mixture was cooled to room temperature. The mixture was filtered through a short pad of celite. After removing the solvent under reduced pressure, the residual was purified by column chromatography on silica gel.

General procedure for the synthesis of eight-membered lactones

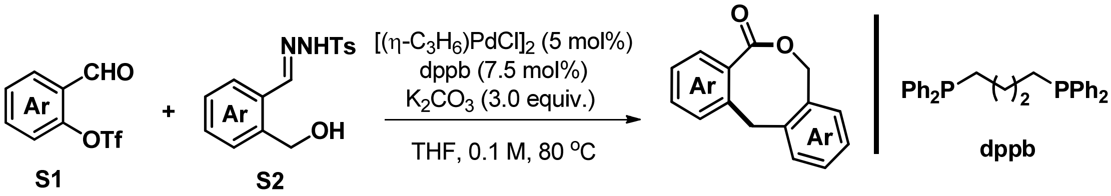

An oven-dried schlenk tube containing a stirring bar was charged with $[(\eta\text{-}C_3H_6)PdCl]_2$ (5 mol%), *N*-tosylhydrazone **S2** (0.4 mmol, 2 equiv), bis(diphenylphosphanyl)butane (dppb) (7.5 mol%), K_2CO_3 (3.0 equiv) and 2-formylaryl trifluoromethanesulfonate **S1** (0.2 mmol, in case it is solid). After

evacuating and back filling with dry argon, the procedure was repeated for three times. 2-formylaryl trifluoromethanesulfonate **S1** (0.2 mmol, in case it is liquid) and tetrahydrofuran (THF) (2.0 mL) were added *via* syringe. The mixture was stirred at 80 ^{o}C for 10 hours. The crude mixture was cooled to room temperature. The mixture was filtered through a short pad of celite. After removing the solvent under reduced pressure, the residual was purified by silica gel column chromatography to obtain the eight-membered lactone compound.

Dibenzo[*b*,*e*]oxepin-6(11*H*)-one (3)

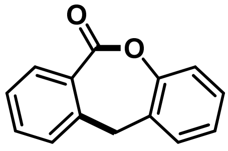

Yield 76% (32.0 mg) from *o*-bromobenzaldehyde for 10 h; Yield 80% (34.1 mg) from 2-formylphenyl trifluoromethanesulfonate for 9 h, white solid; R_f = 0.25 (petroleum ether : ethyl acetate = 50 : 1); **^{1}H NMR (400 MHz, CDCl$_3$)** δ 7.89 (d, J = 8.0 Hz, 1H), 7.45 (td, J_1 = 7.6 Hz, J_2 = 1.2 Hz, 1H), 7.33 (td, J_1 = 7.6 Hz, J_2 = 1.2 Hz, 1H), 7.27-7.19 (m, 4H), 7.14-7.09 (m, 1H), 4.00 (s, 2H); **^{13}C NMR (101 MHz, CDCl$_3$)** δ 166.1, 150.7, 142.6, 133.4, 132.7, 132.6, 128.2, 128.1, 128.0, 127.4, 127.1, 125.8, 120.7, 37.4.

9-Methyldibenzo[*b*,*e*]oxepin-6(11*H*)-one (4)

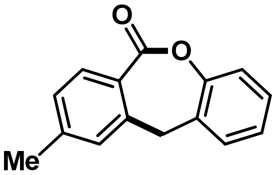

Yield 82% (37.0 mg) for 16 h, white solid; R_f = 0.25 (petroleum ether : ethyl acetate = 50 : 1); **^{1}H NMR (400 MHz, CDCl$_3$)** δ 7.79 (d, J = 8.0 Hz, 1H), 7.26-7.18 (m, 3H), 7.13-7.07 (m, 3H), 3.95 (s, 2H), 2.35 (s, 3H); **^{13}C NMR (101 MHz, CDCl$_3$)** δ 166.1, 150.8, 144.4, 142.5, 132.9, 132.7, 128.2, 128.1, 128.0, 127.8, 125.8, 125.1, 120.7,

37.4, 21.5; **HRMS (ESI)** m/z calcd for $C_{15}H_{13}O_2^+$ $(M+H)^+$ 225.09101, found 225.09140.

9-Methoxydibenzo[*b*,*e*]oxepin-6(11*H*)-one (5)

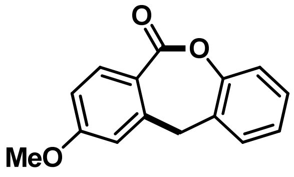

Yield 84% (40.5 mg) from 2-bromo-4-methoxybenzaldehyde for 16 h; Yield 65% (31.6 mg) from 2-formyl-5-methoxyphenyl trifluoromethanesulfonate for 6 h, white solid; R_f = 0.20 (petroleum ether : ethyl acetate = 50 : 1); **^{1}H NMR (400 MHz, $CDCl_3$)** δ 7.88 (d, *J* = 8.8 Hz, 1H), 7.26-7.18 (m, 3H), 7.14-7.09 (m, 1H), 6.81 (dd, J_1 = 8.8 Hz, J_2 =2.4 Hz, 1H), 6.75 (d, *J* = 2.4 Hz, 1H), 3.96 (s, 2H), 3.83 (s, 3H); **^{13}C NMR (101 MHz, $CDCl_3$)** δ 165.8, 163.4, 150.9, 144.7, 135.3, 132.4, 128.12, 128.09, 125.7, 120.7, 120.1, 112.61, 112.57, 55.4, 37.8; **HRMS (ESI)** m/z calcd for $C_{15}H_{13}O_3^+$ $(M+H)^+$ 241.08592, found 241.08603.

9-Fluorodibenzo[*b*,*e*]oxepin-6(11*H*)-one (6)

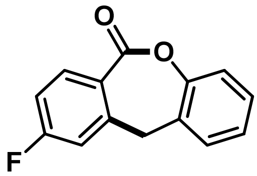

Yield 80% (38.2 mg) for 11 h, white solid; R_f = 0.25 (petroleum ether : ethyl acetate = 50 : 1); **^{1}H NMR (400 MHz, $CDCl_3$)** δ 7.94-7.90 (m, 1H), 7.31-7.22 (m, 5H), 7.27-7.22 (m, 3H), 7.16-7.12 (m, 1H), 7.04-6.96 (m, 2H), 3.99 (s, 2H); **^{13}C NMR (101 MHz, $CDCl_3$)** δ 165.28 (d, *J* = 257.2 Hz), 165.1, 150.7, 145.4 (d, *J* = 8.7 Hz), 135.7, 135.6, 131.9, 128.3 (d, *J* = 18.7 Hz), 126.0, 124.3 (d, *J* = 3.1 Hz), 120.8, 114.7 (d, *J* = 21.8 Hz), 114.3 (d, *J* = 22.4 Hz), 37.3 (d, *J* = 1.0 Hz); **^{19}F NMR (376 MHz, $CDCl_3$)** δ -104.4; **HRMS (ESI)** m/z calcd for $C_{14}H_{10}FO_2^+$ $(M+H)^+$ 229.06593, found 229.06566.

9-Chlorodibenzo[*b*,*e*]oxepin-6(11*H*)-one (7)

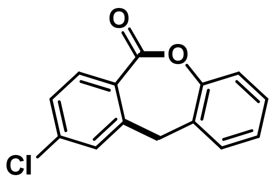

Yield 67% (32.8 mg) for 10 h, white solid; R_f = 0.25 (petroleum ether : ethyl acetate = 50 : 1); **^{1}H NMR (400 MHz, $CDCl_3$)** δ 7.83 (d, *J* = 8.0 Hz, 1H), 7.31-7.22 (m, 5H), 7.17-7.12 (m, 1H), 3.97 (s, 2H); **^{13}C NMR (101 MHz, $CDCl_3$)** δ 165.1, 150.6, 144.0, 139.5, 134.3, 131.8, 128.4, 128.3, 127.8, 127.3, 126.5, 126.1, 120.8, 37.1; **HRMS (ESI)** m/z calcd for $C_{14}H_{10}ClO_2^+$ $(M+H)^+$ 245.03638, found 245.03671.

8-Methyldibenzo[*b*,*e*]oxepin-6(11*H*)-one (8)

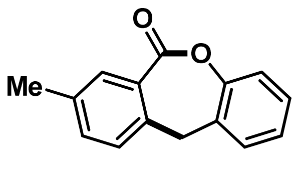

Yield 73% (33.0 mg), white solid; R_f = 0.25 (petroleum ether : ethyl acetate = 50 : 1); **^{1}H NMR (400 MHz, $CDCl_3$)** δ 7.69 (s, 1H), 7.26-7.07 (m, 6H), 3.94 (s, 2H), 2.31 (s, 3H); **^{13}C NMR (101 MHz, $CDCl_3$)** δ 166.3, 150.7, 139.7, 137.3, 134.1, 132.98, 132.92, 128.04, 127.96, 127.7, 127.1, 125.8, 120.6, 36.9, 20.7. **HRMS (ESI)** m/z calcd for $C_{15}H_{13}O_2^+$ $(M+H)^+$ 225.09101, found 225.09117.

8-Methoxydibenzo[*b*,*e*]oxepin-6(11*H*)-one (9)

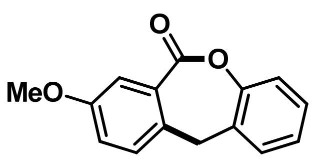

Yield 79% (37.9 mg) from 2-bromo-5-methoxybenz aldehyde for 12 h; Yield 71% (34.2 mg) from 2-formyl-4-methoxy phenyltrifluoromethanesulfonate for 18 h, white solid; R_f = 0.20 (petroleum ether : ethyl acetate = 50 : 1); **^{1}H NMR (400 MHz, $CDCl_3$)** δ 7.40 (d, *J* = 1.4 Hz, 1H), 7.24-7.08 (m, 5H), 7.00-6.98 (m, 1H), 3.93 (s, 2H), 3.78 (s, 3H); **^{13}C NMR (101 MHz, $CDCl_3$)** δ 166.0, 158.7, 150.6, 135.1,

133.2, 128.7, 128.4, 128.0, 125.8, 120.6, 120.3, 116.3, 55.5, 36.5; **HRMS (ESI)** m/z calcd for $C_{15}H_{13}O_3^+$ $(M+H)^+$ 241.08592, found 241.08545.

8-Fluorodibenzo[*b*,*e*]oxepin-6(11*H*)-one (10)

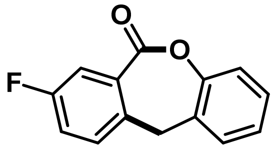

Yield 65% (30.0 mg) for 14 h, white solid; R_f = 0.30 (petroleum ether : ethyl acetate = 50 : 1); **^{1}H NMR (400 MHz, $CDCl_3$)** δ 7.58 (dd, J_1 = 8.8 Hz, J_2 = 2.8 Hz, 1H), 7.26-7.21 (m, 4H), 7.17-7.11 (m, 2H), 3.97 (s, 2H); **^{13}C NMR (101 MHz, $CDCl_3$)** δ 164.8 (d, *J* = 2.6 Hz), 161.6 (d, *J* = 248.0 Hz), 150.4, 138.6 (d, *J* = 3.4 Hz), 132.5, 129.7, 129.6, 129.0 (d, *J* = 7.6 Hz), 128.2 (d, *J* = 16.9 Hz), 126.0, 120.8, 120.3 (d, *J* = 21.5 Hz), 119.3 (d, *J* = 23.7 Hz), 36.6; **^{19}F NMR (376 MHz, $CDCl_3$)** δ -114.3; **HRMS (ESI)** m/z calcd for $C_{14}H_{10}FO_2^+$ $(M+H)^+$ 229.06593, found 229.06554.

8-Chlorodibenzo[*b*,*e*]oxepin-6(11*H*)-one (11)

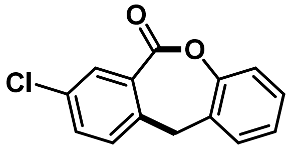

Yield 65% (32.1 mg) for 10 h, white solid; R_f = 0.25 (petroleum ether : ethyl acetate = 50 : 1); **^{1}H NMR (400 MHz, $CDCl_3$)** δ 7.85 (d, *J* = 2.0 Hz, 1H), 7.40 (dd, J_1 = 8.0 Hz, J_2 = 2.0 Hz, 1H), 7.26-7.20 (m, 4H), 7.16-7.11 (m, 1H), 3.97 (s, 2H); **^{13}C NMR (101 MHz, $CDCl_3$)** δ 164.8, 150.4, 140.9, 133.3, 133.2, 132.3, 132.1, 129.5, 128.6, 128.4, 128.2, 126.1, 120.7, 36.7; **HRMS (ESI)** m/z calcd for $C_{14}H_{10}ClO_2^+$ $(M+H)^+$ 245.03638, found 245.03674.

8,9-Dimethoxydibenzo[*b*,*e*]oxepin-6(11*H*)-one (12)

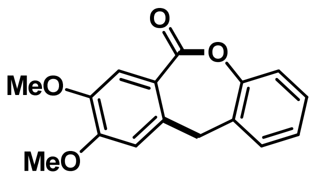

Yield 67% (36.0 mg) for 9 h, white solid; R_f = 0.25 (petroleum ether : ethyl acetate = 5 : 1); **^{1}H NMR (400 MHz, $CDCl_3$)** δ 7.41 (s, 1H), 7.27-7.20 (m, 3H), 7.15-7.10 (m, 1H), 6.72 (s, 1H), 3.95 (s, 5H), 3.87 (s, 3H); **^{13}C NMR (101 MHz, $CDCl_3$)** δ 165.8, 153.0, 150.9, 148.0, 137.1, 133.0, 128.0, 127.9, 125.7, 120.7, 119.4, 114.9, 109.8, 56.09, 56.07, 37.2; **HRMS (ESI)** m/z calcd for $C_{16}H_{15}O_4^+$ $(M+H)^+$ 271.09649, found 271.09689.

[1,3]Dioxolo[4',5':4,5]benzo[1,2-*e*]benzo[*b*]oxepin-5(11*H*)-one (13)

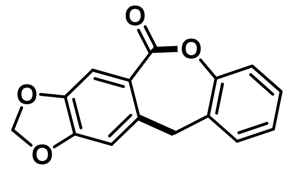

Yield 75% (38.4 mg) for 12 h, white solid; R_f = 0.25 (petroleum ether : ethyl acetate = 50 : 1); **^{1}H NMR (400 MHz, $CDCl_3$)** δ 7.31 (s, 1H), 7.24-7.21 (m, 3H), 7.14-7.09 (m, 1H), 6.70 (s, 1H), 5.98 (s, 2H), 3.89 (s, 2H); **^{13}C NMR (101 MHz, $CDCl_3$)** δ 165.4, 151.7, 150.8, 146.9, 138.9, 132.8, 128.1, 127.9, 125.8, 121.0, 120.6, 112.1, 107.4, 102.0, 37.2; **HRMS (ESI)** m/z calcd for $C_{15}H_{11}O_4^+$ $(M+H)^+$ 255.06519, found 255.06525.

Benzo[*b*]naphtho[1,2-*e*]oxepin-7(13*H*)-one (14)

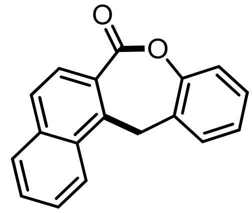

Yield 55% (28.9 mg) for 12 h, white solid; R_f = 0.30 (petroleum ether : ethyl acetate = 20 : 1); **^{1}H NMR (400 MHz, $CDCl_3$)** δ 8.39 (d, *J* = 8.4 Hz, 1H), 7.87-7.82 (m, 2H), 7.77 (d, *J* = 8.8 Hz, 1H), 7.69-7.59 (m, 2H), 7.35 (dd, J_1 = 7.6 Hz, J_2 = 1.2 Hz, 1H), 7.26-7.18 (m, 2H), 7.09 (td, J_1 = 7.2 Hz, J_2 = 1.6 Hz, 1H), 4.44 (s, 2H); **^{13}C NMR (101 MHz, $CDCl_3$)** δ

166.8, 151.2, 140.7, 135.6, 132.3, 129.0, 128.8, 128.2, 128.1, 128.0, 127.6, 127.4, 127.2, 125.7, 125.3, 124.0, 120.3, 30.4; **HRMS (ESI)** m/z calcd for $C_{18}H_{13}O_2^+$ $(M+H)^+$ 261.09101, found 261.09131.

4-Methyldibenzo[*b*,*e*]oxepin-6(11*H*)-one (15)

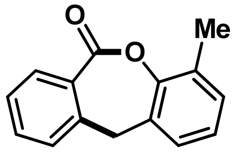

Yield 74% (33.3 mg) for 22 h, white solid; R_f = 0.25 (petroleum ether : ethyl acetate = 50 : 1); **^{1}H NMR (400 MHz, $CDCl_3$)** δ 7.89 (d, *J* = 8.0 Hz, 1H), 7.44 (t, *J* = 7.6 Hz, 1H), 7.31 (t, *J* = 7.6 Hz, 1H), 7.25 (d, *J* = 7.6 Hz, 1H), 7.08-6.98 (m, 3H), 3.96 (s, 2H), 2.37 (s, 3H); **^{13}C NMR (101 MHz, $CDCl_3$)** δ 165.9, 149.1, 142.9, 133.3, 132.8, 132.6, 130.0, 129.7, 128.0, 127.3, 127.1, 125.6, 125.5, 37.5, 16.4; **HRMS (ESI)** m/z calcd for $C_{15}H_{13}O_2^+$ $(M+H)^+$ 225.09101, found 225.09082.

4-Fluorodibenzo[*b*,*e*]oxepin-6(11*H*)-one (16)

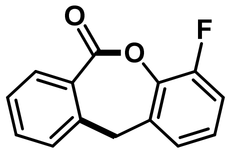

Yield 58% (26.6 mg) for 5 h, white solid; R_f = 0.20 (petroleum ether : ethyl acetate = 50 : 1); **^{1}H NMR (400 MHz, $CDCl_3$)** δ 7.91 (d, *J* = 8.0 Hz, 1H), 7.49 (td, J_1 = 7.6 Hz, J_2 = 1.2 Hz, 1H), 7.36 (t, *J* = 7.6 Hz, 1H), 7.27 (d, *J* = 7.6 Hz, 1H), 7.09-6.88 (m, 3H), 4.03 (s, 2H); **^{13}C NMR (101 MHz, $CDCl_3$)** δ 164.9, 153.1 (d, *J* = 252.8 Hz), 142.1, 138.4 (d, *J* = 11.5 Hz), 135.3, 133.7, 133.0, 127.6, 127.5 (d, *J* = 38.6 Hz), 126.2 (d, *J* = 7.6 Hz), 122.9 (d, *J* = 3.7 Hz), 115.4 (d, *J* = 18.7 Hz), 37.3 (d, *J* = 2.3 Hz); **^{19}F NMR (376 MHz, $CDCl_3$)** δ -128.9; **HRMS (ESI)** m/z calcd for $C_{14}H_{10}FO_2^+$ $(M+H)^+$ 229.06593, found 229.06569.

4-Chlorodibenzo[*b*,*e*]oxepin-6(11*H*)-one (17)

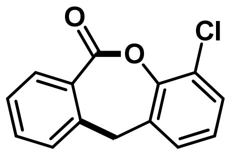

Yield 60% (29.4 mg) for 6 h, white solid; R_f = 0.20 (petroleum ether : ethyl acetate = 50 : 1); **^{1}H NMR (400 MHz, $CDCl_3$)** δ 7.92 (d, *J* = 7.6 Hz, 1H), 7.48 (t, *J* = 7.6 Hz, 1H), 7.36 (t, *J* = 7.6 Hz, 1H), 7.29-7.26 (m, 2H), 7.15 (d, *J* = 7.6 Hz, 1H), 7.06-7.03 (m, 1H), 4.02 (s, 2H); **^{13}C NMR (101 MHz, $CDCl_3$)** δ 164.5, 146.8, 142.1, 134.8, 133.7, 132.9, 129.0, 127.7, 127.4, 127.3, 126.4, 126.3, 126.0, 37.5; **HRMS (ESI)** m/z calcd for $C_{14}H_{10}ClO_2^+$ $(M+H)^+$ 245.03638, found 245.03621.

3-Fluorodibenzo[*b*,*e*]oxepin-6(11*H*)-one (18)

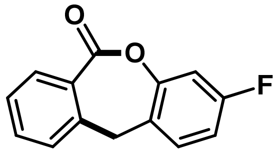

Yield 60% (27.7 mg) for 10 h, white solid; R_f = 0.20 (petroleum ether : ethyl acetate = 50 : 1); **^{1}H NMR (400 MHz, $CDCl_3$)** δ 7.89 (dd, J_1 = 8.0, J_2 = 1.2 Hz, 1H), 7.48 (td, J_1 = 7.6 Hz, J_2 = 1.6 Hz, 1H), 7.34 (td, J_1 = 7.6 Hz, J_2 = 1.2 Hz, 1H), 7.26-7.19 (m, 2H), 6.97 (dd, J_1 = 8.8 Hz, J_2 = 2.4 Hz, 1H), 6.83 (td, J_1 = 8.4 Hz, J_2 = 2.8 Hz, 1H), 3.97 (s, 2H); **^{13}C NMR (101 MHz, $CDCl_3$)** δ 165.4, 161.8 (d, *J* = 248.1 Hz), 151.2 (d, *J* = 11.6 Hz), 142.4, 133.6, 132.8, 128.8 (d, *J* = 9.3 Hz), 128.5 (d, *J* = 3.5 Hz), 127.7, 127.6, 127.1, 112.6 (d, *J* = 21.3 Hz), 108.7 (d, *J* = 24.9 Hz), 36.8; **^{19}F NMR (376 MHz, $CDCl_3$)** δ -113.2; **HRMS (ESI)** m/z calcd for $C_{14}H_{10}FO_2^+$ $(M+H)^+$ 229.06593, found 229.06551.

3-Chlorodibenzo[*b*,*e*]oxepin-6(11*H*)-one (19)

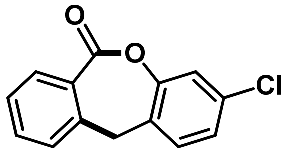

Yield 66% (32.3mg) for 6 h, white solid; R_f = 0.20 (petroleum ether : ethyl acetate = 50 : 1); **^{1}H NMR (400 MHz, $CDCl_3$)** δ 7.88 (d, *J* = 8.0 Hz, 1H), 7.47 (t, *J* = 7.6 Hz, 1H), 7.34 (t, *J* = 7.6 Hz, 1H), 7.26-7.18 (m, 3H), 7.09 (dd, J_1 = 8.0, J_2 =2.0 Hz, 1H), 3.97 (s, 2H); **^{13}C NMR (101 MHz, $CDCl_3$)** δ 165.3, 151.0, 142.1, 133.6, 133.2, 132.8, 131.2, 128.9, 127.72, 127.66, 127.1, 125.9, 121.2, 36.9; **HRMS (ESI)** m/z calcd for $C_{14}H_{10}ClO_2^+$ $(M+H)^+$ 245.03638, found 245.03674.

2-Methyldibenzo[*b*,*e*]oxepin-6(11*H*)-one (20)

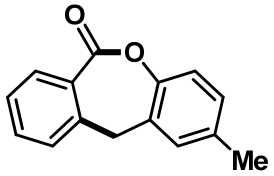

Yield 82% (37.0 mg) for 6 h, white solid; R_f = 0.25 (petroleum ether : ethyl acetate = 50 : 1); **^{1}H NMR (400 MHz, $CDCl_3$)** δ 7.88 (d, *J* = 7.6 Hz, 1H), 7.45 (t, *J* = 7.2 Hz, 1H), 7.31 (t, *J* = 7.6 Hz, 1H), 7.25 (d, *J* = 7.6 Hz, 1H), 7.10 (d, *J* = 8.0 Hz, 1H), 7.05 (s, 1H), 7.00 (d, *J* = 8.4 Hz, 1H), 3.94 (s, 2H), 2.29 (s, 3H); **^{13}C NMR (101 MHz, $CDCl_3$)** δ 166.4, 148.5, 142.6, 135.6, 133.3, 132.7, 132.3, 128.6, 128.5, 128.1, 127.4, 127.1, 120.3, 37.4, 20.6.

2-Methoxydibenzo[b,e]oxepin-6(11H)-one (21)

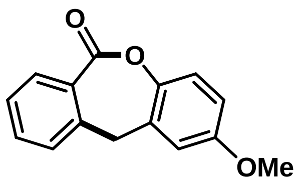

Yield 53% (25.5 mg) for 6 h, white solid; R_f = 0.30 (petroleum ether : ethyl acetate = 10 : 1); **^{1}H NMR (400 MHz, $CDCl_3$)** δ 7.89 (dd, J_1 = 7.6 Hz, J_2 = 1.2 Hz, 1H), 7.46 (td, J_1 = 7.6 Hz, J_2 = 1.6 Hz, 1H), 7.33 (td, J_1 = 7.6 Hz, J_2 = 1.2 Hz, 1H), 7.25 (d, *J* =

6.4 Hz, 1H), 7.14 (d, J = 8.8 Hz, 1H), 6.77 (d, J = 2.8 Hz, 1H), 6.71 (dd, J_1 = 8.8 Hz, J_2 = 3.2 Hz, 1H), 3.95 (s, 2H), 3.77 (s, 3H); **^{13}C NMR (101 MHz, CDCl$_3$)** δ 166.5, 157.1, 144.4, 142.4, 133.8, 133.3, 132.8, 128.1, 127.5, 127.2, 121.5, 113.4, 112.4, 55.6, 37.7; **HRMS (ESI)** m/z calcd for $C_{15}H_{13}O_3^+$ $(M+H)^+$ 241.08592, found 241.08601.

2-Fluorodibenzo[*b*,*e*]oxepin-6(11*H*)-one (22)

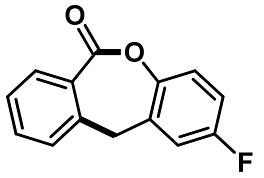

Yield 86% (39.3 mg) for 3 h, white solid; R_f = 0.25 (petroleum ether : ethyl acetate = 50 : 1); **^{1}H NMR (400 MHz, CDCl$_3$)** δ 7.90 (dd, J_1 = 7.6 Hz, J_2 = 1.2 Hz, 1H), 7.48 (td, J_1= 7.6 Hz,, J_2 = 1.2 Hz, 1H), 7.35 (td, J_1= 7.6 Hz, J_2= 0.8 Hz, 1H), 7.26 (d, J = 7.6 Hz, 1H), 7.18 (dd, J_1 = 8.8 Hz, J_2 = 4.8 Hz, 1H), 6.97 (dd, J_1 = 8.4 Hz, J_2 = 3.2 Hz, 1H), 6.89 (td, J_1= 8.4 Hz, J_2 = 3.2 Hz, 1H), 3.97 (s, 2H); **^{13}C NMR (101 MHz, CDCl$_3$)** δ165.7, 159.8 (d, J = 246.5 Hz), 146.7 (d, J = 2.9 Hz), 141.9, 134.5 (d, J = 8.1 Hz), 133.5, 132.9, 127.8, 127.7, 127.2, 122.1 (d, J = 8.9 Hz), 114.8 (d, J = 23.9 Hz), 114.44 (d, J = 23.3 Hz), 37.30 (d, J = 0.9 Hz); **^{19}F NMR (376 MHz, CDCl$_3$)** δ -116.6; **HRMS (ESI)** m/z calcd for $C_{14}H_{10}FO_2^+$ $(M+H)^+$ 229.06593, found 229.06558.

2-Chlorodibenzo[*b*,*e*]oxepin-6(11*H*)-one (23)

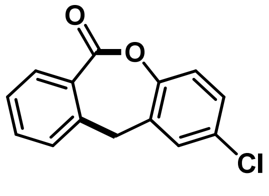

Yield 77% (37.6 mg) for 3 h, white solid; R_f = 0.25 (petroleum ether : ethyl acetate = 50 : 1); **^{1}H NMR (400 MHz, CDCl$_3$)** δ 7.89 (d, J = 7.6 Hz, 1H), 7.48 (td, J_1 = 7.2 Hz, J_2 = 0.8 Hz, 1H), 7.35 (t, J = 7.6 Hz, 1H), 7.27-7.14 (m, 4H), 3.96 (s, 2H); **^{13}C NMR (101 MHz,**

$CDCl_3$) δ 165.5, 149.2, 141.7, 134.2, 133.6, 132.8, 130.8, 128.0, 127.72, 127.67, 127.2, 122.0, 37.1; **HRMS (ESI)** m/z calcd for $C_{14}H_{10}ClO_2^+$ $(M+H)^+$ 245.03638, found 245.03653.

2-Bromodibenzo[*b*,*e*]oxepin-6(11*H*)-one (24)

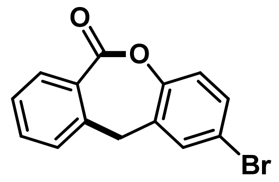

Yield 62% (35.8 mg) for 5 h, white solid; R_f = 0.20 (petroleum ether : ethyl acetate = 50 : 1); **^1H NMR (400 MHz, $CDCl_3$)** δ 7.89 (dd, J_1 = 7.6 Hz, J_2 = 1.0 Hz, 1H), 7.48 (td, J_1 = 7.2 Hz, J_2 = 1.2 Hz, 1H), 7.41-7.25 (m, 4H), 7.10 (d, J = 8.4 Hz, 1H), 3.96 (s, 2H); **^{13}C NMR (101 MHz, $CDCl_3$)** δ 165.4, 149.8, 141.8, 134.7, 133.6, 132.8, 131.0, 130.9, 127.8, 127.7, 127.2, 122.4, 118.5, 37.1; **HRMS (ESI)** m/z calcd for $C_{14}H_{10}BrO_2^+$ $(M+H)^+$ 288.98587, found 288.98636.

1-Methoxydibenzo[*b*,*e*]oxepin-6(11*H*)-one (25)

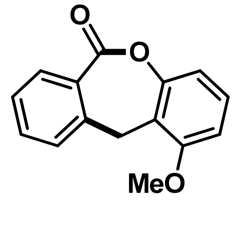

Yield 58% (28.1 mg) for 4 h, white solid; R_f = 0.30 (petroleum ether : ethyl acetate = 10 : 1); **^1H NMR (400 MHz, $CDCl_3$)** δ 7.90 (dd, J_1 = 7.6 Hz, J_2 = 0.8 Hz, 1H), 7.44 (td, J_1 = 7.2 Hz, J_2 = 1.2 Hz, 1H), 7.31 (td, J_1 = 7.6 Hz, J_2 = 0.8 Hz, 1H), 7.24 (d, J = 7.6 Hz, 1H), 7.05 (t, J = 7.6 Hz, 1H), 6.83-6.80 (m, 2H), 3.98 (s, 2H), 3.86 (s, 3H); **^{13}C NMR (101 MHz, $CDCl_3$)** δ 165.7, 150.7, 142.8, 139.6, 134.4, 133.3, 132.8, 128.0, 127.4, 127.2, 126.1, 119.3, 111.1, 56.0, 37.3; **HRMS (ESI)** m/z calcd for $C_{15}H_{13}O_3^+$ $(M+H)^+$ 241.08592, found 241.08583.

1-Fluorodibenzo[*b*,*e*]oxepin-6(11*H*)-one (26)

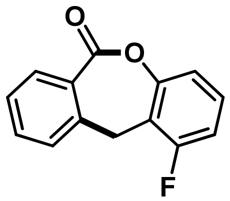

Yield 64% (29.4 mg) for 10 h, white solid; R_f = 0.25 (petroleum ether : ethyl acetate = 50 : 1); **^{1}H NMR (400 MHz, $CDCl_3$)** δ 7.89 (dd, J_1 = 8.0 Hz, J_2 = 1.2 Hz, 1H), 7.48 (td, J_1 = 7.6 Hz, J_2 = 1.2 Hz, 1H), 7.37-7.30 (m, 2H), 7.16 (td, J_1 = 8.0 Hz, J_2 = 6.4 Hz, 1H), 7.04 (d, J = 8.4 Hz, 1H), 6.91 (t, J = 8.8 Hz, 1H), 4.06 (s, 2H); **^{13}C NMR (101 MHz, $CDCl_3$)** δ 165.4, 158.96 (d, J = 247.5 Hz), 151.8 (d, J = 5.9 Hz), 141.6, 133.6, 132.9, 128.2, 127.9 (d, J = 9.7 Hz), 127.7, 127.6, 120.8 (d, J = 20.3 Hz), 116.4 (d, J = 3.5 Hz), 112.6 (d, J = 22.5 Hz), 28.4 (d, J = 3.9 Hz); **^{19}F NMR (376 MHz, $CDCl_3$)** δ -119.6; **HRMS (ESI)** m/z calcd for $C_{14}H_{10}FO_2^+$ $(M+H)^+$ 229.06593, found 229.06546.

1-Chlorodibenzo[b,e]oxepin-6(11H)-one (27)

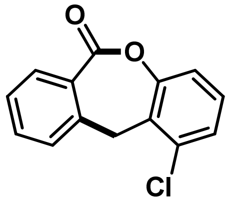

Yield 59% (29.1 mg) for 10 h, white solid; R_f = 0.25 (petroleum ether : ethyl acetate = 50 : 1); **^{1}H NMR (400 MHz, $CDCl_3$)** δ 7.89 (d, J = 7.6 Hz, 1H), 7.49 (t, J = 7.6 Hz, 1H), 7.37-7.33 (m, 2H), 7.21-7.09 (m, 3H), 4.22 (s, 2H); **^{13}C NMR (101 MHz, $CDCl_3$)** δ 165.2, 151.6, 141.5, 133.7, 132.7, 132.3, 131.4, 128.0, 127.8, 127.5, 126.6, 119.5, 32.8; **HRMS (ESI)** m/z calcd for $C_{14}H_{10}ClO_2^+$ $(M+H)^+$ 245.03638, found 245.03622.

2-((Trimethylsilyl)ethynyl)dibenzo[*b*,*e*]oxepin-6(11*H*)-one (28)

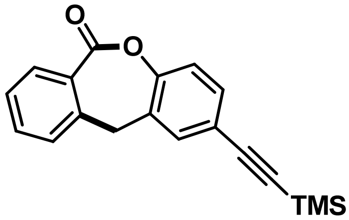

Yield 67% (41.3 mg) from 2-bromobenzaldehyde for 5 h; Yield 86% (52.6 mg) from 2-formylphenyl trifluoromethanesulfonate for 5 h, white solid; R_f = 0.50

(petroleum ether : ethyl acetate = 100 : 1); **^{1}H NMR (600 MHz, $CDCl_3$, TMS)** δ 7.86 (dd, J_1 = 7.8 Hz, J_2 =1.8 Hz, 1H), 7.45 (td, J_1 = 7.8 Hz, J_2 =1.2 Hz, 1H), 7.38 (d, J = 2.4 Hz, 1H), 7.34-7.29 (m, 2H), 7.23 (d, J = 7.8 Hz, 1H), 7.14 (d, J = 8.4 Hz, 1H), 3.95 (s, 2H), 0.23 (s, 9H); **^{13}C NMR (151 MHz, $CDCl_3$)** δ 165.5, 150.6, 142.0, 133.5, 132.7, 132.7, 131.8, 131.7, 127.8, 127.6, 127.2, 120.8, 120.7, 103.7, 94.8, 37.1, -0.2. **HRMS (ESI)** m/z calcd for $C_{19}H_{19}O_2Si^+$ $(M+H)^+$ 307.11488, found 307.11478.

2-(Phenylethynyl)dibenzo[*b*,*e*]oxepin-6(11*H*)-one (29)

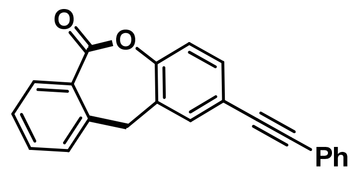

Yield 81% (50.3 mg) for 6 h, white solid; R_f = 0.50 (petroleum ether : ethyl acetate = 25 : 1); **^{1}H NMR (400 MHz, $CDCl_3$)** δ 7.89 (d, J = 7.2 Hz, 1H), 7.51-7.44 (m, 4H), 7.39-7.33 (m, 5H), 7.26 (d, J = 7.2 Hz, 1H), 7.20 (d, J = 8.4 Hz, 1H), 3.99 (s, 2H); **^{13}C NMR (100 MHz, $CDCl_3$)** δ 165.7, 150.4, 142.0, 133.6, 132.8, 132.7, 131.5, 131.4, 131.3, 128.43, 128.35, 127.9, 127.6, 127.2, 122.8, 120.93, 120.90, 89.8, 88.1, 37.2. **HRMS (ESI)** m/z calcd for $C_{22}H_{15}O_2^+$ $(M+H)^+$ 311.10666, found 311.10690.

(S)-3-methoxy-11-phenyldibenzo[b,e]oxepin-6(11H)-one (30)

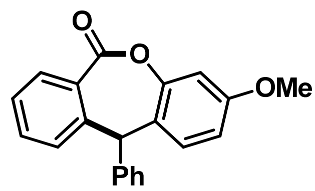

Yield 42% (26.7 mg) for 10 h, colorless oil; R_f = 0.30 (petroleum ether : ethyl acetate = 50 : 1); **^{1}H NMR (400 MHz, $CDCl_3$)** δ 7.83-7.81 (m, 2H), 7.54-7.47 (m, 4H), 7.40-7.29 (m, 3H), 6.84-6.82 (m, 1H), 6.72 (s, 1H), 6.34-6.32 (m, 2H), 3.68 (s, 3H); **^{13}C NMR (101 MHz, $CDCl_3$)** δ 160.6, 150.8, 146.4, 136.7, 135.4, 129.30, 129.27, 128.7, 128.6, 127.8, 124.8, 122.0, 121.9, 120.0, 106.2, 102.5, 100.9, 88.4, 55.2.

Methyl 6-oxo-6,11-dihydrodibenzo[*b*,*e*]oxepine-2-carboxylate (31)

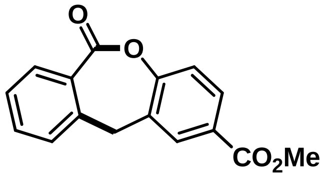

Yield 37% (20.2 mg) from 2-bromobenzaldehyde for 5 h; Yield 37% (19.8 mg) from 2-formylphenyl trifluoromethanesulfonate for 8 h, white solid; R_f = 0.25 (petroleum ether : ethyl acetate = 10 : 1); **^{1}H NMR (400 MHz, $CDCl_3$)** δ 7.98 (d, *J* = 1.6 Hz, 1H), 7.93-7.88 (m, 2H), 7.49 (td, J_1 = 7.6 Hz, J_2 = 1.2 Hz, 1H), 7.36 (t, *J* = 7.6 Hz, 1H), 7.30-7.27 (m, 2H), 4.06 (s, 2H), 3.90 (s, 3H); **^{13}C NMR (101 MHz, $CDCl_3$)** δ 166.0, 165.2, 154.1, 141.9, 133.7, 132.8, 132.7, 129.9, 127.71, 127.69, 127.6, 127.3, 120.9, 52.3, 37.2; **HRMS (ESI)** m/z calcd for $C_{16}H_{13}O_4^+$ $(M+H)^+$ 269.08084, found 269.08090.

N-(6-oxo-6,11-dihydrodibenzo[b,e]oxepin-2-yl)acetamide (32)

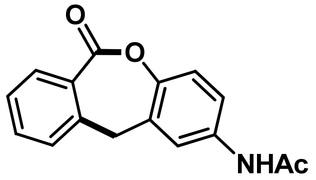

Yield 44% (23.5 mg) for 6 h, white solid; R_f = 0.30 (petroleum ether : ethyl acetate = 1 : 1); **^{1}H NMR (400 MHz, DMSO-d_6)** δ 10.03 (s, 1H), 7.76 (d, *J* = 7.2 Hz, 1H), 7.71 (d, *J* = 2.4 Hz, 1H), 7.57 (t, *J* = 7.2 Hz, 1H), 7.48 (d, *J* = 7.6 Hz, 1H), 7.40 (t, *J* = 7.6 Hz, 1H), 7.34 (dd, J_1 = 8.8 Hz, J_2 = 2.4 Hz, 1H), 7.18 (d, *J* = 8.8 Hz, 1H), 4.05 (s, 2H), 2.03 (s, 3H); **^{13}C NMR (101 MHz, DMSO-d_6)** δ 168.8, 165.9, 145.9, 143.4, 137.5, 134.1, 133.9, 132.6, 128.2, 128.04, 127.98, 121.1, 119.1, 118.8, 36.6, 24.4; **HRMS (ESI)** m/z calcd for $C_{16}H_{14}NO_3^+$ $(M+H)^+$ 268.09682, found 268.09711.

1-Isopropyl-4-methyldibenzo[b,e]oxepin-6(11H)-one (33)

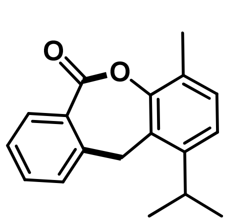

Yield 52% (27.8 mg) for 6 h, colorless oil; R_f = 0.30 (petroleum ether : ethyl acetate = 50 : 1); **^{1}H NMR (400 MHz, CDCl$_3$)** δ 7.91 (d, *J* = 7.6 Hz, 1H), 7.46 (td, J_1 = 7.6, J_2 = 1.2 Hz, 1H), 7.34-7.26 (m, 2H), 7.02 (m, 2H), 4.07 (s, 2H), 3.32 (hept, *J* = 6.8 Hz, 1H), 2.35 (s, 3H), 1.26 (d, *J* = 7.2 Hz, 6H); **^{13}C NMR (101 MHz, CDCl$_3$)** δ 166.1, 149.3, 143.3, 133.4, 133.3, 132.6, 130.9, 129.0, 128.2, 127.4, 127.3, 127.2, 122.1, 31.8, 29.2, 23.7, 16.3; **HRMS (ESI)** m/z calcd for $C_{18}H_{19}O_2^+$ $(M+H)^+$ 267.13796, found 267.13745.

4-Isopropyl-1-methyldibenzo[*b*,*e*]oxepin-6(11*H*)-one (34)

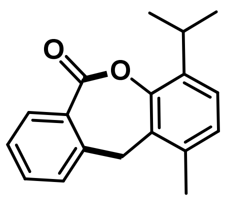

Yield 50% (26.8 mg) for 11 h, colorless oil; R_f = 0.30 (petroleum ether : ethyl acetate = 50 : 1); **^{1}H NMR (400 MHz, CDCl$_3$)** δ 7.90 (dd, J_1 = 7.6 Hz, J_2 = 1.0 Hz, 1H), 7.45 (td, J_1 = 7.6, J_2 = 1.2 Hz, 1H), 7.34-7.27 (m, 2H), 7.02 (d, *J* = 7.6 Hz, 1H), 6.95 (d, *J* = 8.0 Hz, 1H), 4.01 (s, 2H), 3.46 (hept, *J* = 6.8 Hz, 1H), 2.41 (s, 3H), 1.22 (d, *J* = 6.8 Hz, 6H); **^{13}C NMR (101 MHz, CDCl$_3$)** δ 166.3, 148.4, 143.1, 138.0, 133.3, 132.5, 132.4, 131.9, 128.1, 127.38, 127.36, 126.9, 124.3, 32.7, 27.4, 22.9, 19.2; **HRMS (ESI)** m/z calcd for $C_{18}H_{19}O_2^+$ $(M+H)^+$ 267.13796, found 267.13748.

2-Allyl-4-methoxydibenzo[b,e]oxepin-6(11H)-one (35)

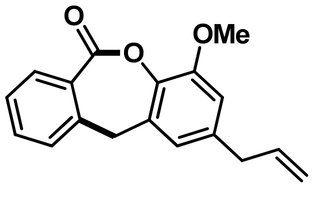

Yield 64% (36.0 mg) from 2-bromobenzaldehyde for 4 h; Yield 37% (20.7 mg) from 2-formylphenyl trifluoromethanesulfonate for 6 h, white solid; R_f = 0.30

(petroleum ether : ethyl acetate = 10 : 1); **^{1}H NMR (400 MHz, $CDCl_3$)** δ 7.90 (d, *J* = 7.6 Hz, 1H), 7.44 (t, *J* = 7.6 Hz, 1H), 7.31 (t, *J* = 7.6 Hz, 1H), 7.24 (d, *J* = 7.6 Hz, 1H), 6.66 (s, 1H), 6.64 (s, 1H), 5.96-5.86 (m, 1H), 5.12-5.07 (m, 2H), 3.95 (s, 2H), 3.85 (s, 3H), 3.31 (d, *J* = 6.4 Hz, 2H); **^{13}C NMR (101 MHz, $CDCl_3$)** δ 165.9, 150.5, 142.8, 138.3, 137.8, 136.7, 134.1, 133.2, 132.8, 128.0, 127.4, 127.2, 119.2, 116.3, 111.2, 55.9, 39.9, 37.4; **HRMS (ESI)** m/z calcd for $C_{18}H_{17}O_3^+$ $(M+H)^+$ 281.11722, found 281.11765.

(3*aR*,3*bS*,14*bR*,16*aR*)-3b,16a-dimethyl-3,3a,3b,4,5,13,14b,15,16,16a-decahydro-1H-benzo[*e*]cyclopenta[7,8]phenanthro[2,3-*b*]oxepine-1,8(2*H*)-dione (36)

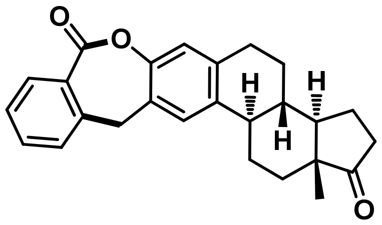

Yield 64% (49.4 mg) from 2-bromobenzaldehyde for 16 h; Yield 77% (59.5 mg) from 2-formylphenyl trifluoromethanesulfonate for 11 h, white solid; R_f = 0.25 (petroleum ether : ethyl acetate = 10 : 1); **^{1}H NMR (400 MHz, $CDCl_3$)** δ 7.86 (d, *J* = 7.2 Hz, 1H), 7.45 (dt, J_1 = 7.6 Hz, J_2 = 0.9 Hz, 1H), 7.31 (t, *J* = 7.6 Hz, 1H), 7.25 (d, *J* = 7.6 Hz, 1H), 7.17 (s, 1H), 6.96 (s, 1H), 3.95 (s, 2H), 2.86-2.84 (m, 2H), 2.54-2.41 (m, 2H), 2.25-1.95 (m, 5H), 1.67-1.36 (m, 6H), 0.89 (s, 3H); **^{13}C NMR (101 MHz, $CDCl_3$)** δ 220.7, 166.4, 148.4, 142.7, 137.3, 136.6, 133.3, 132.6, 129.7, 128.1, 127.3, 127.0, 125.0, 120.6, 50.2, 47.8, 43.9, 37.9, 37.4, 35.7, 31.4, 28.9, 26.2, 25.8, 21.5, 13.7; **HRMS (ESI)** m/z calcd for $C_{26}H_{27}O_3^+$ $(M+H)^+$ 387.19547, found 387.19577.

(*S*)-Methyl 2-(1,3-dioxoisoindolin-2-yl)-3-(6-oxo-6,11-dihydrodibenzo[*b*,*e*]oxepin-2-yl) propanoate (37)

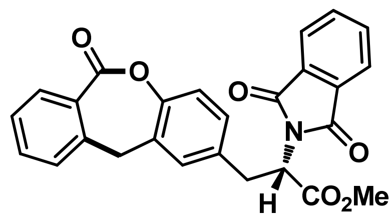

Yield 77% (68.8 mg) from 2-bromobenzaldehyde for 5 h; Yield 81% (71.8 mg) from 2-formylphenyl trifluoromethanesulfonate for 4 h, white solid; R_f = 0.30 (petroleum ether : ethyl acetate = 3 : 1); **^{1}H NMR (400 MHz, $CDCl_3$)** δ 7.81 (d, J = 7.2 Hz, 1H), 7.74-7.68 (m, 4H), 7.39 (t, J = 7.2 Hz, 1H), 7.28 (t, J = 7.2 Hz, 1H), 7.11-7.01 (m, 4H), 5.12 (dd, J_1 = 10.8 Hz, J_2 = 5.6 Hz, 1H), 3.95-3.77 (m, 5H), 3.57-3.45 (m, 2H); **^{13}C NMR (101 MHz, $CDCl_3$)** δ 169.0, 167.3, 165.9, 149.4, 142.3, 134.4, 134.1, 133.3, 132.7, 132.6, 131.3, 128.5, 128.4, 127.8, 127.2, 126.9, 123.4, 120.7, 52.9, 52.8, 37.2, 33.8; **HRMS (ESI)** m/z calcd for $C_{26}H_{20}NO_6^+$ $(M+H)^+$ 442.12851, found 442.12897.

2,8-Dimethyldibenzo[*b*,*e*]oxepin-6(11*H*)-one (38)

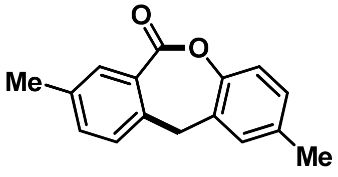

Yield 74% (35.3 mg) for 10 h, white solid; R_f = 0.25 (petroleum ether : ethyl acetate = 50 : 1); **^{1}H NMR (400 MHz, $CDCl_3$)** δ 7.68 (s, 1H), 7.26-7.24 (m, 1H), 7.14-6.97 (m, 4H), 3.90 (s, 2H), 2.31 (s, 3H), 2.28 (s, 3H); **^{13}C NMR (101 MHz, $CDCl_3$)** δ 166.7, 148.5, 139.8, 137.2, 135.5, 134.1, 133.0, 132.6, 128.5, 128.4, 127.8, 127.0, 120.3, 36.9, 20.7, 20.6; **HRMS (ESI)** m/z calcd for $C_{16}H_{15}O_2^+$ $(M+H)^+$ 239.10666, found 239.10661.

2,8-Dimethoxydibenzo[*b*,*e*]oxepin-6(11*H*)-one (39)

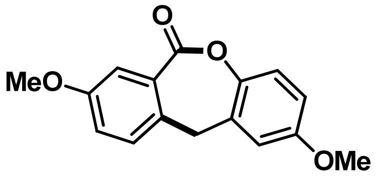

Yield 74% (40 mg) for 12 h, white solid; R_f = 0.3 (petroleum ether : ethyl acetate = 10 : 1); **^{1}H NMR (400 MHz, CDCl$_3$)** δ 7.39 (d, *J* = 2.8 Hz, 1H), 7.14 (t, *J* = 8.4 Hz, 2H), 6.99 (dd, J_1 = 8.4 Hz, J_2 = 2.4 Hz, 1H), 6.75 (d, *J* = 2.8 Hz, 1H), 6.69 (dd, J_1 = 8.8 Hz, J_2 = 2.8 Hz, 1H), 3.88 (s, 2H), 3.78 (s, 3H), 3.76 (s, 3H); **^{13}C NMR (101 MHz, CDCl$_3$)** δ 166.4, 158.7, 157.0, 144.3, 134.9, 134.3, 128.7, 128.5, 121.4, 120.2, 116.3, 113.2, 112.2, 55.6, 55.5, 36.7; **HRMS (ESI)** m/z calcd for $C_{16}H_{15}O_4^+$ $(M+H)^+$ 271.09649, found 271.09668.

1,7-Dimethoxydibenzo[*b*,*e*]oxepin-6(11*H*)-one (40)

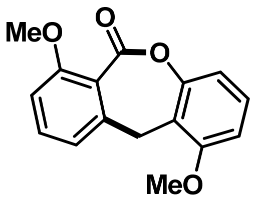

Yield 37% (20.0 mg) for 26 h, white solid; R_f = 0.25 (petroleum ether : ethyl acetate = 10 : 1); **^{1}H NMR (400 MHz, CDCl$_3$)** δ 7.32 (t, *J* = 8.0 Hz, 1H), 7.11 (t, *J* = 8.0 Hz, 1H), 6.87-6.80 (m, 3H), 6.66 (d, *J* = 8.4 Hz, 1H), 3.95 (s, 2H), 3.84 (s, 6H); **^{13}C NMR (101 MHz, CDCl$_3$)** δ 162.9, 159.5, 156.0, 151.5, 144.8, 133.0, 127.4, 121.8, 118.9, 117.0, 112.7, 110.3, 107.5, 56.1, 56.0, 28.1; **HRMS (ESI)** m/z calcd for $C_{16}H_{15}O_4^+$ $(M+H)^+$ 271.09649, found 271.09671.

2,8-Dibromodibenzo[*b*,*e*]oxepin-6(11*H*)-one (41)

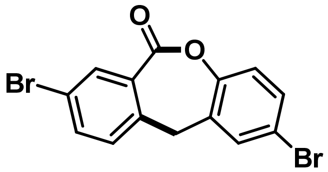

Yield 42 % (31 mg) for 5 h, white solid; R_f = 0.50 (petroleum ether : ethyl acetate = 20 : 1); **^{1}H NMR (400 MHz, CDCl$_3$)** δ 8.00 (s, 1H), 7.59 (d, *J* = 8.0 Hz, 1H),

7.39 (s, 1H), 7.34 (d, J = 8.8 Hz, 1H), 7.15 (d, J = 8.0 Hz, 1H), 7.10 (d, J = 8.8 Hz, 1H), 3.92 (s, 2H); **^{13}C NMR (101 MHz, $CDCl_3$)** δ 164.0, 149.5, 140.6, 136.4, 135.4, 134.1, 131.3, 131.0, 129.4, 128.9, 122.5, 121.4, 118.8, 36.5; **HRMS (ESI)** m/z calcd for $C_{14}H_8Br_2NaO_2^+$ $[M+Na]^+$ 388.8783, found 388.8773.

2,8-Difluorodibenzo[*b*,*e*]oxepin-6(11*H*)-one (42)

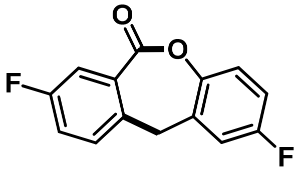

Yield 63% (31 mg) for 10 h, white solid; R_f = 0.50 (petroleum ether : ethyl acetate = 10 : 1); **^{1}H NMR (400 MHz, $CDCl_3$)** δ 7.59 (dd, J_1 = 8.4 Hz, J_2 = 2.4 Hz, 1H), 7.27-7.16 (m, 3H), 7.00-6.89 (m, 2H), 3.96 (s, 2H); **^{13}C NMR (101 MHz, $CDCl_3$)** δ 164.5 (d, J = 2.2 Hz), 161.7 (d, J = 247.8 Hz), 159.8 (d, J = 245.9 Hz), 146.4 (d, J = 2.8 Hz), 137.9 (d, J = 3.3 Hz), 134.4 (d, J = 8.3 Hz), 129.4 (d, J = 7.4 Hz), 129.1 (d, J = 7.6 Hz), 122.2 (d, J = 8.8 Hz), 120.5 (d, J = 21.5 Hz), 119.4 (d, J = 23.6 Hz), 114.8 (d, J = 14.8 Hz), 114.6 (d, J = 14.0 Hz), 36.4; **^{19}F NMR (376 MHz, $CDCl_3$)** δ -113.8, -116.2; **HRMS (ESI)** m/z calcd for $C_{14}H_9F_2O_2^+$ $(M+H)^+$ 247.05651, found 247.05618.

4,8-Dimethyldibenzo[*b*,*e*]oxepin-6(11*H*)-one (43)

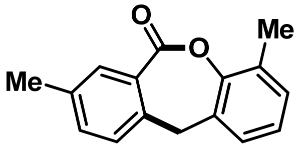

Yield 60% (29.0 mg) for 29 h, white solid; R_f = 0.25 (petroleum ether : ethyl acetate = 50 : 1); **^{1}H NMR (400 MHz, $CDCl_3$)** δ 7.71 (s, 1H), 7.26-7.24 (m, 1H), 7.14 (d, J = 8.0 Hz, 1H), 7.07-6.97 (m, 3H), 3.93 (s, 2H), 2.37 (s, 3H), 2.32 (s, 3H); **^{13}C NMR (101 MHz, $CDCl_3$)** δ 166.3, 149.1, 140.0, 137.2, 134.1, 133.1, 132.9, 130.0, 129.6, 127.6, 127.1, 125.53,

125.45, 37.0, 20.7, 16.4; **HRMS (ESI)** m/z calcd for $C_{16}H_{15}O_2^+$ $(M+H)^+$ 239.10666, found 239.10674.

2-Methoxy-8-methyldibenzo[*b*,*e*]oxepin-6(11*H*)-one (44)

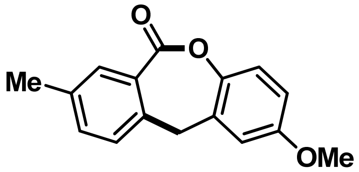

Yield 71% (36.2 mg) for 7 h, white solid; R_f = 0.20 (petroleum ether : ethyl acetate = 50 : 1); **^{1}H NMR (400 MHz, $CDCl_3$)** δ 7.69 (s, 1H), 7.26-7.24 (m, 1H), 7.15-7.12 (m, 2H), 6.76 (d, J = 2.8 Hz, 1H), 6.69 (dd, J_1 = 8.8 Hz, J_2 = 2.8 Hz, 1H), 3.90 (s, 2H), 3.76 (s, 3H), 2.32 (s, 3H); **^{13}C NMR (101 MHz, $CDCl_3$)** δ 166.8, 157.0, 144.4, 139.5, 137.3, 134.1, 134.0, 133.0, 127.7, 127.1, 121.4, 113.3, 112.3, 55.5, 37.2, 20.7; **HRMS (ESI)** m/z calcd for $C_{16}H_{15}O_3^+$ $(M+H)^+$ 255.10157, found 255.10164.

1-Methoxy-8-methyldibenzo[*b*,*e*]oxepin-6(11*H*)-one (45)

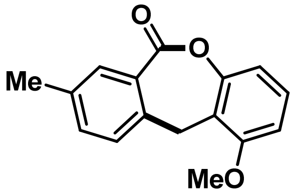

Yield 92% (47.2 mg) for 4 h, white solid; R_f = 0.25 (petroleum ether : ethyl acetate = 10 : 1); **^{1}H NMR (400 MHz, $CDCl_3$)** δ 7.71 (s, 1H), 7.24 (dd, J_1 = 7.6 Hz, J_2 = 1.2 Hz, 1H), 7.13 (d, J = 7.6 Hz, 1H), 7.04 (t, J = 8.0 Hz, 1H), 6.82-6.79 (m, 2H), 3.93 (s, 2H), 3.85 (s, 3H), 2.31 (s, 3H); **^{13}C NMR (101 MHz, $CDCl_3$)** δ 166.0, 150.7, 139.9, 139.5, 137.2, 134.7, 134.0, 133.0, 127.6, 127.1, 126.0, 119.2, 110.9, 55.9, 36.9, 20.7; **HRMS (ESI)** m/z calcd for $C_{16}H_{15}O_3^+$ $(M+H)^+$ 255.10157, found 255.10127.

2,7-Dimethoxydibenzo[*b*,*e*]oxepin-6(11*H*)-one (46)

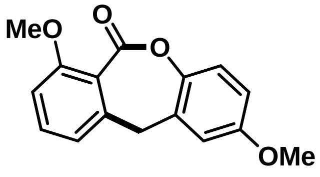

Yield 62% (33.9 mg) for 8 h, white solid; R_f = 0.25 (petroleum ether : ethyl acetate = 5 : 1); **^{1}H NMR (400 MHz, $CDCl_3$)** δ 7.33 (t, *J* = 8.0 Hz, 1H), 7.10 (d, *J* = 8.4 Hz, 1H), 6.86-6.81 (m, 2H), 6.74-6.68 (m, 2H), 3.85 (s, 5H), 3.76 (s, 3H); **^{13}C NMR (101 MHz, $CDCl_3$)** δ 163.3, 159.6, 156.7, 144.24, 144.22, 134.0, 133.1, 121.0, 118.6, 116.8, 113.3, 112.3, 110.5, 56.1, 55.6, 37.4; **HRMS (ESI)** m/z calcd for $C_{16}H_{15}O_4^+$ $(M+H)^+$ 271.09649, found 271.09625.

1,8-Dimethoxydibenzo[*b*,*e*]oxepin-6(11*H*)-one (47)

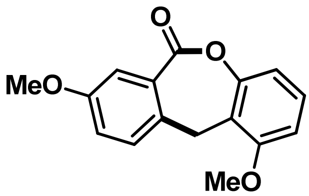

Yield 70% (38 mg) for 7 h, white solid; R_f = 0.30 (petroleum ether : ethyl acetate = 10 : 1); **^{1}H NMR (400 MHz, $CDCl_3$)** δ 7.40 (d, *J* = 2.4 Hz, 1H), 7.14 (d, *J* = 8.4 Hz, 1H), 7.04 (t, *J* = 8.0 Hz, 1H), 6.97 (dd, J_1 = 8.4 Hz, J_2 = 2.4 Hz, 1H), 6.80 (d, *J* = 7.6 Hz, 2H), 3.91 (s, 2H), 3.85 (s, 3H), 3.77 (s, 3H); **^{13}C NMR (101 MHz, $CDCl_3$)** δ 165.7, 158.6, 150.7, 139.4, 135.3, 135.0, 128.54, 128.46, 126.1, 120.2, 119.1, 116.2, 110.9, 55.9, 55.4, 36.3; **HRMS (ESI)** m/z calcd for $C_{16}H_{15}O_4^+$ $(M+H)^+$ 271.09649, found 271.09683.

7-Methoxy-2-methyldibenzo[*b*,*e*]oxepin-6(11*H*)-one (48)

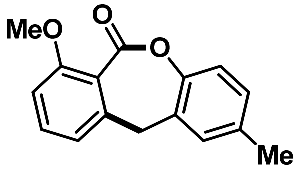

Yield 65% (33.0 mg) for 10 h, white solid; R_f = 0.30 (petroleum ether : ethyl acetate = 5 : 1); **^{1}H NMR (400 MHz, $CDCl_3$)** δ 7.32 (t, *J* = 8.0 Hz, 1H), 7.07- 6.97 (m, 3H), 6.82 (t,

J = 8.4 Hz, 2H), 3.84 (s, 5H), 2.27 (s, 3H); **^{13}C NMR (101 MHz, CDCl$_3$)** δ 163.2, 159.5, 148.3, 144.5, 135.1, 133.1, 132.5, 128.5, 128.4, 119.9, 118.5, 116.8, 110.4, 56.1, 37.1, 20.6; **HRMS (ESI)** m/z calcd for $C_{16}H_{15}O_3^+$ $(M+H)^+$ 255.10157, found 255.10165.

3-Chloro-8-methoxydibenzo[*b*,*e*]oxepin-6(11*H*)-one (49)

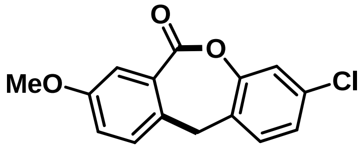

Yield 60% (33 mg) for 12 h, white solid; R_f = 0.40 (petroleum ether : ethyl acetate = 10 : 1); **^{1}H NMR (400 MHz, CDCl$_3$)** δ 7.38 (d, *J* = 2.4 Hz, 1H), 7.23 (s, 1H), 7.16 (t, *J* = 7.6 Hz, 2H), 7.09 (d, *J* = 8.0 Hz, 1H), 7.01 (dd, J_1 = 8.4 Hz, J_2 = 2.4 Hz, 1H), 3.90 (s, 2H), 3.80 (s, 3H); **^{13}C NMR (101 MHz, CDCl$_3$)** δ 165.3, 158.8, 150.9, 134.5, 133.0, 131.8, 128.7, 128.5, 128.4, 125.9, 121.2, 120.5, 116.4, 55.6, 36.0; **HRMS (ESI)** m/z calcd for $C_{15}H_{12}ClO_3^+$ $(M+H)^+$ 275.04695, found 275.04706.

8-(Benzyloxy)dibenzo[*b*,*e*]oxepin-6(11*H*)-one (50)

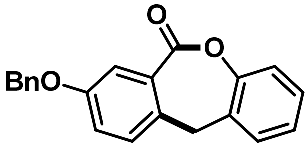

Yield 73% (46.7 mg) for 6 h, light yellow solid; R_f = 0.20 (petroleum ether : ethyl acetate = 50 : 1); **^{1}H NMR (400 MHz, CDCl$_3$)** δ 7.87 (d, *J* = 8.4 Hz, 1H), 7.40-7.33 (m, 5H), 7.25-7.19 (m, 3H), 7.14-7.09 (m, 1H), 6.89-6.84 (m, 2H), 5.09 (s, 2H), 3.95 (s, 2H); **^{13}C NMR (101 MHz, CDCl$_3$)** δ 165.8, 162.6, 150.9, 144.7, 135.8, 135.3, 132.3, 128.7, 128.3, 128.13, 128.11, 127.5, 125.8, 120.7, 120.3, 113.5, 113.2, 70.1, 37.8; **HRMS (ESI)** m/z calcd for $C_{21}H_{17}O_3^+$ $(M+H)^+$ 317.11722, found 317.11731.

8-(Benzyloxy)-2-methoxydibenzo[*b,e*]oxepin-6(11*H*)-one (51)

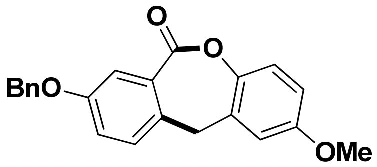

Yield 88% (61 mg) for 5 h, white solid; R_f = 0.30 (petroleum ether : ethyl acetate = 10 : 1); **^{1}H NMR (400 MHz, CDCl$_3$)** δ 7.86 (d, *J* = 8.6 Hz, 1H), 7.39-7.33 (m, 5H), 7.13 (d, *J* = 8.8 Hz, 1H), 6.88 (dd, J_1 = 8.7 Hz, J_2 = 2.4 Hz, 1H), 6.83-6.75 (m, 1H), 6.77 (d, *J* = 2.8 Hz, 1H), 6.70 (dd, J_1 = 8.9 Hz, J_2 = 2.9 Hz, 1H), 5.08 (s, 2H), 3.90 (s, 2H), 3.76 (s, 3H); **^{13}C NMR (101 MHz, CDCl$_3$)** δ 166.1, 162.4, 157.0, 144.6, 144.4, 135.8, 135.3, 133.4, 128.6, 128.2, 127.4, 121.4, 120.3, 113.5, 113.3, 113.2, 112.4, 70.1, 55.5, 37.9; **HRMS (ESI)** m/z calcd for $C_{22}H_{19}O_4^+$ $(M+H)^+$ 347.12779, found 347.12796.

8-(Benzyloxy)-2-methyldibenzo[*b,e*]oxepin-6(11*H*)-one (52)

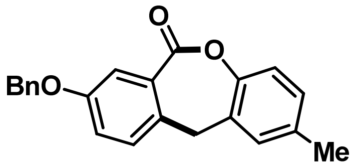

Yield 67 % (44 mg) for 9 h, white solid; R_f = 0.50 (petroleum ether : ethyl acetate = 10 : 1); **^{1}H NMR (400 MHz, CDCl$_3$)** δ 7.87 (d, *J* = 8.6 Hz, 1H), 7.40-7.33 (m, 5H), 7.10 (d, *J* = 8.2 Hz, 1H), 7.05 (s, 1H), 7.00 (d, *J* = 8.2 Hz, 1H), 6.88 (dd, J_1 = 8.8 Hz, J_2 = 2.4 Hz, 1H), 6.83 (d, *J* = 2.0 Hz, 1H), 5.09 (s, 2H), 3.91 (s, 2H), 2.29 (s, 3H); **^{13}C NMR (101 MHz, CDCl$_3$)** δ 166.1, 162.5, 148.7, 144.8, 135.8, 135.5, 135.3, 132.0, 128.67, 128.63, 128.5, 128.3, 127.5, 120.4, 113.5, 113.1, 70.1, 37.8, 20.7; **HRMS (ESI)** m/z calcd for $C_{22}H_{19}O_3^+$ $(M+H)^+$ 331.13287, found 331.13297.

8-(Benzyloxy)-2-chlorodibenzo[*b*,*e*]oxepin-6(11*H*)-one (53)

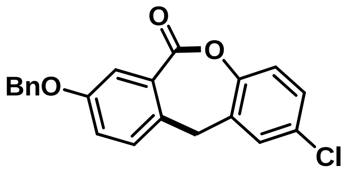

Yield 90 % (63 mg) for 8 h, white solid; R_f = 0.40 (petroleum ether : ethyl acetate = 10 : 1); **^{1}H NMR (400 MHz, CDCl$_3$)** δ 7.88 (d, J = 8.8 Hz, 1H), 7.41-7.34 (m, 5H), 7.23 (s, 1H), 7.18-7.13 (m, 2H), 6.90 (dd, J_1 = 8.8 Hz, J_2 = 2.4 Hz, 1H), 6.83 (d, J = 2.0 Hz, 1H), 5.10 (s, 2H), 3.92 (s, 2H); **^{13}C NMR (101 MHz, CDCl$_3$)** δ 165.1, 162.7, 149.4, 143.9, 135.7, 135.4, 134.0, 130.7, 128.7, 128.3, 127.9, 127.4, 122.0, 119.9, 113.6, 113.5, 70.1, 37.4; **HRMS (ESI)** m/z calcd for $C_{21}H_{16}ClO_3^+$ $(M+H)^+$ 351.07825, found 351.07843.

8-(Benzyloxy)-2-bromodibenzo[*b*,*e*]oxepin-6(11*H*)-one (54)

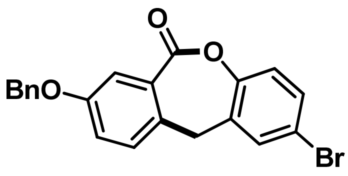

Yield 65 % (51 mg) for 5 h, white solid; R_f = 0.50 (petroleum ether : ethyl acetate = 10 : 1); **^{1}H NMR (400 MHz, CDCl$_3$)** δ 7.87 (d, J = 8.8 Hz, 1H), 7.41-7.30 (m, 7H), 7.09 (d, J = 8.8 Hz, 1H), 6.90 (dd, J_1 = 8.4 Hz, J_2 = 2.0 Hz, 1H), 6.83 (d, J = 2.0 Hz, 1H), 5.10 (s, 2H), 3.92 (s, 2H); **^{13}C NMR (101 MHz, CDCl$_3$)** δ 165.1, 162.7, 150.0, 143.9, 135.7, 135.5, 134.4, 131.0, 130.9, 128.7, 128.3, 127.5, 122.5, 119.9, 118.4, 113.6, 113.5, 70.2, 37.4; **HRMS (ESI)** m/z calcd for $C_{21}H_{16}BrO_3^+$ $(M+H)^+$ 395.02773, found 395.02777.

2-Bromo-8-fluorodibenzo[*b*,*e*]oxepin-6(11*H*)-one (55)

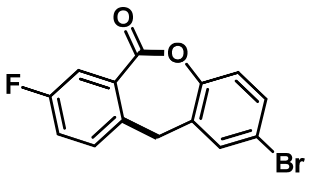

Yield 65% (40 mg) for 6 h, white solid; R_f = 0.30 (petroleum ether : ethyl acetate = 10 : 1); **^{1}H NMR (400 MHz, $CDCl_3$)** δ 7.58 (d, *J* = 7.6 Hz, 1H), 7.40-7.09 (m, 5H), 3.94 (s, 2H); **^{13}C NMR (101 MHz, $CDCl_3$)** δ 164.1 (d, *J* = 2.2 Hz), 161.7 (d, *J* = 249.0 Hz), 149.5, 137.7 (d, *J* = 3.2 Hz), 134.5, 131.2, 130.9, 129.3 (d, *J* = 7.6 Hz), 129.1 (d, *J* = 7.6 Hz), 122.5, 120.6 (d, *J* = 21.4 Hz), 119.4 (d, *J* = 23.8 Hz), 118.7, 36.2; **HRMS (ESI)** m/z calcd for $C_{14}H_9BrFO_2^+$ $(M+H)^+$ 306.97645, found 306.97629.

8-((Trimethylsilyl)ethynyl)dibenzo[b,e]oxepin-6(11H)-one (56)

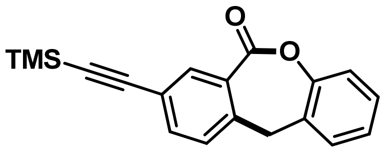

Yield 70% (43.0 mg) for 12 h, white solid; R_f = 0.50 (petroleum ether : ethyl acetate = 50 : 1); **^{1}H NMR (600 MHz, $CDCl_3$, TMS)** δ 7.98 (d, *J* = 1.8 Hz, 1H), 7.51 (dd, J_1 = 7.8 Hz, J_2 =1.8 Hz, 1H), 7.24-7.19 (m, 4H), 7.13-7.10 (m, 1H), 3.98 (s, 2H), 0.22 (s, 9H); **^{13}C NMR (151 MHz, $CDCl_3$)** δ 165.3, 150.5, 142.4, 136.2, 132.1, 128.3, 128.24, 128.19, 127.2, 126.0, 122.9, 120.7, 103.0, 96.0, 37.3, -0.2. **HRMS (ESI)** m/z calcd for $C_{19}H_{19}O_2Si^+$ $(M+H)^+$ 307.11488, found 307.11453.

3-((Trimethylsilyl)ethynyl)-7,12-dihydro-5*H*-dibenzo[*c*,*f*]oxocin-5-one (57)

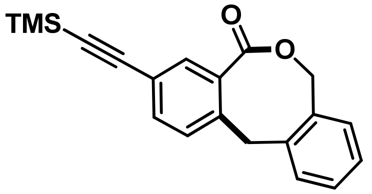

Yield 51% (33 mg), white solid; R_f = 0.50 (petroleum ether : ethyl acetate = 10 : 1); **^{1}H NMR (400 MHz, $CDCl_3$)** δ 7.71 (s, 1H), 7.56 (d, *J* = 7.6 Hz, 1H), 7.37-7.22 (m, 4H), 7.08 (d, *J* = 7.2 Hz, 1H), 5.15 (s, 2H), 4.13 (s, 2H), 0.27 (s, 9H);

^{13}C NMR (101 MHz, $CDCl_3$) δ 171.6, 136.4, 135.2, 134.4, 133.6, 132.5, 131.2, 130.22, 130.17, 129.3, 128.5, 127.2, 122.8, 103.2, 96.1, 71.5, 40.1, -0.2; **HRMS (ESI)** m/z calcd for $C_{20}H_{21}O_2Si^+$ $(M+H)^+$ 321.13053, found 321.13077.

7,12-Dihydro-5*H*-dibenzo[*c,f*]oxocin-5-one (58)

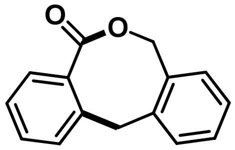

Yield 76% (34 mg), white solid; R_f = 0.40 (petroleum ether : ethyl acetate = 10 : 1); **^{1}H NMR (400 MHz, $CDCl_3$)** δ 7.47 (d, *J* = 7.6 Hz, 1H), 7.34 (t, *J* = 7.6 Hz, 1H), 7.24 (t, *J* = 7.6 Hz, 1H), 7.20-7.15 (m, 3H), 7.10-7.07 (m, 1H), 6.93 (d, *J* = 7.6 Hz, 1H), 5.01 (s, 2H), 4.00 (s, 2H); **^{13}C NMR (151 MHz, $CDCl_3$)** δ 172.2, 136.5, 133.9, 133.4, 132.0, 131.8, 129.8, 129.8, 128.8, 128.1, 127.3, 127.2, 126.7, 71.1, 39.7; **HRMS (ESI)** m/z calcd for $C_{15}H_{13}O_2^+$ $[M + H]^+$ 225.09101 , found 225.09116.

3-Fluoro-7,12-dihydro-5*H*-dibenzo[*c,f*]oxocin-5-one (59)

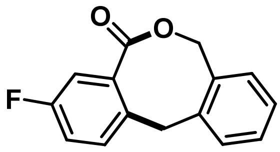

Yield 70% (34 mg), white solid; R_f = 0.40 (petroleum ether : ethyl acetate = 10 : 1); **^{1}H NMR (400 MHz, $CDCl_3$)** δ 7.35-7.13 (m, 6H), 7.07 (d, *J* = 7.2 Hz, 1H), 5.16 (s, 2H), 4.10 (s, 2H); **^{13}C NMR (101 MHz, $CDCl_3$)** δ 171.1 (d, , *J* = 2.6 Hz), 161.6 (d, *J* = 248.3 Hz), 136.6, 133.9 (d, *J* = 7.5 Hz), 133.5, 132.1 (d, *J* = 7.8 Hz), 130.3 (d, *J* = 3.6 Hz), 130.2, 129.3, 129.5, 127.2, 119.2 (d, *J* = 21.3 Hz), 114.7 (d, *J* = 23.6 Hz), 71.6, 39.4; **^{19}F NMR (376 MHz, $CDCl_3$)** δ -113.6; **HRMS (ESI)** m/z calcd for $C_{15}H_{12}FO_2^+$ $(M+H)^+$ 243.08158, found 243.08176.

3-Bromo-7,12-dihydro-5*H*-dibenzo[*c*,*f*]oxocin-5-one (60)

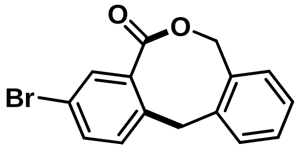

Yield 51% (31 mg), white solid; R_f = 0.40 (petroleum ether : ethyl acetate = 10 : 1); **^{1}H NMR (600 MHz, $CDCl_3$)** δ 7.72 (d, *J* = 1.8 Hz, 1H), 7.58 (dd, J_1 = 8.4 Hz, J_2 = 2.4 Hz, 1H), 7.32 (td, J_1 = 7.8 Hz, J_2 = 1.2 Hz, 1H), 7.28 (d, *J* = 7.2 Hz, 1H), 7.22 (td, J_1 = 7.2 Hz, J_2 = 1.2 Hz, 1H), 7.19 (d, *J* = 7.8 Hz, 1H), 7.08 (d, *J* = 7.2 Hz, 1H), 5.16 (s, 2H), 4.09 (s, 2H); **^{13}C NMR (151 MHz, $CDCl_3$)** δ 170.9, 136.1, 135.1, 134.1, 133.5, 133.4, 131.8, 130.5, 130.2, 129.3, 128.5, 127.2, 121.2, 71.6, 39.7; **HRMS (ESI)** m/z calcd for $C_{15}H_{11}BrNaO_2^+$ (M + Na)$^+$ 324.9835, found 324.9831.

3-Methyl-7,12-dihydro-5*H*-dibenzo[*c*,*f*]oxocin-5-one (61)

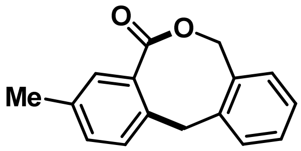

Yield 84% (40 mg), white solid; R_f = 0.40 (petroleum ether : ethyl acetate = 10 : 1); **^{1}H NMR (400 MHz, $CDCl_3$)** δ 7.38 (s, 1H), 7.32-7.25 (m, 3H), 7.20-7.16 (m, 2H), 7.03 (d, *J* = 6.8 Hz, 1H), 5.13 (s, 2H), 4.08 (s, 2H), 2.36 (s, 3H); **^{13}C NMR (101 MHz, $CDCl_3$)** δ 172.8, 137.4, 137.1, 133.8, 132.9, 132.1, 131.3, 130.04, 130.02, 129.1, 128.4, 128.1, 126.9, 71.5, 39.7, 20.8; **HRMS (ESI)** m/z calcd for $C_{16}H_{15}O_2^+$ (M+H)$^+$ 239.10666, found 239.10683.

9-Methoxy-7,12-dihydro-5*H*-dibenzo[*c*,*f*]oxocin-5-one (62)

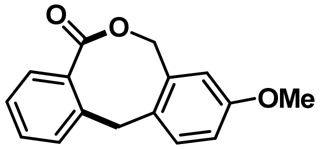

Yield 64% (32 mg), white solid; R_f = 0.30 (petroleum ether : ethyl acetate = 10 : 1); **^{1}H NMR (400 MHz, $CDCl_3$)** δ 7.57 (d, *J* = 7.6 Hz, 1H), 7.46 (t, d, *J* = 7.6 Hz, 1H), 7.36 (t, d, *J* = 7.6 Hz, 1H), 7.28 (d, *J* = 7.6 Hz, 1H), 7.22 (d, *J* = 8.4 Hz, 1H), 6.83 (dd, J_1 = 8.4 Hz, J_2 = 2.4 Hz, 1H),

6.61 (d, J = 2.0 Hz, 1H), 5.09 (s, 2H), 4.07 (s, 2H), 3.77 (s, 3H); **^{13}C NMR (101 MHz, CDCl$_3$)** δ 172.7, 158.3, 134.99, 134.91, 132.2, 132.1, 131.2, 130.0, 129.0, 127.7, 127.4, 114.4, 113.6, 71.4, 55.3, 39.3; **HRMS (ESI)** m/z calcd for $C_{16}H_{15}O_3^+$ (M+H)$^+$ 255.10157, found 255.10168.

10-Methyl-7,12-dihydro-5*H*-dibenzo[*c,f*]oxocin-5-one (63)

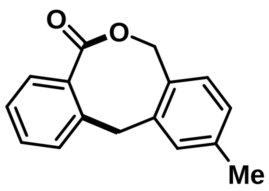

Yield 73% (35 mg), white solid; R_f = 0.40 (petroleum ether : ethyl acetate = 10 : 1); **^{1}H NMR (600 MHz, CDCl$_3$)** δ 7.57 (dd, J_1 = 7.8 Hz, J_2 = 1.2 Hz, 1H), 7.45 (td, J_1 = 7.2 Hz, J_2 = 1.2 Hz, 1H), 7.35 (td, J_1 = 7.2 Hz, J_2 = 0.6 Hz, 1H), 7.30 (d, J = 7.2 Hz, 1H), 7.13 (s, 1H), 7.01 (d, J = 7.2 Hz, 1H), 6.94 (d, J = 7.2 Hz, 1H), 5.10 (s, 2H), 4.08 (s, 2H), 2.36 (s, 3H); **^{13}C NMR (151 MHz, CDCl$_3$)** δ 172.7, 139.0, 136.5, 134.3, 132.3, 132.0, 130.9, 130.7, 130.0, 128.4, 127.6, 127.45, 127.41, 71.4, 40.1, 20.9; **HRMS (ESI)** m/z calcd for $C_{16}H_{15}O_2^+$ (M+H)$^+$ 239.10666, found 239.10678.

Methyl 6-oxo-6,11-dihydrodibenzo[*b,e*]oxepine-8-carboxylate (64)

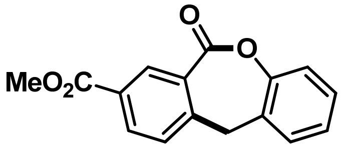

Yield 36% (19.4 mg) for 4 h, white solid; R_f = 0.25 (petroleum ether : ethyl acetate = 10 : 1); **^{1}H NMR (400 MHz, CDCl$_3$)** δ 8.55 (d, J = 1.2 Hz, 1H), 8.12 (dd, J_1 = 8.0 Hz, J_2 = 1.6 Hz, 1H), 7.37 (d, J = 8.0 Hz, 1H), 7.28-7.24 (m, 3H), 7.17-7.12 (m, 1H), 4.06 (s, 2H), 3.91 (s, 3H); **^{13}C NMR (101 MHz, CDCl$_3$)** δ 165.6, 165.2, 150.5, 147.0, 134.2, 134.1, 131.7, 129.8, 128.5, 128.4, 128.3, 127.5, 126.1, 120.8, 52.4, 37.4; **HRMS (ESI)** m/z calcd for $C_{16}H_{13}O_4^+$ (M+H)$^+$ 269.08084, found 269.08075.

N-(6-oxo-6,11-dihydrodibenzo[*b*,*e*]oxepin-8-yl)acetamide (65)

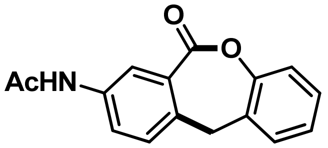

Yield 34% (18.4 mg) for 4 h, white solid; R_f = 0.25 (petroleum ether : ethyl acetate = 2 : 1); **^{1}H NMR (400 MHz, $CDCl_3$)** δ 8.00 (dd, J_1 = 8.4 Hz, J_2 = 2.0 Hz, 1H), 7.87 (s, 1H), 7.75 (d, J = 2.0 Hz, 1H), 7.26-7.19 (m, 4H), 7.12 (td, J_1 = 6.8 Hz, J_2 = 2.0 Hz, 1H), 3.96 (s, 2H), 2.19 (s, 3H); **^{13}C NMR (101 MHz, $CDCl_3$)** δ 168.8, 166.1, 150.5, 138.2, 137.5, 132.7, 128.2, 128.13, 128.1, 126.0, 125.2, 123.4, 120.7, 36.8, 24.4; **HRMS (ESI)** m/z calcd for $C_{16}H_{14}NO_3^+$ $(M+H)^+$ 268.09682, found 268.09698.

(2*aR*,5*aR*,5*bS*,16*bR*)-2a-Methyl-2,2a,5,5a,6,7,9,16b-octahydro-1H-benzo[*b*]cyclopenta[7,8]phenanthro[2,3-e]oxepine-3,15(4*H*,5*bH*)-dione (66)

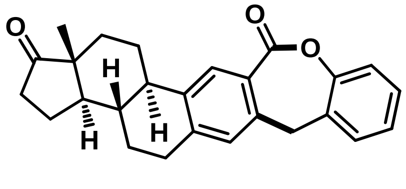

Yield 70% (54.6 mg) for 10 h, white solid; R_f = 0.30 (petroleum ether : ethyl acetate = 5 : 1); **^{1}H NMR (400 MHz, $CDCl_3$)** δ 7.82 (s, 1H), 7.25-7.18 (m, 3H), 7.14-7.09 (m, 1H), 6.99 (s, 1H), 3.94 (dd, J_1 = 34.4 Hz, J_2 = 13.6 Hz, 2H), 2.97-2.84 (m, 2H), 2.53-2.43 (m, 2H), 2.23-1.95 (m, 5H), 1.66-1.37 (m, 6H), 0.87 (s, 3H); **^{13}C NMR (101 MHz, $CDCl_3$)** δ 220.5, 166.4, 150.8, 143.0, 139.9, 139.3, 132.9, 130.1, 128.1, 128.0, 127.7, 125.8, 125.2, 120.7, 50.3, 47.8, 43.9, 37.7, 37.0, 35.7, 31.3, 29.3, 26.0, 25.5, 21.5, 13.7; **HRMS (ESI)** m/z calcd for $C_{26}H_{27}O_3^+$ $(M+H)^+$ 387.19547, found 387.19522.

8-Allyl-10-methoxydibenzo[*b*,*e*]oxepin-6(11*H*)-one (67)

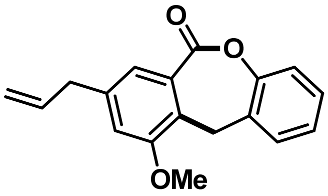

Yield 75% (42.4 mg) for 10 h, white solid; R_f = 0.20 (petroleum ether : ethyl acetate = 50 : 1); **^{1}H NMR (400 MHz, $CDCl_3$)** δ 7.28-7.19 (m, 4H), 7.12-7.07 (m, 1H), 6.85 (s, 1H), 5.94-5.84 (m, 1H), 5.11-5.07 (m, 2H), 4.05 (s, 2H), 3.87 (s, 3H), 3.35 (d, *J* = 6.4 Hz, 2H); **^{13}C NMR (101 MHz, $CDCl_3$)** δ 166.3, 154.7, 151.2, 140.1, 136.3, 133.0, 129.28, 129.26, 128.4, 127.8, 125.6, 123.8, 120.4, 116.7, 115.2, 56.1, 39.9, 27.2; **HRMS (ESI)** m/z calcd for $C_{18}H_{17}O_3^+$ $(M+H)^+$ 281.11722, found 281.11737.

7-Isopropyl-10-methyldibenzo[*b*,*e*]oxepin-6(11*H*)-one (68)

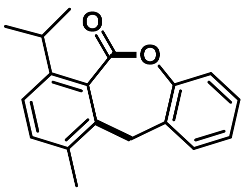

Yield 52% (27.6 mg) for 9 h, white solid; R_f = 0.45 (petroleum ether : ethyl acetate = 50 : 1); **^{1}H NMR (400 MHz, $CDCl_3$)** δ 7.24-7.13 (m, 5H), 7.06 (td, J_1 = 7.2 Hz, J_2 = 1.2 Hz, 1H), 3.90 (s, 2H), 3.40-3.29 (m, 1H), 2.42 (s, 3H), 1.20 (d, *J* = 7.2 Hz, 6H); **^{13}C NMR (101 MHz, $CDCl_3$)** δ 166.2, 150.9, 147.7, 140.3, 133.1, 132.8, 130.1, 128.0, 127.7, 127.6, 125.2, 124.5, 119.6, 32.1, 29.9, 24.0, 19.4; **HRMS (ESI)** m/z calcd for $C_{18}H_{19}O_2^+$ $(M+H)^+$ 267.13796, found 267.13785.

(S)-Methyl 2-(1,3-dioxoisoindolin-2-yl)-3-(6-oxo-6,11-dihydrodibenzo[*b*,*e*]oxepin-8-yl)propanoate (69)

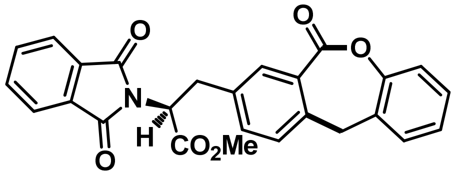

Yield 64% (57.3 mg) for 10 h, white solid; R_f = 0.25 (petroleum ether : ethyl acetate = 3 : 1); **^{1}H NMR (400 MHz, $CDCl_3$)** δ 7.77-7.74 (m, 1H),

7.71-7.68 (m, 2H), 7.31 (dd, J_1 = 7.6 Hz, J_2 = 1.6 Hz, 1H), 7.20-7.06 (m, 3H), 5.10 (dd, J_1 = 10.4 Hz, J_2 = 5.2 Hz, 1H), 3.94-3.86 (m, 1H), 3.76 (s, 2H), 3.61-3.48 (m, 1H); **^{13}C NMR (101 MHz, $CDCl_3$)** δ 169.0, 167.4, 165.6, 150.5, 141.2, 136.3, 134.2, 133.7, 133.2, 132.4, 131.4, 128.1, 128.0, 127.6, 125.7, 123.5, 120.6, 53.0, 52.7, 37.0, 34.0; **HRMS (ESI)** m/z calcd for $C_{26}H_{20}NO_6^+$ $(M+H)^+$ 442.12851, found 442.12909.

***N*-(5-oxo-7,12-dihydro-5*H*-dibenzo[*c,f*]oxocin-3-yl)acetamide (70)**

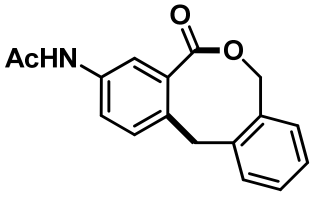

Yield 70% (39 mg), white solid; R_f = 0.40 (petroleum ether : ethyl acetate = 2 : 1); **^{1}H NMR (600 MHz, DMSO-d_6)** δ 10.16 (s, 1H), 7.84 (d, J = 2.4 Hz, 1H), 7.66 (dd, J_1 = 8.4 Hz, J_2 = 1.8 Hz, 1H), 7.38 (d, J = 7.2 Hz, 1H), 7.34-7.31 (m, 2H), 7.22 (td, J_1 = 7.8 Hz, J_2 = 1.2 Hz, 1H), 7.17 (d, J = 6.6 Hz, 1H), 5.13 (s, 2H), 4.01 (s, 2H), 2.05 (s, 3H); **^{13}C NMR (151 MHz, DMSO-d_6)** δ 172.1, 169.1, 139.1, 137.4, 134.4, 132.9, 131.1, 130.7, 129.5, 129.1, 128.9, 127.3, 122.8, 117.9, 71.3, 38.9, 24.5; **HRMS (ESI)** m/z calcd for $C_{17}H_{16}NO_3^+$ $(M+H)^+$ 282.11247, found 282.11264.

(3*aR*,3*bS*,15*bR*,17*aR*)-17a-Methyl-3,3a,4,5,7,12,15b,16,17,17a-decahydrobenzo[*c*] cyclopenta[7,8]phenanthro[2,3-f]oxocine-1,14(2*H*,3*bH*)-dione (71)

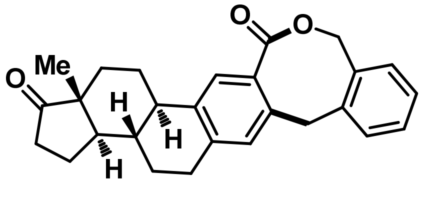

Yield 72% (58 mg), white solid; R_f = 0.30 (petroleum ether : ethyl acetate = 5 : 1); **^{1}H NMR (400 MHz, $CDCl_3$)** δ 7.50 (s, 1H), 7.31-7.27 (m, 2H), 7.19 (td, J_1 = 6.8 Hz, J_2 = 1.6 Hz, 1H), 7.06 (d, J = 7.4 Hz, 1H), 7.02 (s, 1H), 5.16 (s, 2H), 4.08 (s, 2H), 2.91-2.87 (m, 2H), 2.55-2.43 (m, 2H), 2.31-2.26 (m, 1H), 2.20-1.97 (m, 4H), 1.61-1.41 (m, 6H), 0.90 (s, 3H); **^{13}C NMR (101 MHz, $CDCl_3$)** δ

220.4, 173.0, 141.3, 139.5, 137.2, 133.9, 131.9, 130.7, 130.1, 129.8, 129.1, 128.3, 126.9, 125.0, 71.6, 50.4, 47.8, 44.0, 39.8, 37.9, 35.7, 31.4, 29.3, 26.2, 25.6, 21.5, 13.7; **HRMS (ESI)** m/z calcd for $C_{27}H_{29}O_3^+$ $(M+H)^+$ 401.21112, found 401.21170.

(2*aS*,2*bR*,5*aR*,7*aR*)-5a-Methyl-2,2a,3,4,5a,6,7,7a,11,17-decahydro-[1,3]dioxolo[4',5':4,5]benzo[1,2-*c*]cyclopenta[7,8]phenanthro[2,3-*f*]oxocine-5,9(1*H*,2*bH*)-dione (72)

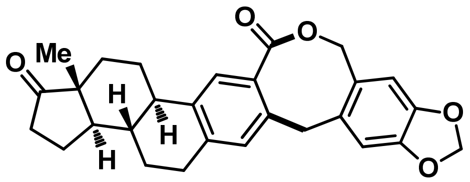

Yield 78% (69 mg), white solid; R_f = 0.30 (petroleum ether : ethyl acetate = 5 : 1); **^{1}H NMR (600 MHz, CDCl$_3$)** δ 7.49 (s, 1H), 7.00 (s, 1H), 6.77 (s, 1H), 6.55 (s, 1H), 5.95 (s, 2H), 5.06 (s, 2H), 3.98 (s, 2H), 2.92-2.88 (m, 2H), 2.55-2.43 (m, 2H), 2.31-2.26 (m, 1H), 2.20-197 (m, 4H), 1.66-1.41 (m, 6H), 0.91 (s, 3H); **^{13}C NMR (151 MHz, CDCl$_3$)** δ 220.5, 173.0, 147.8, 146.1, 141.4, 139.4, 132.2, 131.0, 130.5, 129.7, 127.2, 125.1, 110.7, 108.8, 101.3, 71.3, 50.3, 47.8, 44.0, 39.5, 37.8, 35.7, 31.4, 29.3, 26.1, 25.6, 21.5, 13.7; **HRMS (ESI)** m/z calcd for $C_{28}H_{29}O_5^+$ $(M+H)^+$ 445.20095, found 445.20068.

Methyl 2-(1,3-dioxoisoindolin-2-yl)-3-(5-oxo-7,12-dihydro-5*H*-dibenzo[*c*,*f*]oxocin-3-yl)propanoate (73)

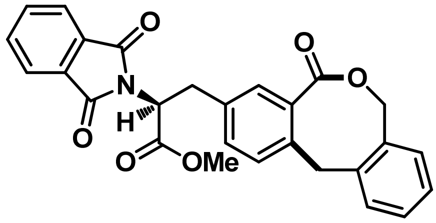

Yield 78% (71 mg), white solid; R_f = 0.30 (petroleum ether : ethyl acetate = 5 : 1); **^{1}H NMR (600 MHz, CDCl$_3$)** δ 7.70 (q, *J* = 3.0 Hz, 2H), 7.65 (q, *J* = 3.0 Hz, 2H), 7.33 (q, *J* = 1.8 Hz, 1H), 7.26-7.24 (m, 2H), 7.19 (d, *J* = 6.6 Hz, 1H), 7.14 (td, J_1 = 7.8 Hz, J_2 = 1.2 Hz, 1H), 7.09 (d, *J* = 7.8 Hz, 1H), 6.97 (d, *J* =

7.2 Hz, 1H), 5.09 (q, J = 5.4 Hz, 1H), 4.93-4.85 (m, 2H), 4.02-3.94 (m, 2H), 3.75 (s, 3H), 3.59 (dd, J_1 = 14.4 Hz, J_2 = 5.4 Hz, 1H), 3.51 (dd, J_1 = 14.4 Hz, J_2 = 10.8 Hz, 1H), **^{13}C NMR (151 MHz, $CDCl_3$)** δ 172.0, 168.8, 167.2, 136.7, 136.5, 134.2, 133.7, 132.8, 132.6, 132.5, 131.3, 130.4, 130.1, 129.1, 128.3, 128.1, 126.9, 123.4, 71.2, 52.90, 52.88, 39.6, 34.1; **HRMS (ESI)** m/z calcd for $C_{27}H_{22}NO_6^+$ $(M+H)^+$ 456.14416, found 456.14447.

Methyl 5-oxo-7,12-dihydro-5*H*-dibenzo[*c,f*]oxocine-3-carboxylate (74)

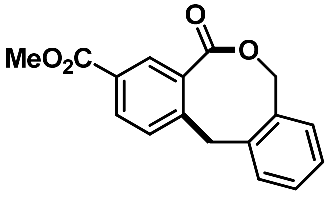

Yield 54% (30 mg), white solid; R_f = 0.30 (petroleum ether : ethyl acetate = 5 : 1); **^{1}H NMR (400 MHz, $CDCl_3$)** δ 8.26 (d, J = 1.6 Hz, 1H), 8.12 (dd, J_1 = 8.0 Hz, J_2 = 1.6 Hz, 1H), 7.40 (d, J = 8.0 Hz, 1H), 7.35-7.30 (m, 2H), 7.23 (td, J_1 = 7.2 Hz, J_2 = 2.0 Hz, 1H), 7.07 (d, J = 7.6 Hz, 1H), 5.14 (s, 2H), 4.18 (s, 2H), 3.93 (s, 3H); **^{13}C NMR (101 MHz, $CDCl_3$)** δ 171.5, 165.7, 139.2, 135.9, 133.6, 133.1, 132.6, 130.4, 130.3, 129.6, 129.3, 129.1, 128.5, 127.3, 71.5, 52.4, 40.2; **HRMS (ESI)** m/z calcd for $C_{17}H_{15}O_4^+$ $(M+H)^+$ 283.09649, found 283.09698.

(3*aR*,5*aR*,11*aS*,11*bR*,14*aR*,16*aR*,21*aS*,21*bR*)-3a,14a-Dimethyl-1,3a,4,5,5a,11,11a,12,13,14a,15,16,16a,18,20,21,21a,21b-octadecahydrocyclopenta[7,8]phenanthro[2,3-*b*]cyclopenta[7,8]phenanthro[2,3-*e*]oxepine-3,7,14(2*H*,10*H*,11b*H*)-trione (75)

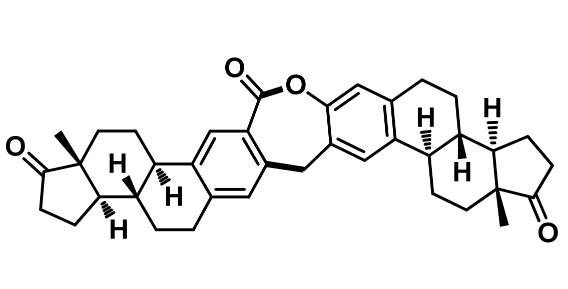

Yield 74% (83.6 mg) for 21 h, white solid; R_f = 0.25 (petroleum ether : ethyl acetate = 3 : 1); **^{1}H NMR (400 MHz,**

$CDCl_3$) δ 7.80 (s, 1H), 7.15 (s, 1H), 6.98 (s, 1H), 6.95 (s, 1H), 3.89 (dd, J_1 = 38.4 Hz, J_2 = 11.6 Hz, 2H), 2.92-2.82 (m, 4H), 2.53-2.41 (m, 4H), 2.24-1.94 (m, 10H), 1.64-1.36 (m, 12H), 0.89 (s, 3H), 0.87 (s, 3H); **^{13}C NMR (101 MHz, $CDCl_3$)** δ 220.7, 220.5, 166.7, 148.6, 142.8, 140.0, 139.1, 137.2, 136.5, 130.0, 129.9, 127.6, 125.3, 124.9, 120.6, 50.22, 50.19, 47.8, 47.7, 43.9, 43.8, 37.9, 37.7, 36.9, 35.73, 35.69, 31.4, 31.3, 29.3, 28.9, 26.2, 26.0, 25.8, 25.5, 21.4, 13.69, 13.65; **HRMS (ESI)** m/z calcd for $C_{38}H_{43}O_4^+$ $(M+H)^+$ 563.31559, found 563.31580.

(2*aR*,5*aR*,5*bS*,16*bR*)-11-Allyl-13-methoxy-2a-methyl-2,2a,5,5a,6,7,9,16b-octahydro-1H-benzo[b]cyclopenta[7,8]phenanthro[2,3-*e*]oxepine-3,15(4H,5*bH*)-dione (76)

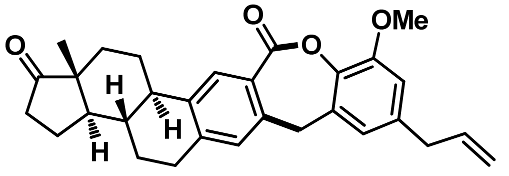

Yield 43% (38.8 mg) for 8 h, white solid; R_f = 0.50 (petroleum ether : ethyl acetate = 3 : 1); **^{1}H NMR (400 MHz, $CDCl_3$)** δ 7.83 (s, 1H), 6.97 (s, 1H), 6.64 (s, 1H), 6.62 (s, 1H), 5.96-5.86 (m, 1H), 5.12- 5.07 (m, 2H), 3.93 (s, 2H), 3.84 (s, 3H), 3.31 (d, J = 6.4 Hz, 2H), 2.97-2.84 (m, 2H), 2.53-2.42 (m, 2H), 2.23-1.94 (m, 5H), 1.66-1.39 (m, 6H), 0.87 (s, 3H); **^{13}C NMR (101 MHz, $CDCl_3$)** δ 220.5, 166.2, 150.6, 142.8, 140.1, 139.2, 138.2, 138.0, 136.7, 134.4, 130.2, 127.7, 125.2, 119.1, 116.3, 111.2, 56.0, 50.3, 47.8, 43.9, 39.9, 37.8, 37.0, 35.7, 31.3, 29.3, 26.0, 25.5, 21.5, 13.7; **HRMS (ESI)** m/z calcd for $C_{30}H_{33}O_4^+$ $(M+H)^+$ 457.23734, found 457.23764.

(*S*)-Methyl 2-(1,3-dioxoisoindolin-2-yl)-3-((2*aR*,5*aR*,5*bS*,16*bR*)-2a-methyl-3,15-dioxo-2,2a,3,4,5,5a,5b,6,7,9,15,16b-dodecahydro-1H-benzo[*b*]cyclopenta[7,8]phenanthro[2,3-*e*]oxepin-11-yl)propanoate (77)

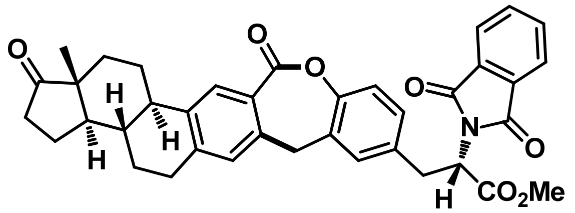

Yield 64% (79.2 mg) for 5 h, white solid; R_f = 0.25 (petroleum ether : ethyl acetate = 2 : 1); **^{1}H NMR (400 MHz, $CDCl_3$)** δ 7.80-7.70 (m, 5H), 7.08-6.97 (m, 3H), 6.91 (d, *J* = 6.8 Hz, 1H), 5.15-5.10 (m, 1H), 3.95-3.77 (m, 5H), 3.58-3.45 (m, 2H), 2.97-2.92 (m, 2H), 2.53-2.37 (m, 2H), 2.22-1.94 (m, 5H), 1.66-1.36 (m, 6H), 0.87 (d, *J* = 2.4 Hz, 3H); **^{13}C NMR (101 MHz, $CDCl_3$)** δ 220.4, 169.1, 167.4, 166.2, 149.6, 142.9, 139.6, 139.2, 134.3, 134.2, 132.92, 132.90, 131.4, 129.98, 129.97, 128.5, 128.4, 128.3, 128.2, 127.62, 127.58, 125.1, 123.5, 120.82, 120.78, 52.9, 52.82, 52.76, 50.2, 47.8, 43.8, 37.7, 36.9, 35.7, 33.8, 31.3, 29.3, 26.0, 25.4, 21.4, 13.7; **HRMS (ESI)** m/z calcd for $C_{38}H_{36}NO_7^+$ $(M+H)^+$ 618.24863, found 618.24878.

(3*aR*,5*aR*,16*aS*,16*bR*)-10-Allyl-8-methoxy-3a-methyl-3a,4,5,5a,15,16,16a,16b-octahydro-1H-benzo[*e*]cyclopenta[7,8]phenanthro[2,3-*b*]oxepine-3,12(2*H*,7*H*)-dione (78)

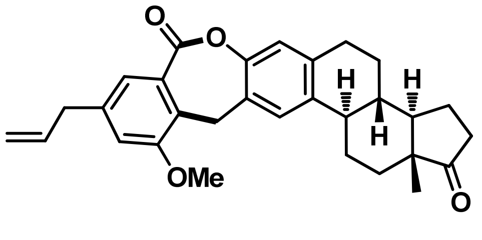

Yield 42% (39.0 mg) for 17 h, white solid; R_f = 0.15 (petroleum ether : ethyl acetate = 10 : 1); **^{1}H NMR (400 MHz, $CDCl_3$)** δ 7.27 (s, 1H), 7.16 (s, 1H), 6.93 (s, 1H), 6.84 (s, 1H), 5.93-5.83 (m, 1H), 5.11-5.06 (m, 2H),

4.04-3.95 (m, 2H), 3.87 (s, 3H), 3.34 (d, J = 6.8 Hz, 2H), 2.84 (dd, J_1 = 8.8 Hz, J_2 = 4.0 Hz, 2H), 2.54-2.43 (m, 2H), 2.22-1.96 (m, 5H), 1.67-1.48 (m, 6H), 0.90 (s, 3H); **^{13}C NMR (101 MHz, CDCl$_3$)** δ 220.8, 166.7, 154.6, 149.0, 140.0, 137.0, 136.34, 136.31, 130.1, 129.4, 125.3, 123.8, 120.3, 116.6, 115.2, 56.1, 50.3, 47.9, 44.0, 39.9, 37.9, 35.8, 31.5, 29.0, 27.1, 26.8, 26.2, 25.8, 21.5, 13.8; **HRMS (ESI)** m/z calcd for $C_{30}H_{33}O_4^+$ $(M+H)^+$ 457.23734, found 457.23749.

2,8-Diallyl-4,10-dimethoxydibenzo[*b*,*e*]oxepin-6(11*H*)-one (79)

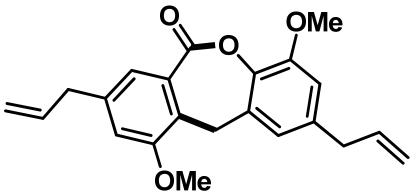

Yield 53% (37.5 mg) for 13 h, white solid; R_f = 0.25 (petroleum ether : ethyl acetate = 20 : 1); **^{1}H NMR (400 MHz, CDCl$_3$)** δ 7.30 (s, 1H), 6.84 (s, 1H), 6.67 (s, 1H), 6.61 (s, 1H), 5.96-5.83 (m, 2H), 5.12-5.06 (m, 4H), 4.00 (s, 2H), 3.87 (s, 3H), 3.83 (s, 3H), 3.32 (dd, J_1 = 11.4 Hz, J_2 = 6.8 Hz, 4H); **^{13}C NMR (101 MHz, CDCl$_3$)** δ 166.1, 154.7, 150.3, 140.0, 138.4, 138.0, 136.8, 136.3, 134.4, 129.5, 129.3, 124.0, 119.6, 116.6, 116.2, 115.2, 111.0, 56.1, 55.9, 40.0, 39.9, 27.1; **HRMS (ESI)** m/z calcd for $C_{22}H_{23}O_4^+$ $(M+H)^+$ 351.15909, found 351.15933.

(*S*)-Methyl 3-(8-allyl-10-methoxy-6-oxo-6,11-dihydrodibenzo[*b*,*e*]oxepin-2-yl)-2-(1,3-dioxoisoindolin-2-yl)propanoate (80)

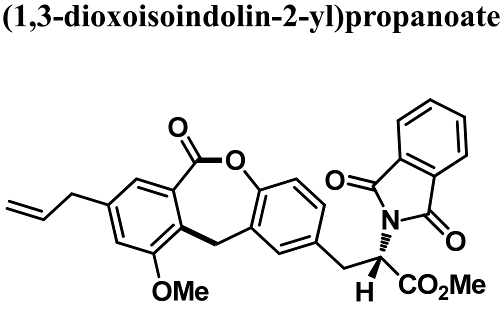

Yield 52% (53.8 mg) for 5 h, white solid; R_f = 0.18 (petroleum ether : ethyl acetate = 5 : 1); **^{1}H NMR (400 MHz, CDCl$_3$)** δ 7.77-7.67 (m, 4H), 7.22 (s, 1H), 7.09 (s, 1H), 7.01-6.97 (m, 2H), 6.80 (s, 1H), 5.93-5.83 (m, 1H),

5.15-5.06 (m, 3H), 3.91 (s, 2H), 3.84 (s, 3H), 3.77 (s, 3H), 3.57-3.45 (m, 2H), 3.33 (d, *J* = 6.8 Hz, 2H); **^{13}C NMR (101 MHz, CDCl$_3$)** δ 169.1, 167.4, 166.2, 154.6, 150.0, 140.0, 136.2, 134.2, 134.1, 133.1, 131.4, 129.1, 129.0, 128.8, 128.1, 123.7, 123.4, 120.4, 116.6, 115.2, 56.0, 52.9, 39.9, 33.8, 27.0; **HRMS (ESI)** m/z calcd for $C_{30}H_{26}NO_7^+$ $(M+H)^+$ 512.17038, found 512.17053.

(*S*)-methyl 2-(1,3-dioxoisoindolin-2-yl)-3-((3*aR*,3*bS*,14*bR*,16*aR*)-16a-methyl-1,8-dioxo-2,3,3a,3b,4,5,8,13,14b,15,16,16a-dodecahydro-1H-benzo[*e*]cyclopenta[7,8]phenanthro[2,3-*b*]oxepin-10-yl)propanoate (81)

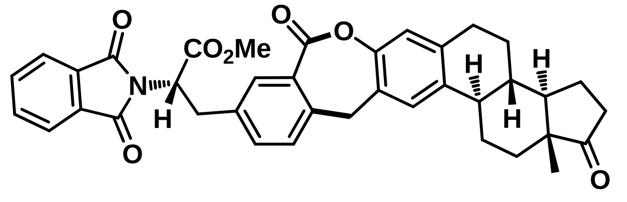

Yield 31% (38.2 mg) for 28 h, white solid; R_f = 0.20 (petroleum ether : ethyl acetate = 3 : 1); **^{1}H NMR (400 MHz, CDCl$_3$)** δ 7.79-7.68 (m, 5H), 7.31-7.27 (m, 1H), 7.12-7.10 (m, 2H), 6.90 (d, *J* = 3.6 Hz, 1H), 5.10 (dd, J_1 = 10.4 Hz, J_2 = 5.2 Hz, 1H), 3.85 (s, 2H), 3.76 (s, 3H), 3.61-3.49 (m, 2H), 2.83 (d, *J* = 4.8 Hz, 2H), 2.53-2.34 (m, 2H), 2.21-1.94 (m, 5H), 1.66-1.33 (m, 6H), 0.88 (s, 3H); **^{13}C NMR (101 MHz, CDCl$_3$)** δ 220.7, 169.0, 167.4, 165.9, 148.4, 141.3, 137.2, 136.6, 136.2, 134.2, 133.6, 133.2, 131.5, 129.5, 128.3, 127.6, 125.0, 123.6, 120.58, 120.56, 53.0, 52.8, 50.3, 47.8, 43.9, 37.9, 37.0, 35.8, 33.9, 31.4, 28.9, 26.2, 25.8, 21.5, 13.7; **HRMS (ESI)** m/z calcd for $C_{38}H_{36}NO_7^+$ $(M+H)^+$ 618.24863, found 618.24884.

(*S*)-Methyl 3-(2-allyl-4-methoxy-6-oxo-6,11-dihydrodibenzo[*b*,*e*]oxepin-8-yl)-2-(1,3-dioxoisoindolin-2-yl)propanoate (82)

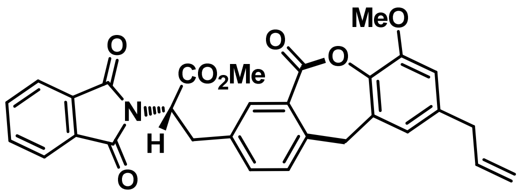

Yield 47% (48.1 mg) for 11 h, white solid; R_f = 0.24 (petroleum ether : ethyl acetate = 3 : 1); **^{1}H NMR (400 MHz, $CDCl_3$)** δ 7.79-7.69 (m, 5H), 7.30 (dd, J_1 = 8.0 Hz, J_1 = 1.6 Hz, 1H), 7.11 (d, J = 8.0 Hz, 1H), 6.60 (d, J = 1.2 Hz, 2H), 5.94-5.84 (m, 1H), 5.12-5.06 (m, 3H), 3.84 (s, 2H), 3.82 (s, 3H), 3.76 (s, 3H), 3.61-3.48 (m, 2H), 3.29 (d, J = 6.8 Hz, 2H).; **^{13}C NMR (101 MHz, $CDCl_3$)** δ 169.0, 167.4, 165.4, 150.5, 141.4, 138.2, 137.8, 136.7, 136.3, 134.2, 133.9, 133.5, 133.4, 131.5, 128.2, 127.7, 123.6, 119.2, 116.3, 111.2, 55.9, 53.0, 52.8, 39.9, 37.0, 34.0; **HRMS (ESI)** m/z calcd for $C_{30}H_{26}NO_7^+$ $(M+H)^+$ 512.17038, found 512.17078.

Deuterium-labeling experiments

In the presence of D_2O:

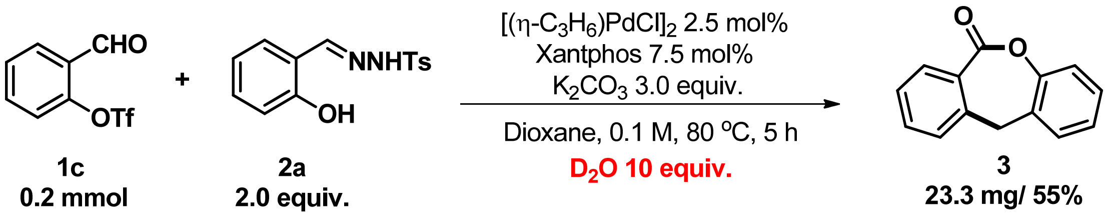

An oven-dried reaction tube containing a stirring bar was charged with [(η-C_3H_6)PdCl]$_2$ (2.5 mol%), Xantphos (7.5 mol%), K_2CO_3 (3.0 equiv.) and **2a** (0.4 mmol, 116 mg). After degassed and filled with dry argon repeated three times, dioxane (2.0 mL), **1c** (0.2 mmol, 51 mg) and D_2O (10.0 equiv.) were added via syringe. The mixture was stirred at 80 ^{o}C for 5 h. The crude mixture was cooled to room temperature. EtOAc was added to the mixture. The reaction mixture was filtered through a short pad of celite. After removing the solvent under reduced pressure, the residual was purified by silica gel column chromatography to obtain the seven-membered lactone (**3**).

8.2 [D]-1c instead of 1c:

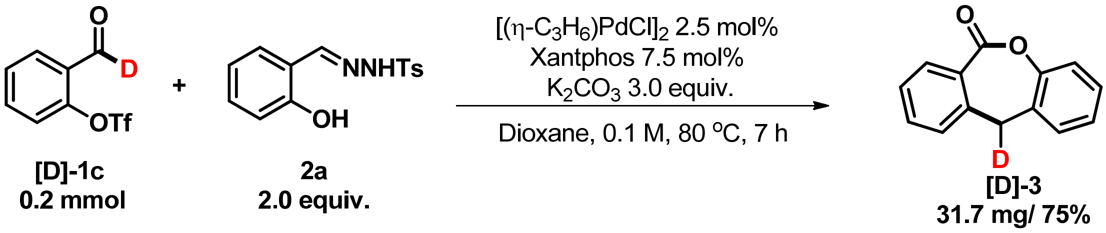

Following the general procedure for the synthesis of seven-membered lactone, the reaction was generate the deuterated product (**[D]-3**) in 75% yield. **^{1}H NMR (400 MHz, $CDCl_3$)** δ 7.89 (dd, J_1 = 7.6 Hz, J_2 = 0.8 Hz, 1H), 7.46 (td, J_1 = 7.6 Hz, J_2 = 1.2

Hz, 1H), 7.32 (td, J_1 = 7.6 Hz, J_2 = 0.8 Hz, 1H), 7.27-7.19 (m, 4H), 7.15-7.10 (m, 1H), 3.99 (s, 1H); **^{13}C NMR (101 MHz, $CDCl_3$)** δ 166.1, 150.6, 142.5, 133.4, 132.7, 132.6, 128.13, 128.10, 128.0, 127.4, 127.1, 125.8, 120.7, 37.4, 37.1 (t, J = 20.0 Hz).

Kinetic isotope effect experiment:

a) One-pot experiment

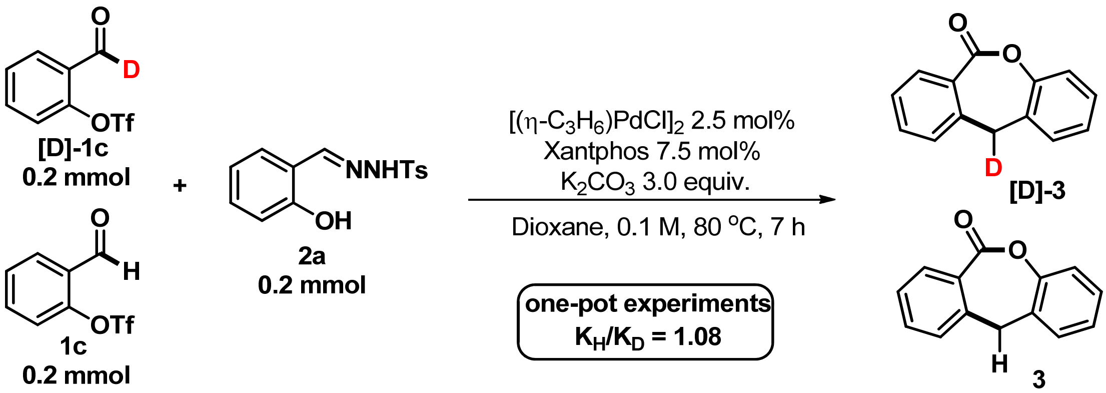

One-pot experiment was conducted using 0.2 mmol **1c**, 0.2 mmol **[D]-1c,** 0.2 mmol **2a** as the substrates. Following the general procedure for the synthesis of seven-membered lactone, the reaction was stopped after 7 h. The crude mixture was cooled to room temperature. EtOAc was added to the mixture. The reaction mixture was filtered through a short pad of celite. After removing the solvent under reduced pressure, the residual was purified by silica gel column chromatography to obtain the seven-membered lactone (**3**/**[D]-3**) compound. The KIE value (K_H/K_D = 0.96) was determined on the basis of ^{1}H NMR analysis.

b) Parallel experiments

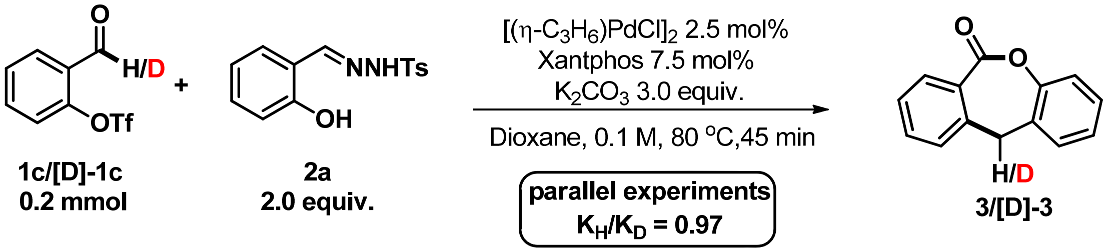

1c/[D]-1c
0.2 mmol

2a
2.0 equiv.

3/[D]-3

Parallel experiments were conducted using **1c** and **[D]-1c** as substrates. Following the general procedure for the synthesis of seven-membered lactone, the two reactions were stopped after 45 min. The crude mixture was cooled to room temperature. EtOAc was added to the mixture. The mixture was filtered through Celite. The solvents were evaporated and the yields of the products (**3/[D]-3**) were estimated by GC using *n*-decane as the internal standard. KIE = 8.5/8.8 = 0.97 was obtained.

Representative synthetic application

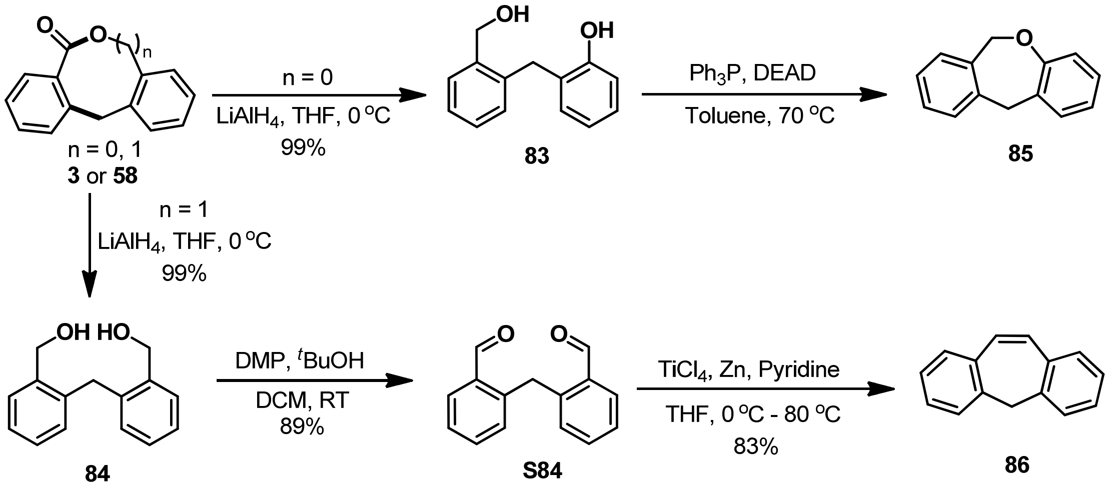

To a mixture of $LiAlH_4$ (1.01 equiv) in 3 mL of THF at 0 °C, a solution of **3** (161 mg, 0.76 mmol) in 7 mL of THF was added slowly. The reaction mixture was stirred for 4 h and carefully quenched with 2 mL of aq. NH_4Cl solution. 15 mL of EtOAc and 3 mL of 10% aq. NaOH solution were added sequentially and stirred for another 15

minutes at room temperature. The resulting mixture was then filtered. The filtrate was extracted with EtOAc (3×15 mL). The combined EtOAc layer was washed with 5 mL of 10% aq. HCl, brine and dried over Na_2SO_4. The solvent was removed under reduced pressure. The residues were purified by column chromatography on silica gel to get product (**83**) (162 mg, 99%) as a white solid. R_f = 0.5 (petroleum ether : ethyl acetate = 2 : 1); **^{1}H NMR (400 MHz, CD_3CN)** δ 7.11 (s, 1H), 7.00-6.79 (m, 1H), 6.82-6.80 (m, 2H), 6.73-6.65 (m, 3H), 6.44-6.40 (m, 2H), 4.29 (d, *J* = 4.8 Hz, 2H), 3.60 (s, 2H), 3.20 (t, *J* = 4.8 Hz, 1H); **^{13}C NMR (101 MHz, CD_3CN)** δ 155.3, 139.5, 139.3, 131.4, 130.2, 128.6, 128.20, 128.15, 127.4, 126.8, 120.4, 115.9, 62.8, 32.2; **HRMS (ESI)** m/z calcd for $C_{14}H_{15}O_2^+$ $(M+H)^+$ 215.10666, found 215.10617.

Compound **85** was prepared from **83** using the literature procedure.[8] Diol **84** (128.4 mg, 0.60 mmol), triphenylphosphine (165.2 mg, 0.63 mmol) in toluene (6 mL) solution of diethyl diazene-1,2-dicarboxylate (95 μL, 0.60 mmol) was added dropwise at room temperature, the reaction mixture was stirred under argon 6 hours at 70 °C. The solvent of the reaction mixture was evaporated, and the residue was purified by column chromatography to give compound **85** (116.8 mg, 98%) as light yellow liquid; R_f = 0.7 (petroleum ether : ethyl acetate = 50 : 1); **^{1}H NMR (400 MHz, CD_3CN)** δ 7.06-6.97 (m, 1H), 6.87 (d, *J* = 7.2 Hz, 1H), 6.82 (t, *J* = 7.2 Hz, 1H), 6.58 (t, *J* = 7.2 Hz, 1H), 6.48 (d, *J* = 8.4 Hz, 1H), 5.04 (s, 1H), 3.98 (s, 1H); **^{13}C NMR (101 MHz, CD_3CN)** δ 157.3, 141.3, 136.6, 131.6, 129.4, 128.8, 128.7, 128.4, 127.7, 126.1, 121.9, 120.0, 70.6, 38.6.

To a mixture of $LiAlH_4$ (2.5 equiv) in 3 mL of THF at 0 °C, a solution of **58** (150 mg, 0.66 mmol) in 7 mL of THF was added slowly. The reaction mixture was stirred for 3 h and carefully quenched with 2 mL of aq. NH_4Cl solution. 15 mL of EtOAc and 3 mL of 10% aq. NaOH solution were added sequentially and stirred for another 15 minutes at room temperature. The resulting mixture was then filtered. The filtrate was extracted with EtOAc (3×15 mL). The combined EtOAc layer was washed with 5 mL of 10% aq. HCl, brine and dried over Na_2SO_4. The solvent was removed under reduced pressure and the residual solid was washed and filtered again using DCM. The pure compound **84** (150 mg, 99%) was obtained as a white solid. R_f = 0.3 (petroleum ether : ethyl acetate = 1 : 1); **^{1}H NMR (600 MHz, DMSO-*d₆*)** δ 7.43 (d, *J* = 7.2 Hz, 2H), 7.21 (td, J_1 = 7.2 Hz, J_2 = 0.6 Hz, 2H), 7.14 (td, J_1 = 7.2 Hz, J_2 = 0.6 Hz, 2H), 6.84 (d, *J* = 7.2 Hz, 2H), 5.13 (t, *J* = 5.4 Hz, 2H), 4.47 (d, *J* = 5.4 Hz, 4H), 3.98 (s, 2H); **^{13}C NMR (151 MHz, DMSO-d_6)** δ 140.7, 137.7, 129.3, 127.4, 127.2, 126.5, 61.2, 34.0; **HRMS (ESI)** m/z calcd for $C_{15}H_{17}O_2^+$ $(M+H)^+$ 229.12231, found 229.12238.

Compound **S84** was prepared from **84** using an oxidation method described in literature.[9] To a stirred mixture of **84** (160 mg, 0.7 mmol) in dichloromethane (10 mL) at room temperature was added sequentially *tert*-butyl alcohol (10 equiv) and Desse-Martin periodinane (3 equiv). After stirring for 5 h, saturated aqueous sodium hydrogen carbonate (5 mL) and saturated aqueous sodium thiosulfate (5 mL) were added to the mixture, which was extracted with dichloromethane (2×20 mL). The organic layer was washed with brine and dried over Na_2SO_4. The solvent was

evaporated under reduced pressure and the residue was purified by silica gel column to get **S84** (140 mg, 89% yield) as colourless liquid; $R_f = 0.3$ (petroleum ether : ethyl acetate = 10 : 1); **^{1}H NMR (400 MHz, $CDCl_3$)** δ 10.16 (s, 2H), 7.84 (d, *J* = 7.2 Hz, 2H), 7.46-7.37 (m, 4H), 7.01 (d, *J* = 7.6 Hz, 2H), 4.88 (s, 2H); **^{13}C NMR (101 MHz, $CDCl_3$)** δ 192.5, 141.9, 133.7, 133.6, 132.8, 130.8, 126.8, 34.6; **HRMS (ESI)** m/z calcd for $C_{15}H_{13}O_2^+$ $(M+H)^+$ 225.09101, found 225.09125.

Compound **86** was prepared from **S84** using the literature procedure.[10] In a two-neck round-bottom flask containing **S84** (60 mg, 0.27 mmol) and zinc dust (6.0 equiv) in dry THF (10 mL) was added pyridine (12.0 equiv). The stirred mixture was cooled to 0 °C and titanium(IV) chloride (3.0 equiv) was added dropwise. The mixture was then heated at 80 °C for 6 h before being cooled to room temperature and quenched with saturated aqueous $NaHCO_3$ solution. It was then filtered through a pad of celite. The filtrate was extracted with DCM (2×20 mL), washed brine and dried over Na_2SO_4. Evaporation of the solvent under reduced pressure and purification of the crude product by column chromatography afforded **86** (43 mg, 83%) as white solid; $R_f = 0.6$ (petroleum ether : ethyl acetate = 20 : 1); **^{1}H NMR (400 MHz, $CDCl_3$)** δ 7.35-7.29 (m, 6H), 7.22 (td, J_1 = 7.2 Hz, J_2 = 1.6 Hz, 2H), 7.05 (s, 2H); 3.76 (s, 2H); **^{13}C NMR (101 MHz, $CDCl_3$)** δ 138.1, 135.1, 131.5, 128.4, 128.1, 127.8, 126.0, 41.6; **HRMS (ESI)** m/z calcd for $C_{15}H_{13}^+$ $(M+H)^+$ 193.10118, found 193.10083

Computational details

The geometries were optimized by hybrid functional B3LYP[11-12] with the LANL2DZ[13-15] effective core potential for Pd and Br, and 6-31G* basis set for the rest atoms. The same level calculation of vibrational frequency was carried out to check the stationary point or transition state as minima or saddle point, and to obtain the thermodynamic corrections. Single point energy was performed by meta-GGA hybrid functional M06[16] with def2-TZVP[17] basis set for all atoms. Solvation energy was obtained by SMD[18] continuum solvation model in 1,4-Dioxane solution. The changes in Gibbs free energy with single point and solvation energy are reported in the content. All the calculations were performed in Gaussian09 package.[19]

Pathway for ligand dissociation

Following one reviewer's suggestion, the possibility of the pathway through attack of **2a'** to **Int2** was further explored. However, all attempts to locate an intermediate with one Pd-P opened with **2a'** failed, presumably because the steric hindrance of ligands prevents the binding of **2a'** to the metal center.

In order to compare with the favorable pathway already shown in the manuscript, we further explored another two possible reaction pathways. As can be seen from the following Fig. SII-1, the direct dissociation of Br^- anion from **Int2** to **Int3'** is energetically unfavorable as this process requires the energy of 48.9 kcal/mol. Another process involves the dissociation of one arm of the bidentated ligand to form **Int3** without counter anion exchange, followed by the reaction of **Int3** with **2a'** leading to dediazonation**.** Such a process is energetically unfavorable as it needs to

overcome a large barrier of 38.2 kcal/mol relative to **Int2**. All in all, these two pathways are not favored compared to the one shown in Figure 3 in the manuscript.

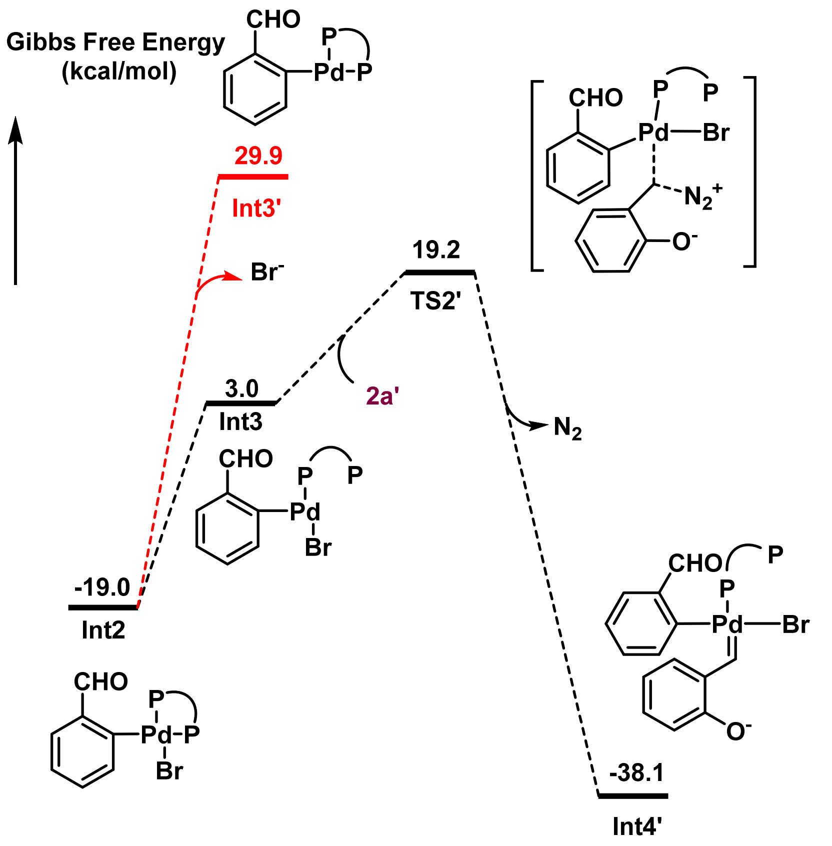

Supplementary Figure 3. Reaction Energy Profiles of Ligand Dissociation Calculated at M06/def2-TZVP//B3LYP/6-31G(d)(LANL2DZ) Level.

Crystal data and structure refinement for 66

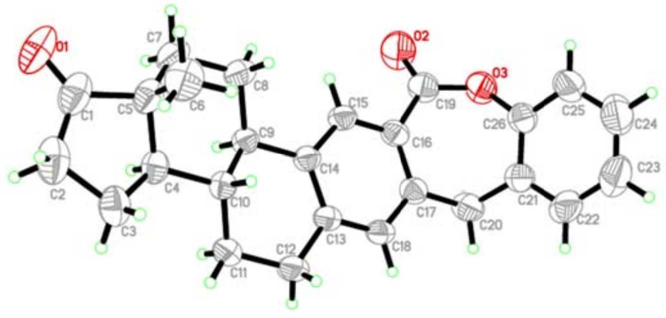

Supplementary Figure 4. X-ray structure of **66**. The thermal ellipsoid was drawn at the 50% probability level.

Empirical formula	$C_{26}H_{26}O_3$
Formula weight	386.47
Temperature	293(2) K
Wavelength	0.71073 A
Crystal system, space group	Orthorhombic, P212121
Unit cell dimensions	a = 9.841(2) Å alpha = 90 deg.
	b = 13.449(3) Å beta = 90 deg.
	c = 15.601(3) Å gamma = 90 deg.
Volume	2064.8(7) $Å^3$
Z, Calculated density	4, 1.243 Mg/m^3
Absorption coefficient	0.080 mm^{-1}
F(000)	824
Crystal size	0.90 x 0.45 x 0.30 mm
Theta range for data collection	2.88 to 27.48 deg.
Limiting indices	-12<=h<=12, -17<=k<=16, -20<=l<=20

Reflections collected / unique	16011 / 4666 [R(int) = 0.0219]
Completeness to theta = 27.48	98.7 %
Refinement method	Full-matrix least-squares on F^2
Data / restraints / parameters	4666 / 0 / 264
Goodness-of-fit on F^2	1.077
Final R indices [I>2sigma(I)]	R_1 = 0.0370, wR_2 = 0.1000
R indices (all data)	R_1 = 0.0383, wR_2 = 0.1012
Absolute structure parameter	1.4(11)
Largest diff. peak and hole	0.160 and -0.157 e.A^{-3}

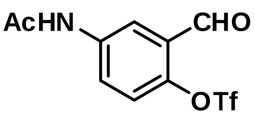

S1t

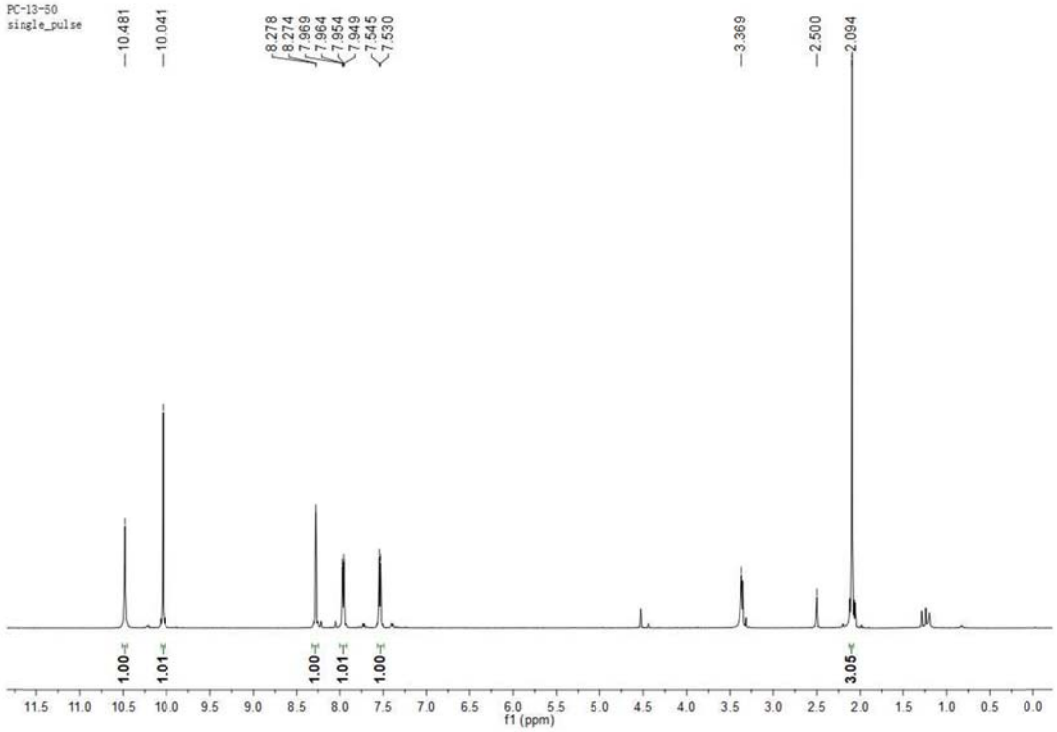

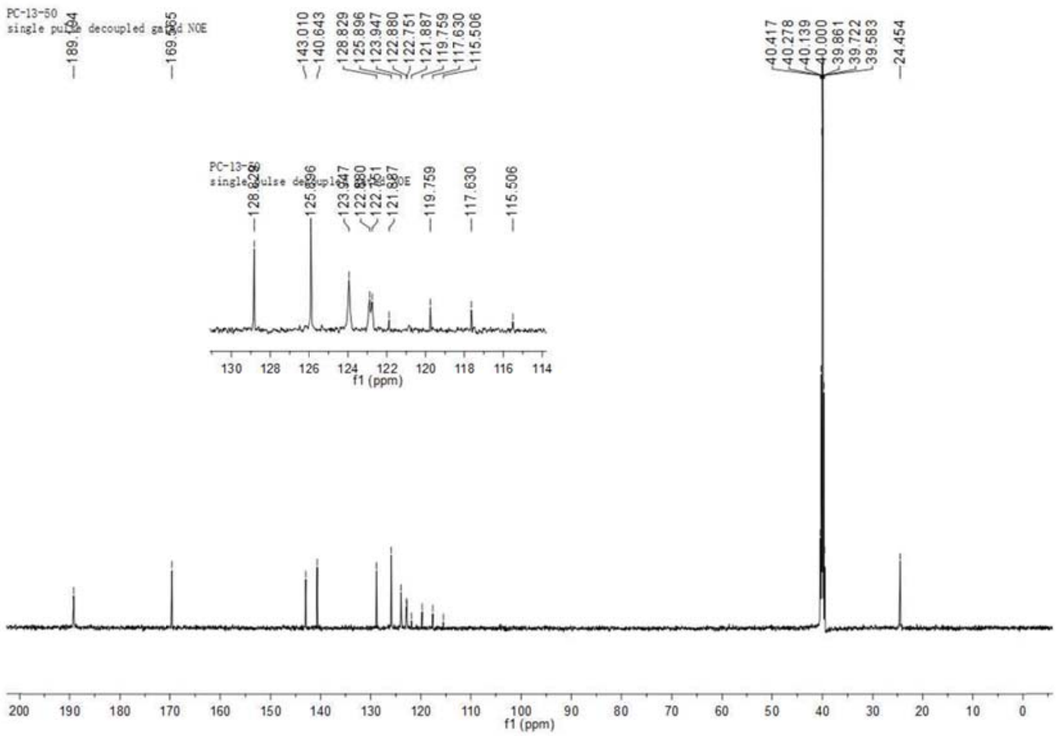

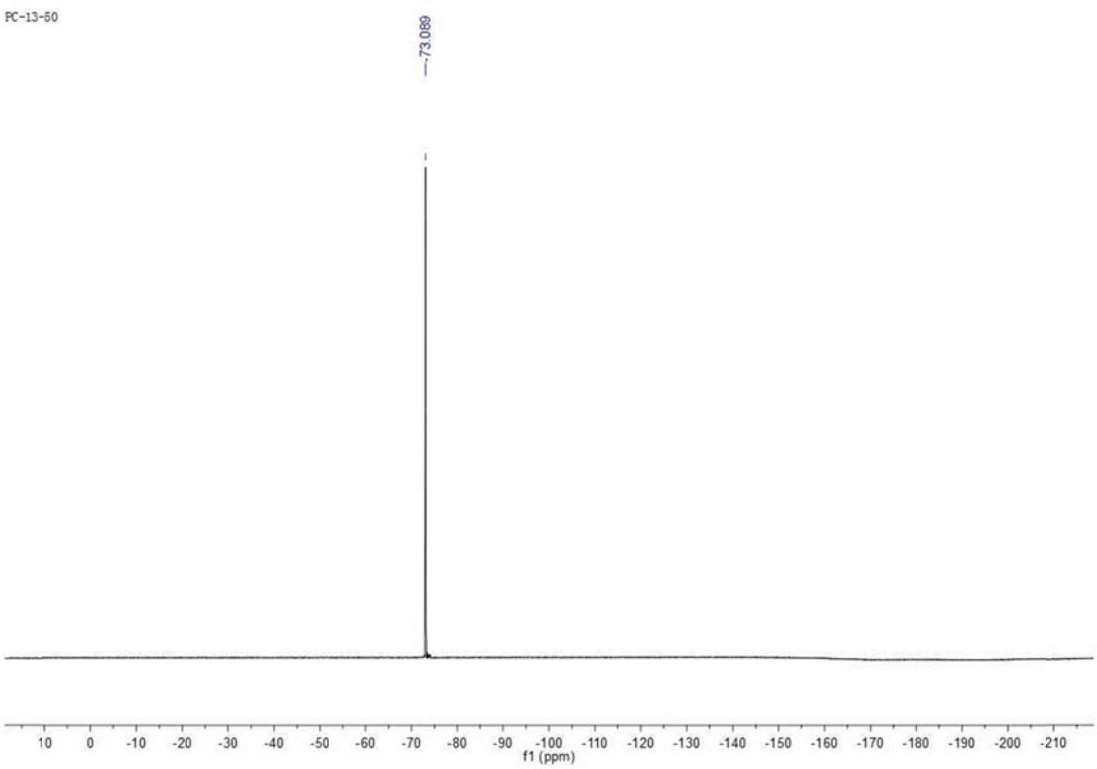

Supplementary Figure 5 ^{1}H NMR, ^{13}C NMR and ^{19}F NMR spectra for compound S1t.

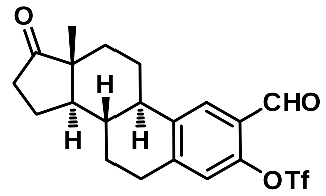
O
H
CHO
H
H
OTf

S1w

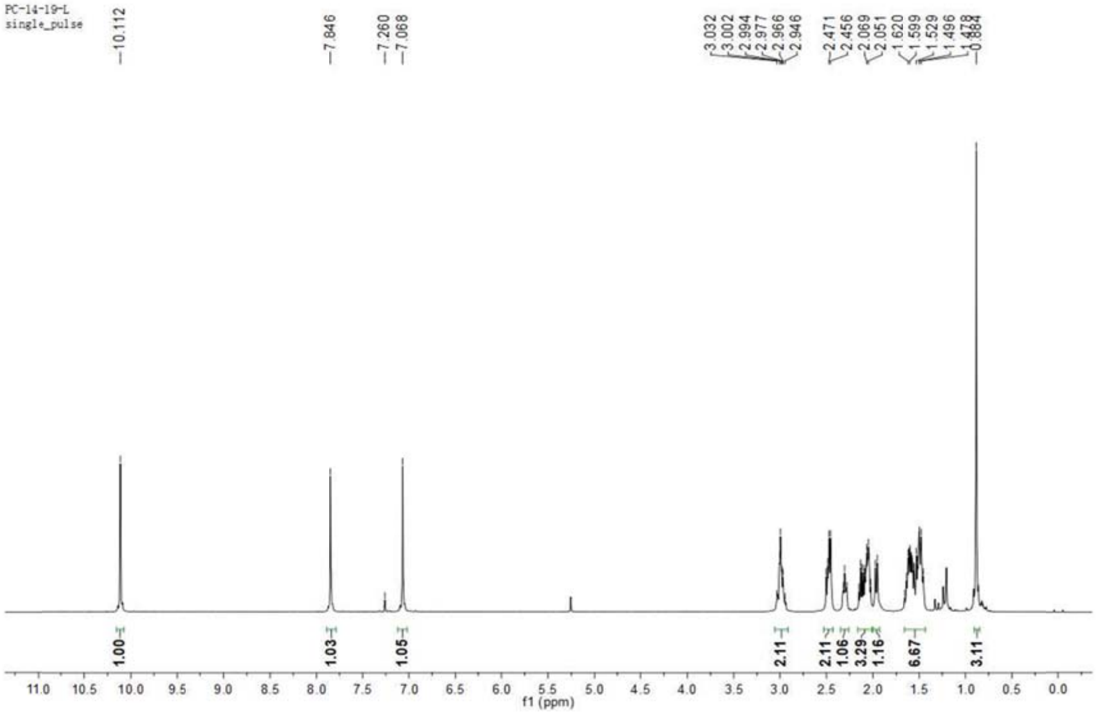
PC-14-19-L
single_pulse
10.112
7.846
7.260
7.068
3.032
3.002
2.994
2.977
2.966
2.946
2.471
2.456
2.069
2.051
1.620
1.599
1.529
1.496
0.884
1.00
1.03
1.05
2.11
2.11
1.06
3.29
1.16
6.67
3.11
f1 (ppm)

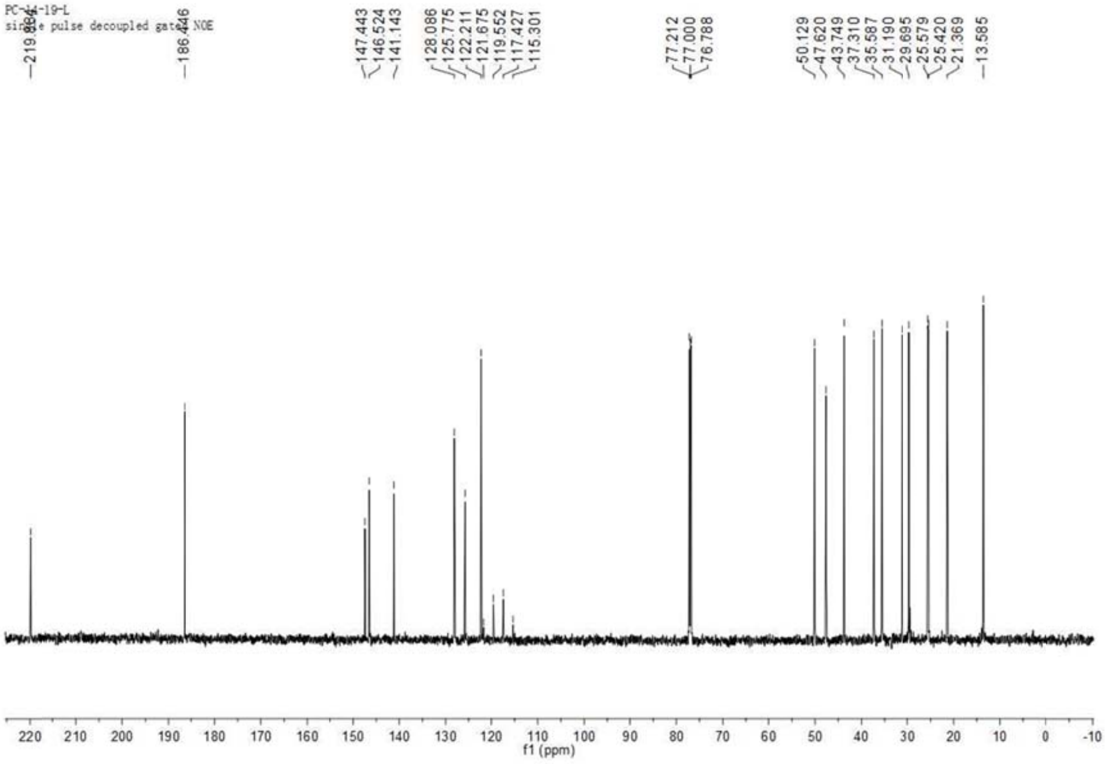
PC-14-19-L
single pulse decoupled gated NOE
147.443
146.524
141.143
128.086
125.775
122.211
121.675
119.552
117.427
115.301
77.212
77.000
76.788
50.129
47.620
43.749
37.310
35.587
31.190
29.695
25.579
25.420
21.369
13.585
f1 (ppm)

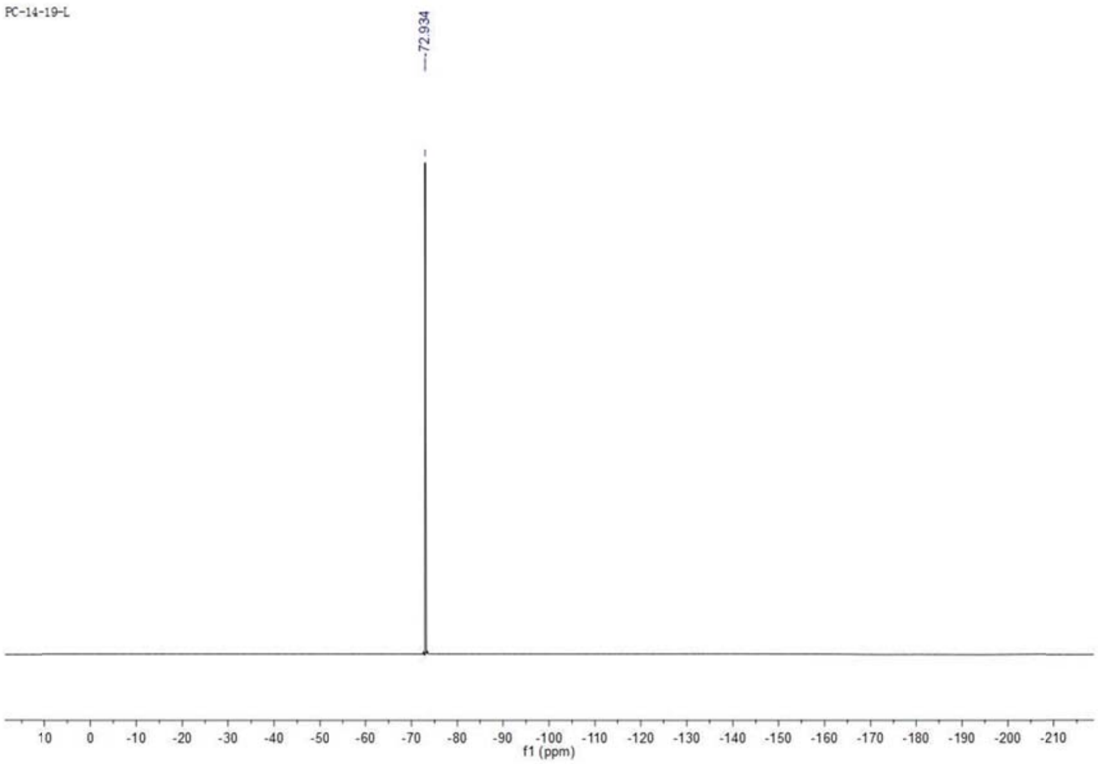

Supplementary Figure 6 ^{1}H NMR, ^{13}C NMR and ^{19}F NMR spectra for compound S1w.

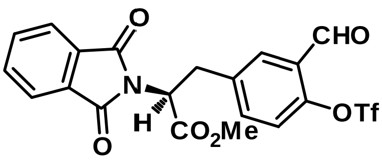
O
N
H
CO2Me
CHO
OTf
O

S1x

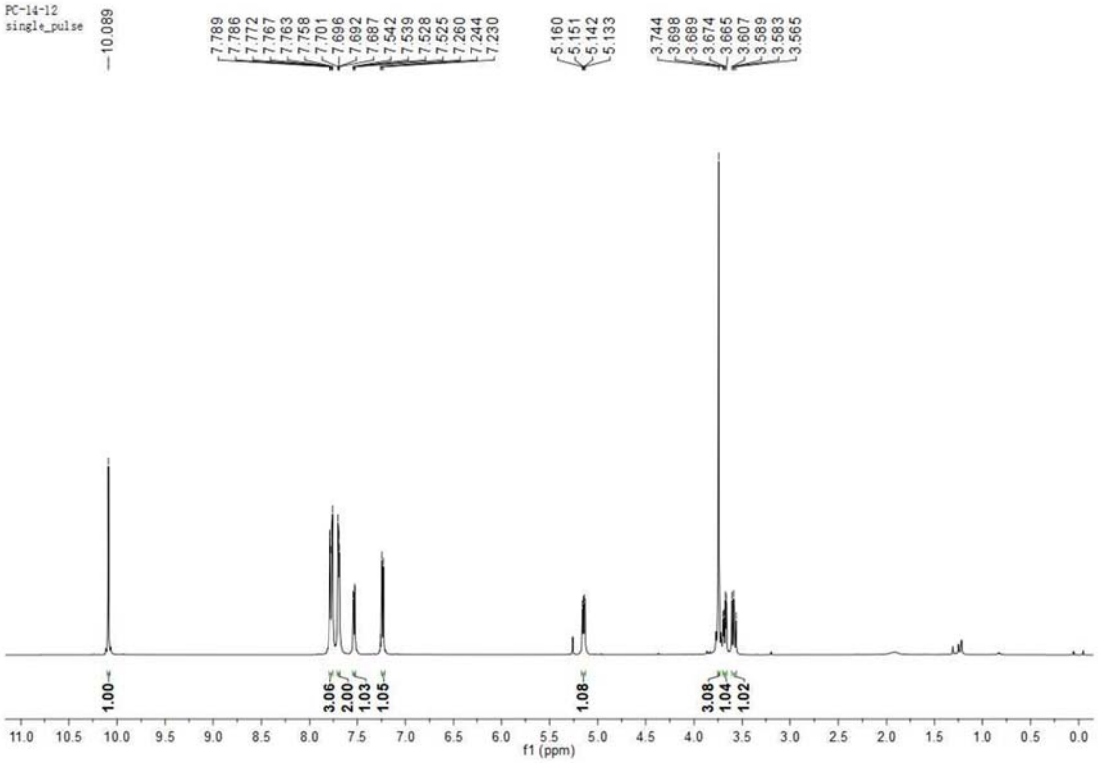
PC-14-12
single_pulse
10.089
7.789
7.786
7.772
7.767
7.763
7.758
7.701
7.696
7.692
7.687
7.542
7.539
7.528
7.525
7.260
7.244
7.230
5.160
5.151
5.142
5.133
3.744
3.698
3.689
3.674
3.665
3.607
3.589
3.583
3.565
1.00
3.06
2.00
1.03
1.05
1.08
3.08
1.04
1.02
11.0 10.5 10.0 9.5 9.0 8.5 8.0 7.5 7.0 6.5 6.0 5.5 5.0 4.5 4.0 3.5 3.0 2.5 2.0 1.5 1.0 0.5 0.0
f1 (ppm)

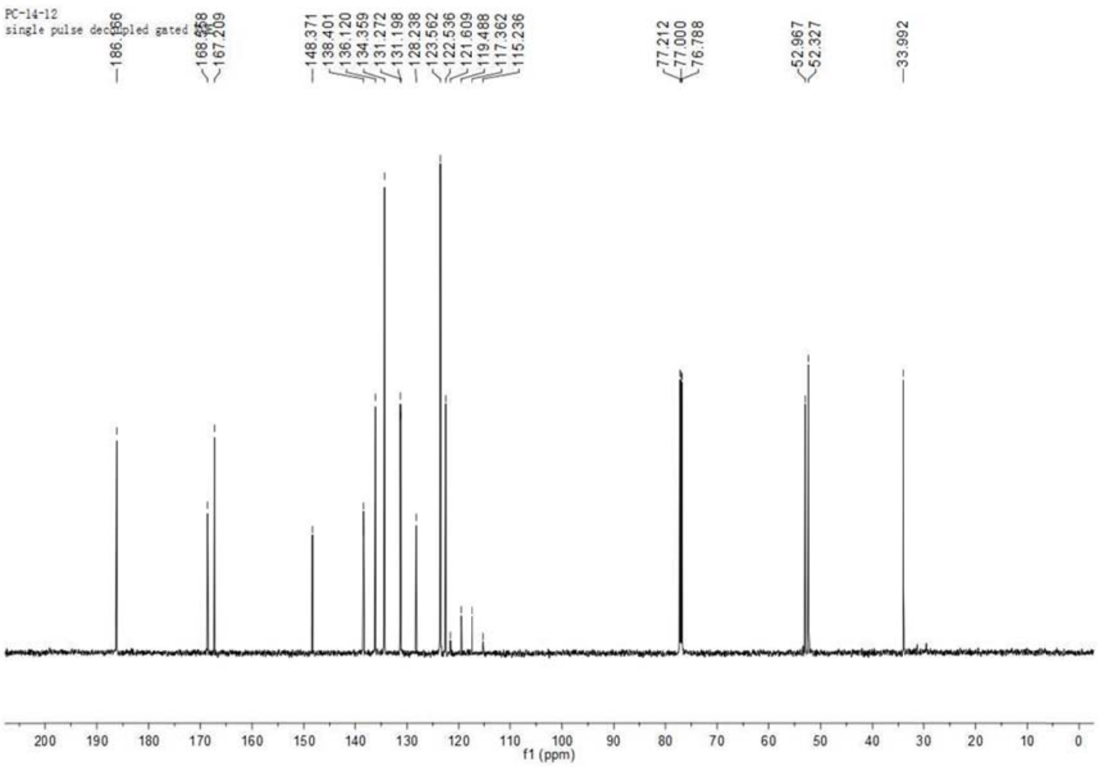
PC-14-12
167.209
148.371
138.401
136.120
134.359
131.272
131.198
128.238
123.562
122.536
121.609
119.488
117.362
115.236
77.212
77.000
76.788
52.967
52.327
33.992
200 190 180 170 160 150 140 130 120 110 100 90 80 70 60 50 40 30 20 10 0
f1 (ppm)

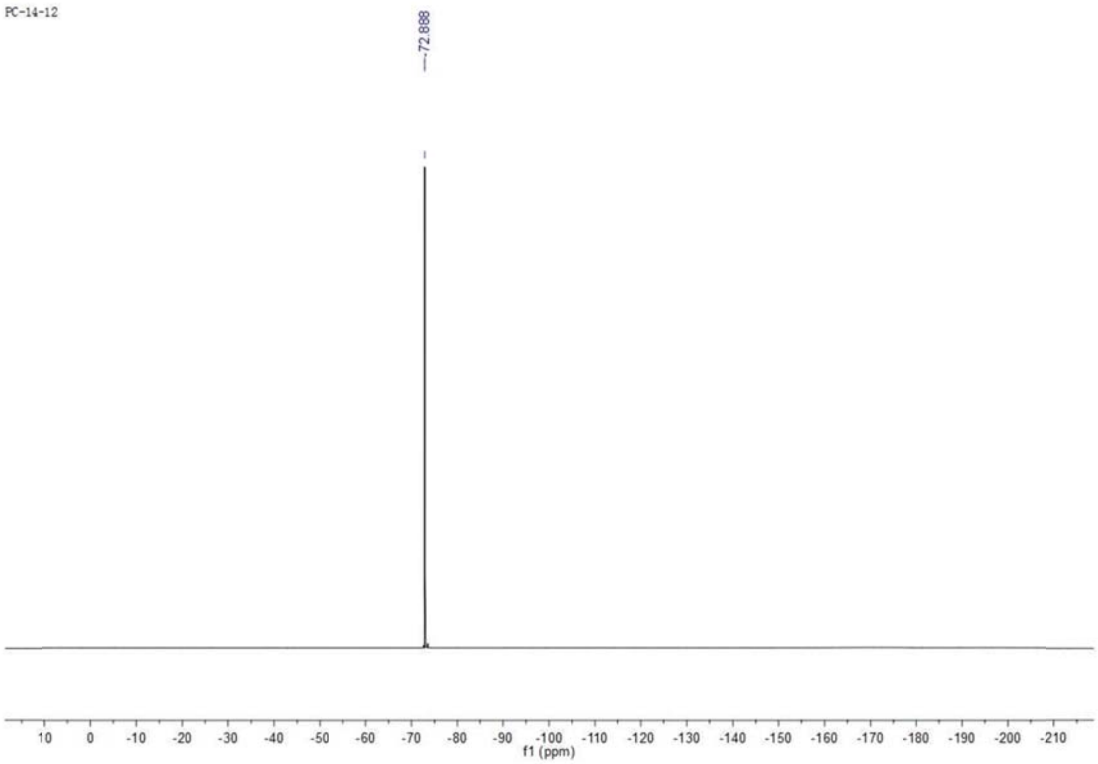

Supplementary Figure 7 ^{1}H NMR, ^{13}C NMR and ^{19}F NMR spectra for compound S1x

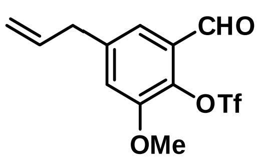
CHO
OTf
OMe

S1y

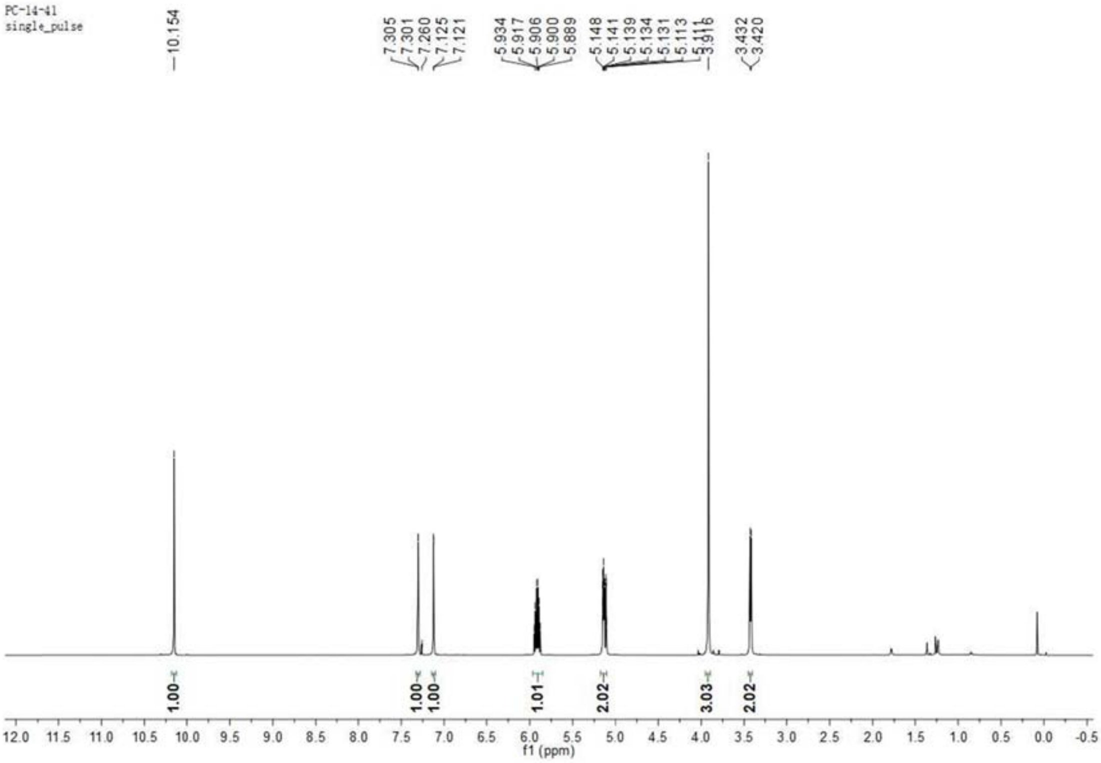
PC-14-41
single_pulse
10.154
7.305
7.301
7.260
7.125
7.121
5.934
5.917
5.906
5.900
5.889
5.148
5.141
5.139
5.134
5.131
5.113
3.916
3.432
3.420
1.00
1.00
1.00
1.01
2.02
3.03
2.02
f1 (ppm)

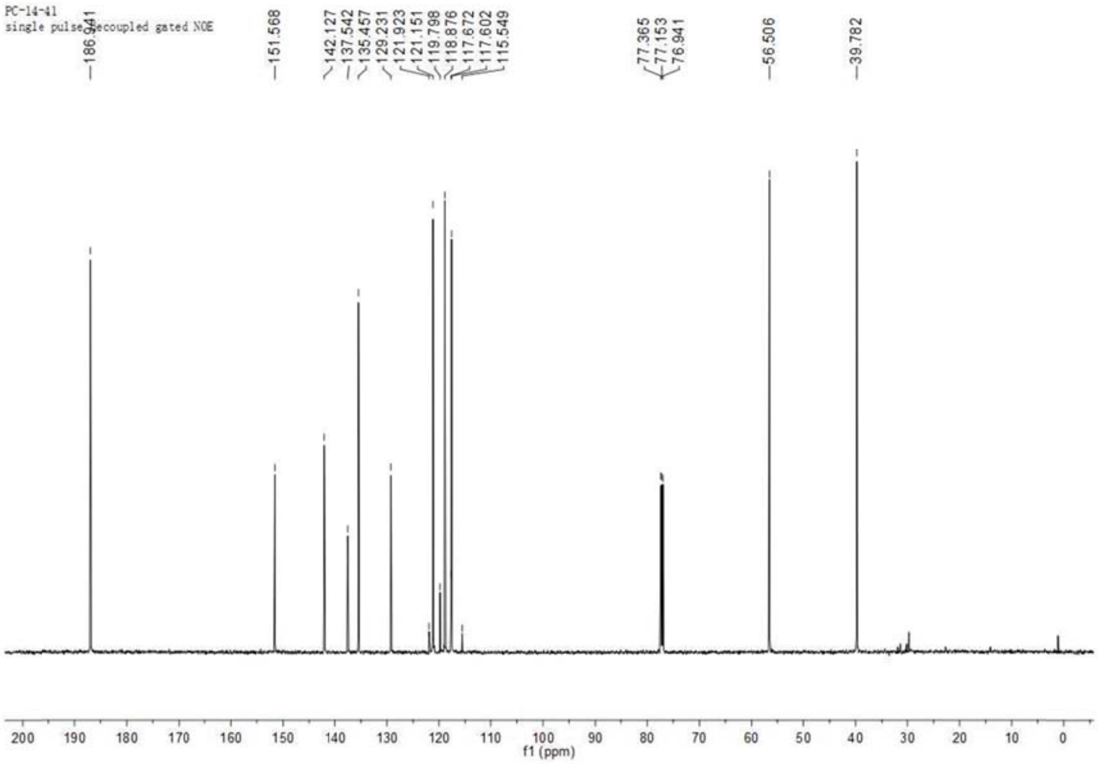
PC-14-41
single pulse decoupled gated NOE
151.568
142.127
137.542
135.457
129.231
121.923
121.151
119.798
118.876
117.672
117.602
115.549
77.365
77.153
76.941
56.506
39.782
f1 (ppm)

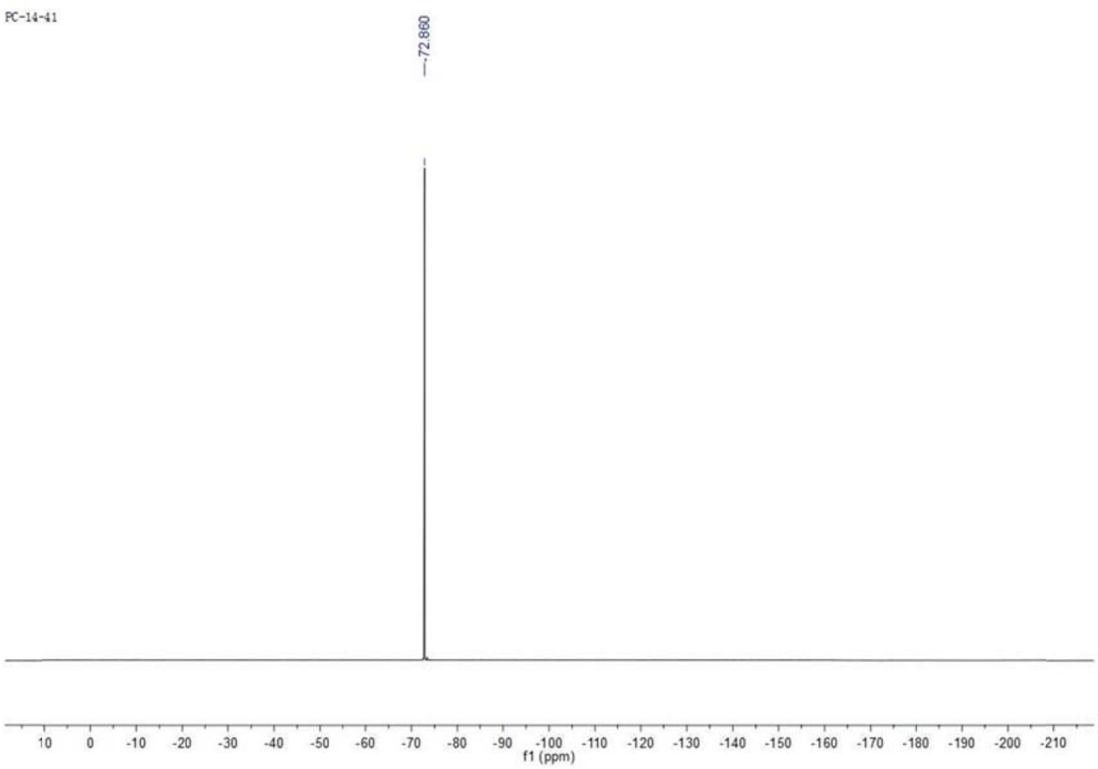

Supplementary Figure 8 ^{1}H NMR, ^{13}C NMR and ^{19}F NMR spectra for compound S1y

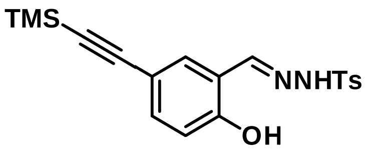

S2o

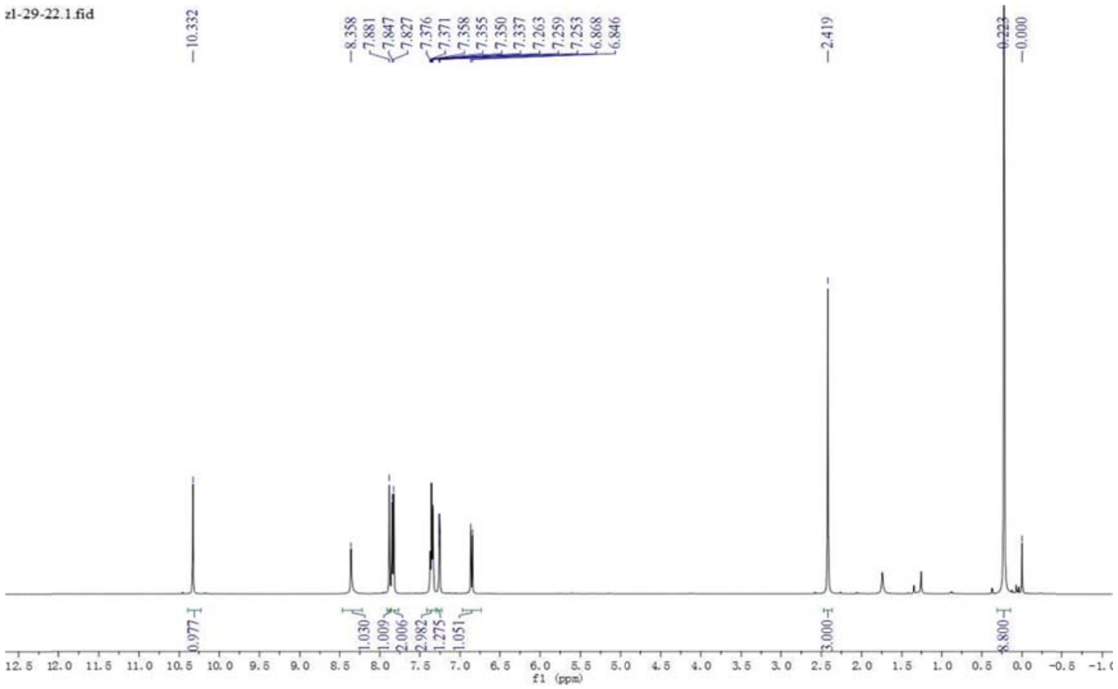

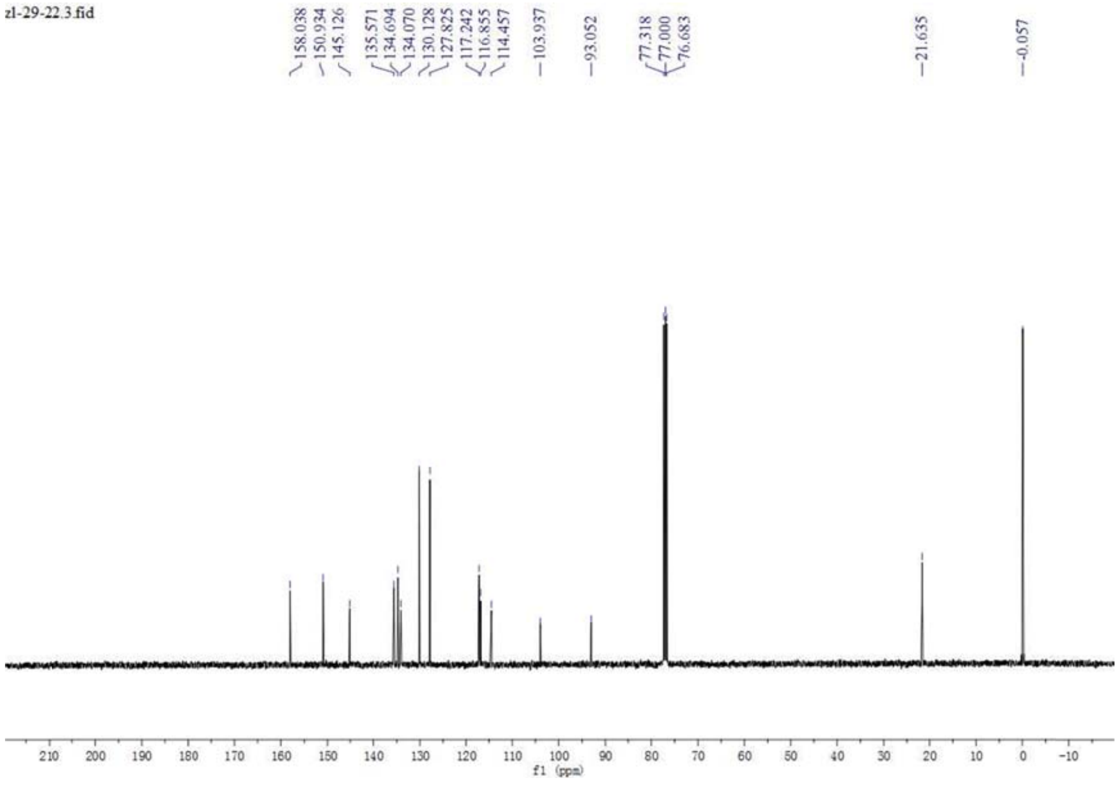

Supplementary Figure 9 ^{1}H NMR and ^{13}C NMR spectra for compound S2o

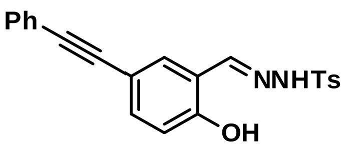

S2p

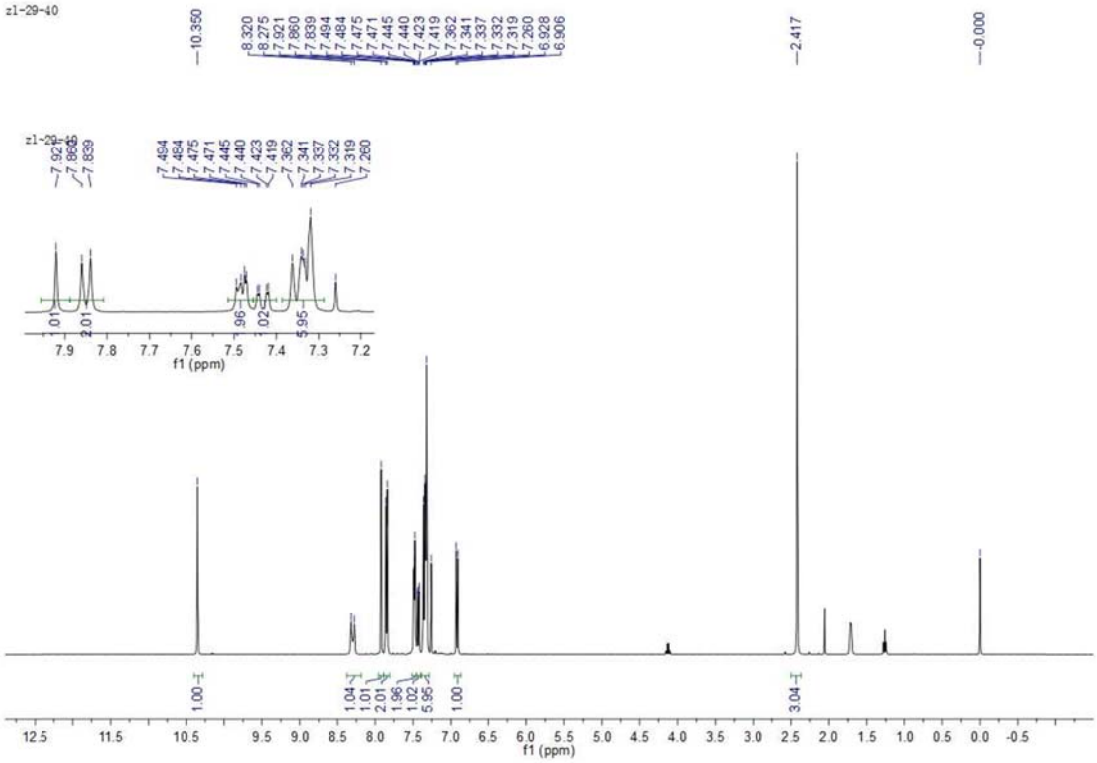

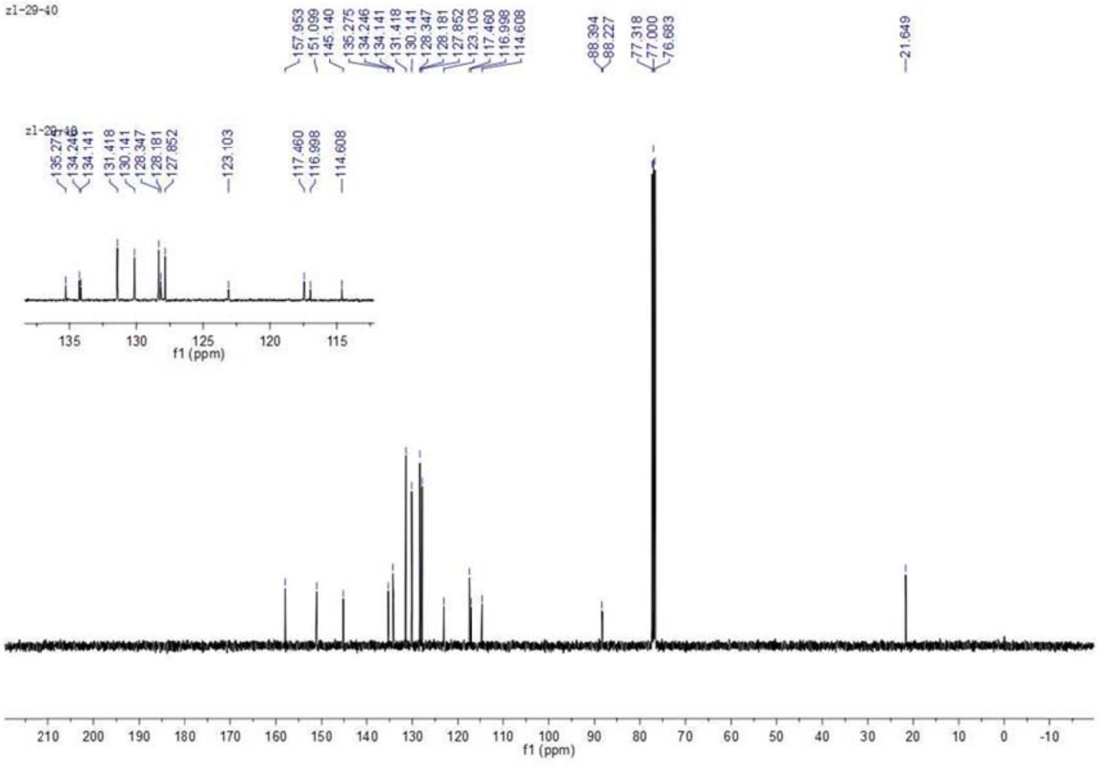

Supplementary Figure 10 ^{1}H NMR and ^{13}C NMR spectra for compound S2p

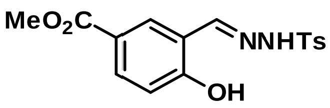

S2m

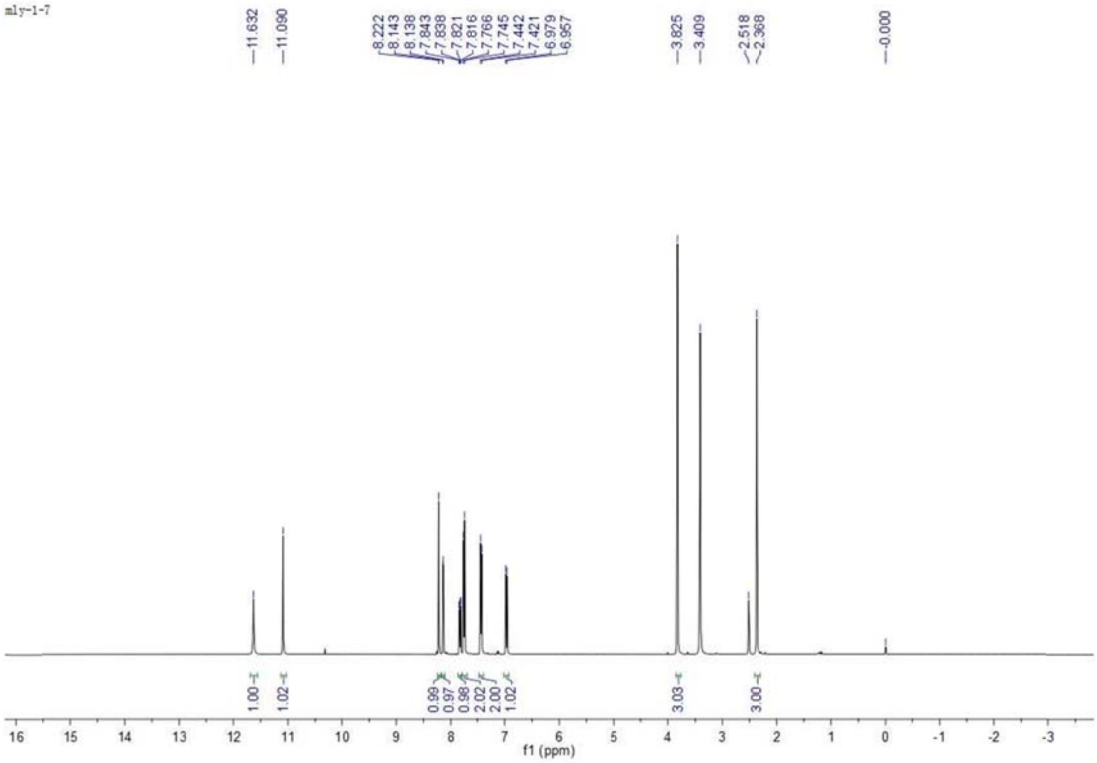

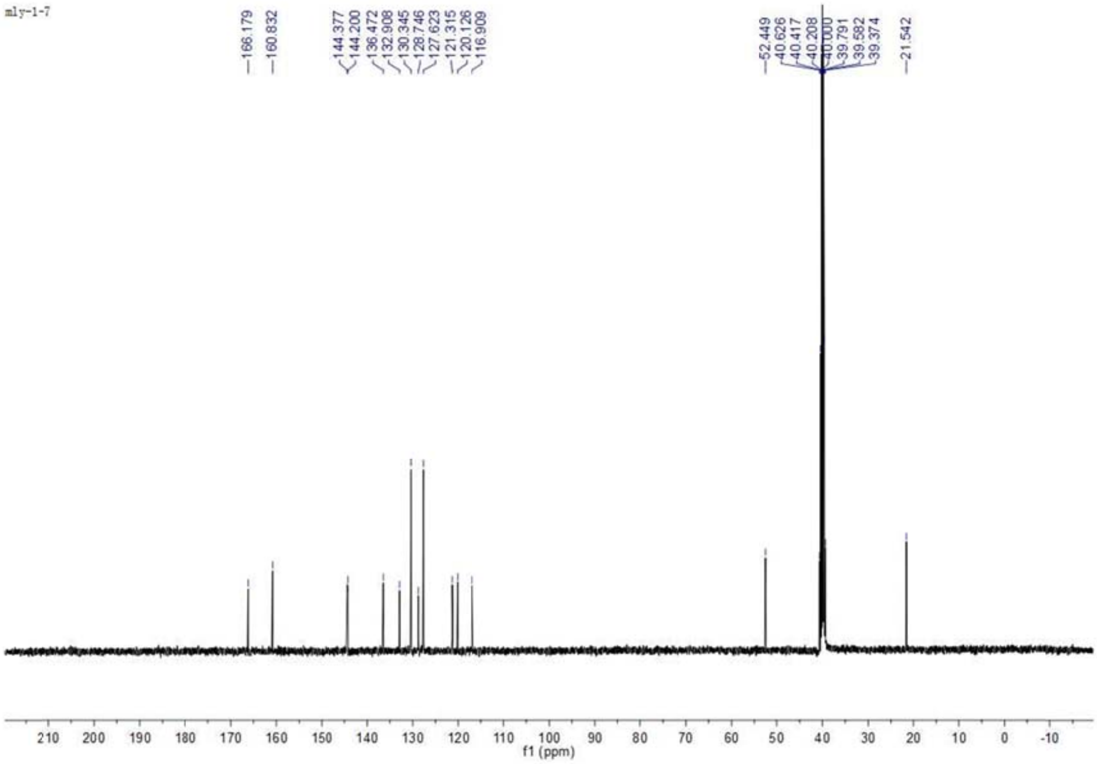

Supplementary Figure 11 ^{1}H NMR and ^{13}C NMR spectra for compound S2m

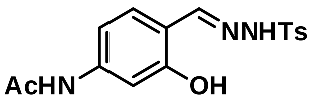

S2n

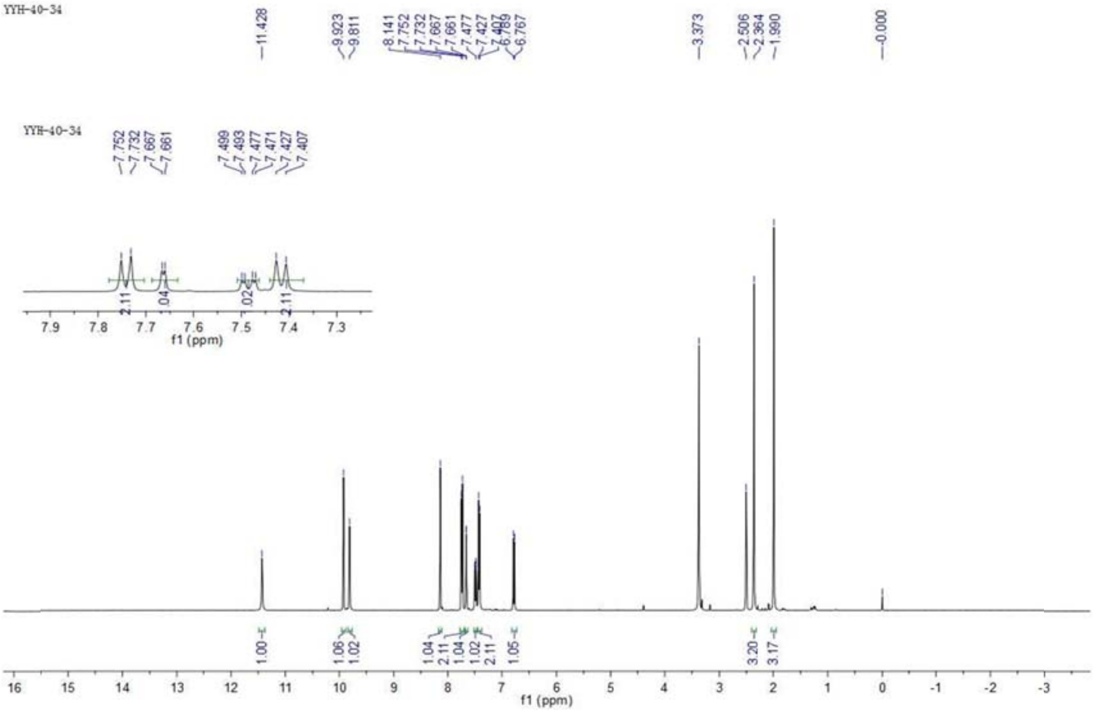

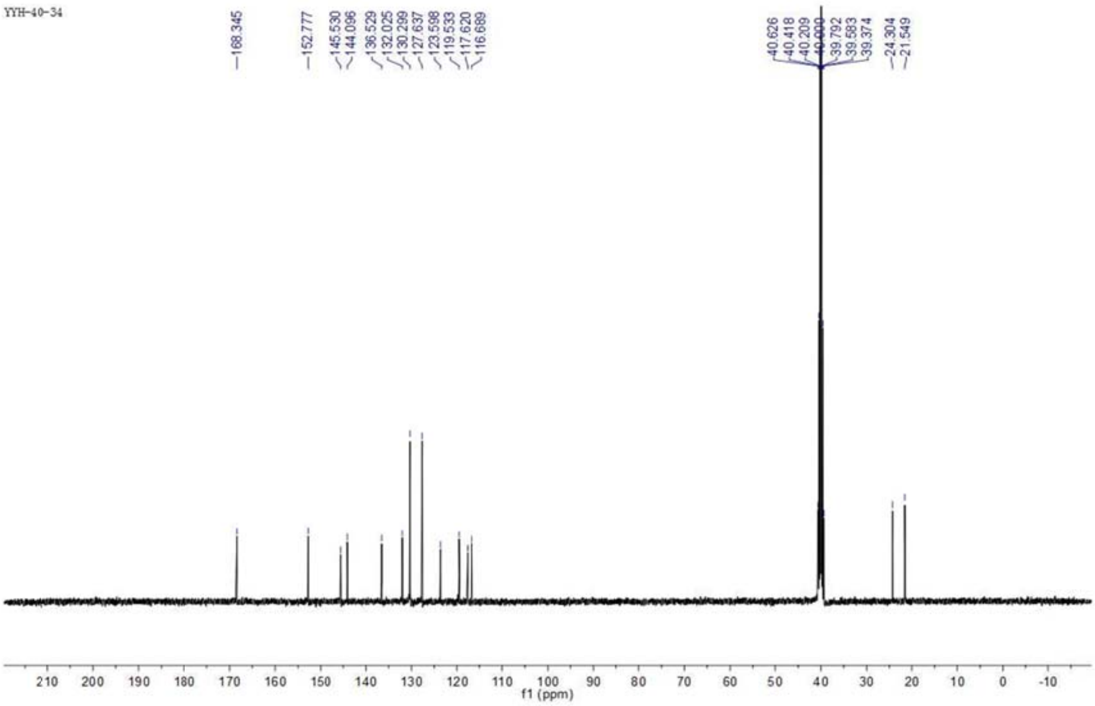

Supplementary Figure 11 ^{1}H NMR and ^{13}C NMR spectra for compound S2n

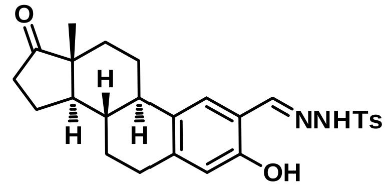

S2u

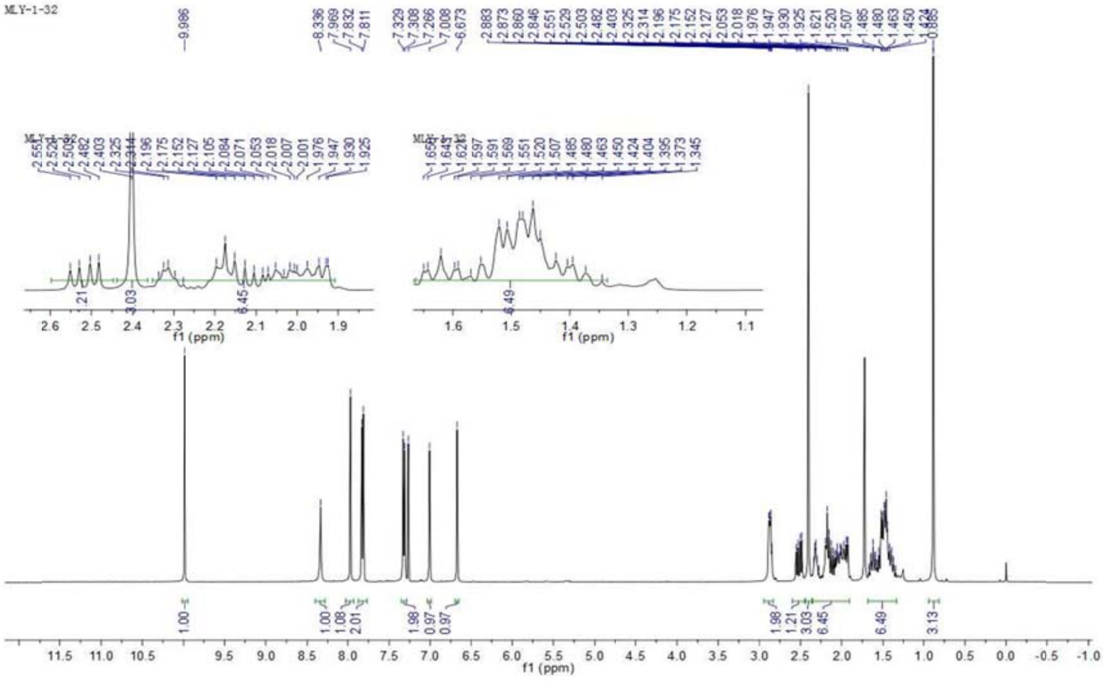

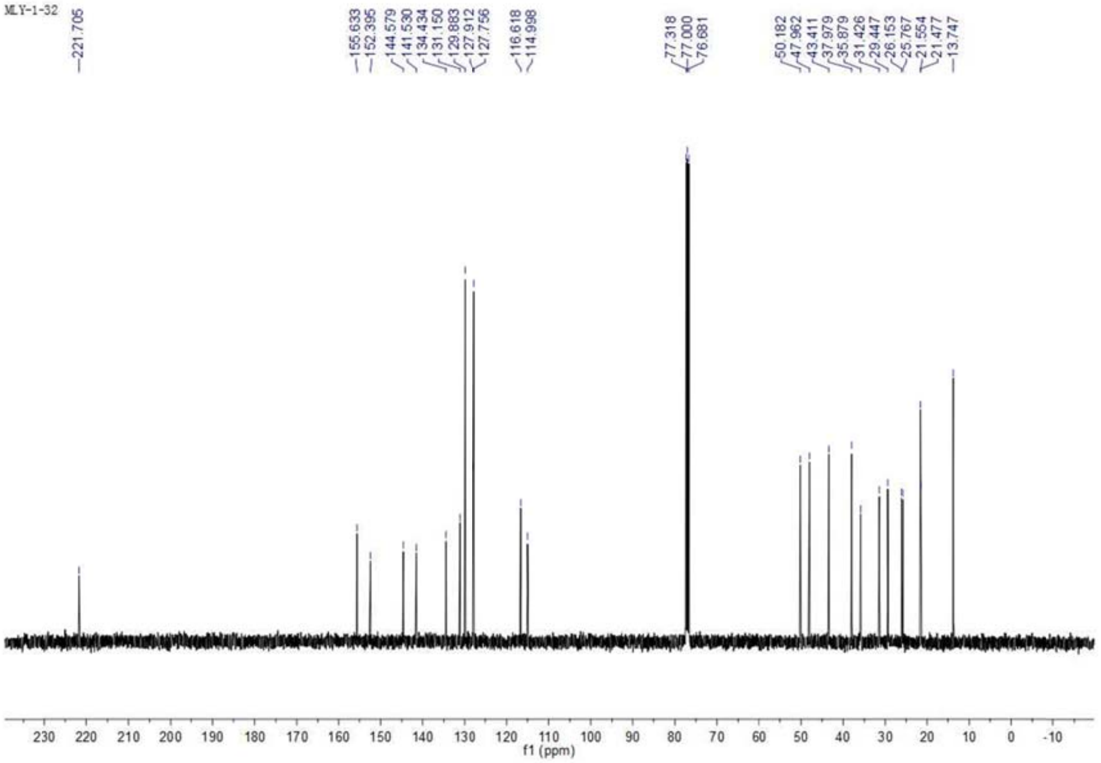

Supplementary Figure 12 ^{1}H NMR and ^{13}C NMR spectra for compound S2u

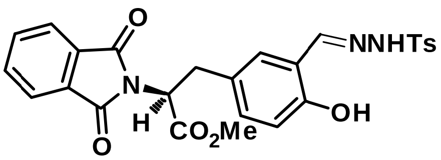

S2v

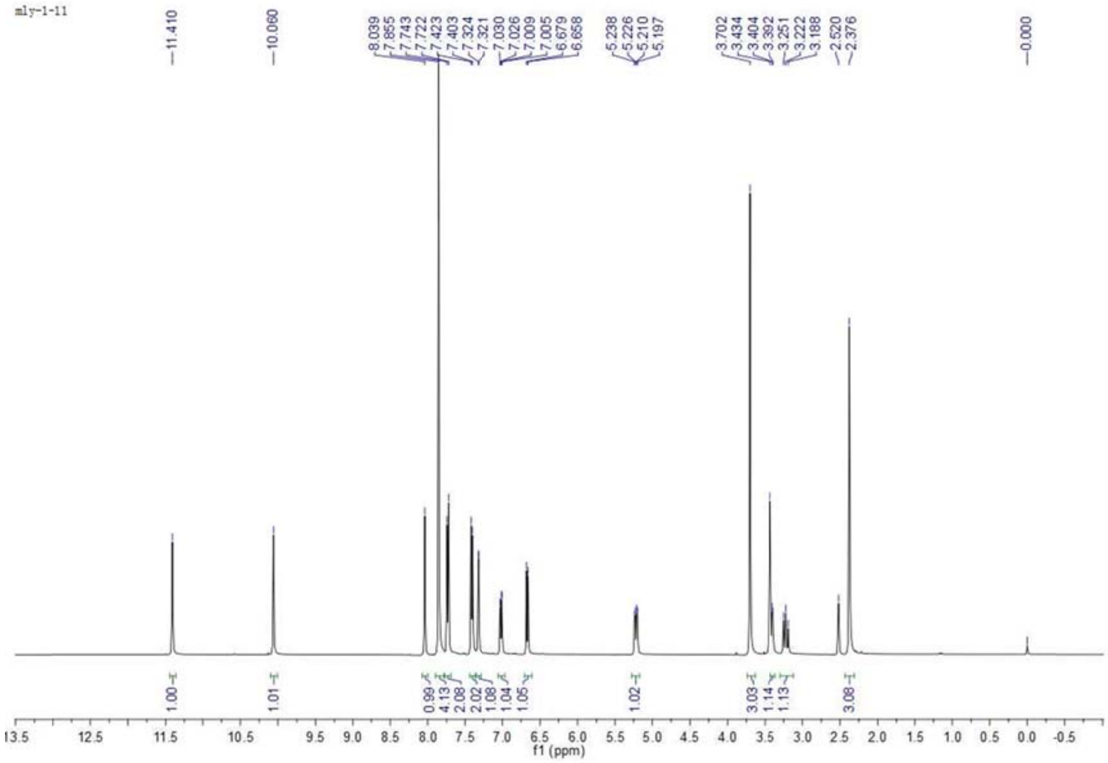

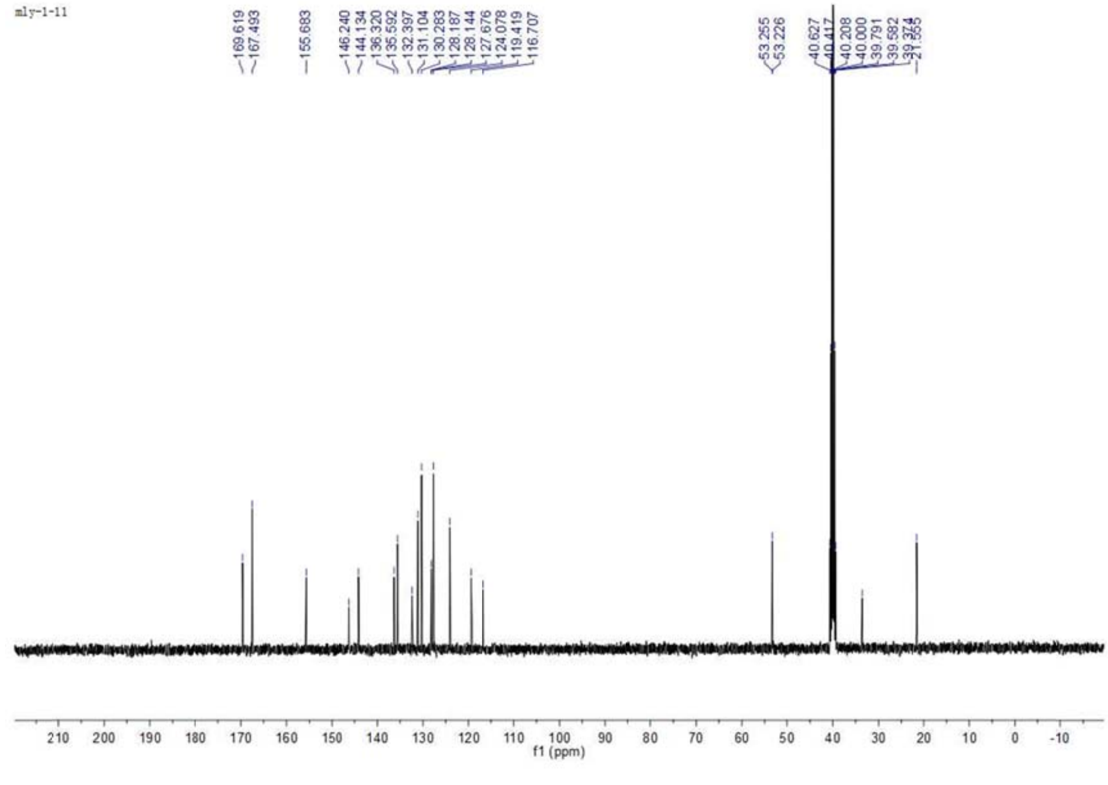

Supplementary Figure 13 ^{1}H NMR and ^{13}C NMR spectra for compound S2v

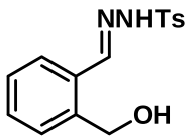

S2w

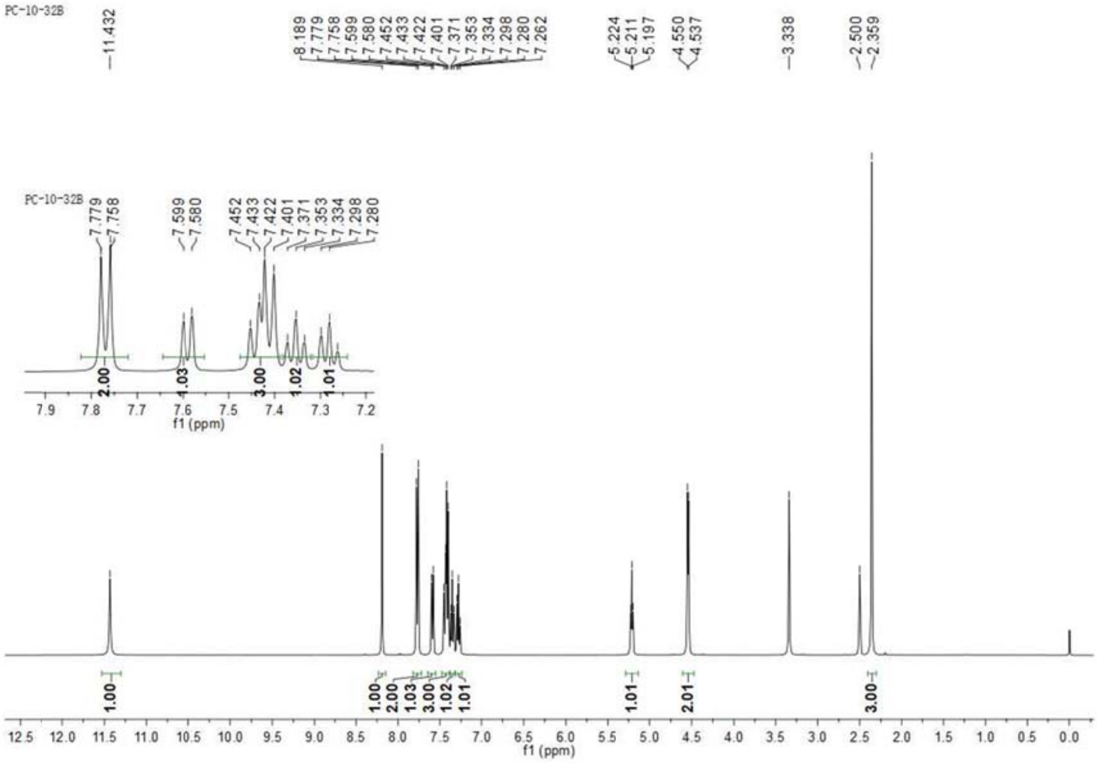

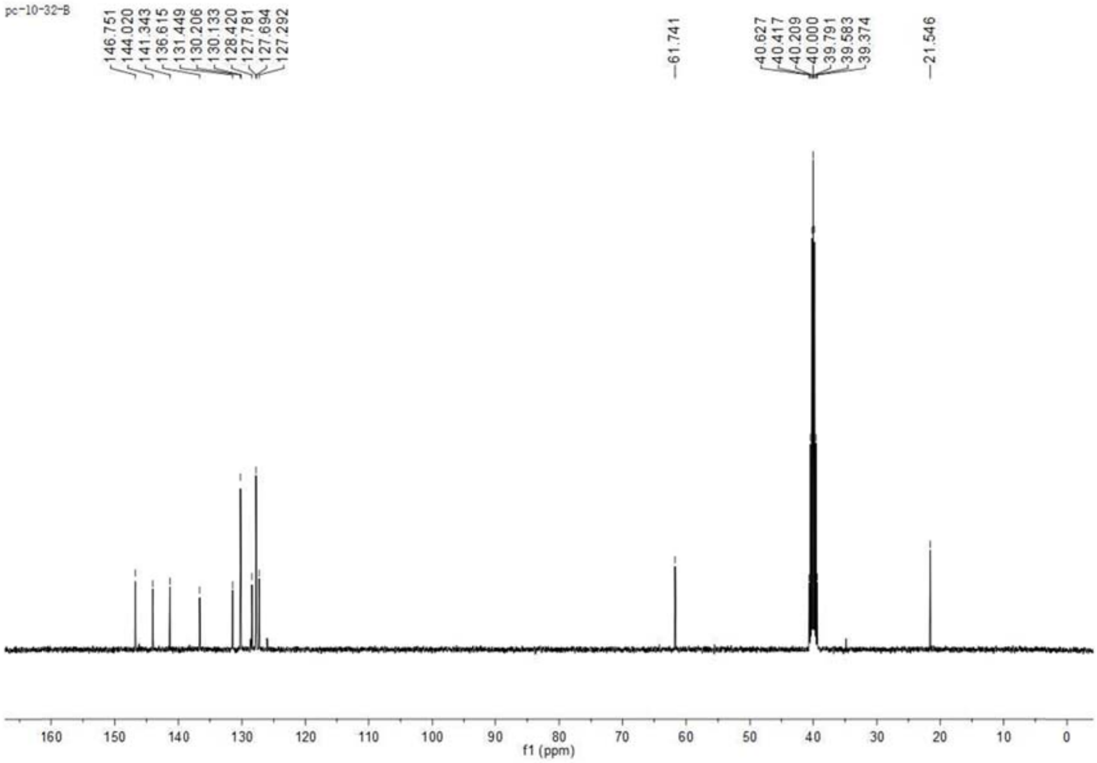

Supplementary Figure 14 ^{1}H NMR and ^{13}C NMR spectra for compound S2w

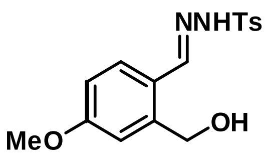

S2x

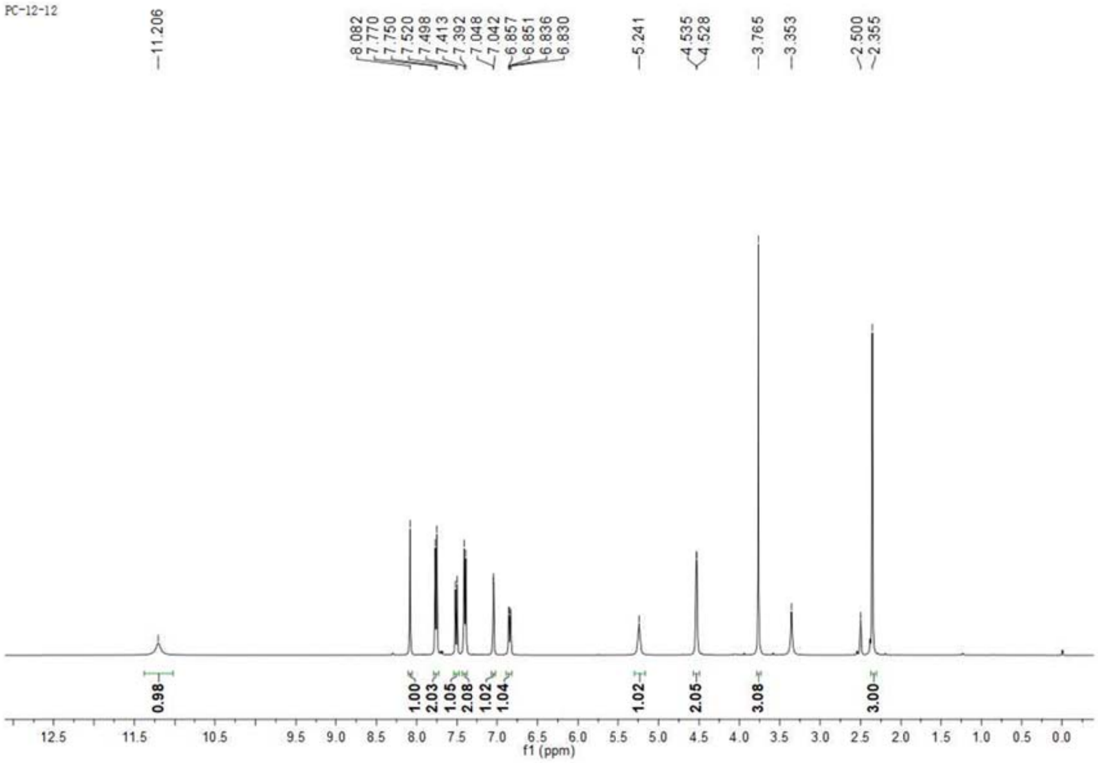

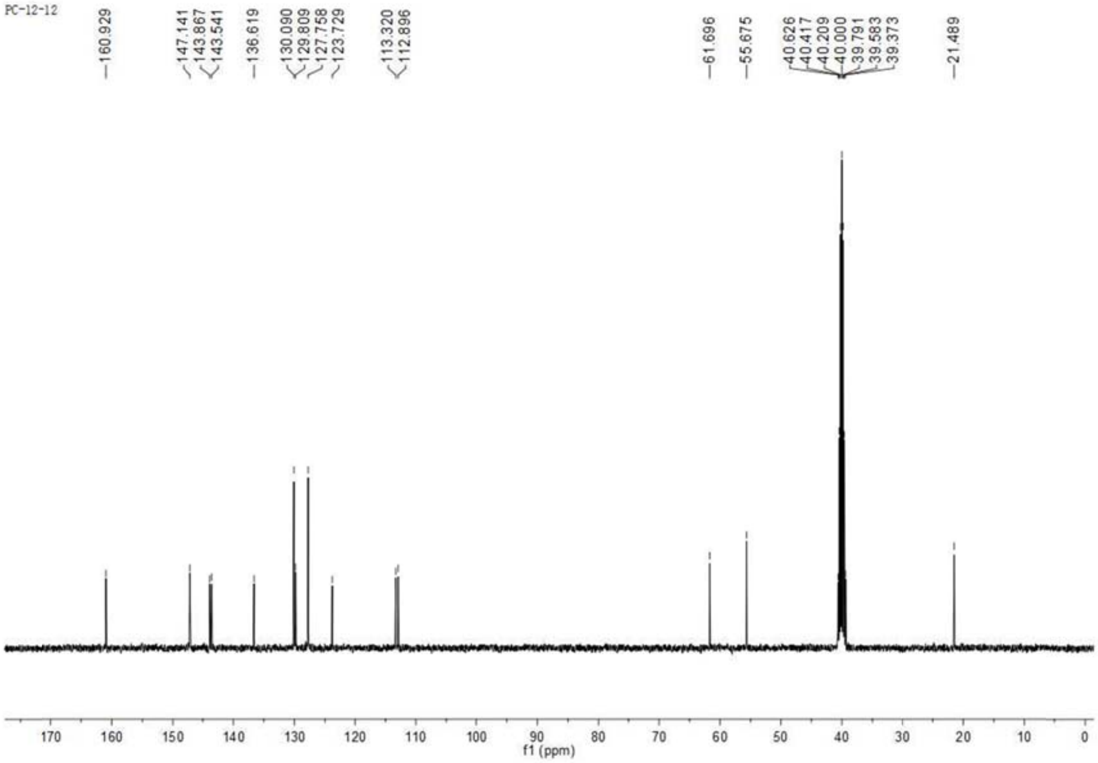

Supplementary Figure 15 ^{1}H NMR and ^{13}C NMR spectra for compound S2x

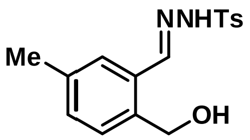

S2y

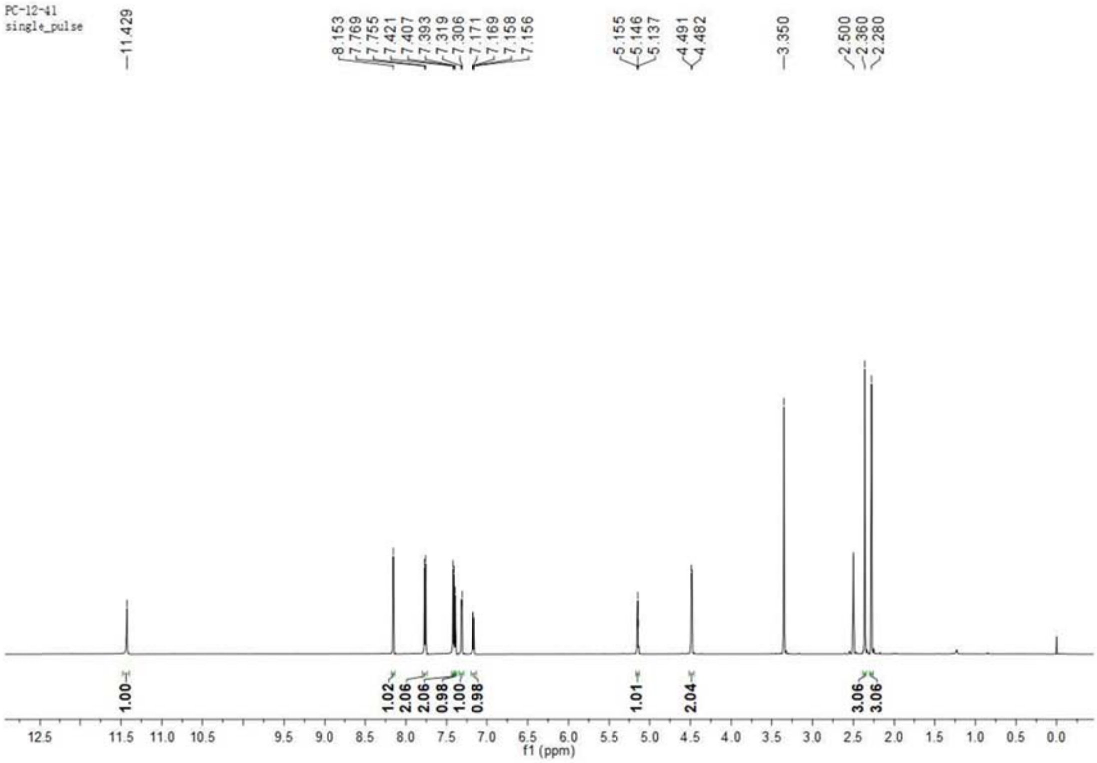

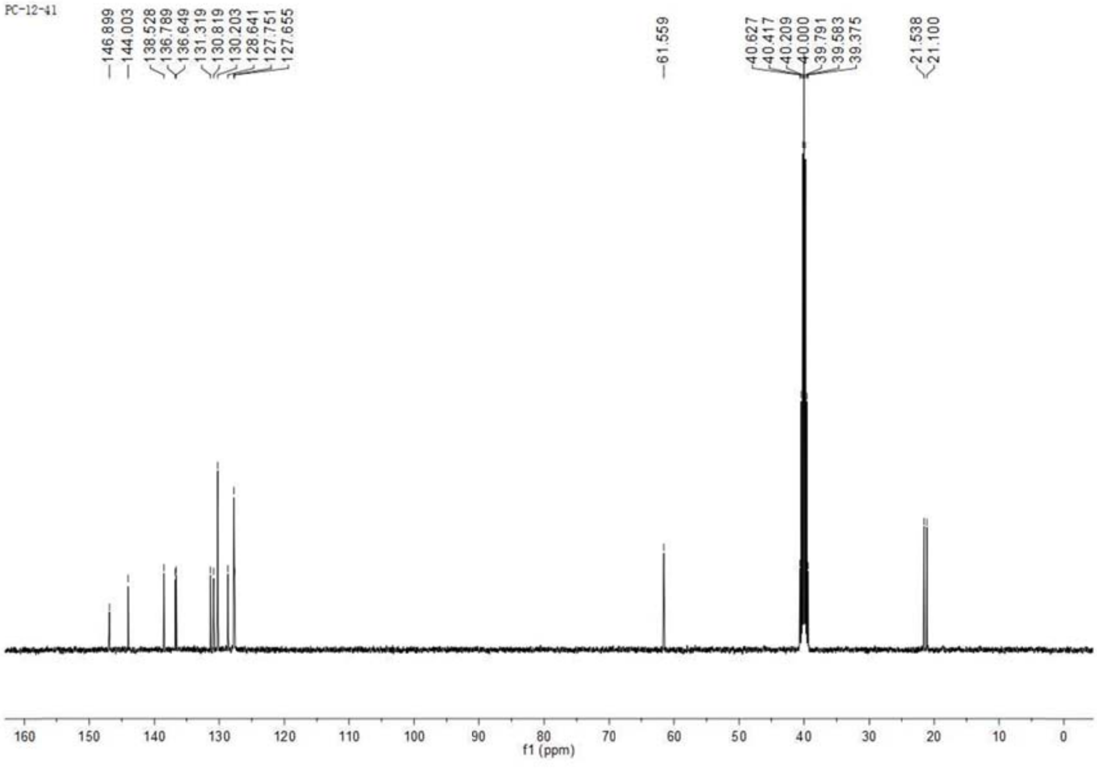

Supplementary Figure 16 ^{1}H NMR and ^{13}C NMR spectra for compound S2y

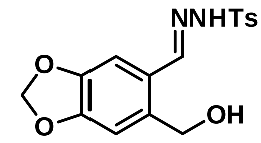

S2z

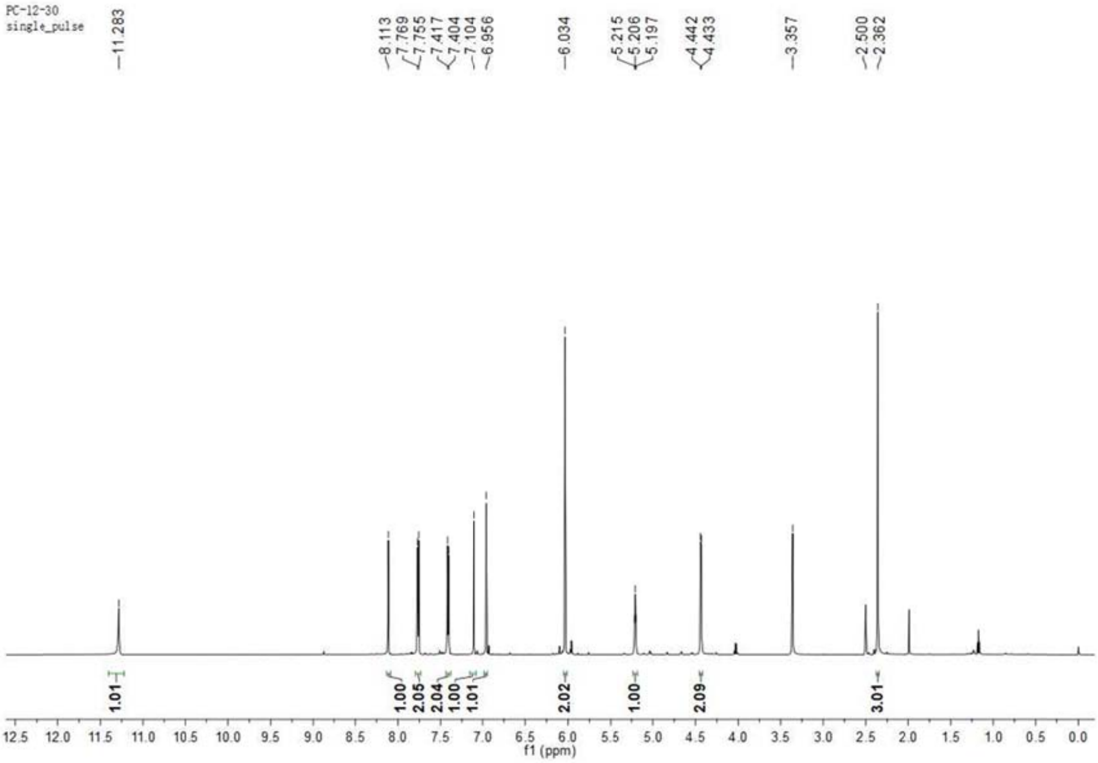

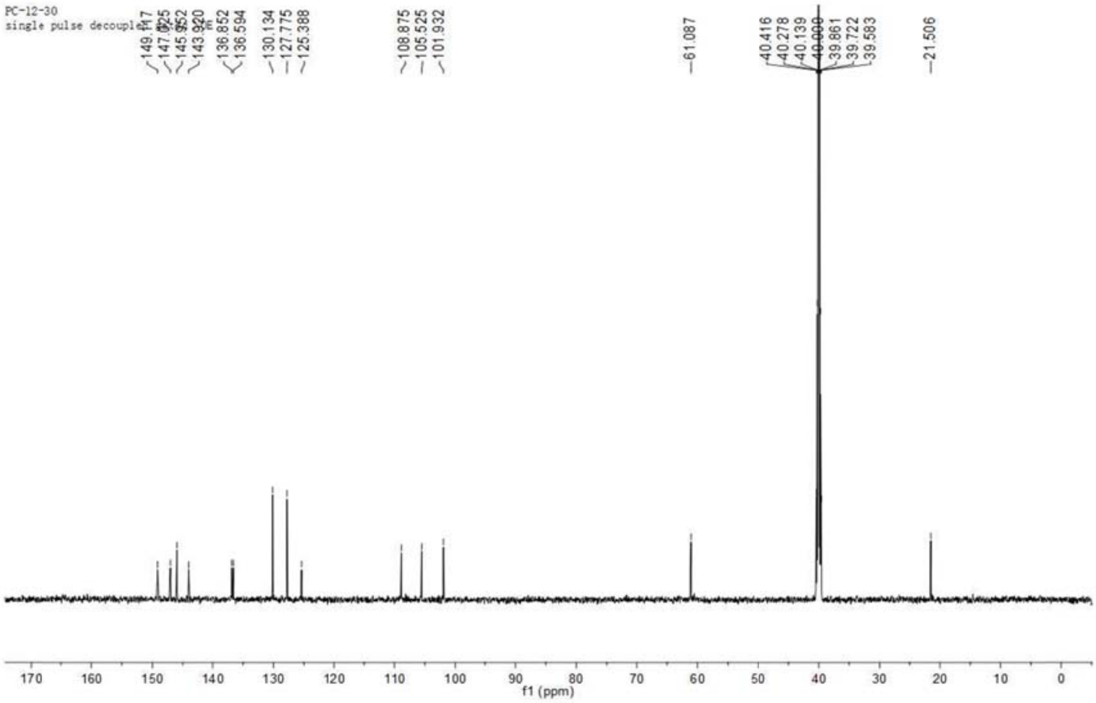

Supplementary Figure 17 ^{1}H NMR and ^{13}C NMR spectra for compound S2z

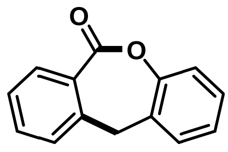

3

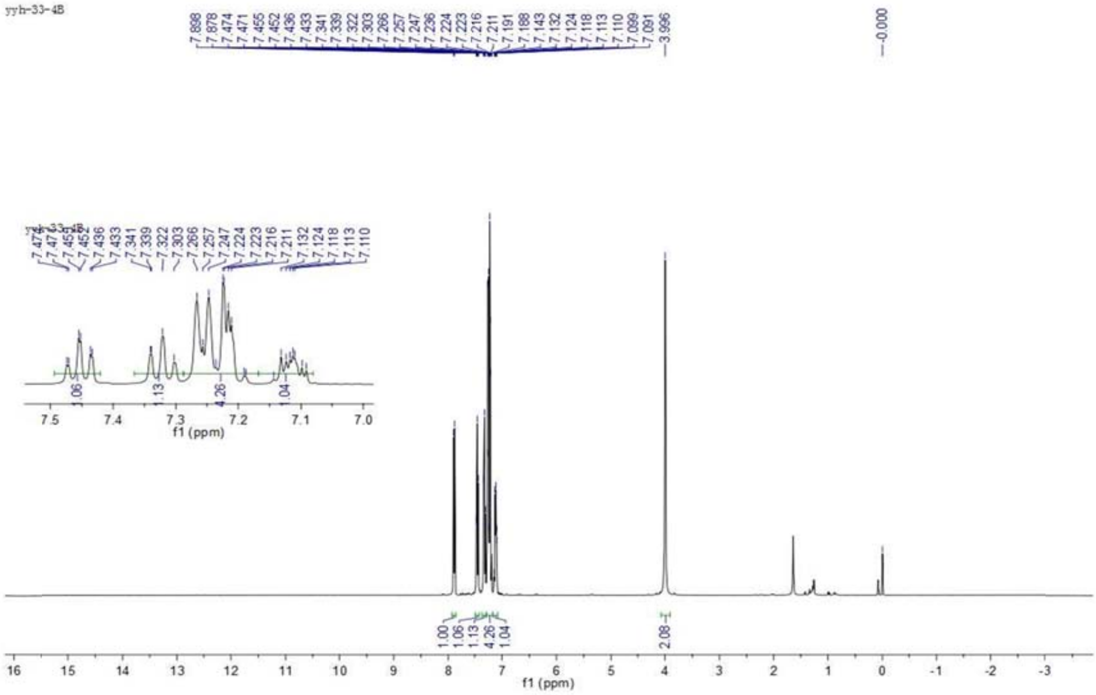

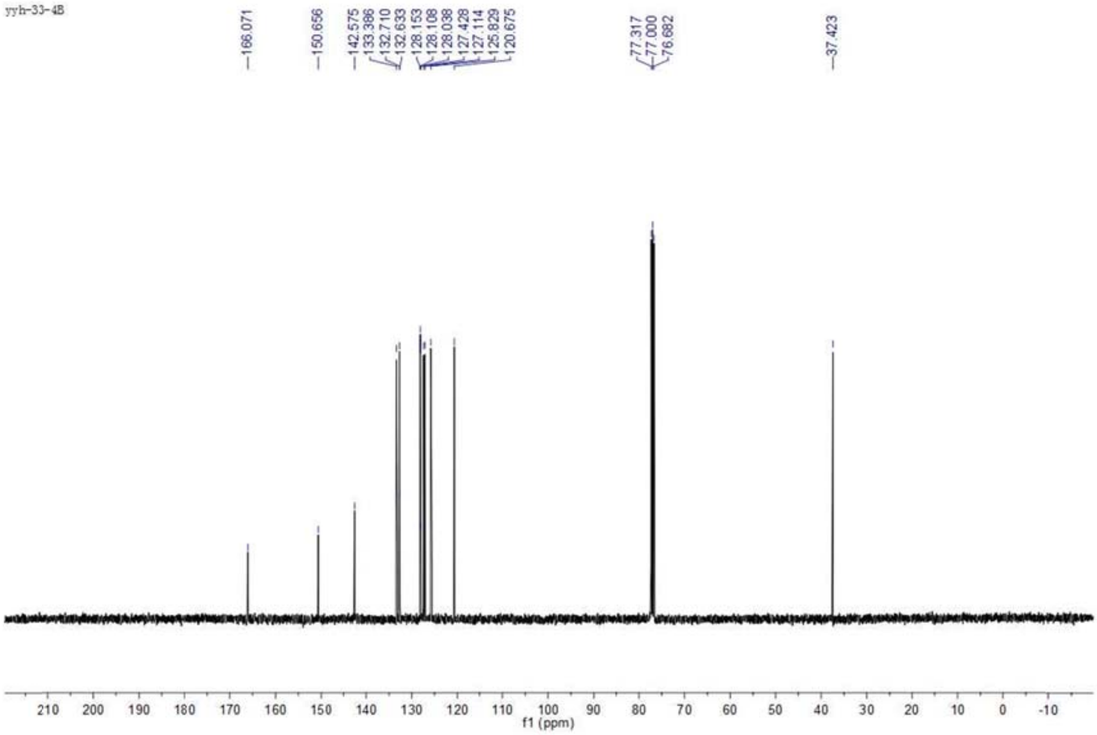

Supplementary Figure 18 ^{1}H NMR and ^{13}C NMR spectra for compound 3

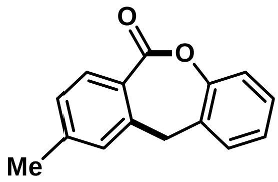

4

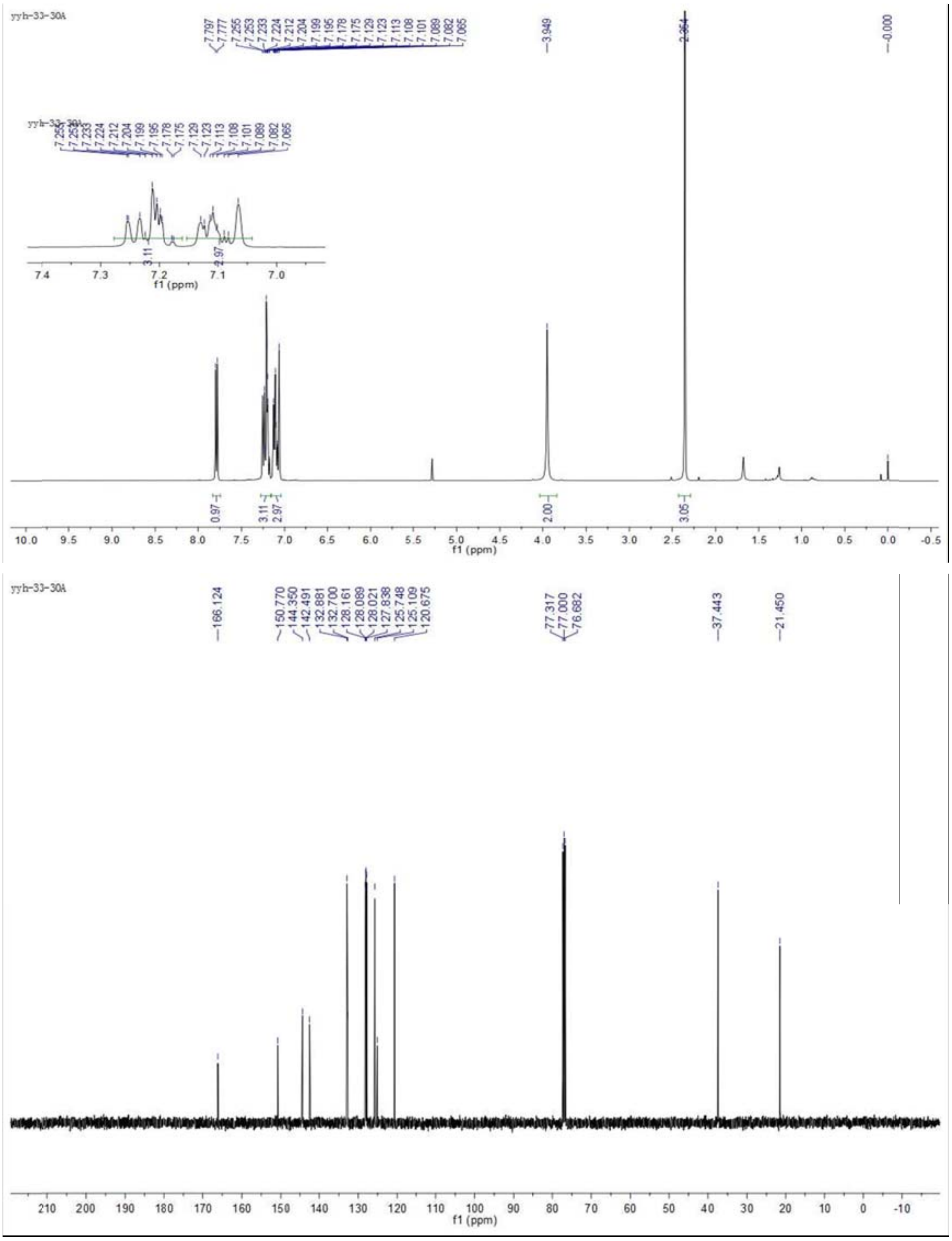

Supplementary Figure 19 ^{1}H NMR and ^{13}C NMR spectra for compound 4

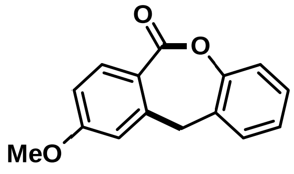

5

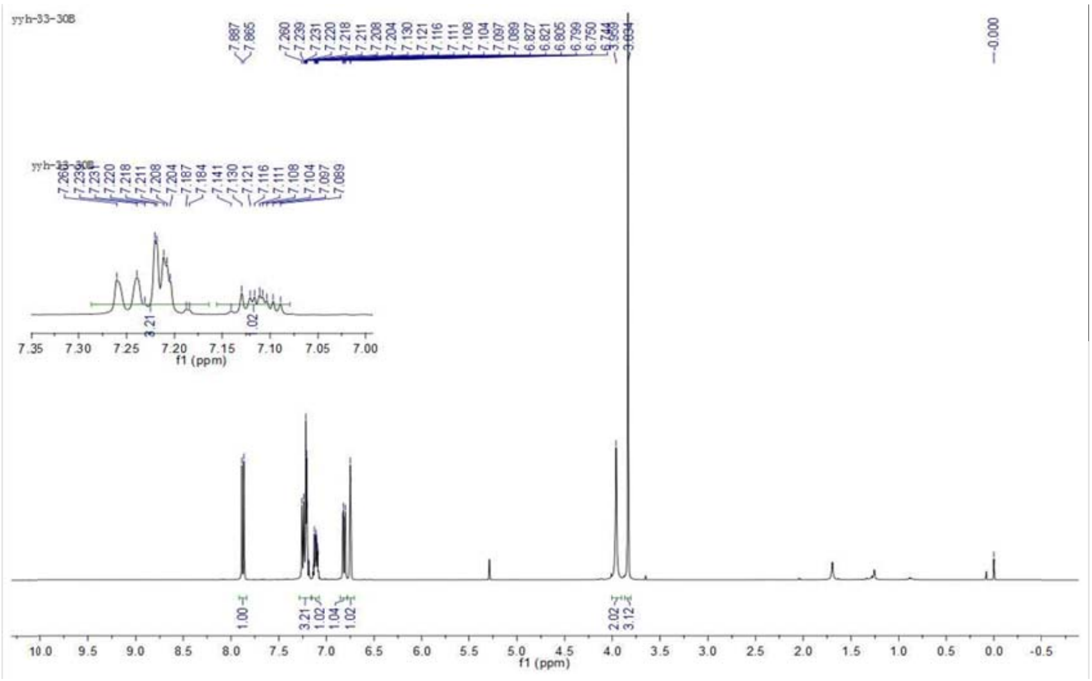

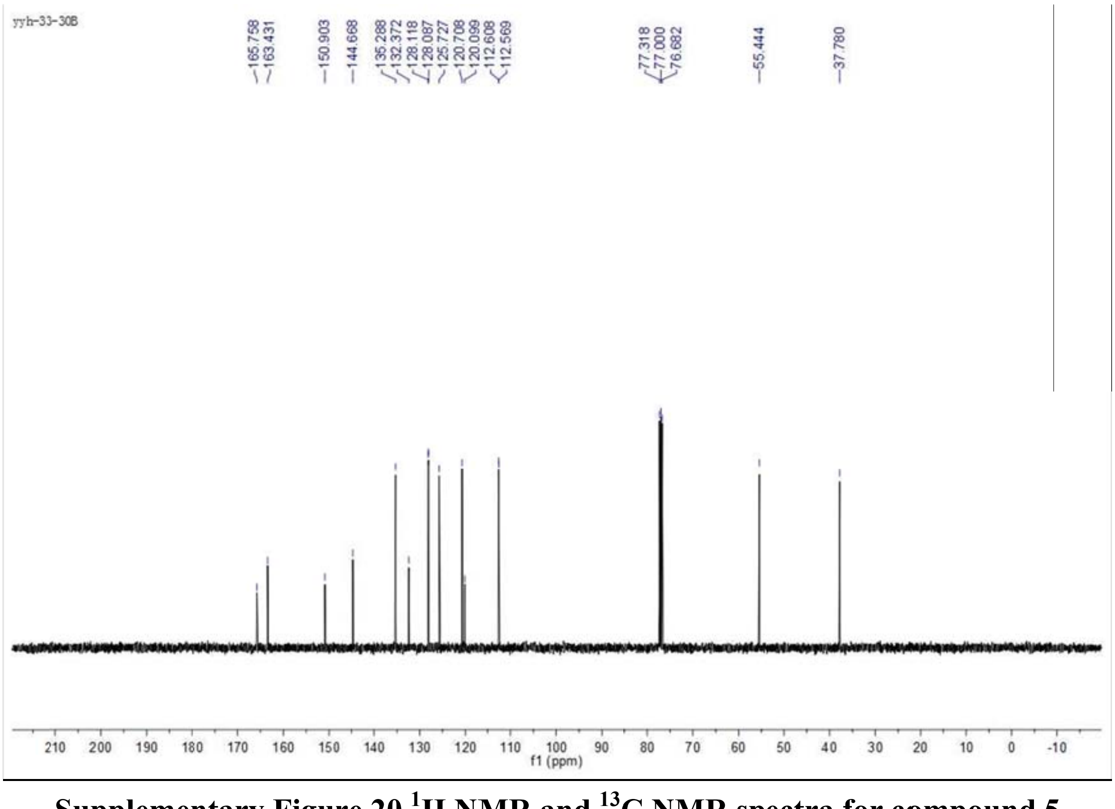

Supplementary Figure 20 ^{1}H NMR and ^{13}C NMR spectra for compound 5

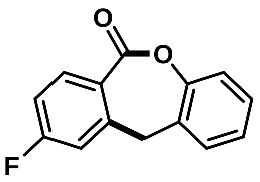

6

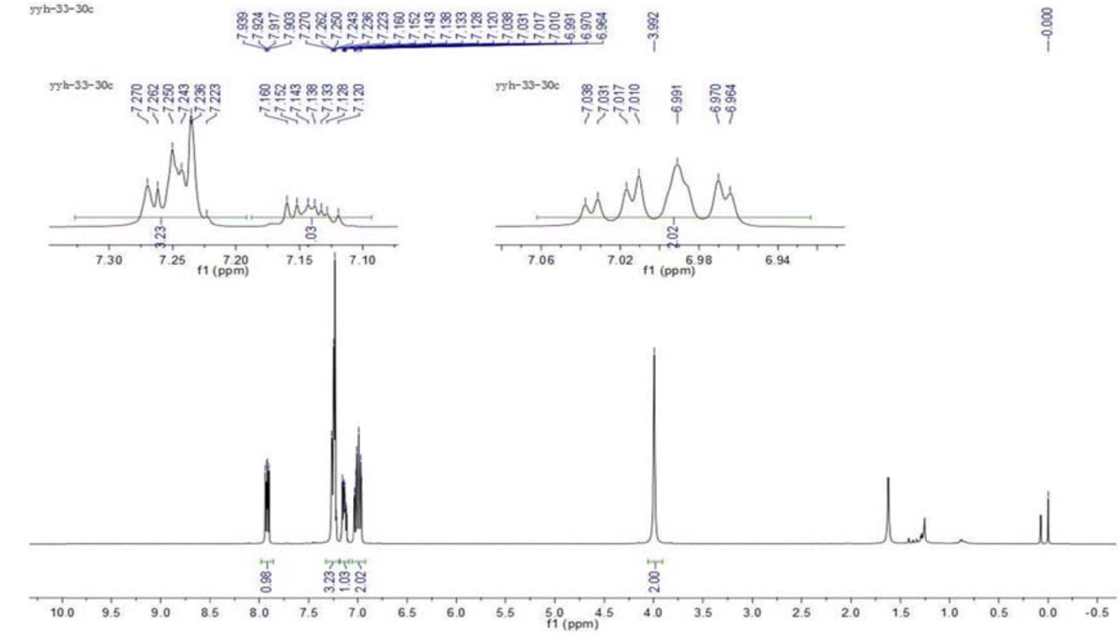

yyh-33-30c
7.939
7.924
7.917
7.903
7.270
7.262
7.250
7.243
7.236
7.223
7.160
7.152
7.143
7.138
7.133
7.128
7.120
7.038
7.031
7.017
7.010
6.991
6.970
6.964
3.992
0.000
3.23
03
7.30
7.25
7.20
7.15
7.10
f1 (ppm)
2.02
7.06
7.02
6.98
6.94
0.98
3.23
1.03
2.02
2.00
10.0
9.5
9.0
8.5
8.0
7.5
7.0
6.5
6.0
5.5
5.0
4.5
4.0
3.5
3.0
2.5
2.0
1.5
1.0
0.5
0.0
-0.5

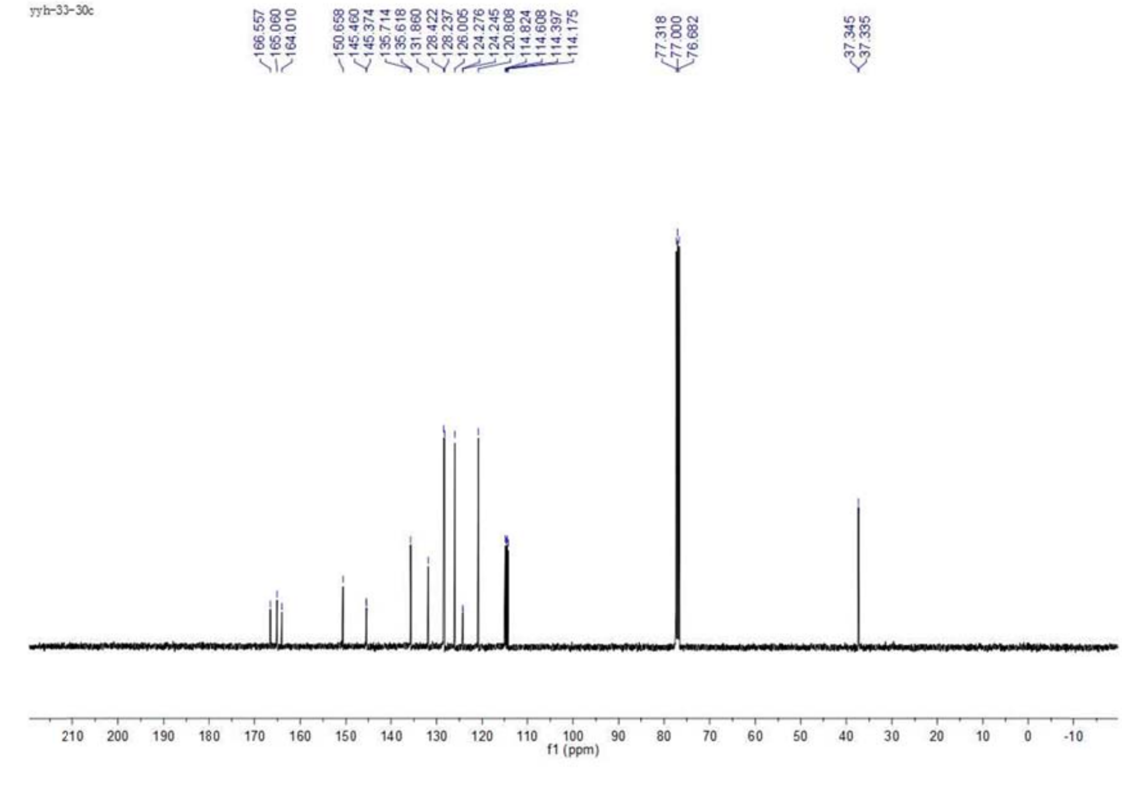

yyh-33-30c
166.557
165.060
164.010
150.658
145.460
145.374
135.714
135.618
131.860
128.422
128.237
126.005
124.276
124.245
120.808
114.824
114.608
114.397
114.175
77.318
77.000
76.682
37.345
37.335
210
200
190
180
170
160
150
140
130
120
110
100
90
80
70
60
50
40
30
20
10
0
-10
f1 (ppm)

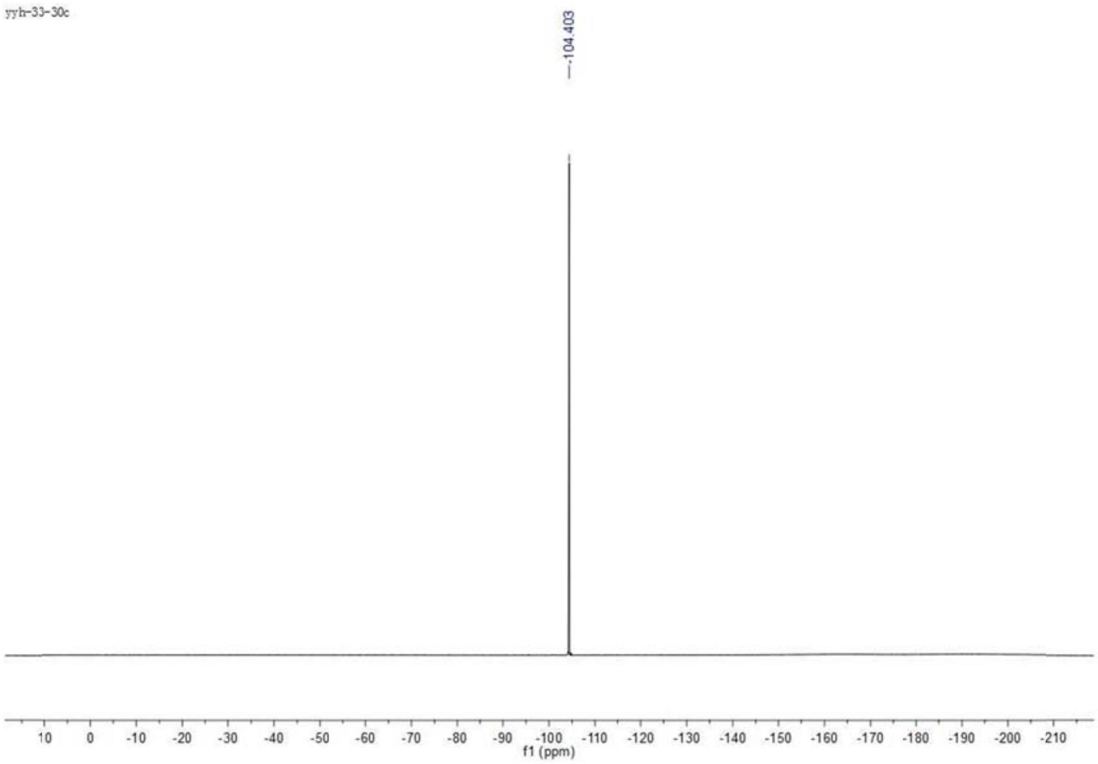

Supplementary Figure 21 ^{1}H NMR, ^{13}C NMR and ^{19}F NMR spectra for compound 6

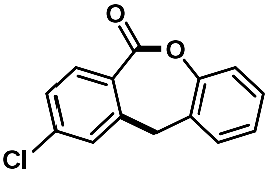

7

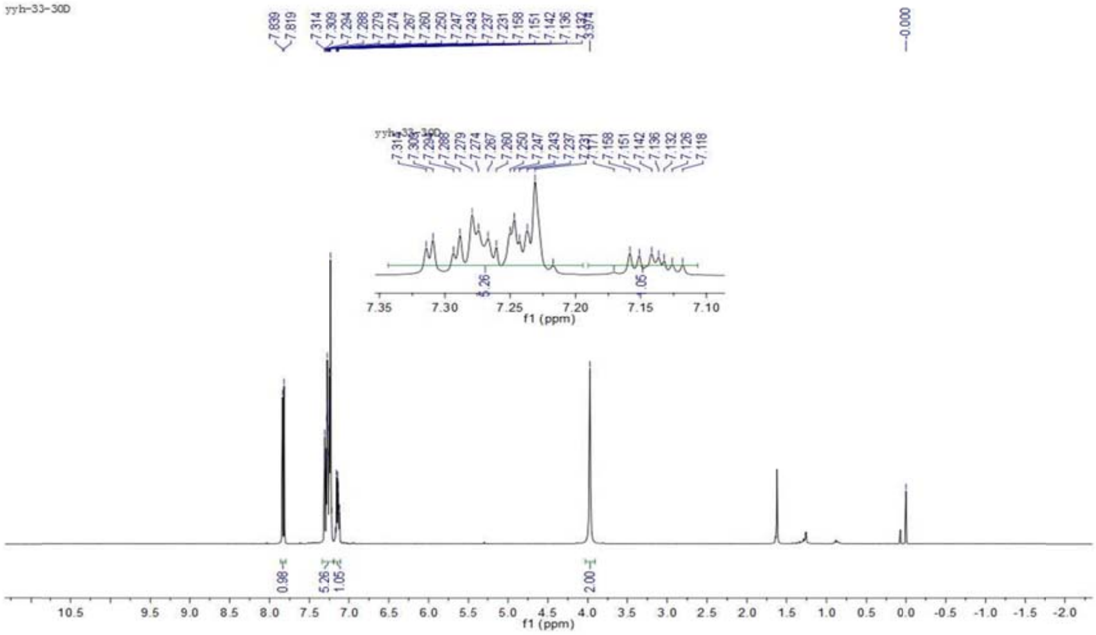

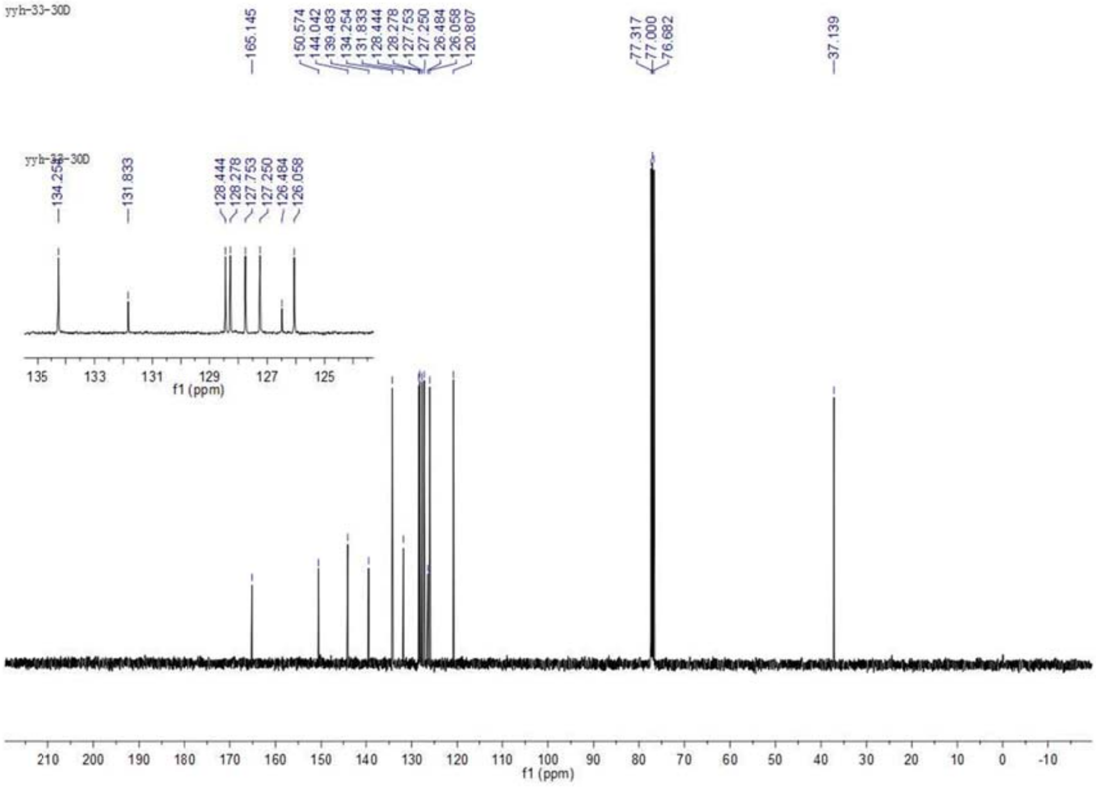

Supplementary Figure 22 ^{1}H NMR and ^{13}C NMR spectra for compound 7

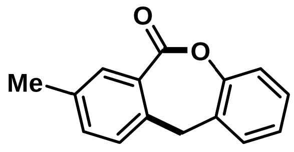

8

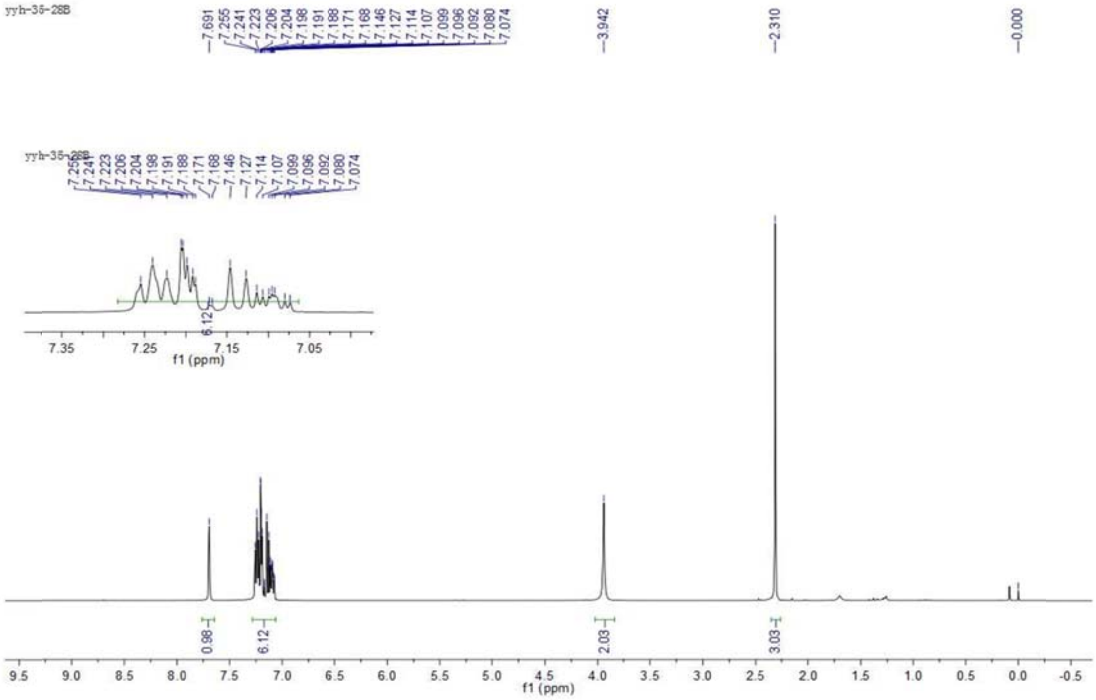

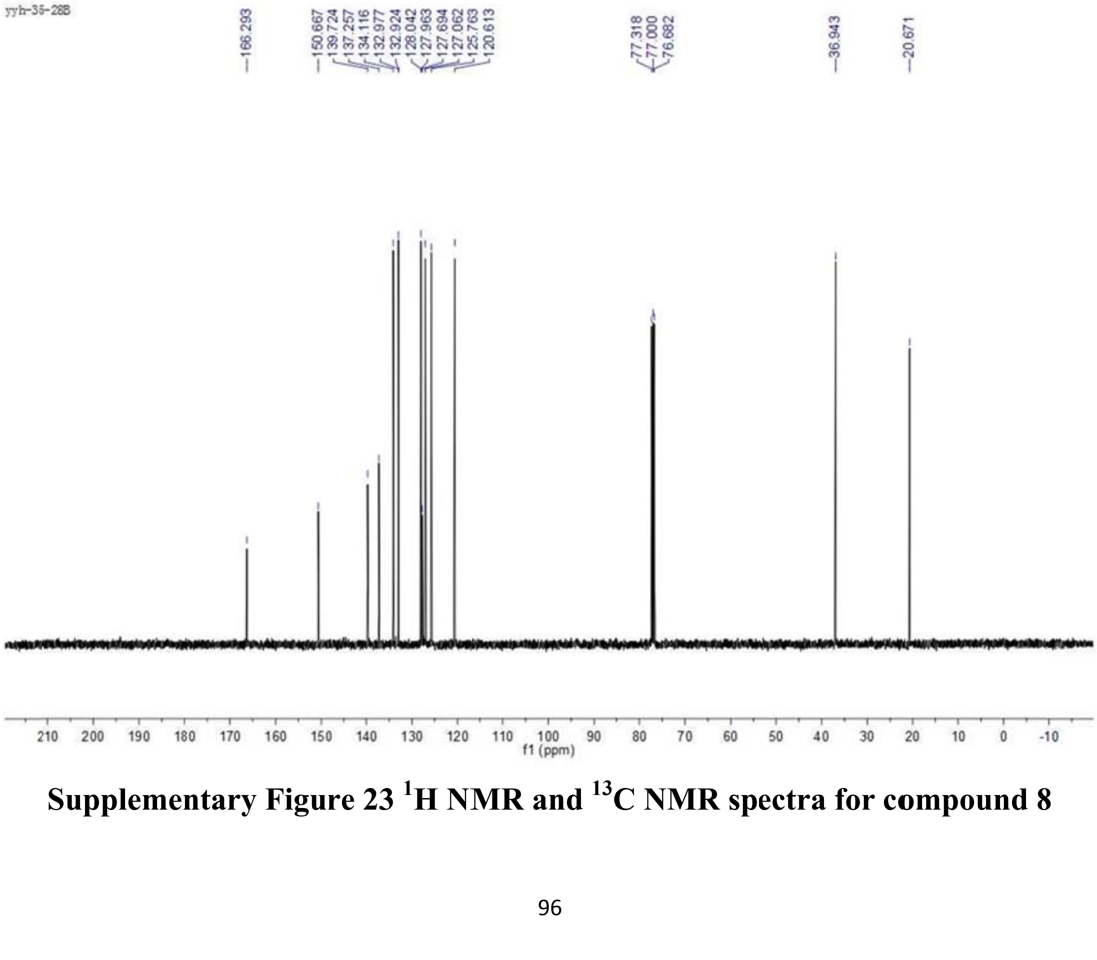

Supplementary Figure 23 ^{1}H NMR and ^{13}C NMR spectra for compound 8

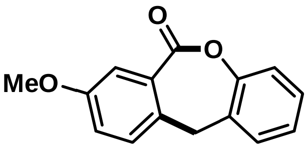

9

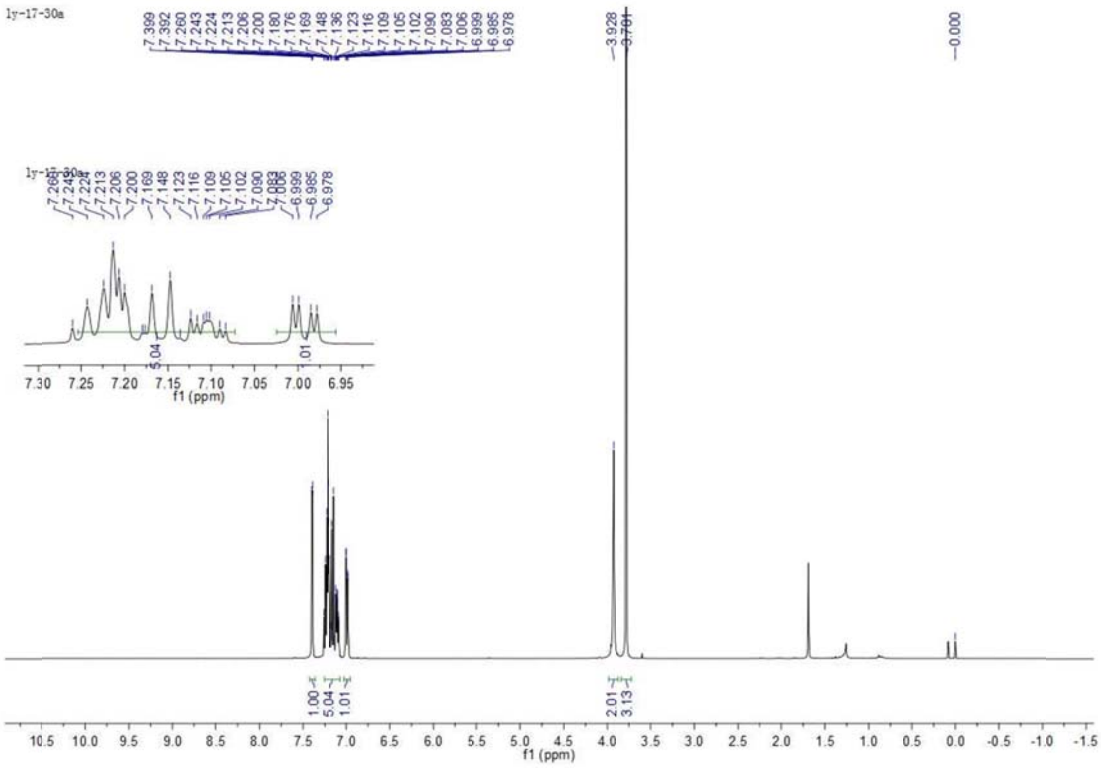

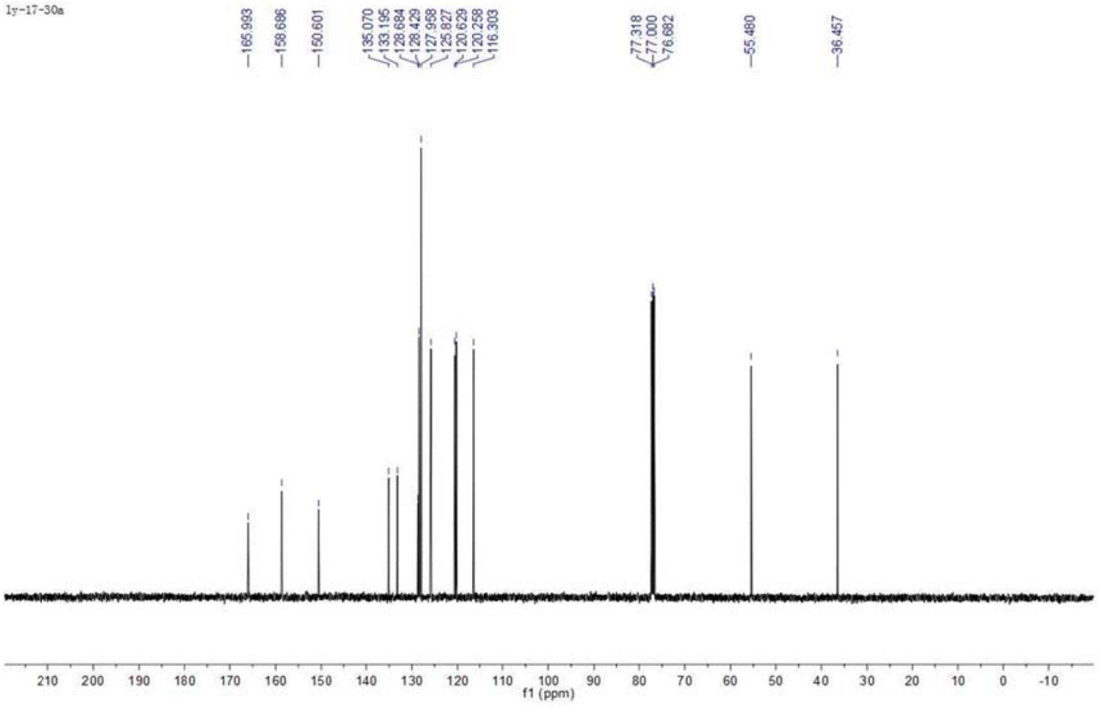

Supplementary Figure 24 ^{1}H NMR and ^{13}C NMR spectra for compound 9

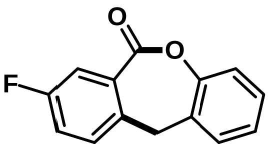

10

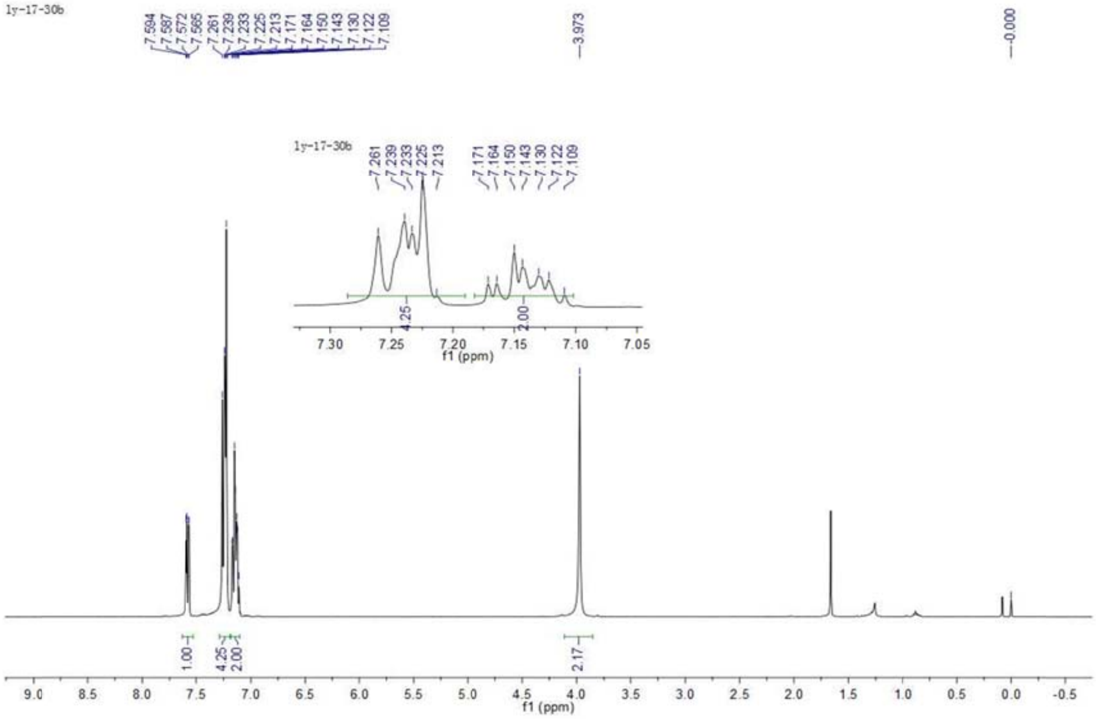

ly-17-30b
7.594
7.587
7.572
7.565
7.261
7.239
7.233
7.225
7.213
7.171
7.164
7.150
7.143
7.130
7.122
7.109
3.973
0.000
4.25
2.00
7.30
7.25
7.20
7.15
7.10
7.05
f1 (ppm)
1.00
2.17
9.0
8.5
8.0
7.5
7.0
6.5
6.0
5.5
5.0
4.5
4.0
3.5
3.0
2.5
2.0
1.5
1.0
0.5
0.0
-0.5

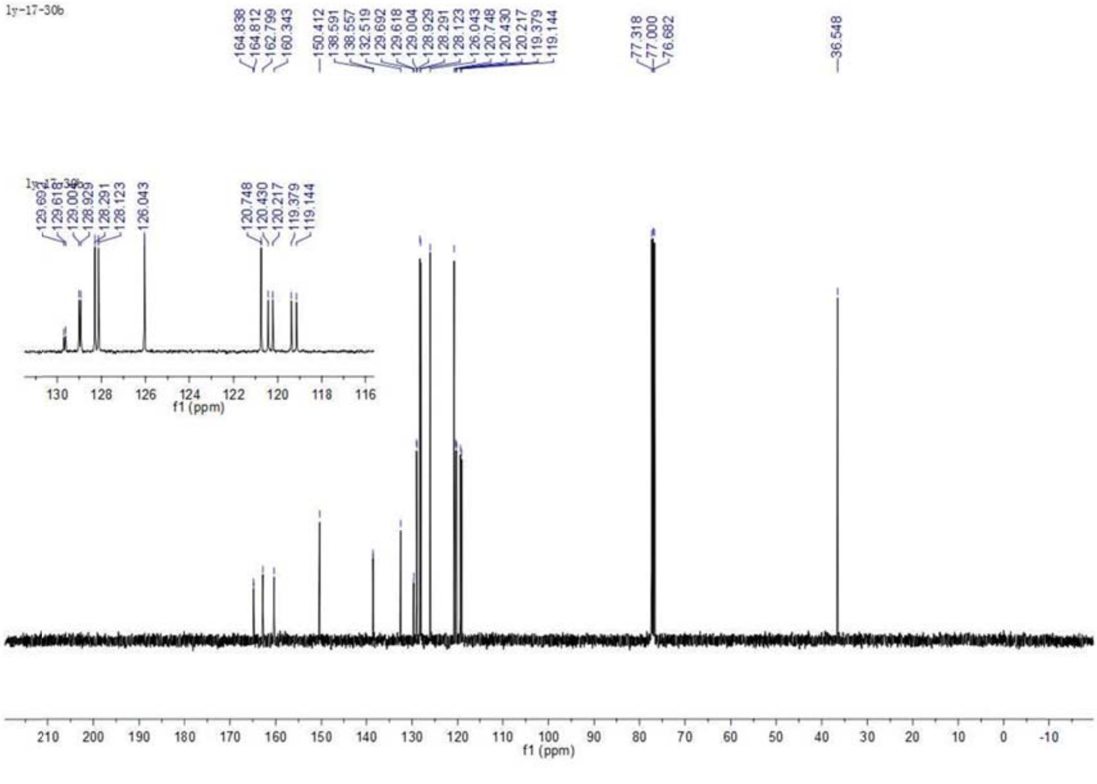

ly-17-30b
164.838
164.812
162.799
160.343
150.412
138.591
138.557
132.519
129.692
129.618
129.004
128.929
128.291
128.123
126.043
120.748
120.430
120.217
119.379
119.144
77.318
77.000
76.682
36.548
130
128
126
124
122
120
118
116
f1 (ppm)
210
200
190
180
170
160
150
140
130
120
110
100
90
80
70
60
50
40
30
20
10
0
-10

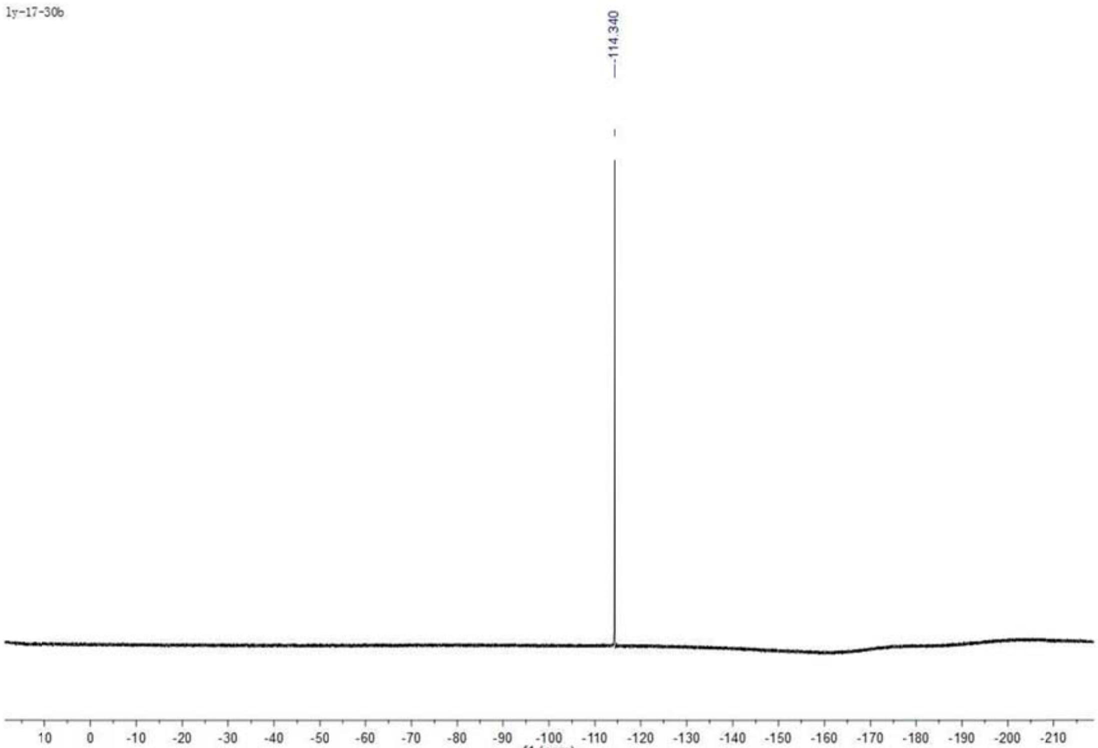

Supplementary Figure 25 ^{1}H NMR, ^{13}C NMR and ^{19}F NMR spectra for compound 10

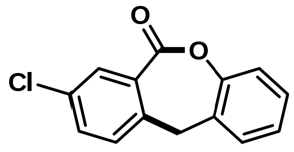

11

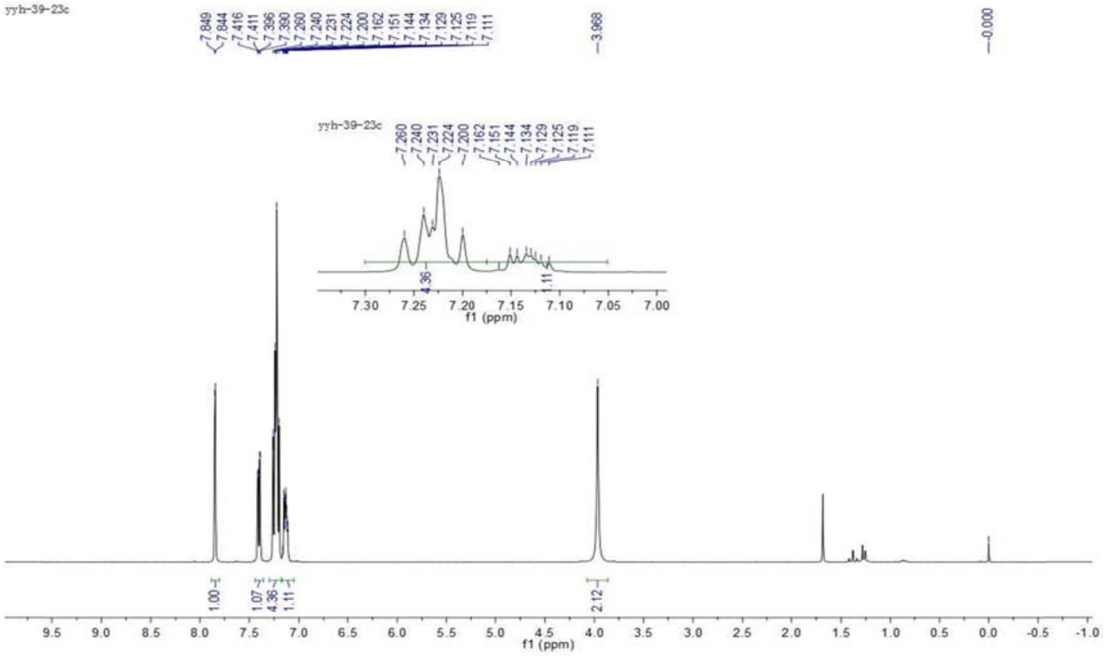

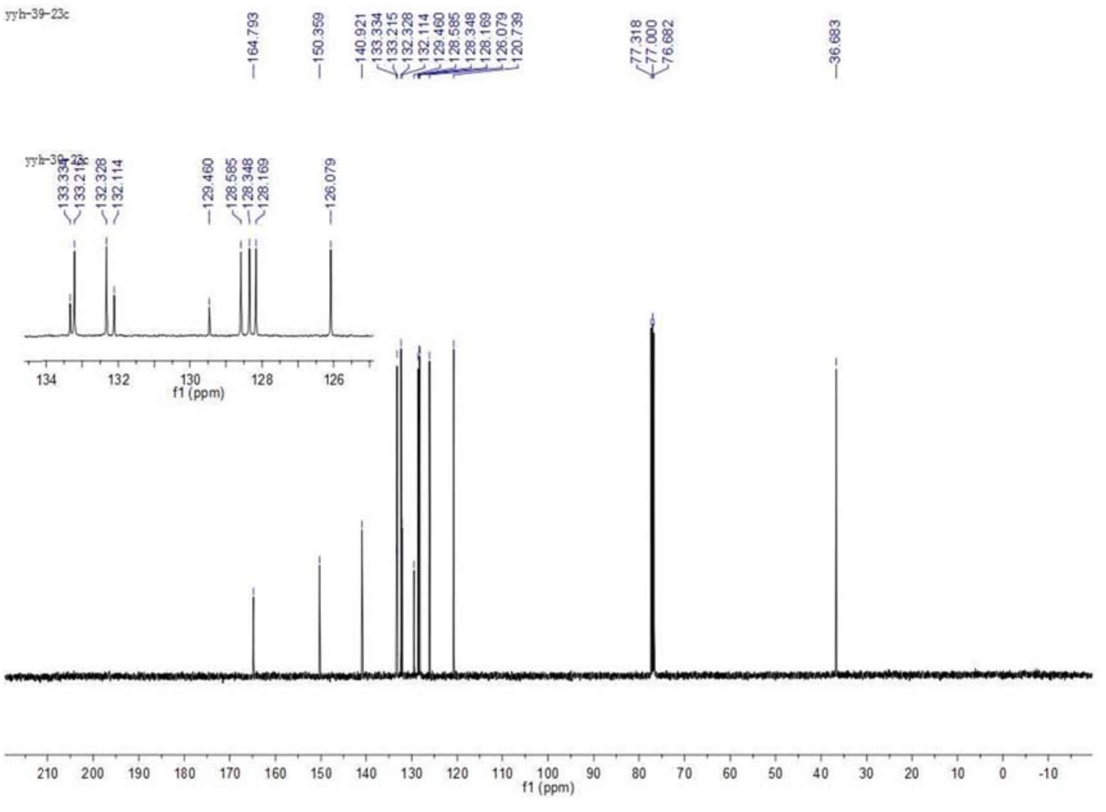

Supplementary Figure 26 ^{1}H NMR and ^{13}C NMR spectra for compound 11

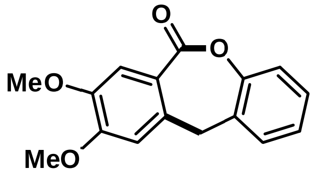

12

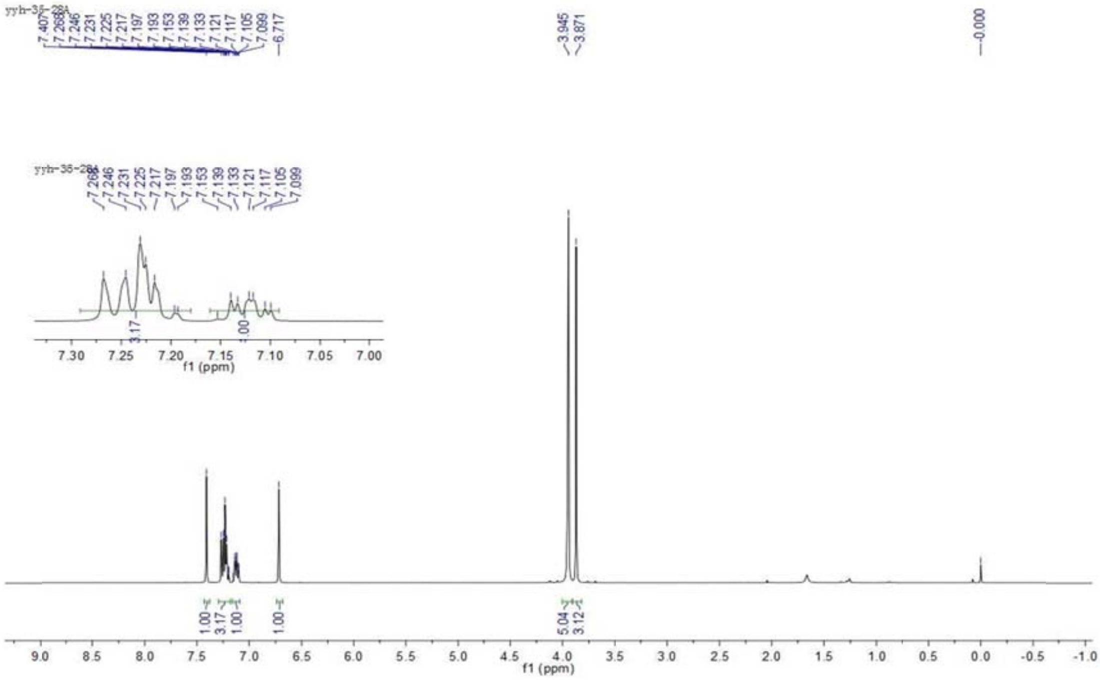

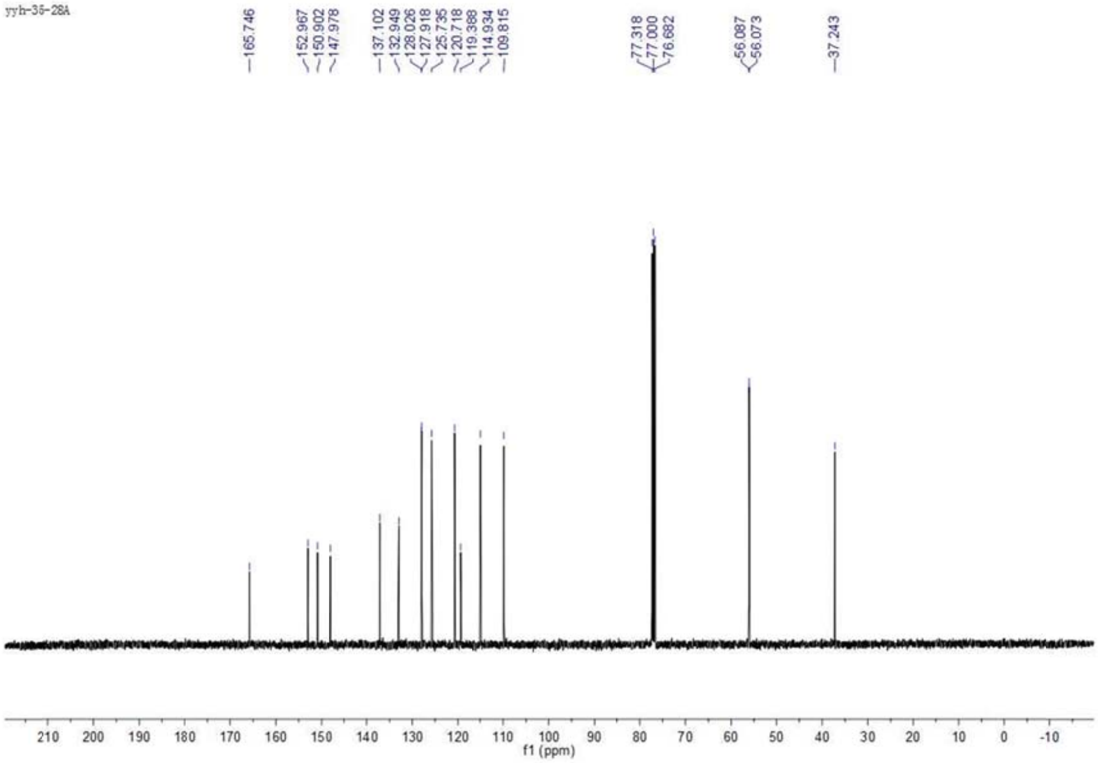

Supplementary Figure 27 ^{1}H NMR and ^{13}C NMR spectra for compound 12

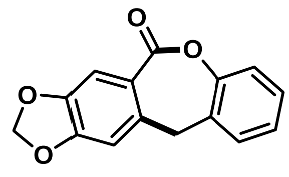

13

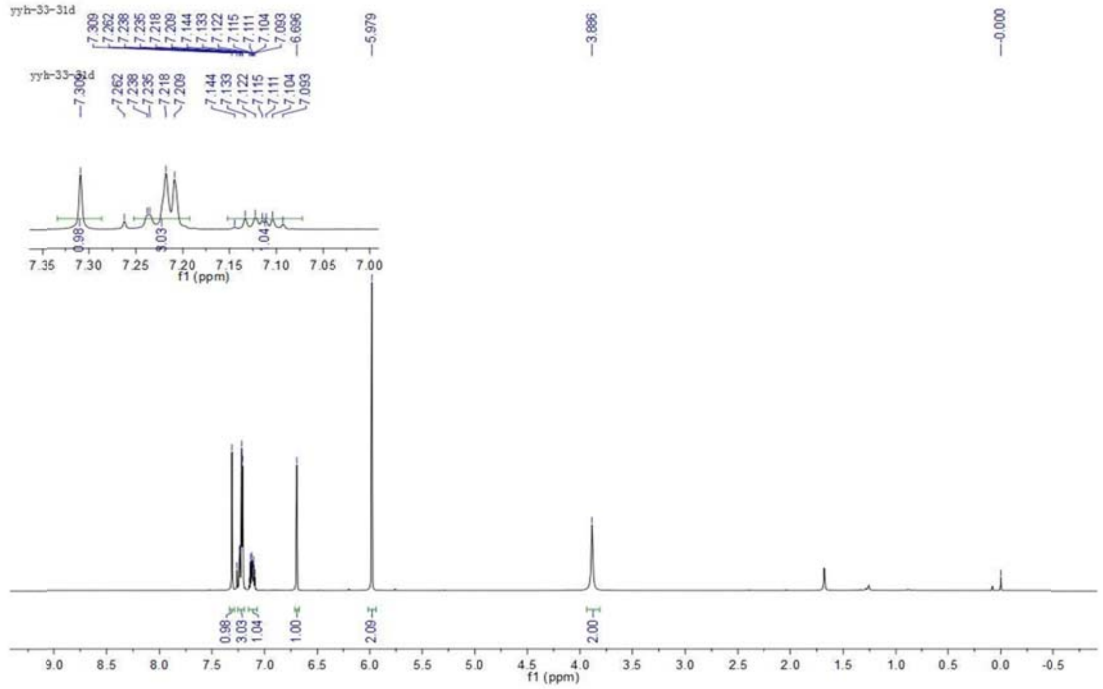

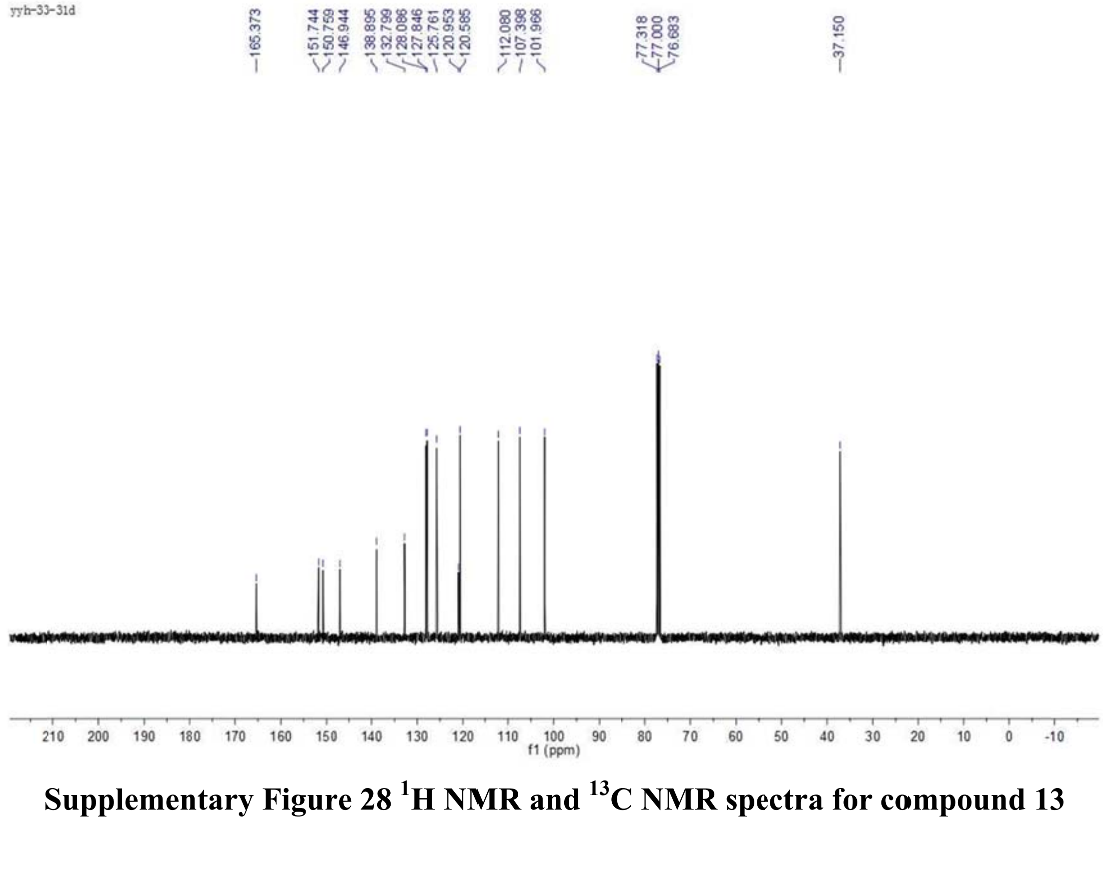

Supplementary Figure 28 ^{1}H NMR and ^{13}C NMR spectra for compound 13

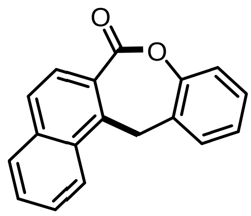

14

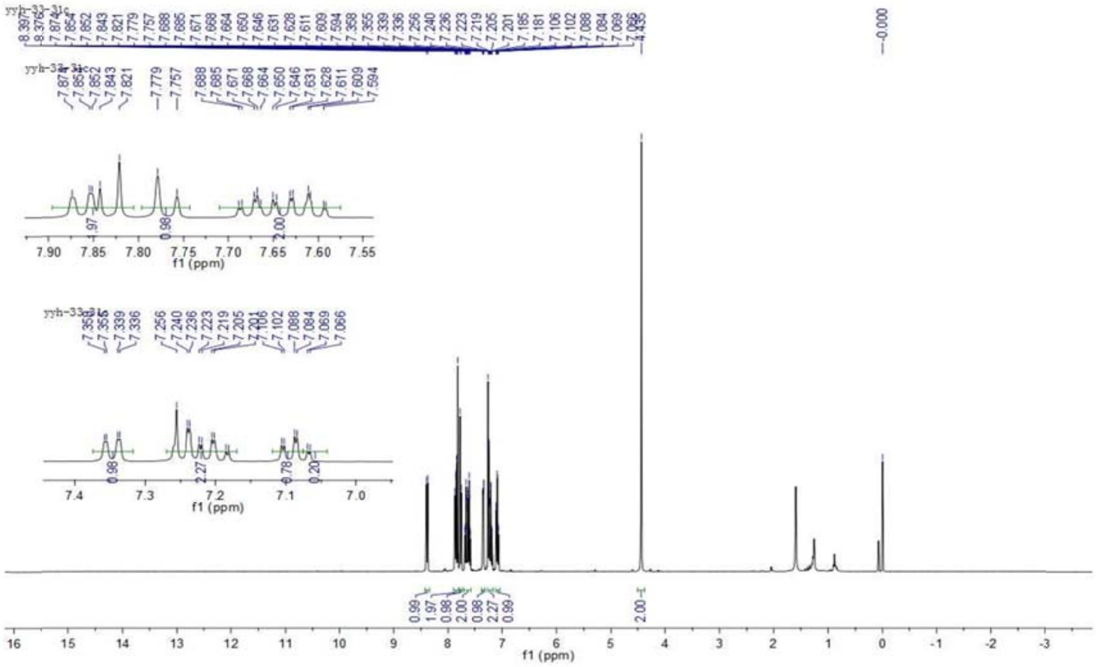

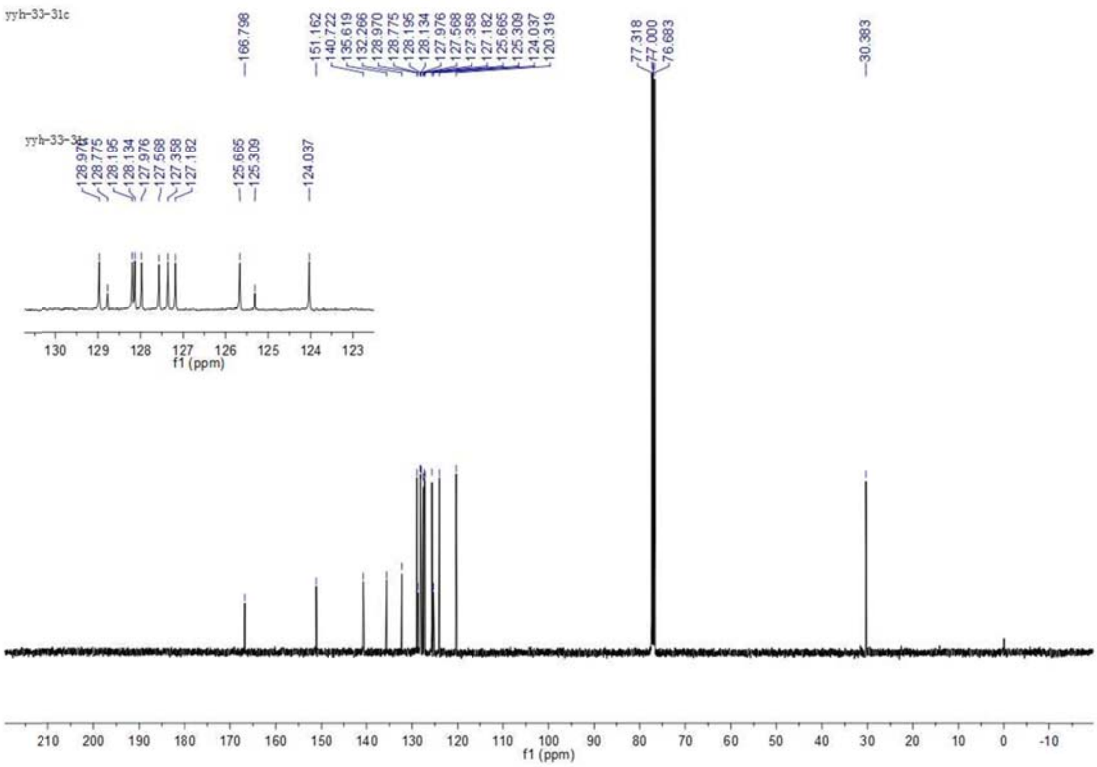

Supplementary Figure 29 ^{1}H NMR and ^{13}C NMR spectra for compound 14

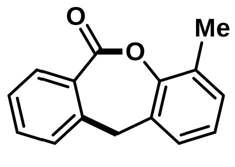

15

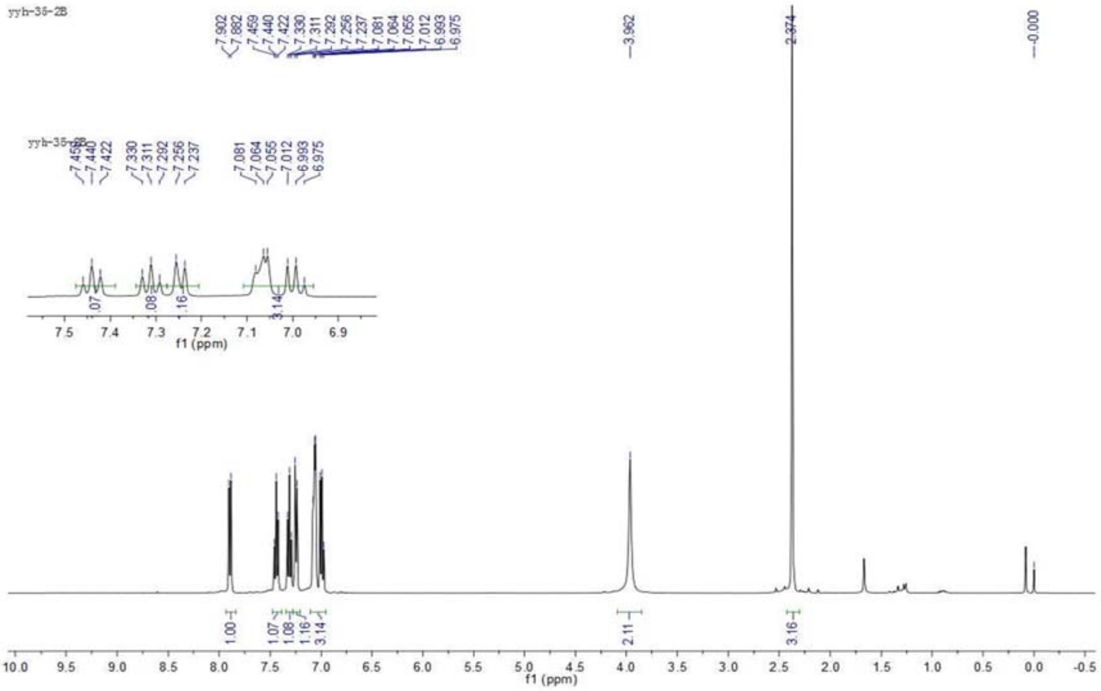

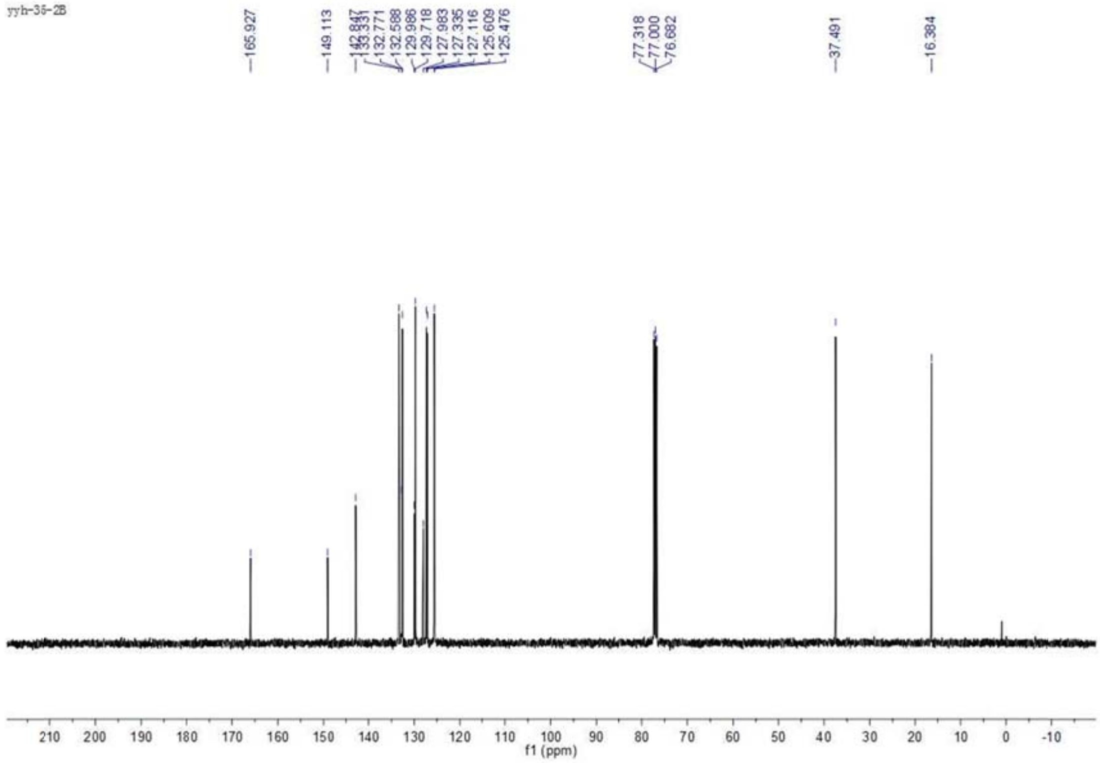

Supplementary Figure 30 ^{1}H NMR and ^{13}C NMR spectra for compound 15

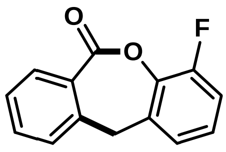
O
O
F

16

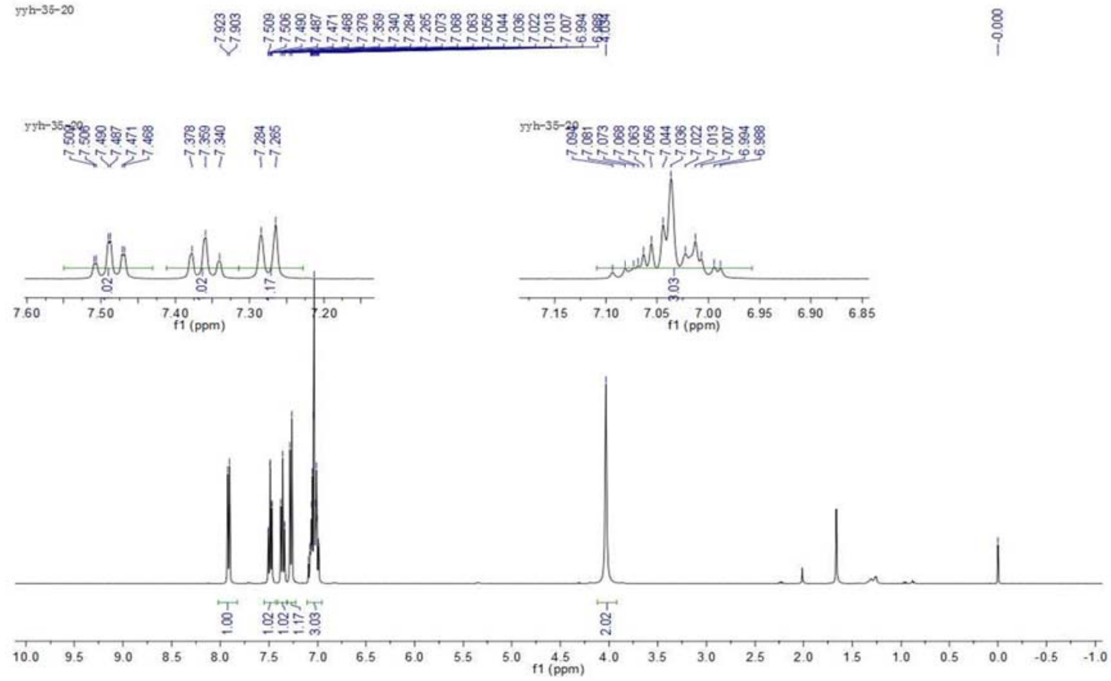
yyh-35-20
7.923
7.903
-0.000
f1 (ppm)

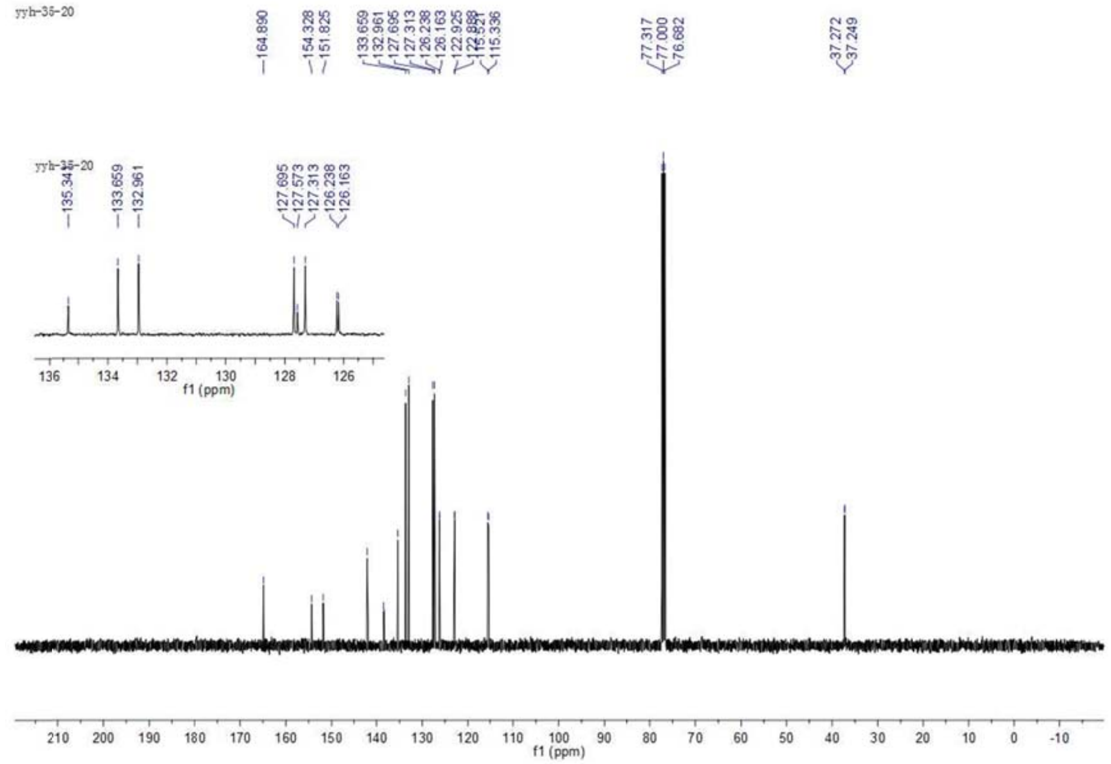
yyh-35-20
164.890
154.328
151.825
77.317
77.000
76.682
37.272
37.249
f1 (ppm)

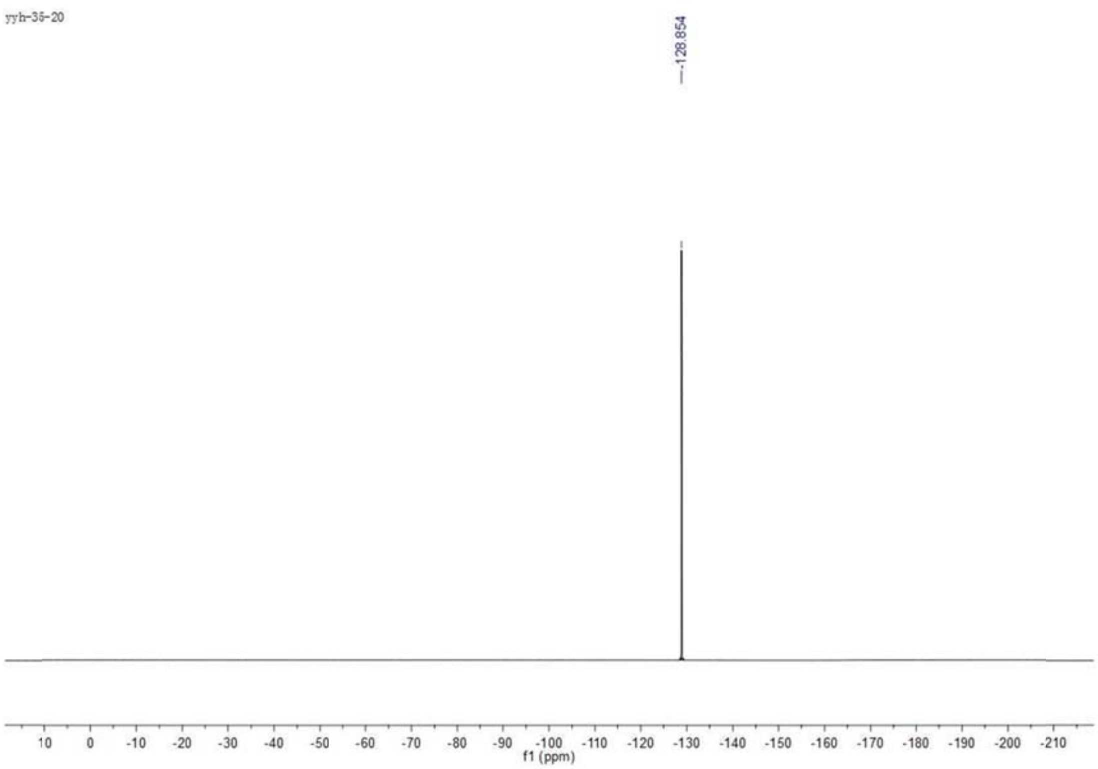

Supplementary Figure 31 ^{1}H NMR, ^{13}C NMR and ^{19}F NMR spectra for compound 16

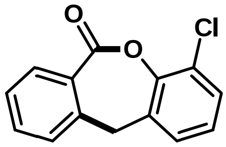

17

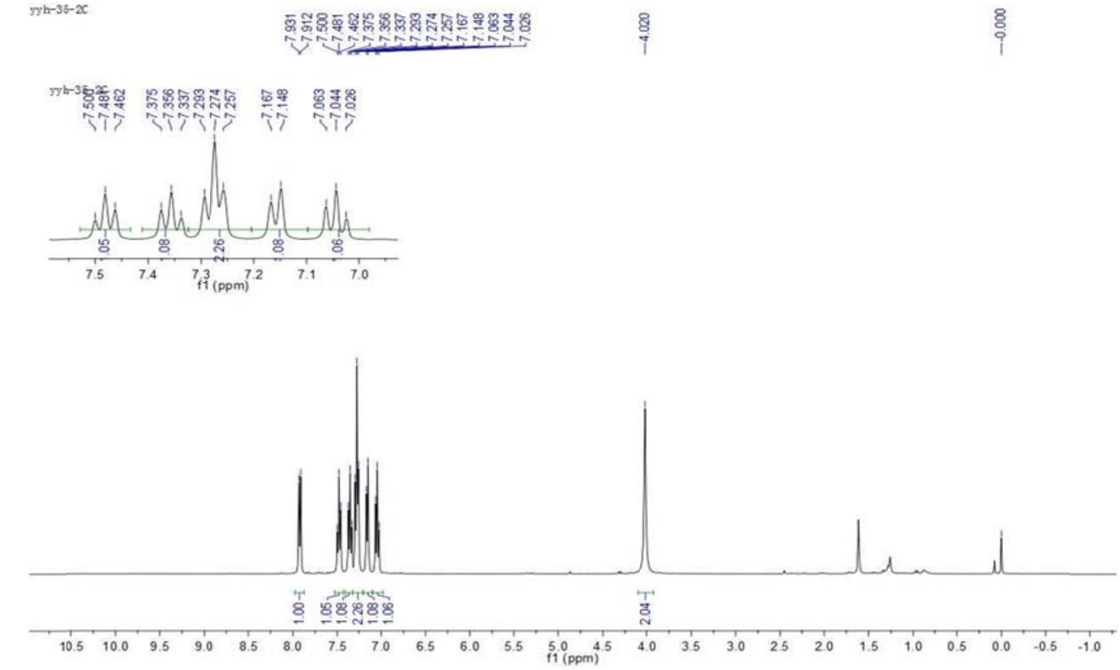

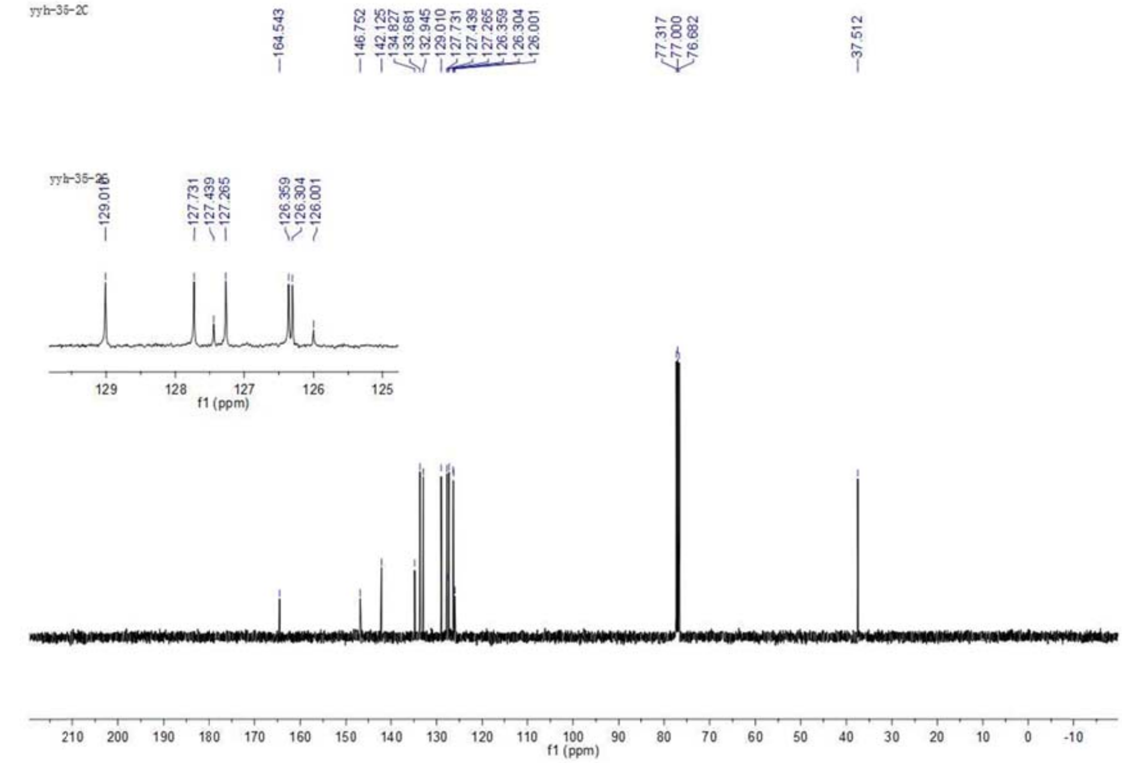

Supplementary Figure 32 ^{1}H NMR and ^{13}C NMR spectra for compound 17

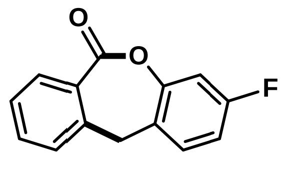
O
O
F

18

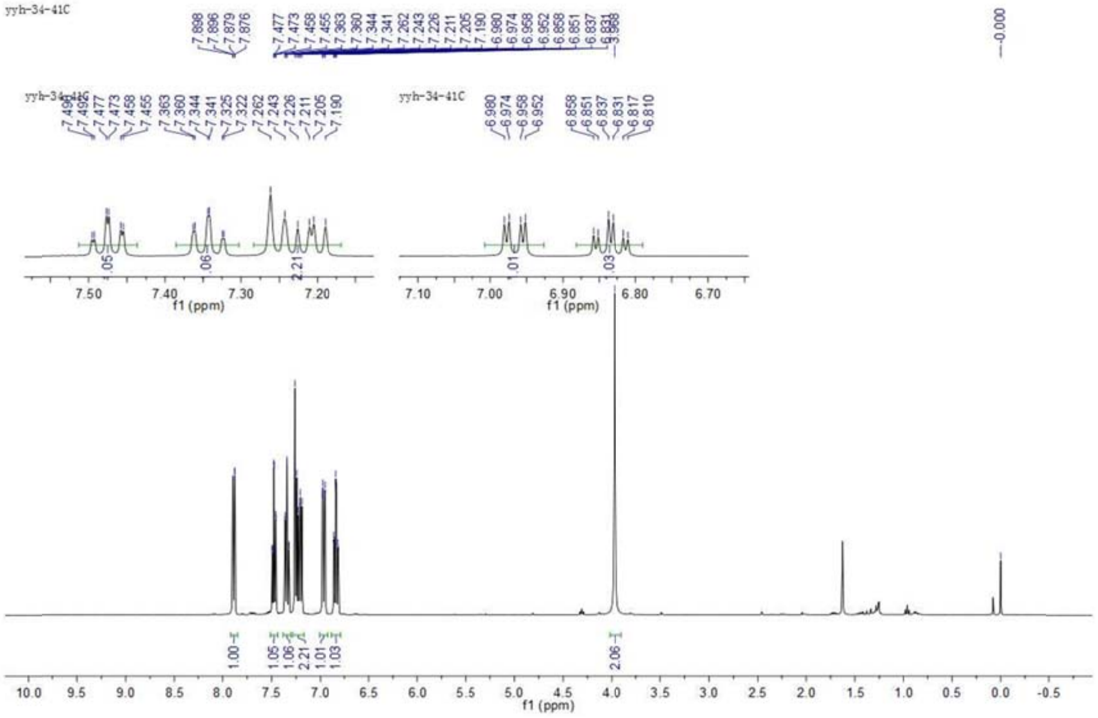
yyh-34-41C
7.898
7.896
7.879
7.876
f1 (ppm)

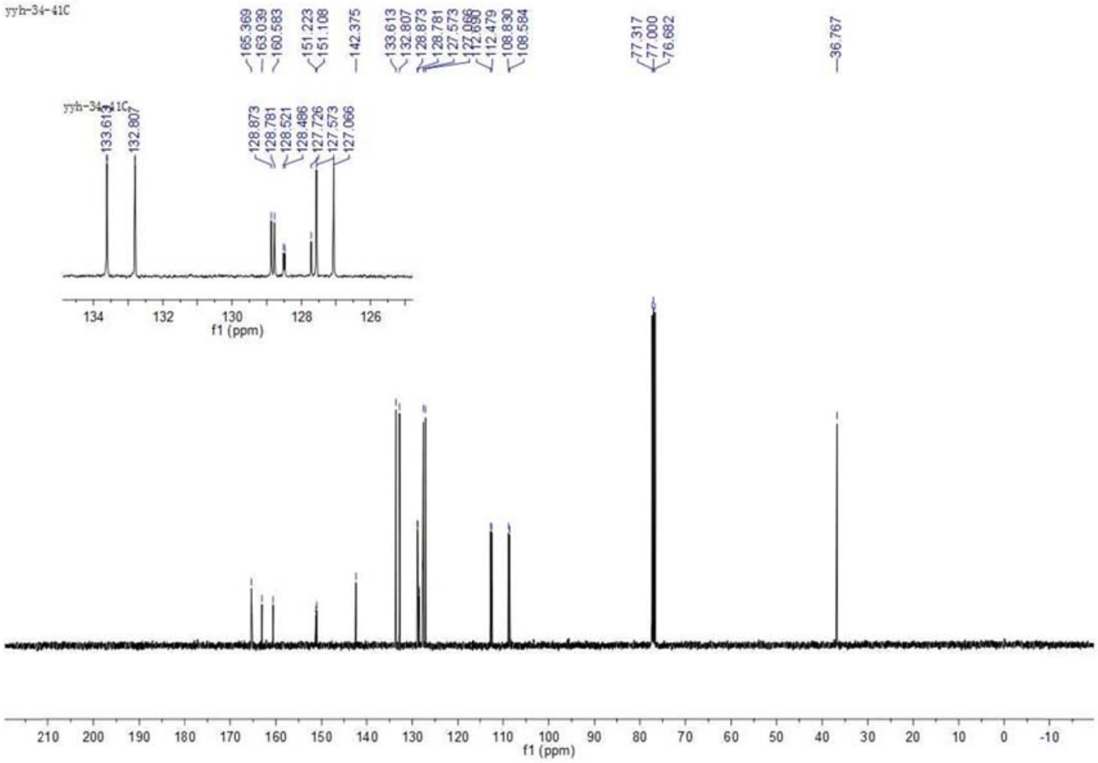
yyh-34-41C
165.369
163.039
160.583
151.223
151.108
142.375
133.613
132.807
77.317
77.000
76.682
36.767
f1 (ppm)

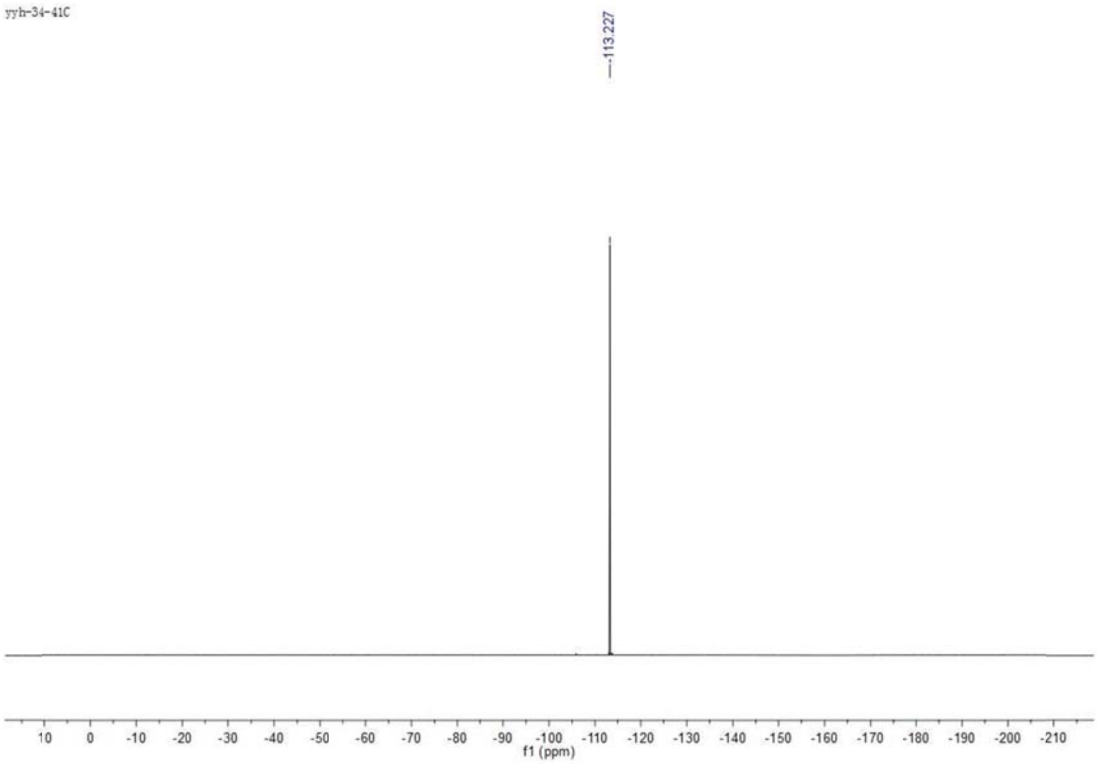

Supplementary Figure 33 ^{1}H NMR, ^{13}C NMR and ^{19}F NMR spectra for compound 18

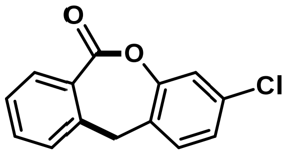

19

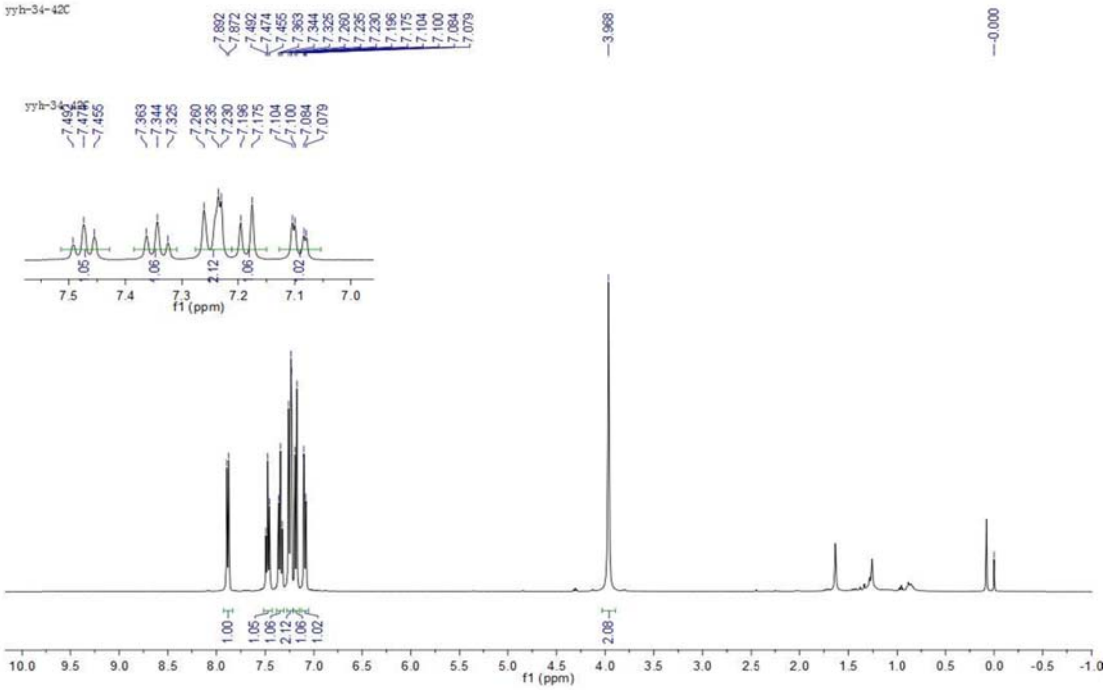

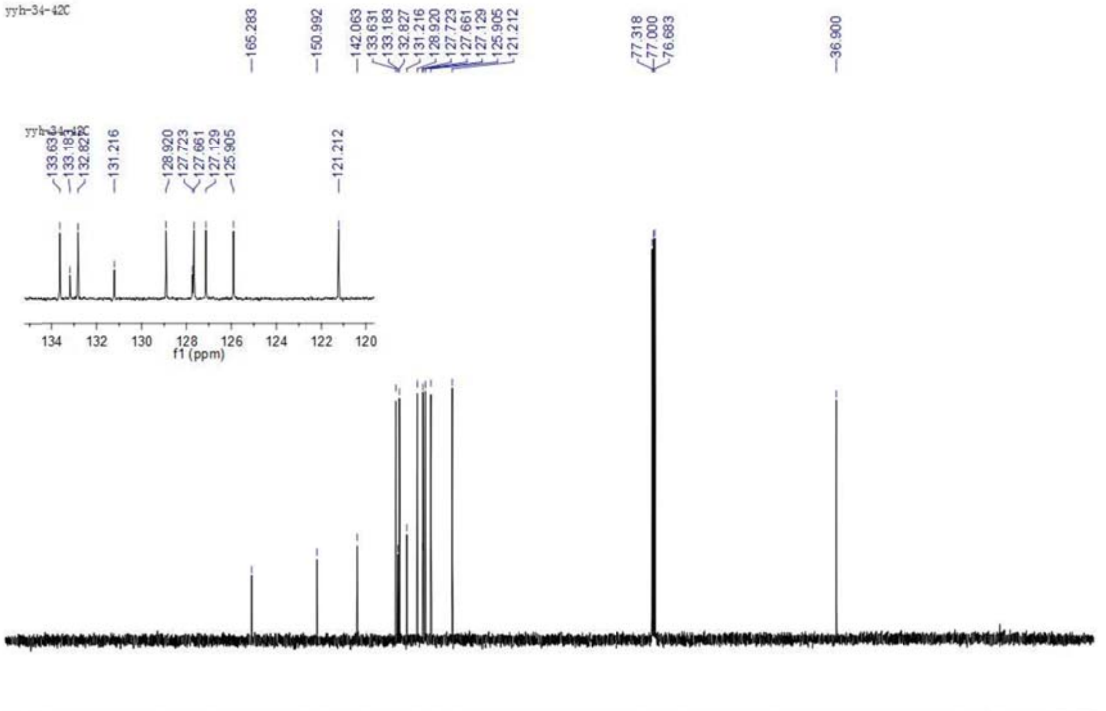

Supplementary Figure 34 ^{1}H NMR and ^{13}C NMR spectra for compound 19

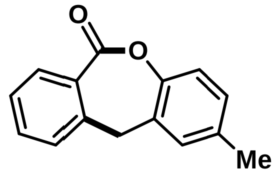

20

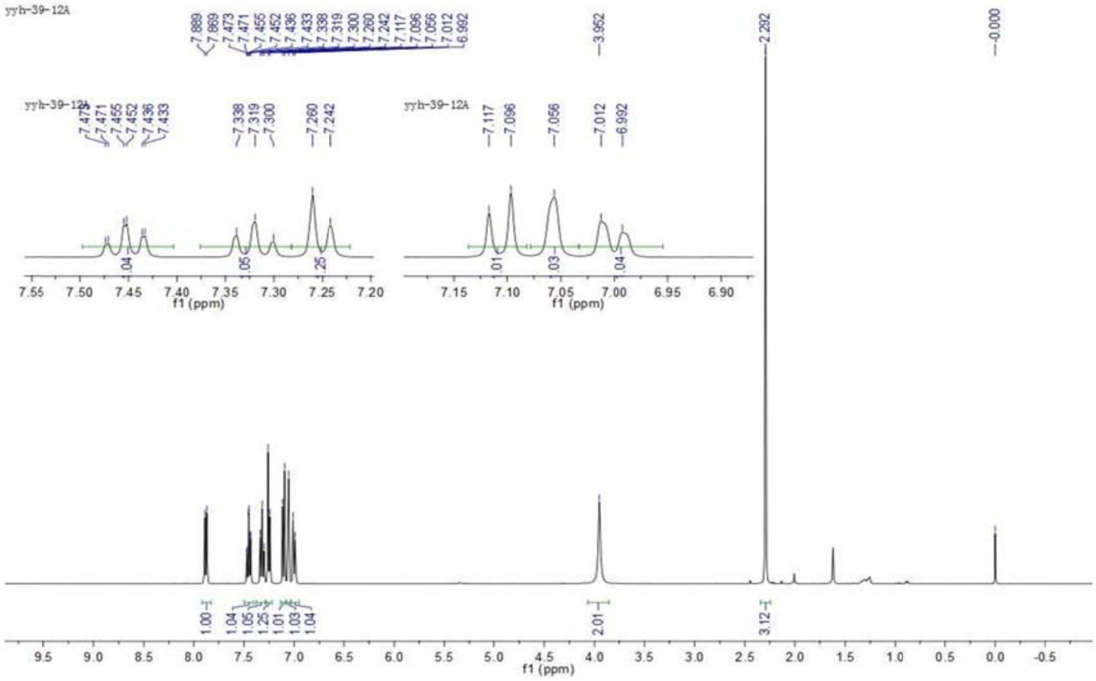

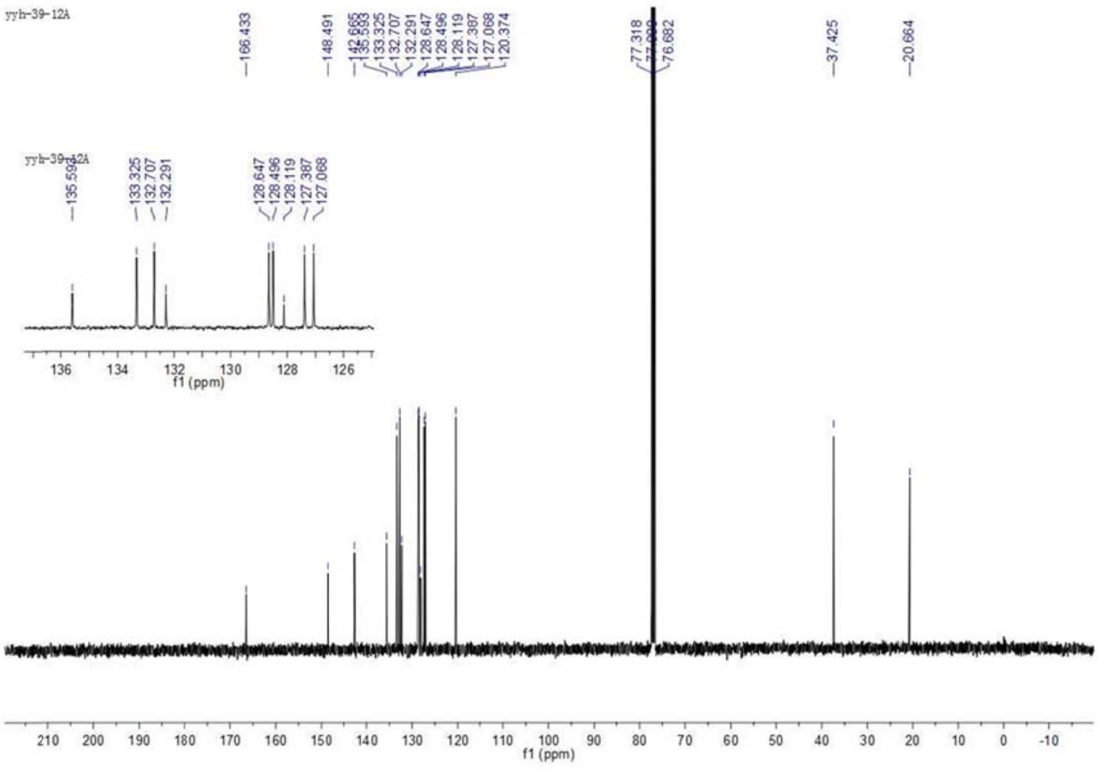

Supplementary Figure 35 ^{1}H NMR and ^{13}C NMR spectra for compound 20

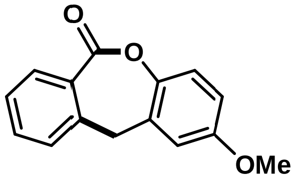

21

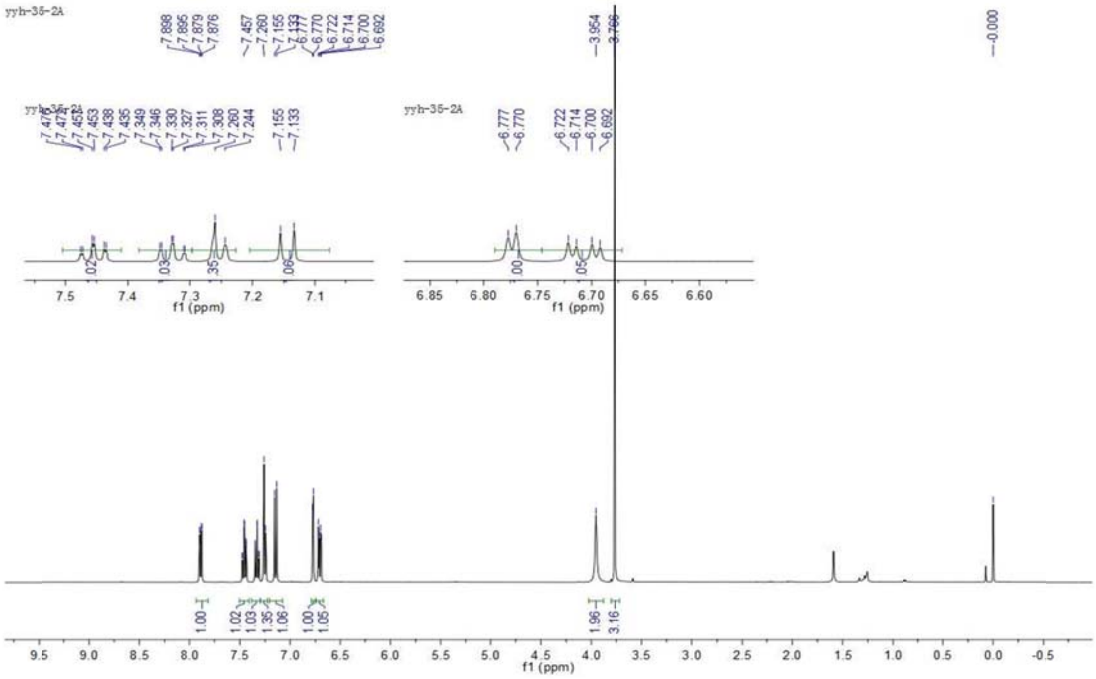

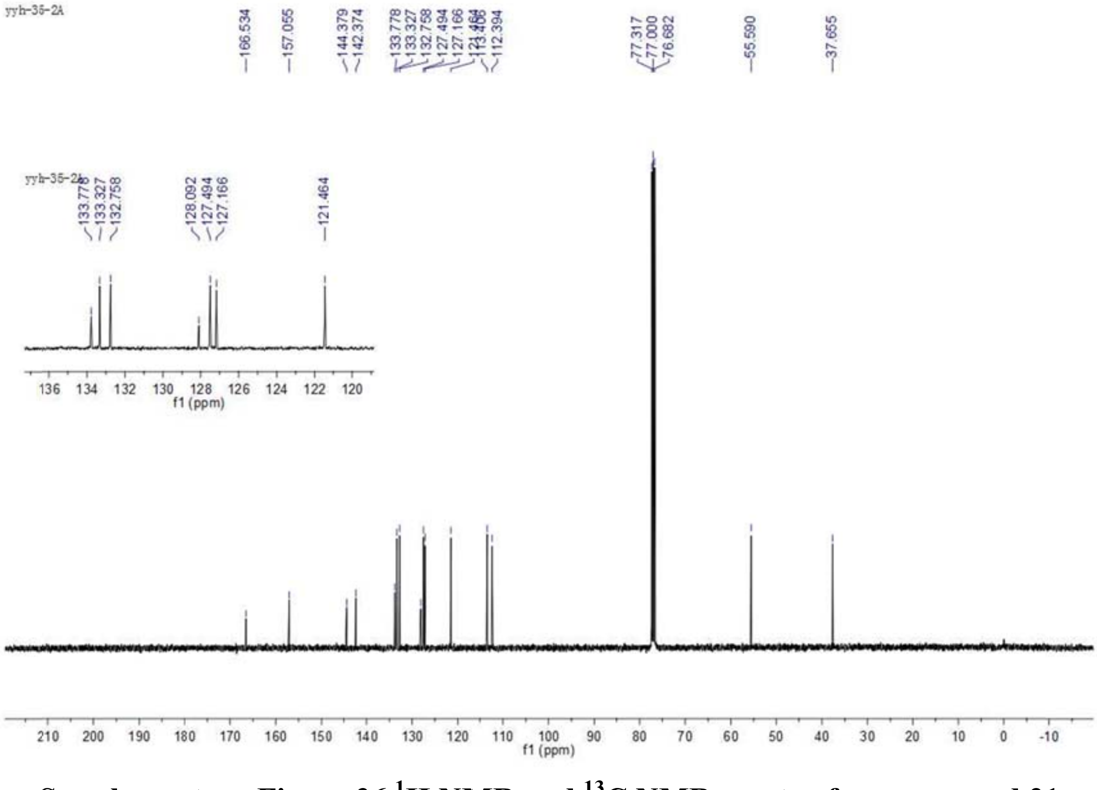

Supplementary Figure 36 ^{1}H NMR and ^{13}C NMR spectra for compound 21

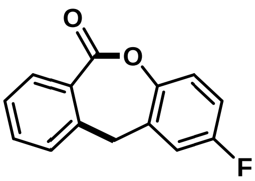
O
O
F

22

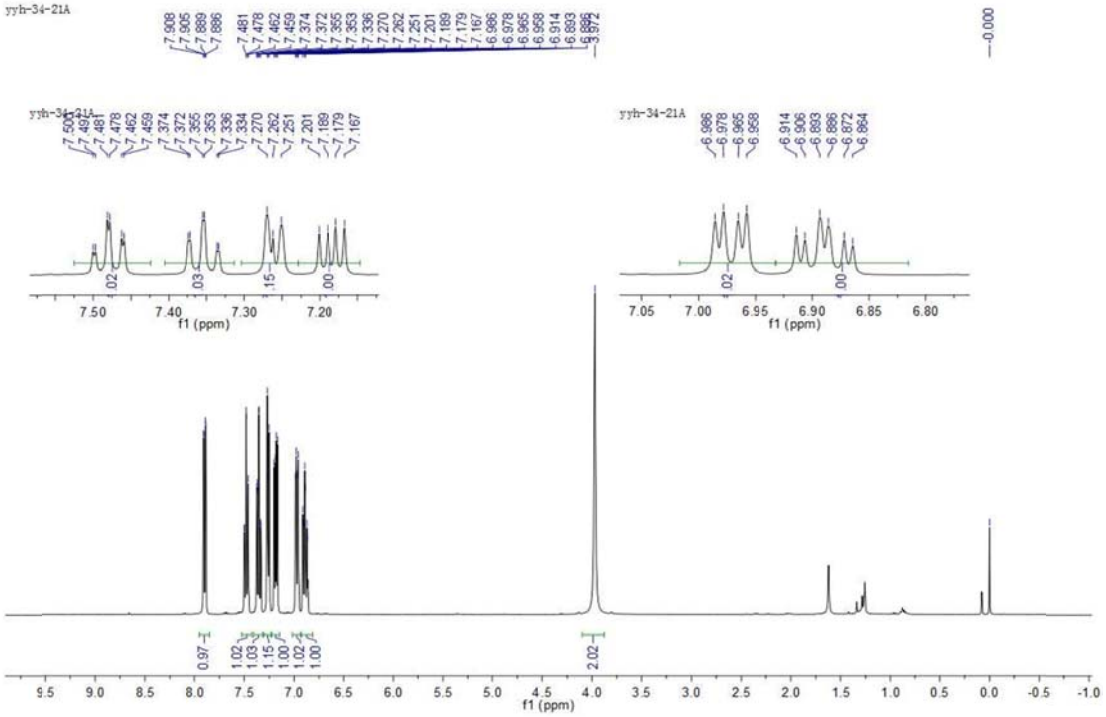
yyh-34-21A
7.908
7.905
7.889
7.886
7.481
7.478
7.462
7.459
7.374
7.372
7.355
7.353
7.336
7.270
7.262
7.251
7.201
7.189
7.179
7.167
6.986
6.978
6.965
6.958
6.914
6.893
3.972
0.000
0.97
1.02
1.03
1.15
1.00
1.02
1.00
2.02
f1 (ppm)

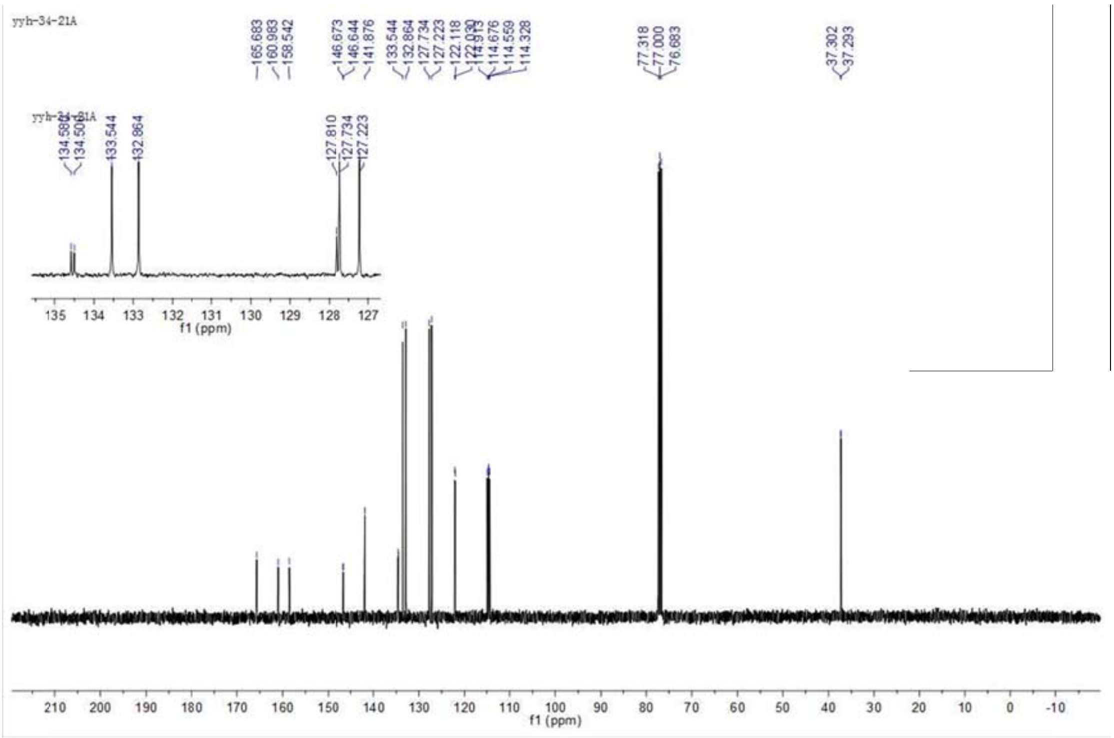
yyh-34-21A
165.683
160.983
158.542
146.673
146.644
141.876
133.544
132.864
127.734
127.223
122.118
114.913
114.676
114.559
114.328
77.318
77.000
76.683
37.302
37.293
f1 (ppm)

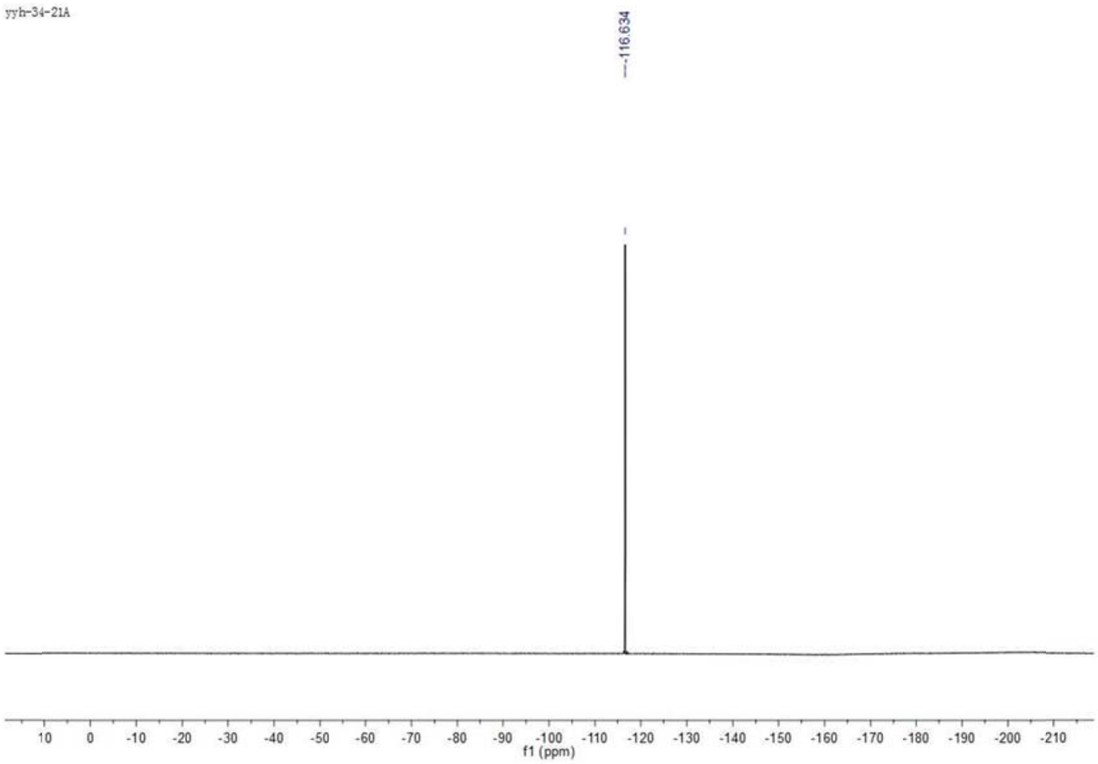

Supplementary Figure 37 ^{1}H NMR, ^{13}C NMR and ^{19}F NMR spectra for compound 22

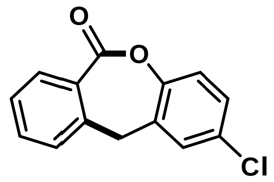

23

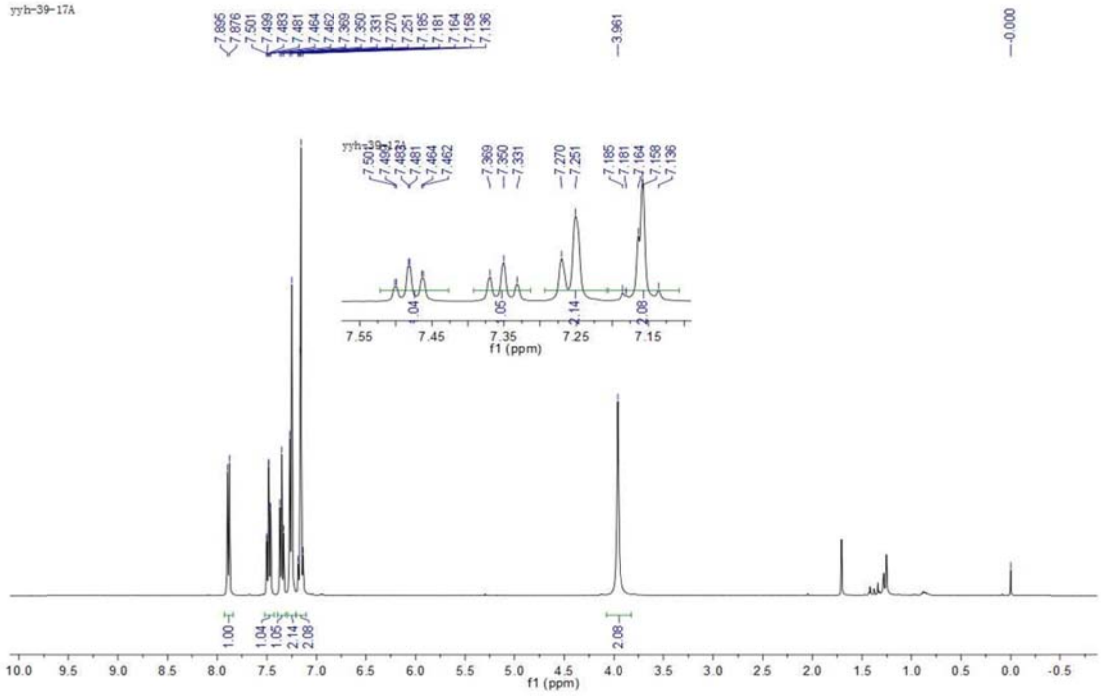

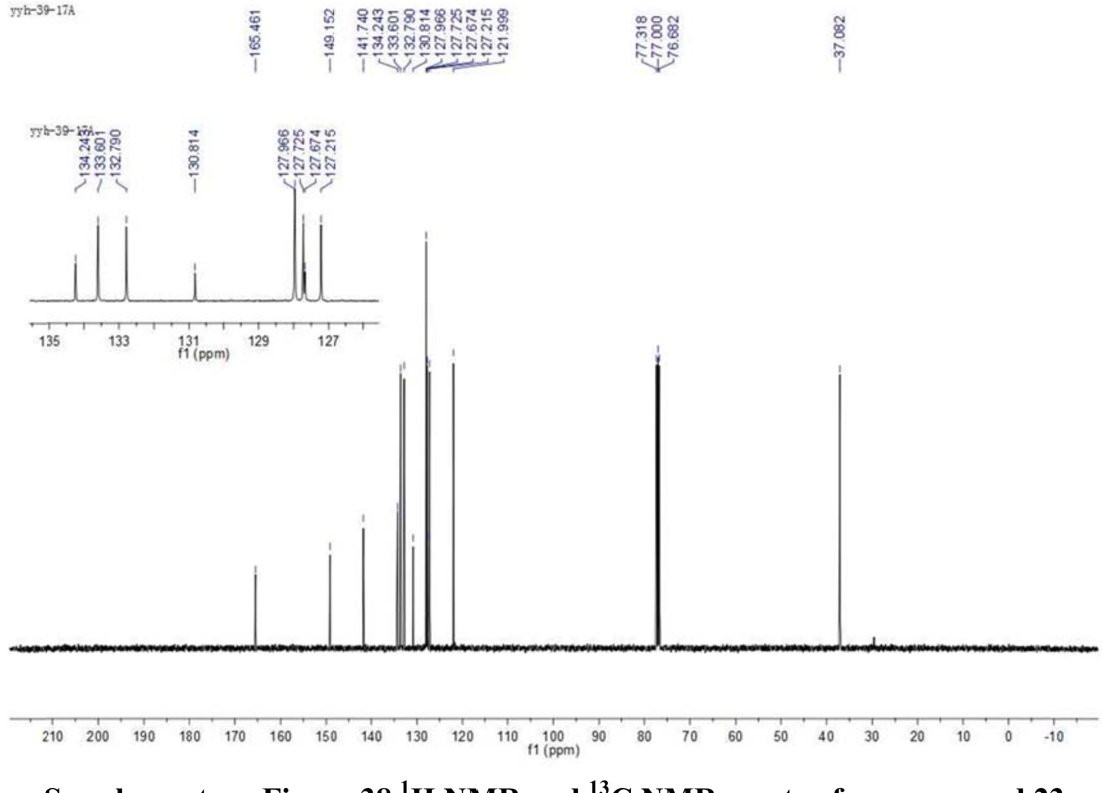

Supplementary Figure 38 ^{1}H NMR and ^{13}C NMR spectra for compound 23

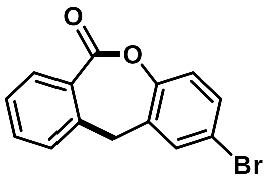

24

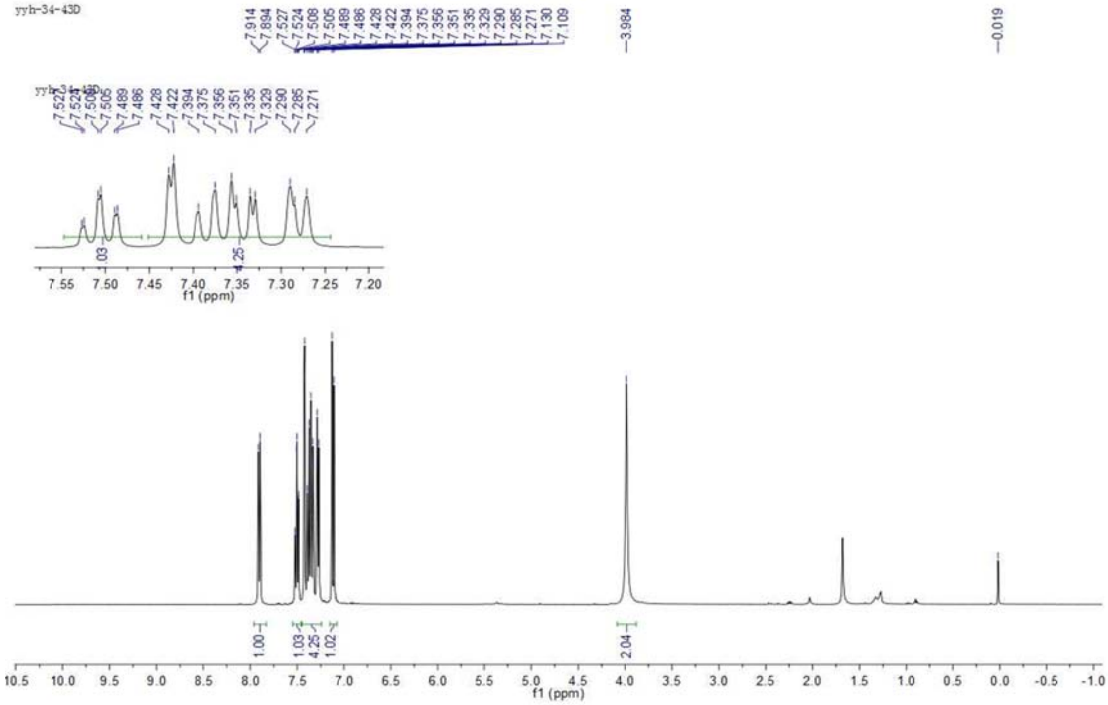

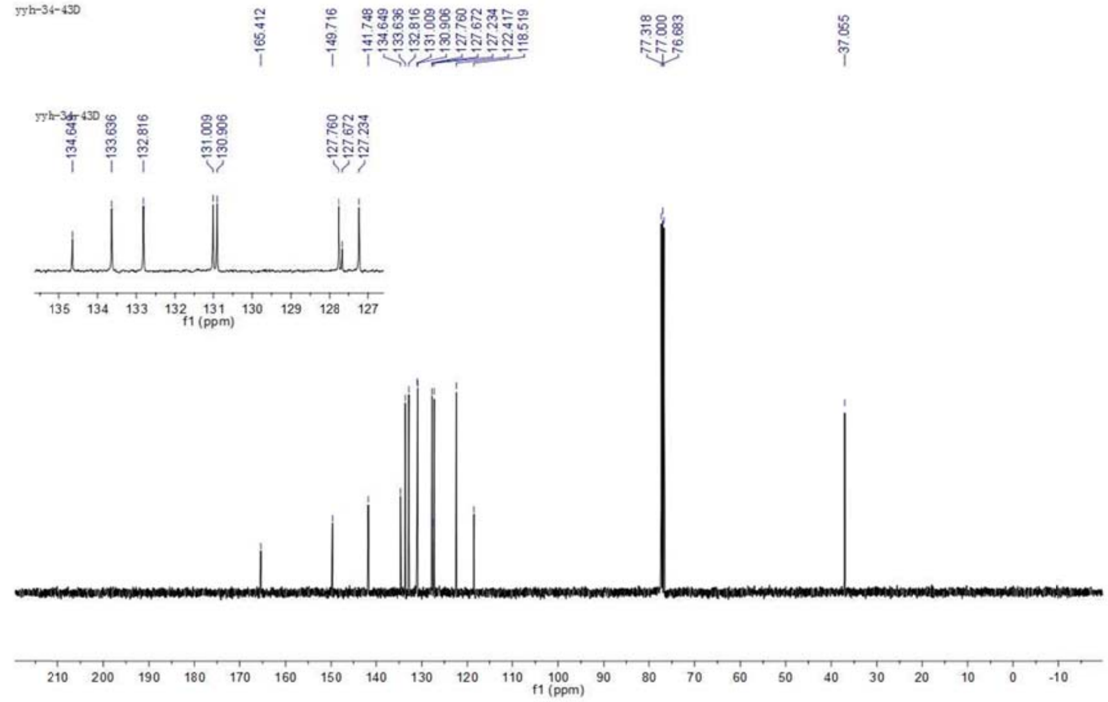

Supplementary Figure 39 ^{1}H NMR and ^{13}C NMR spectra for compound 24

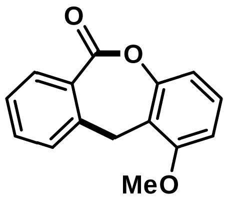

25

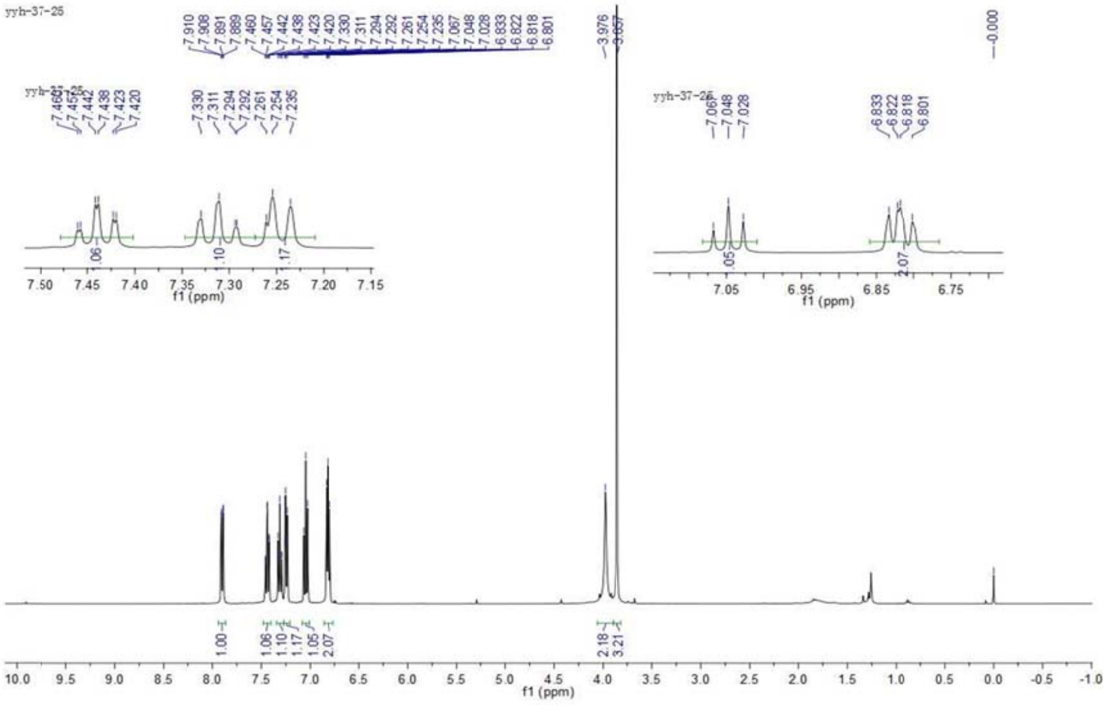

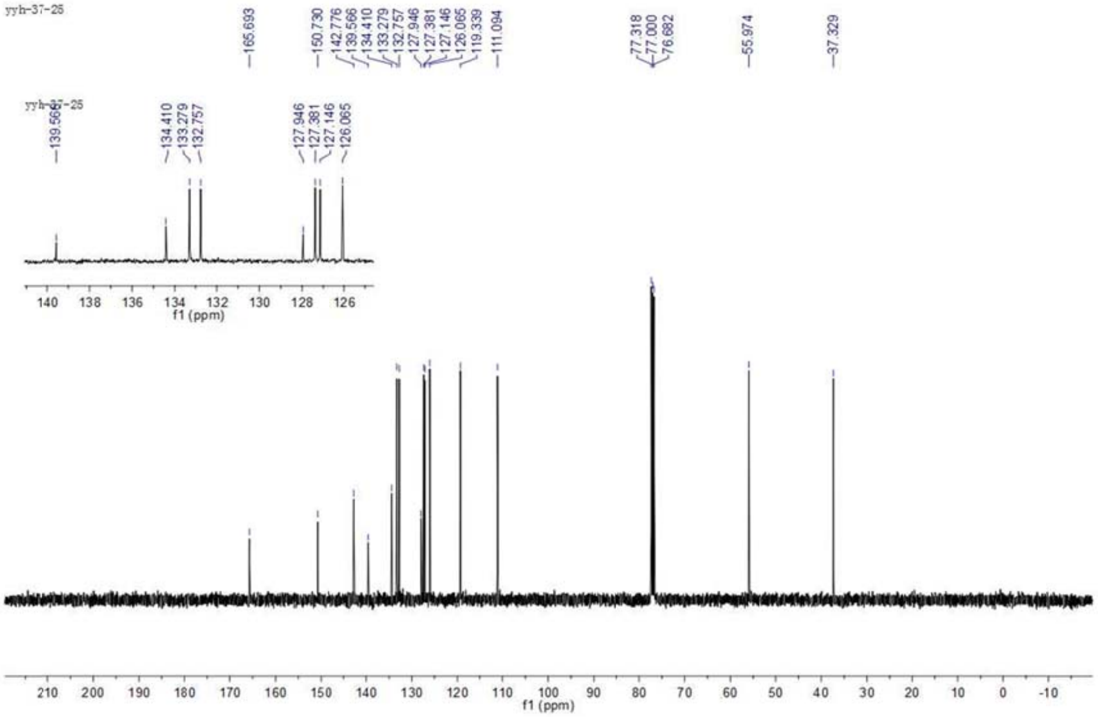

Supplementary Figure 40 ^{1}H NMR and ^{13}C NMR spectra for compound 25

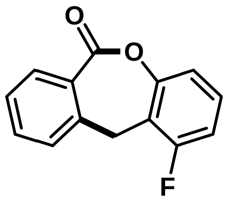

26

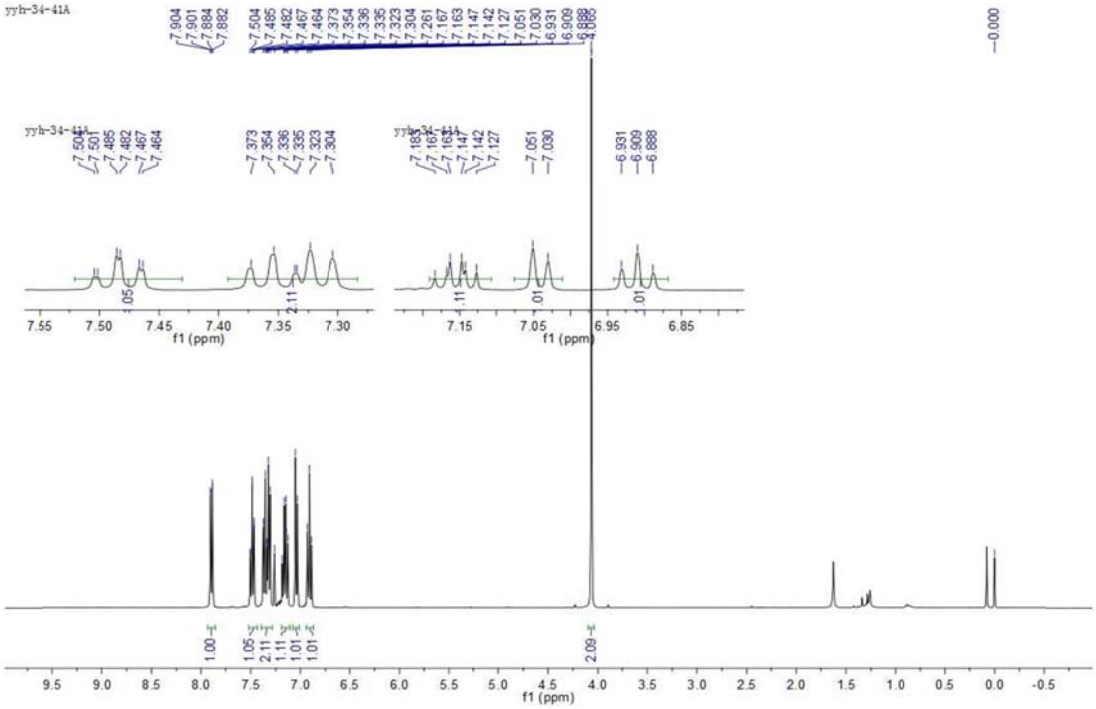

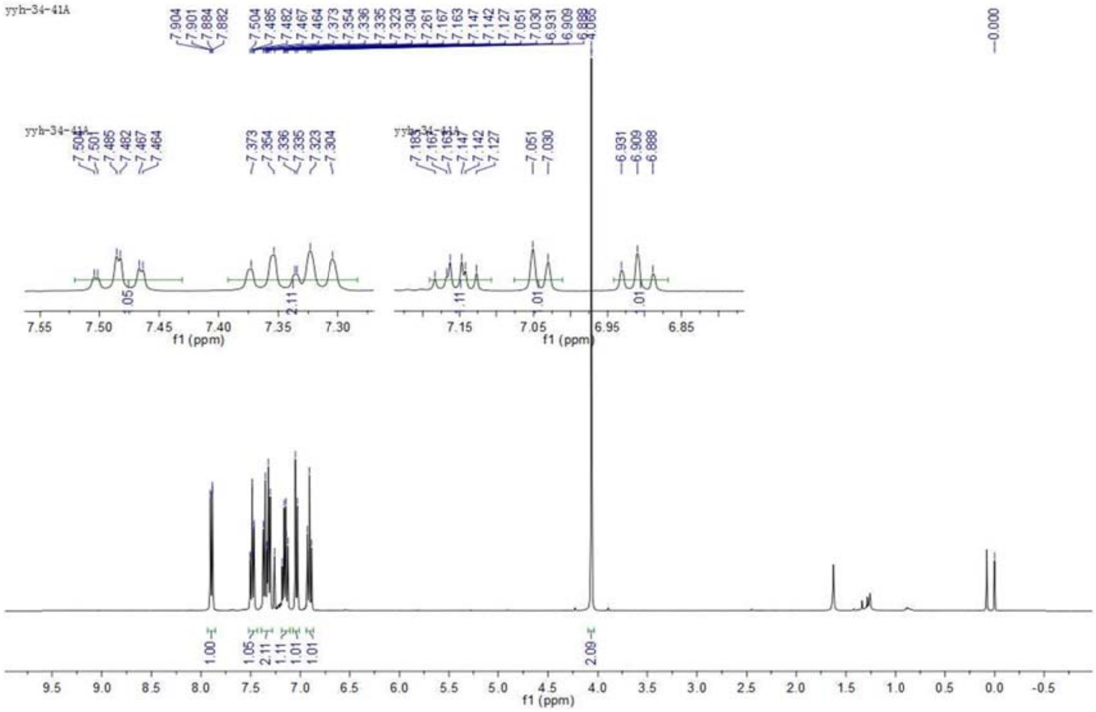

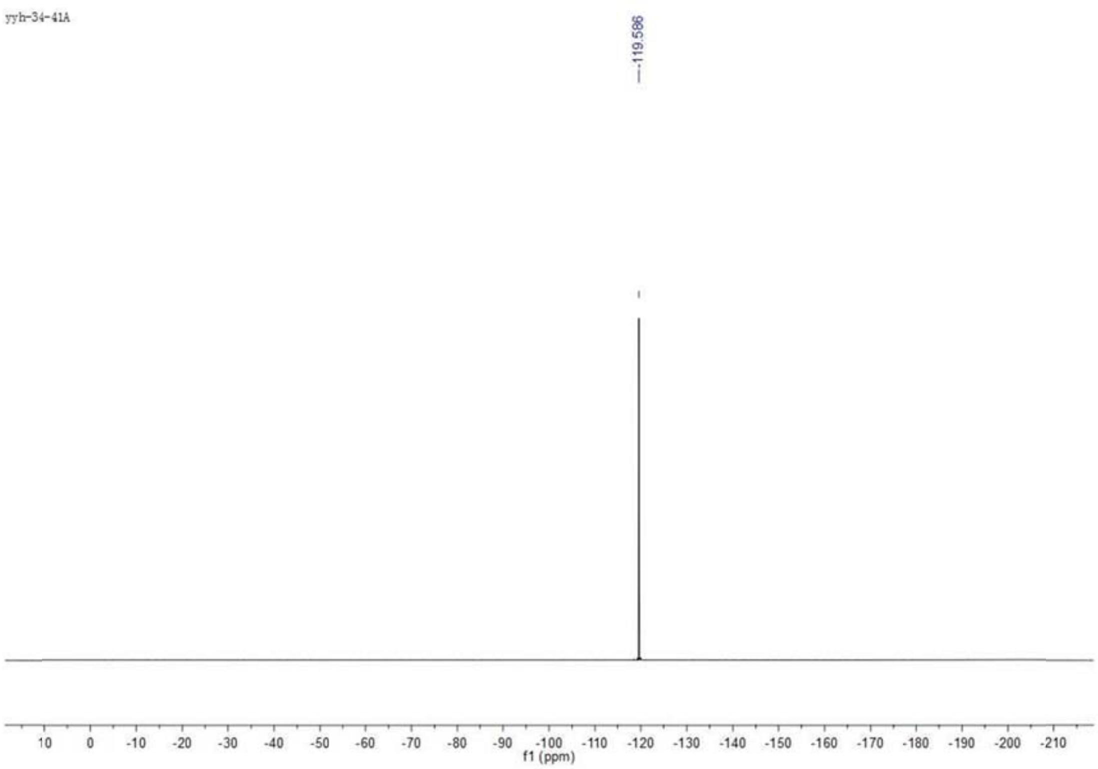

Supplementary Figure 41 ^{1}H NMR, ^{13}C NMR and ^{19}F NMR spectra for compound 26

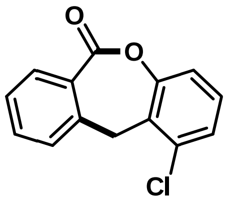

27

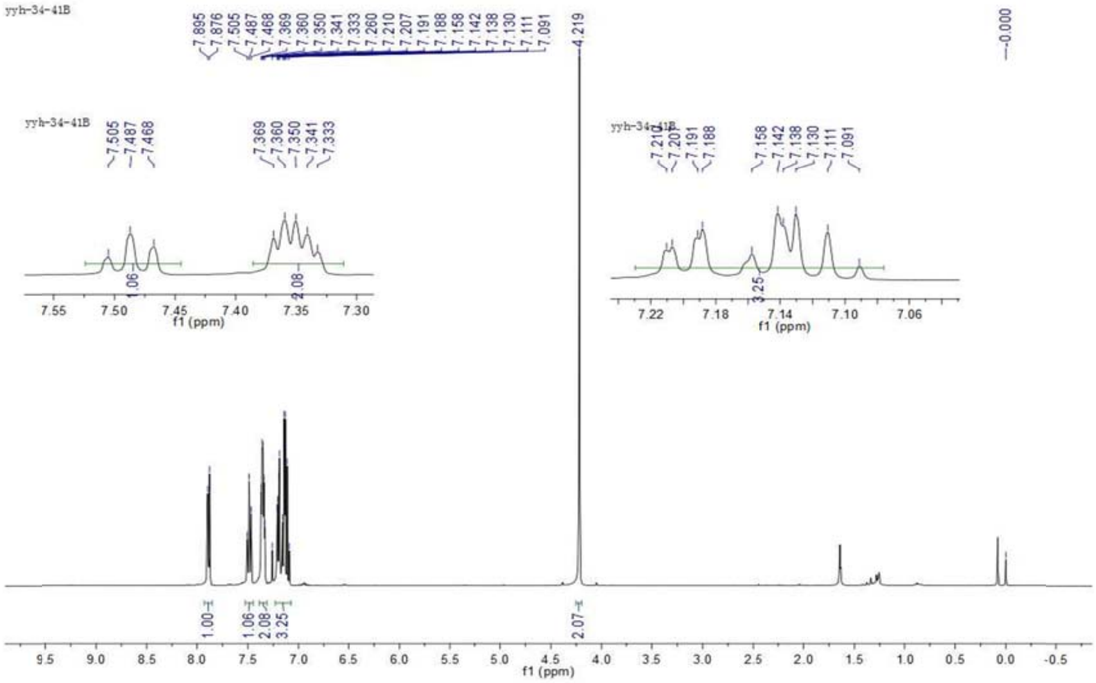

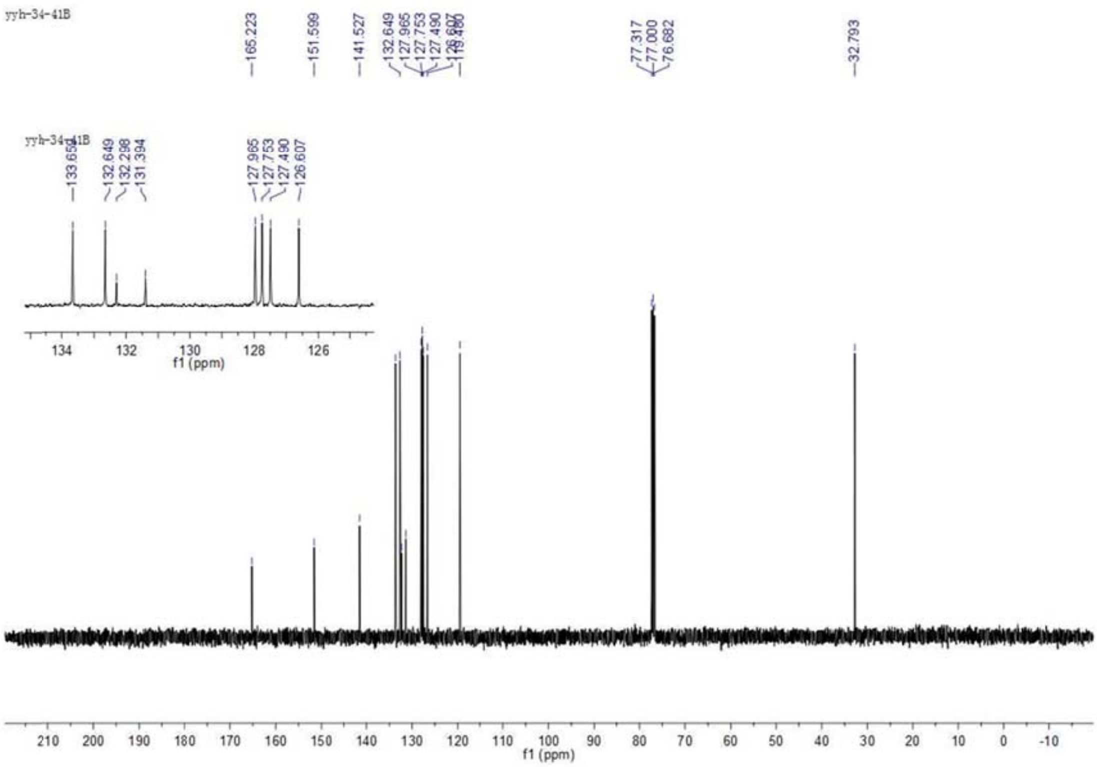

Supplementary Figure 42 ^{1}H NMR and ^{13}C NMR spectra for compound 27

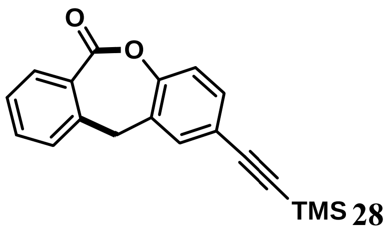

28

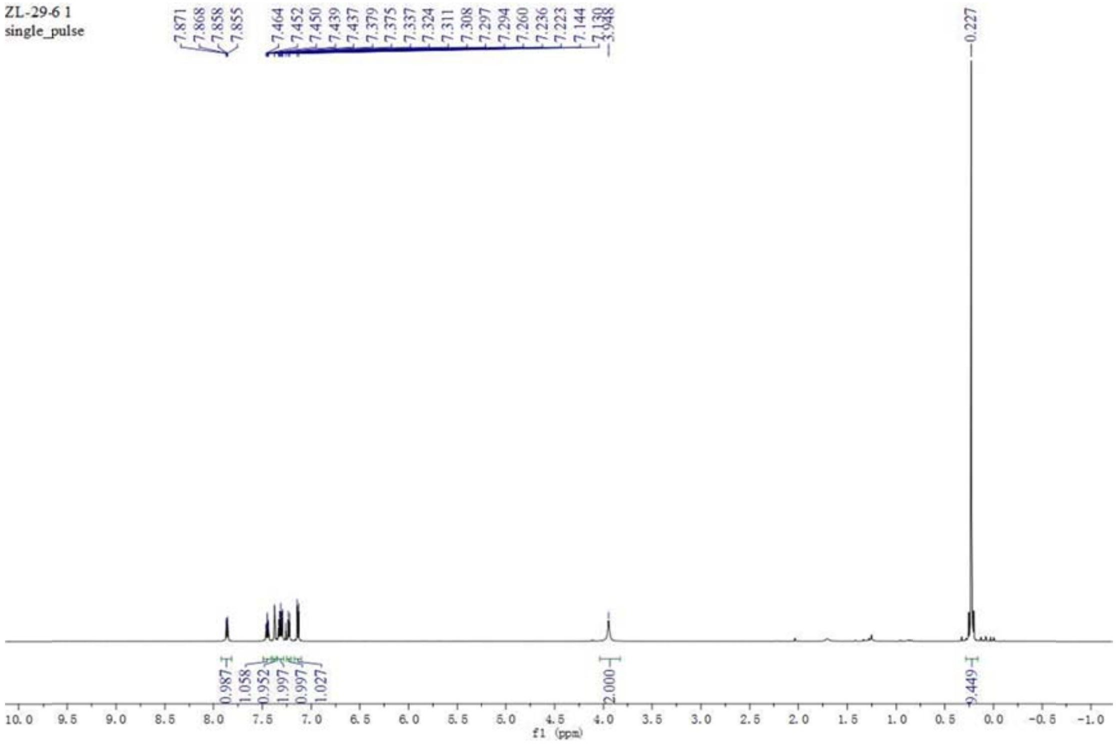

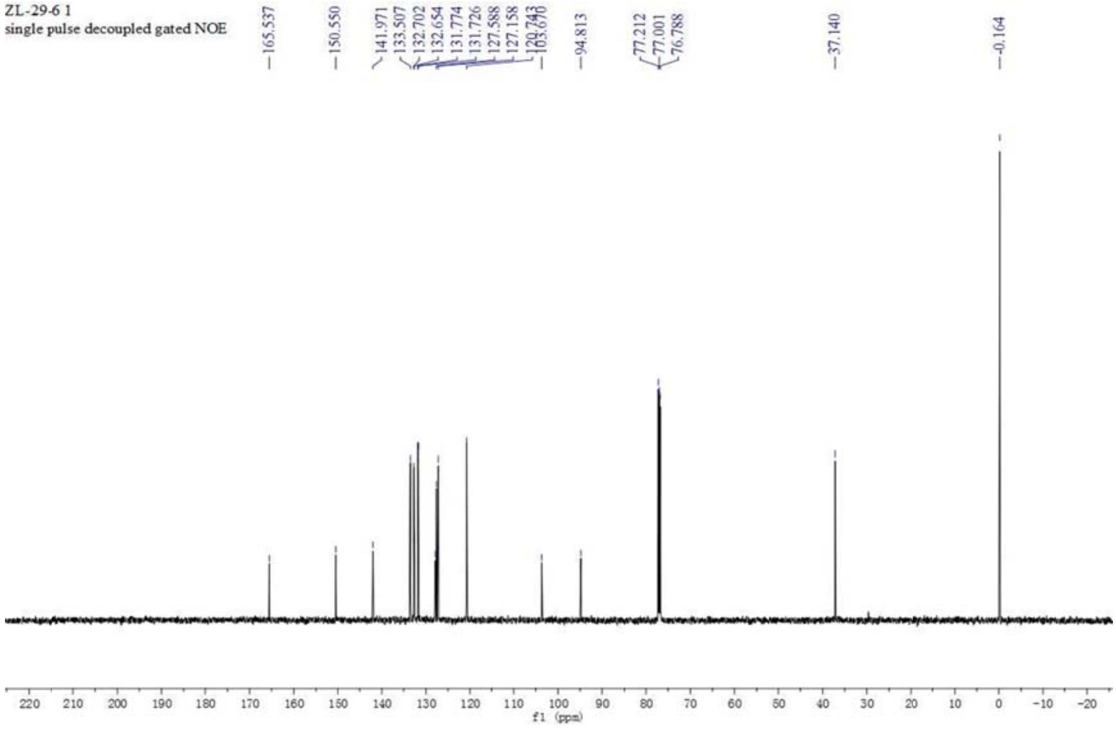

Supplementary Figure 43 ^{1}H NMR and ^{13}C NMR spectra for compound 28

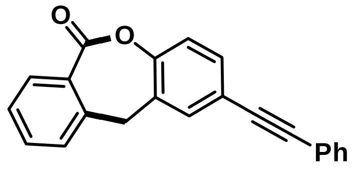

29

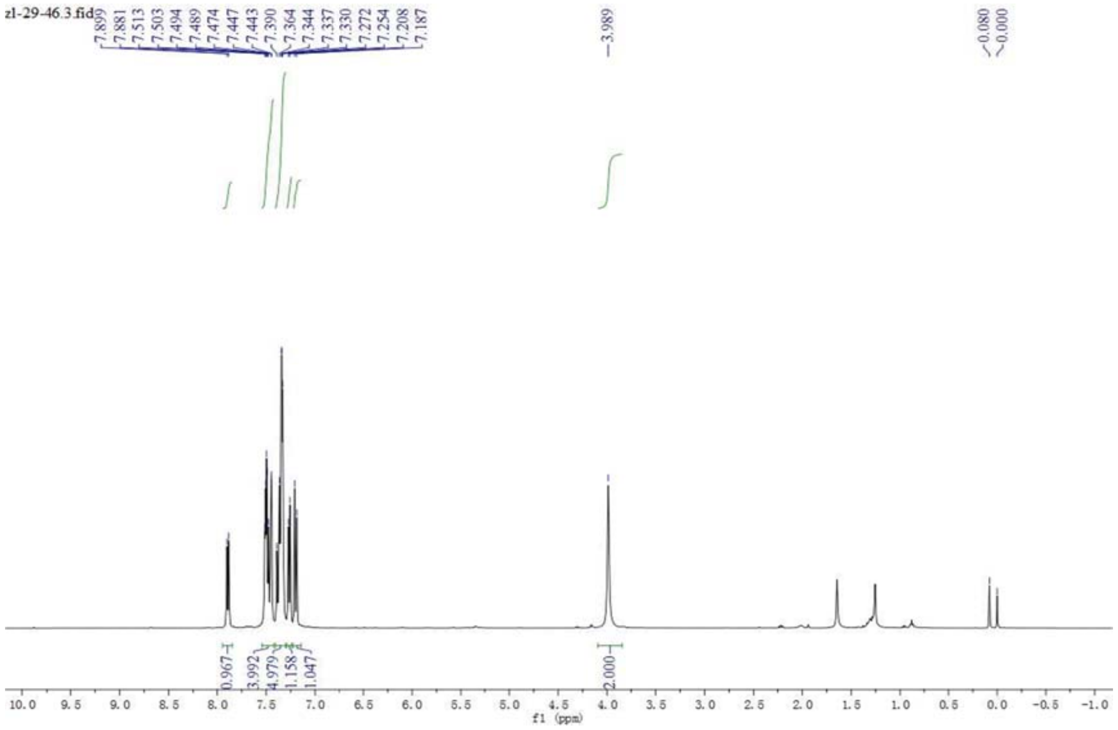

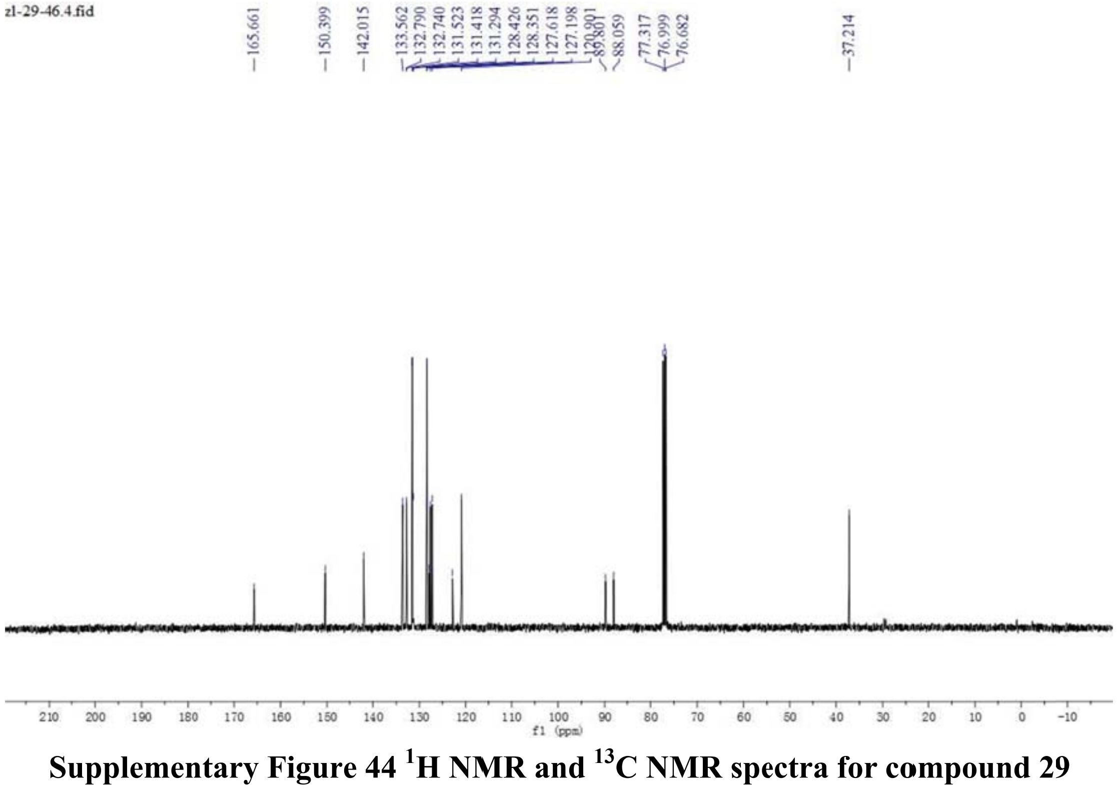

Supplementary Figure 44 ^{1}H NMR and ^{13}C NMR spectra for compound 29

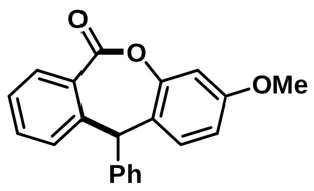

30

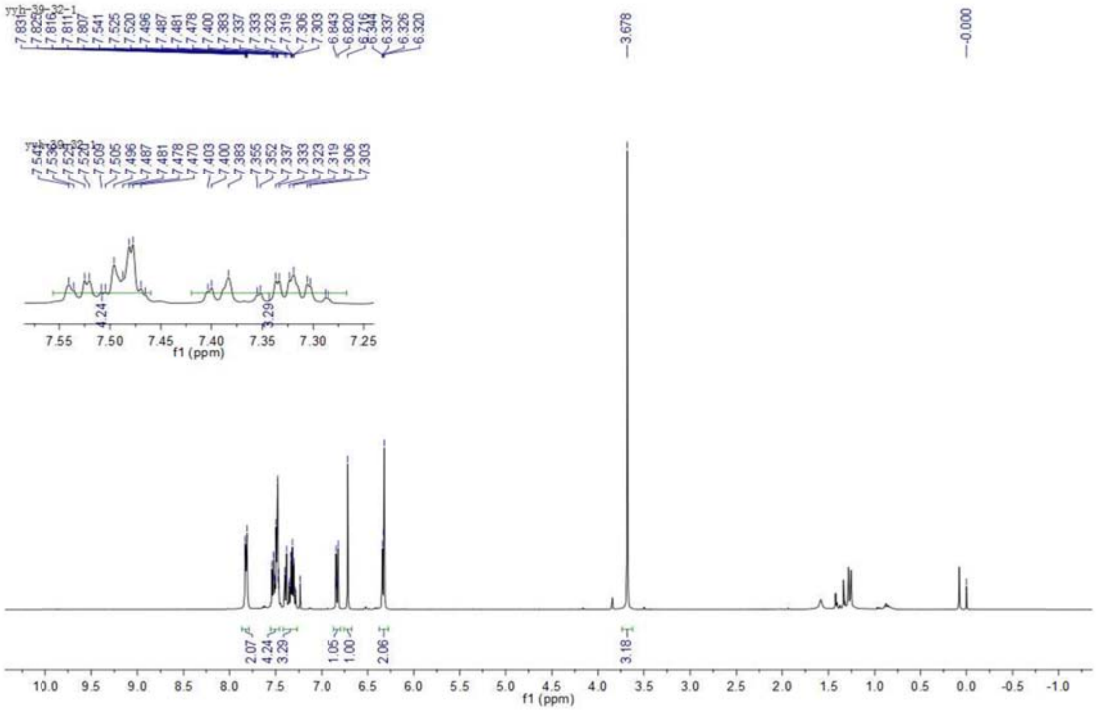

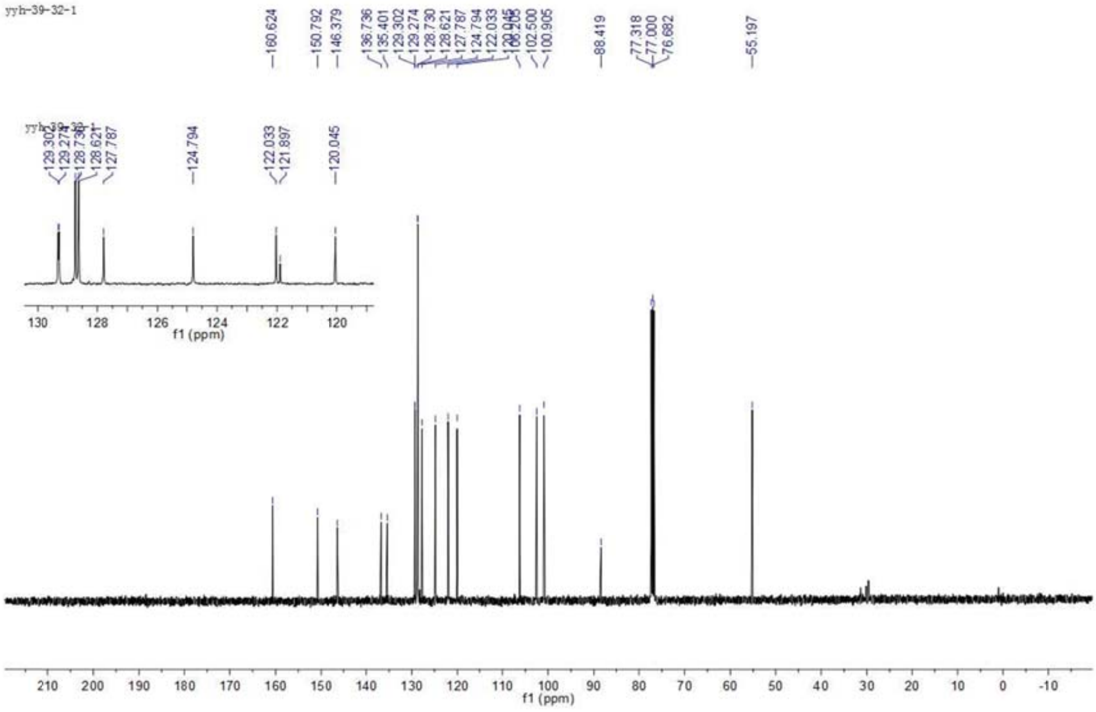

Supplementary Figure 45 ^{1}H NMR and ^{13}C NMR spectra for compound 30

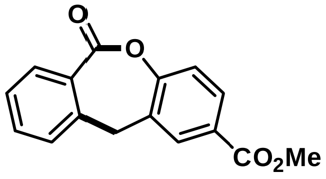

31

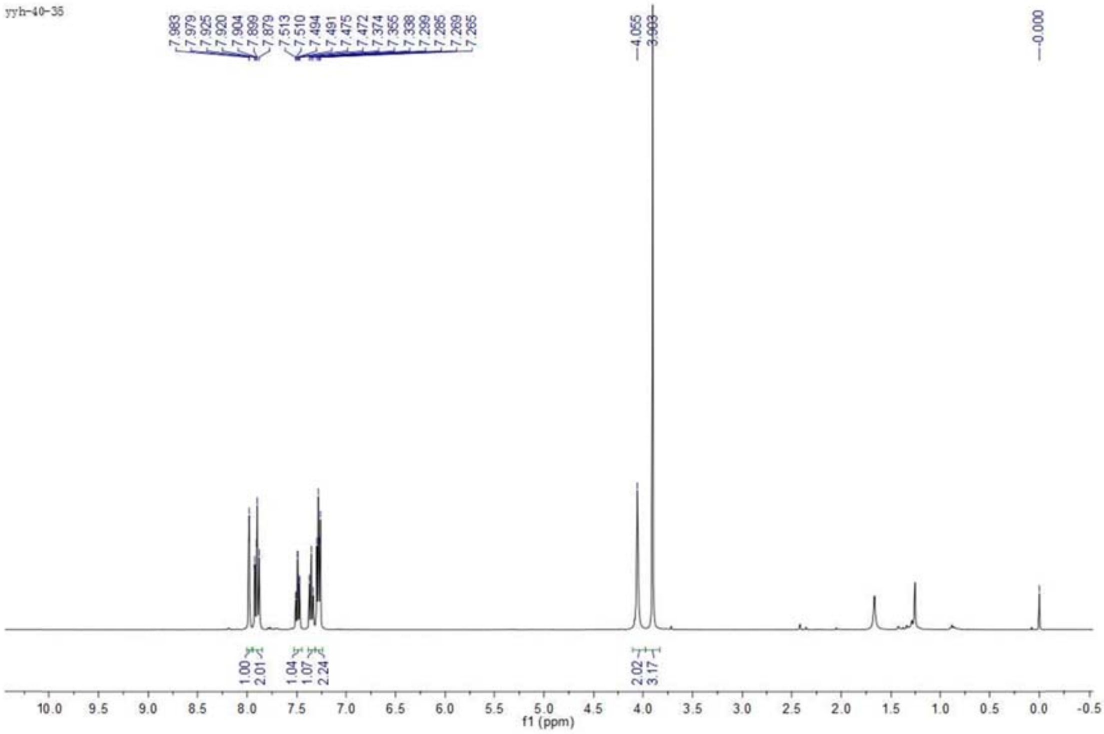

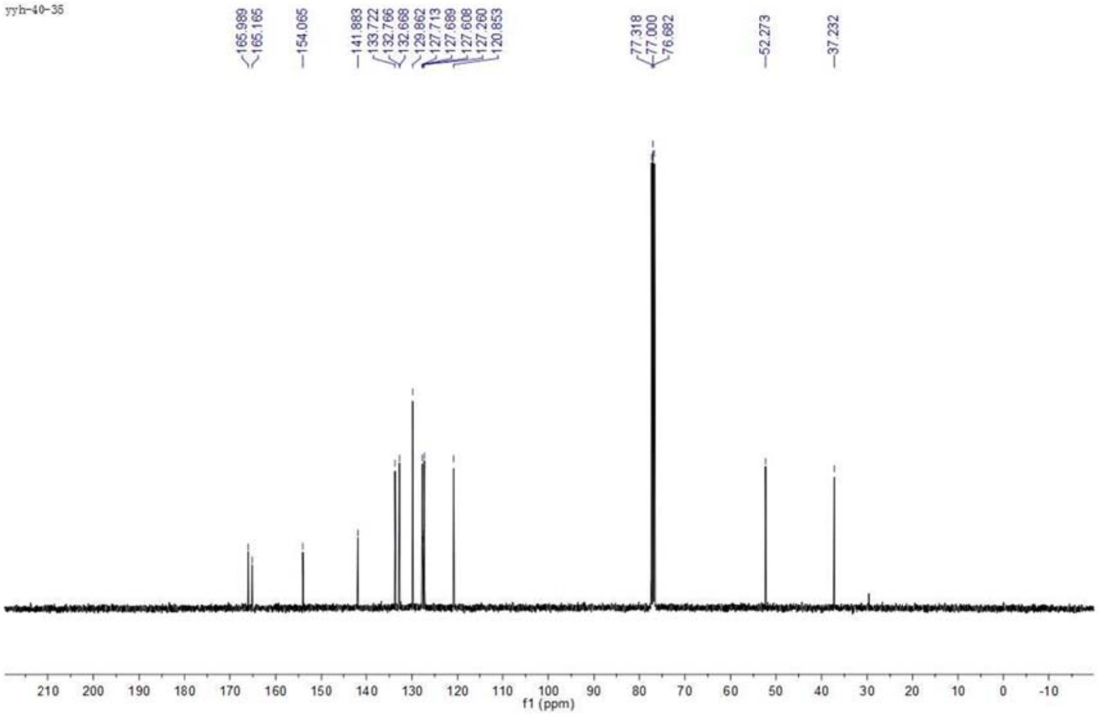

Supplementary Figure 46 ^{1}H NMR and ^{13}C NMR spectra for compound 31

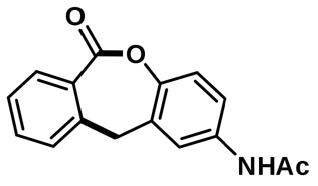

32

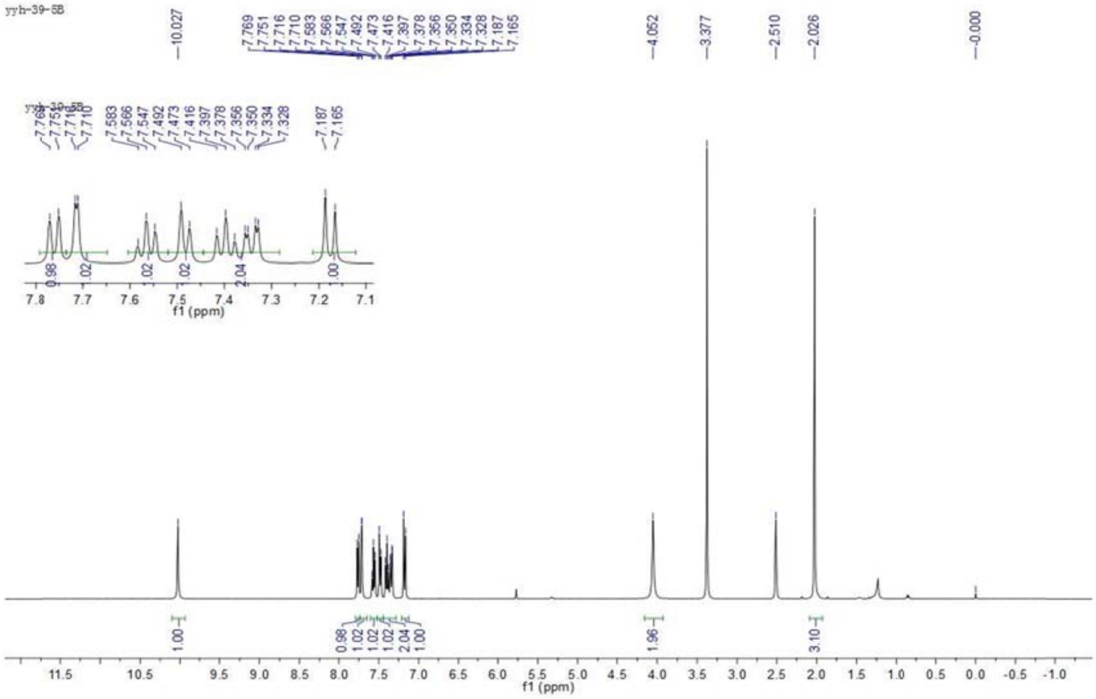

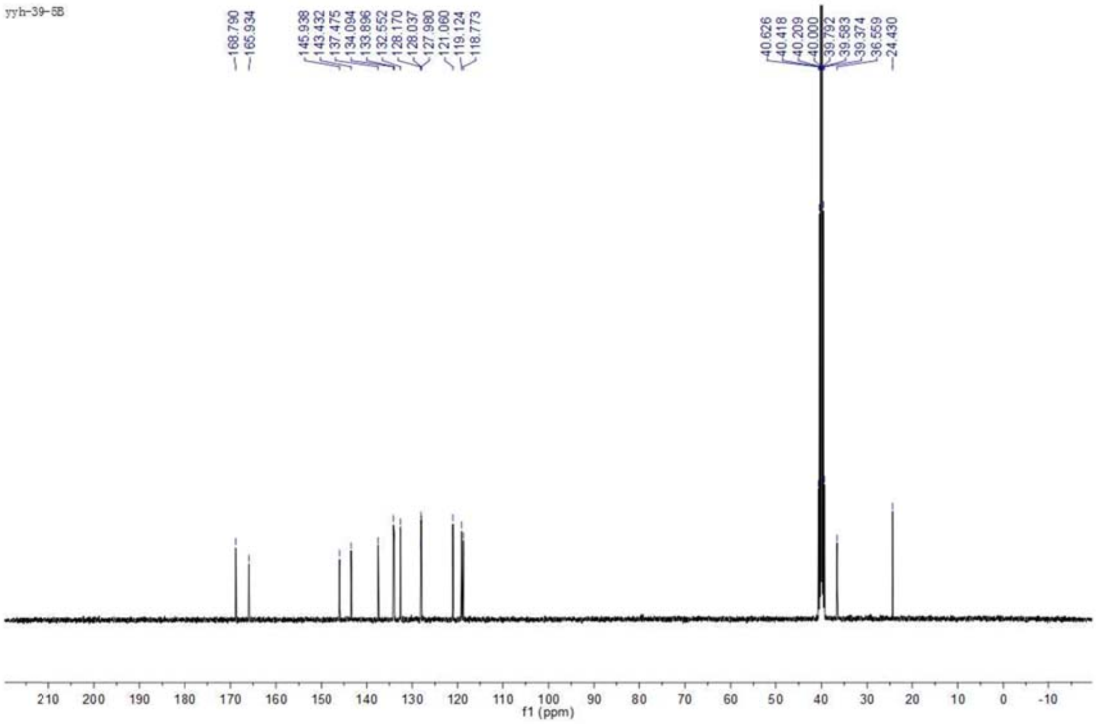

Supplementary Figure 47 ^{1}H NMR and ^{13}C NMR spectra for compound 32

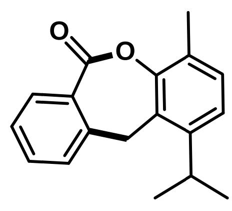

33

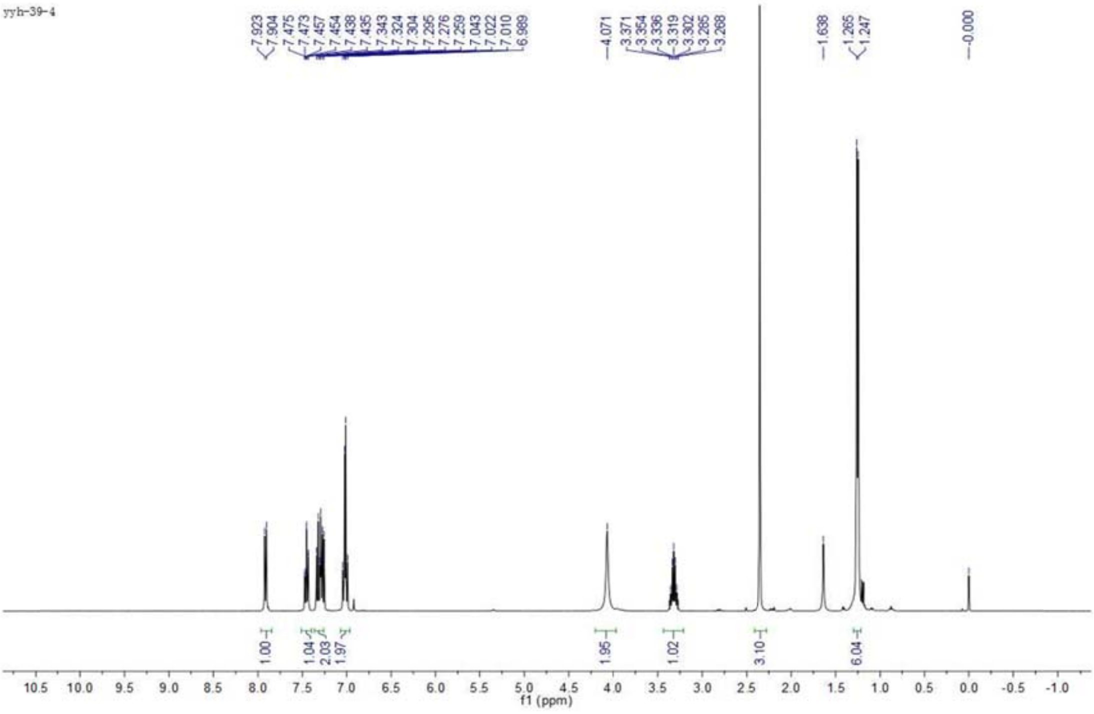

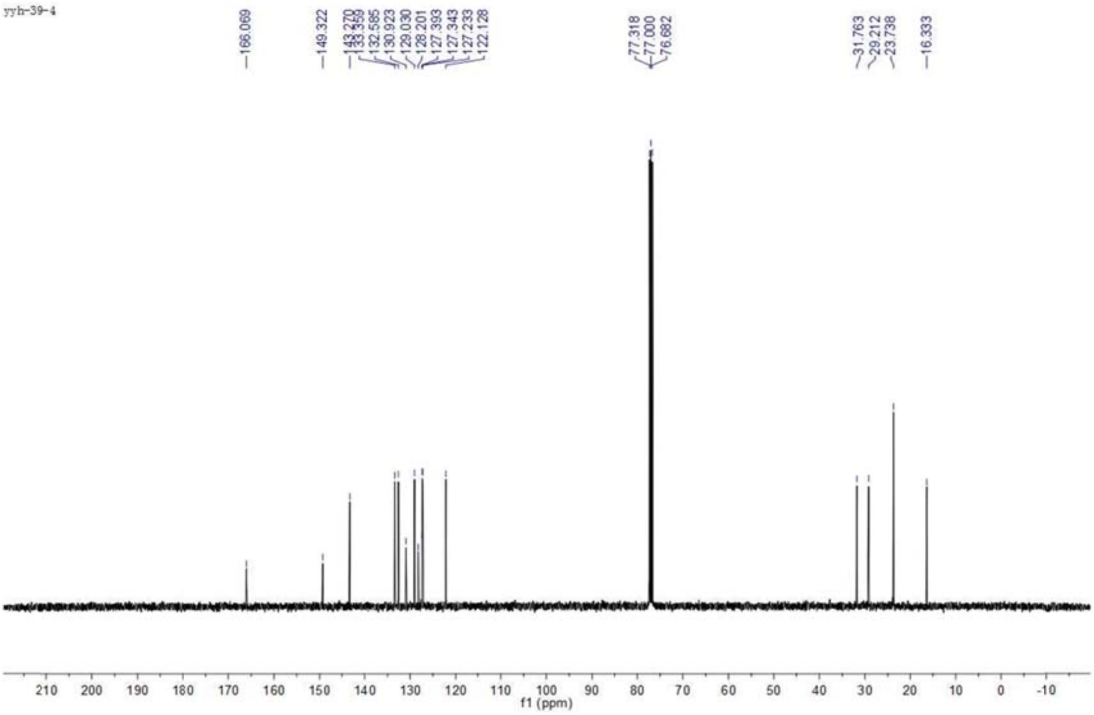

Supplementary Figure 48 ^{1}H NMR and ^{13}C NMR spectra for compound 33

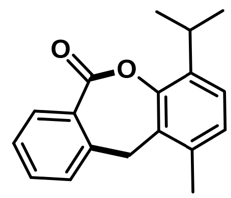

34

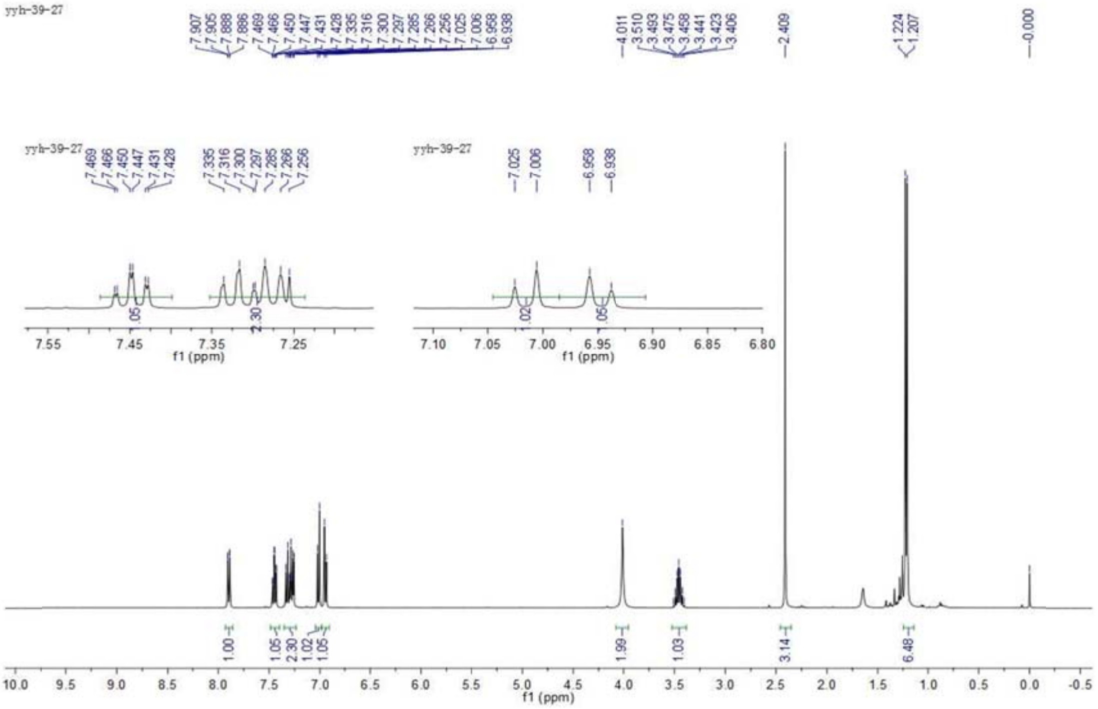

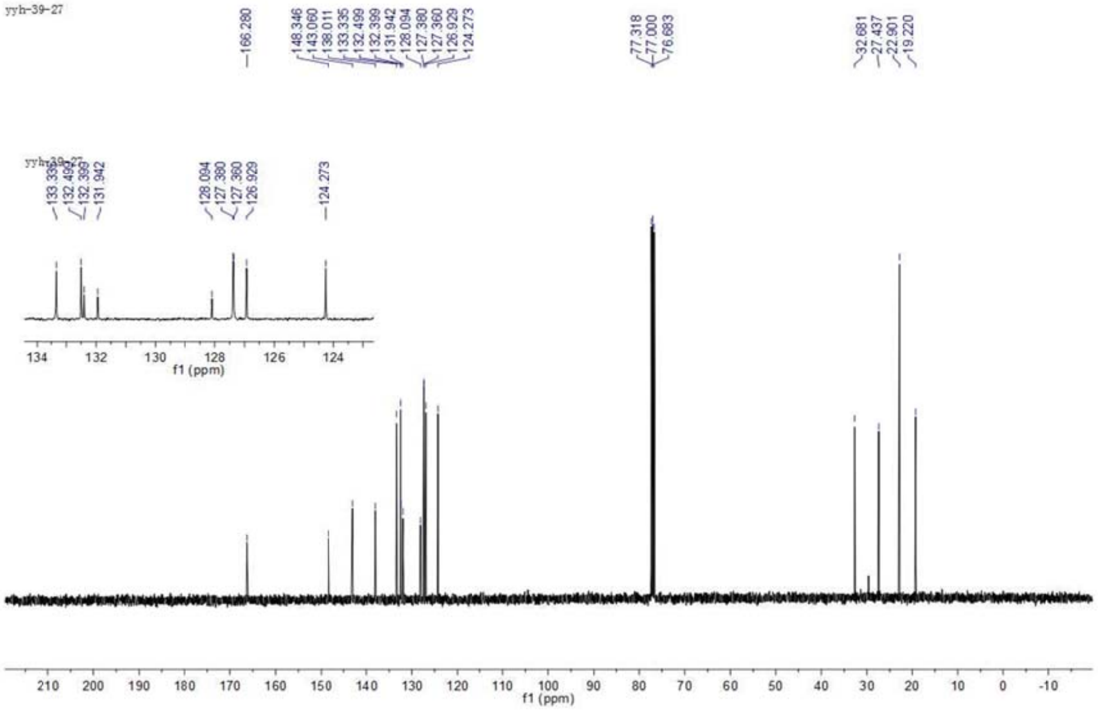

Supplementary Figure 49 ^{1}H NMR and ^{13}C NMR spectra for compound 34

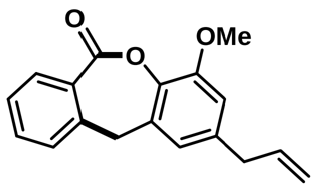

35

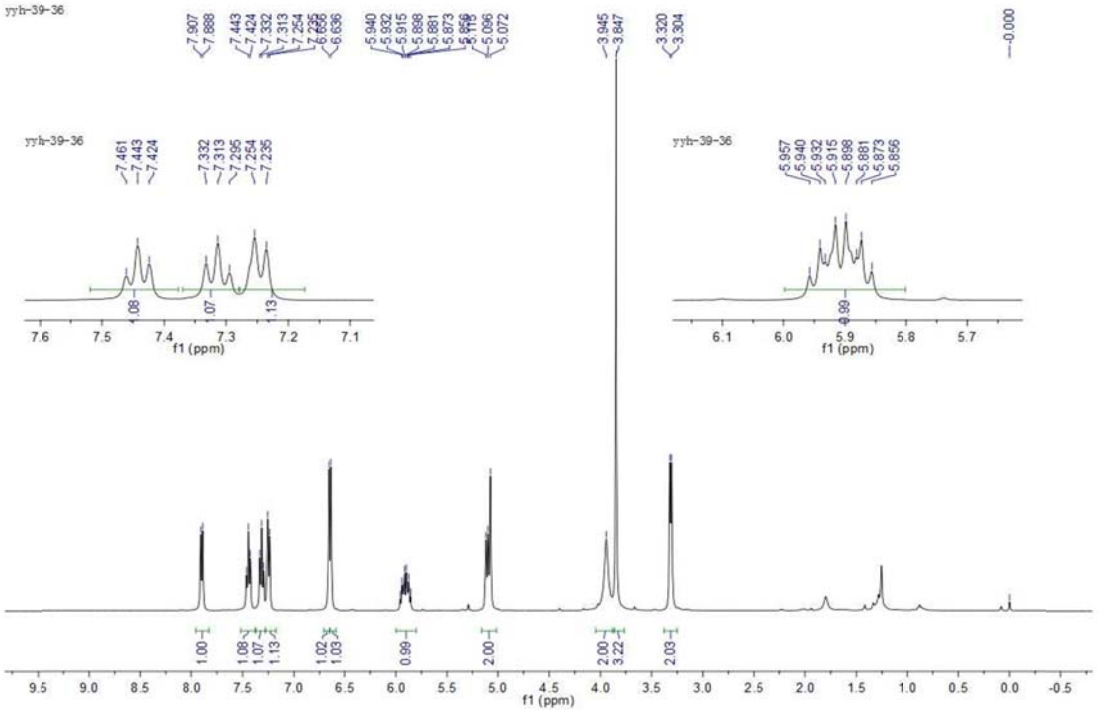

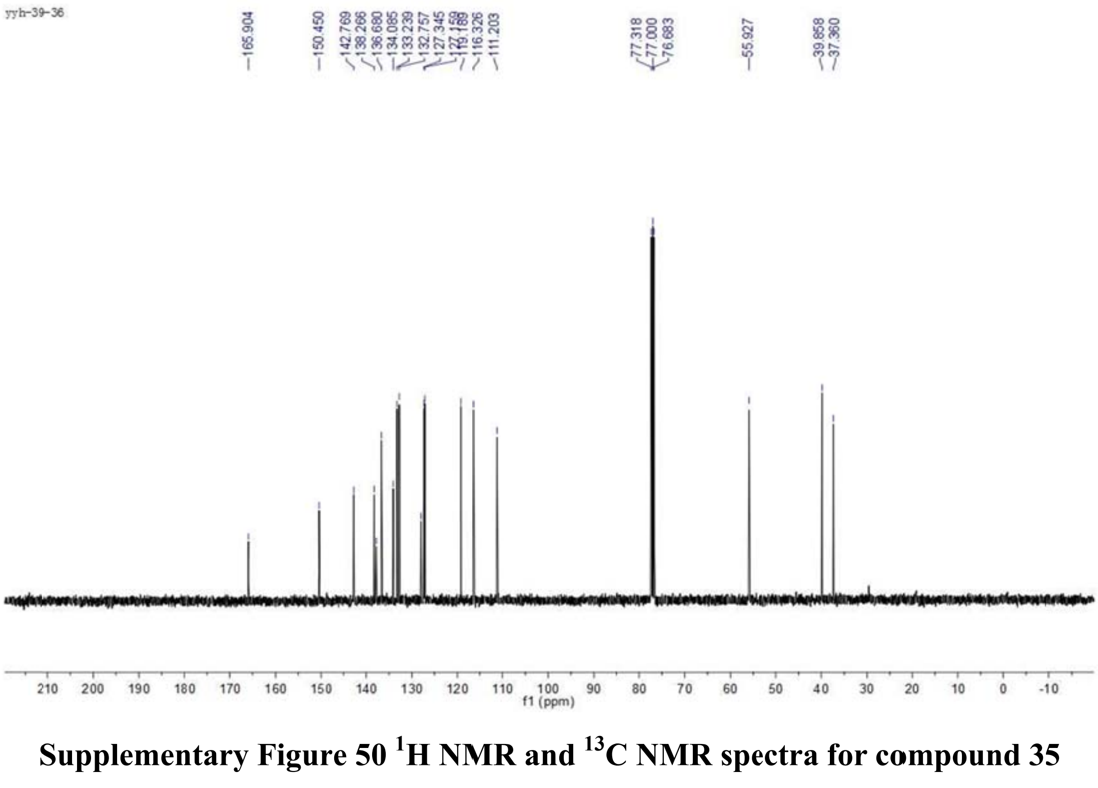

Supplementary Figure 50 ^{1}H NMR and ^{13}C NMR spectra for compound 35

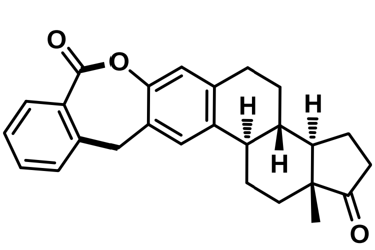

36

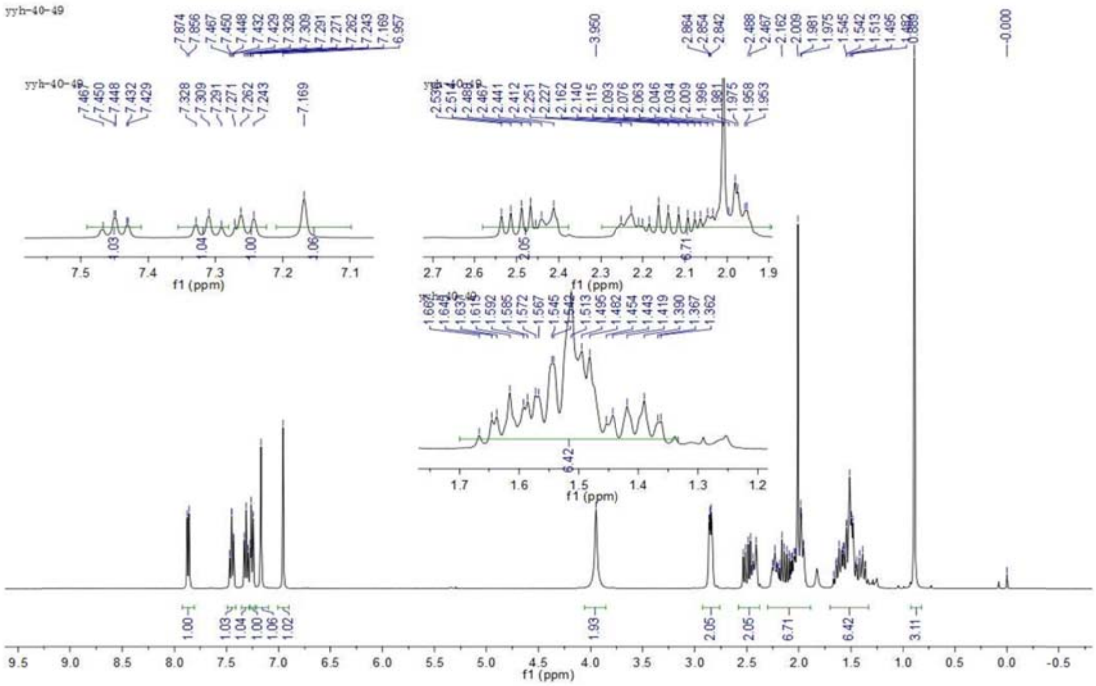

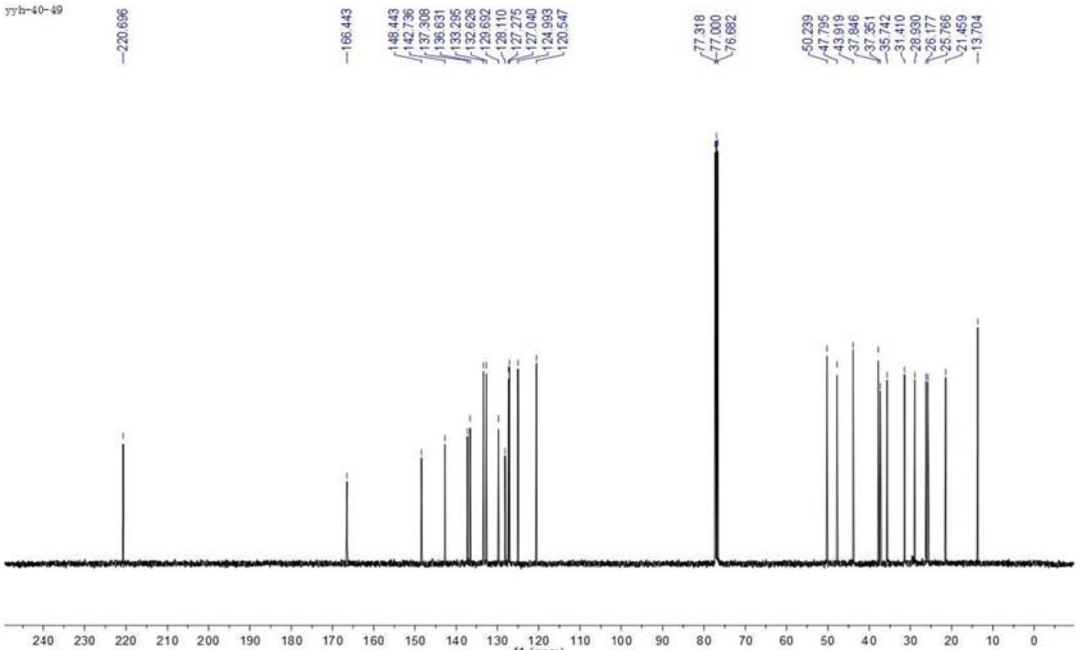

Supplementary Figure 51 ^{1}H NMR and ^{13}C NMR spectra for compound 36

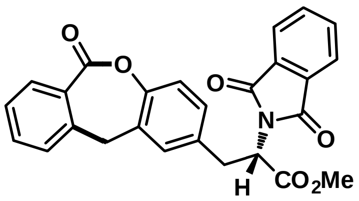

37

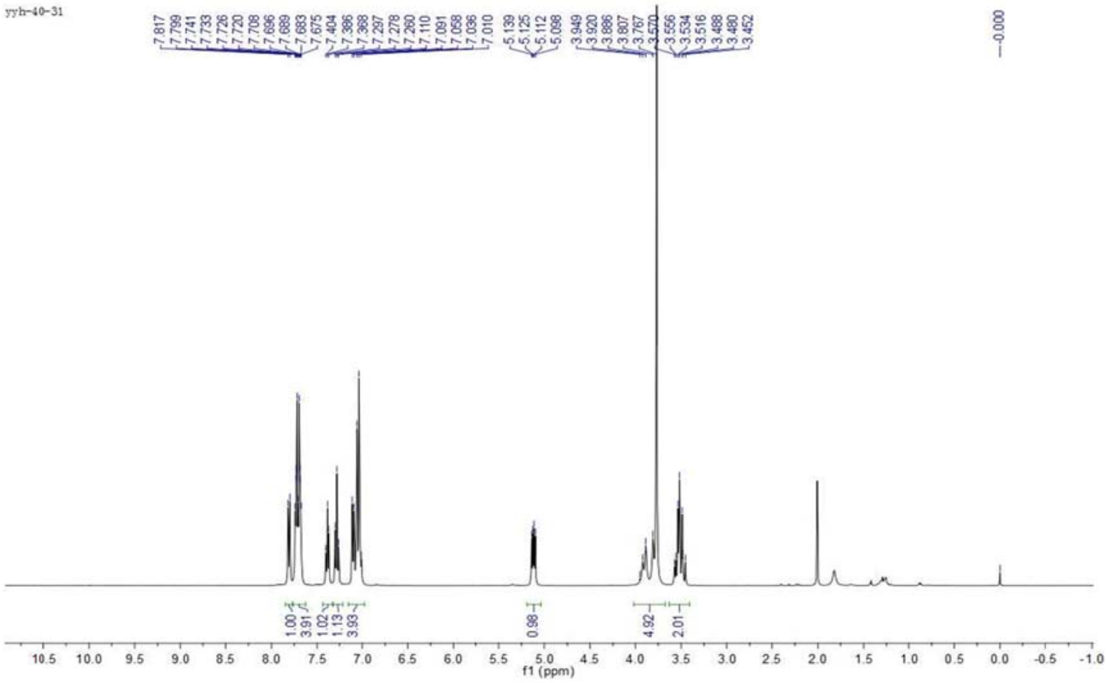

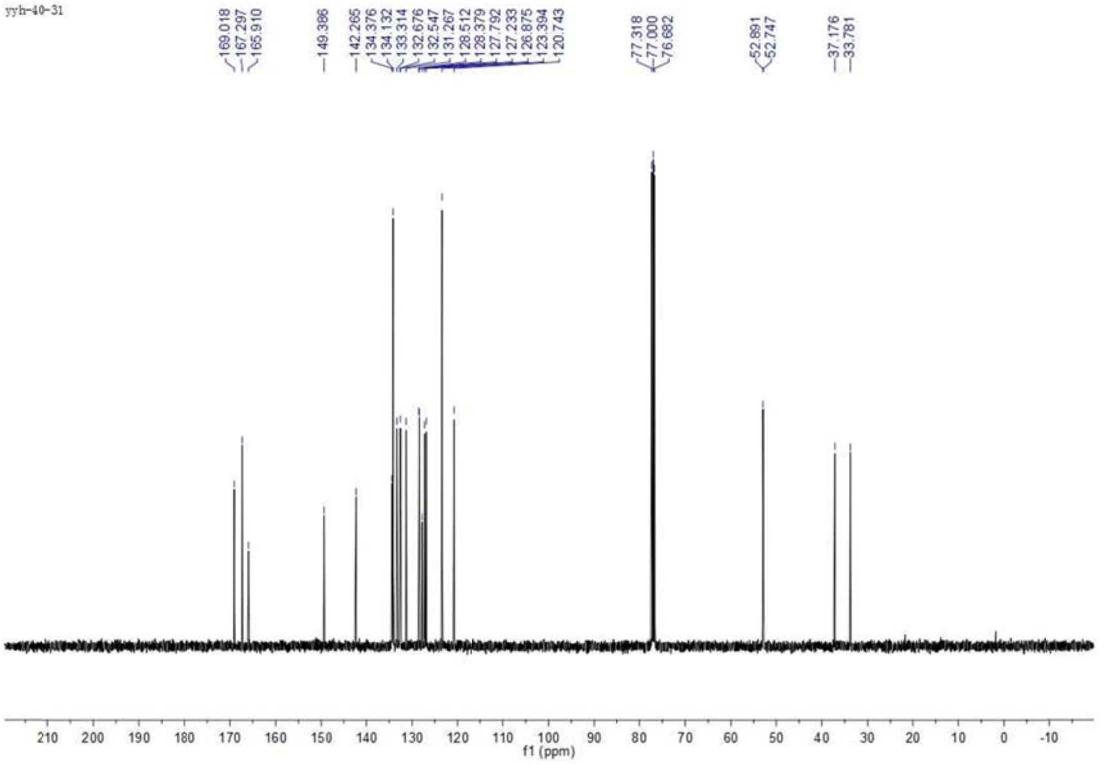

Supplementary Figure 52 ^{1}H NMR and ^{13}C NMR spectra for compound 37

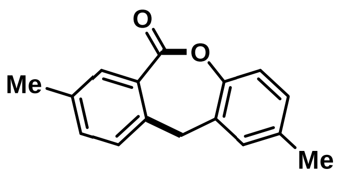

38

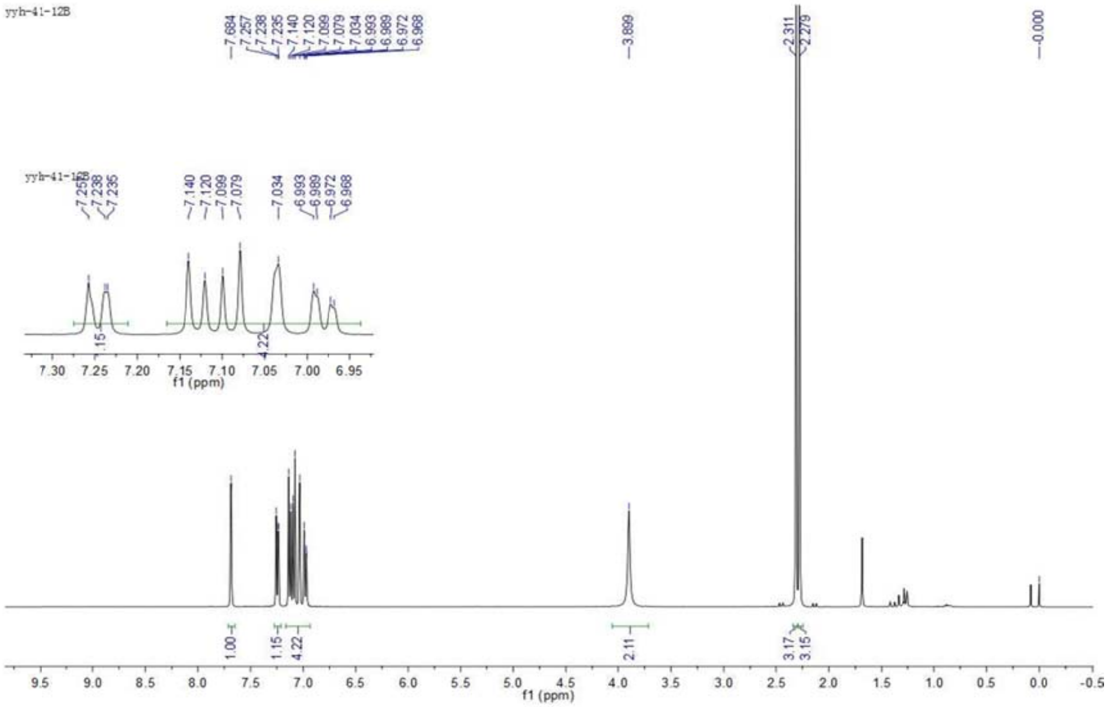

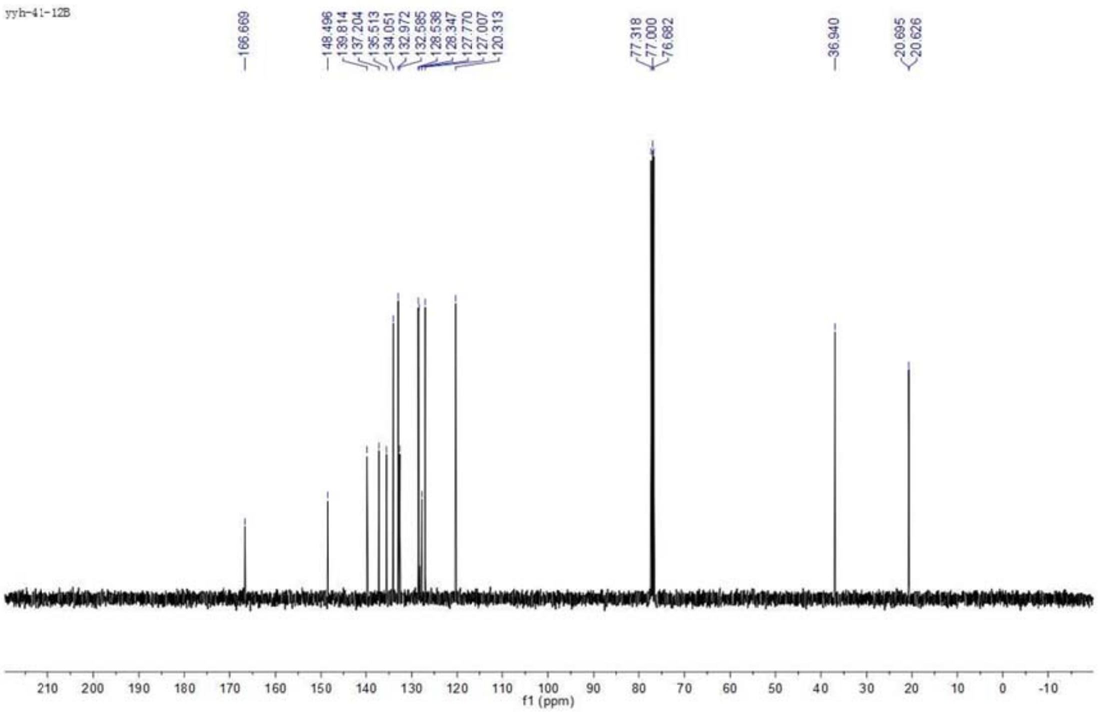

Supplementary Figure 53 ^{1}H NMR and ^{13}C NMR spectra for compound 38

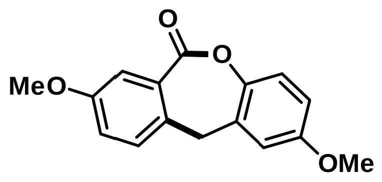

39

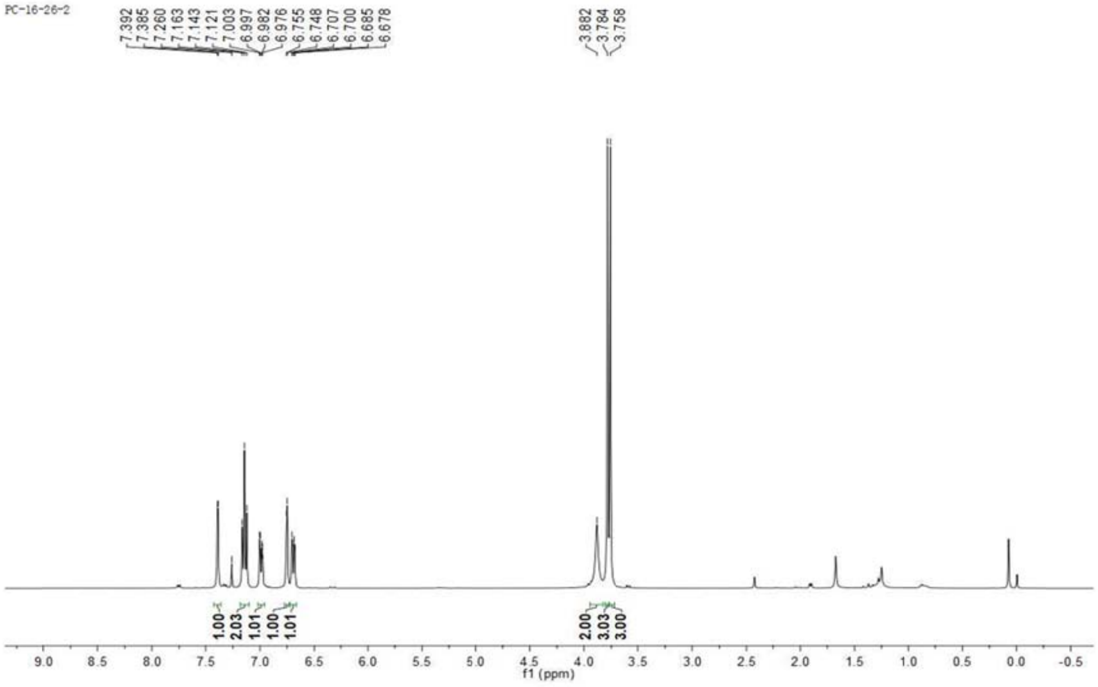

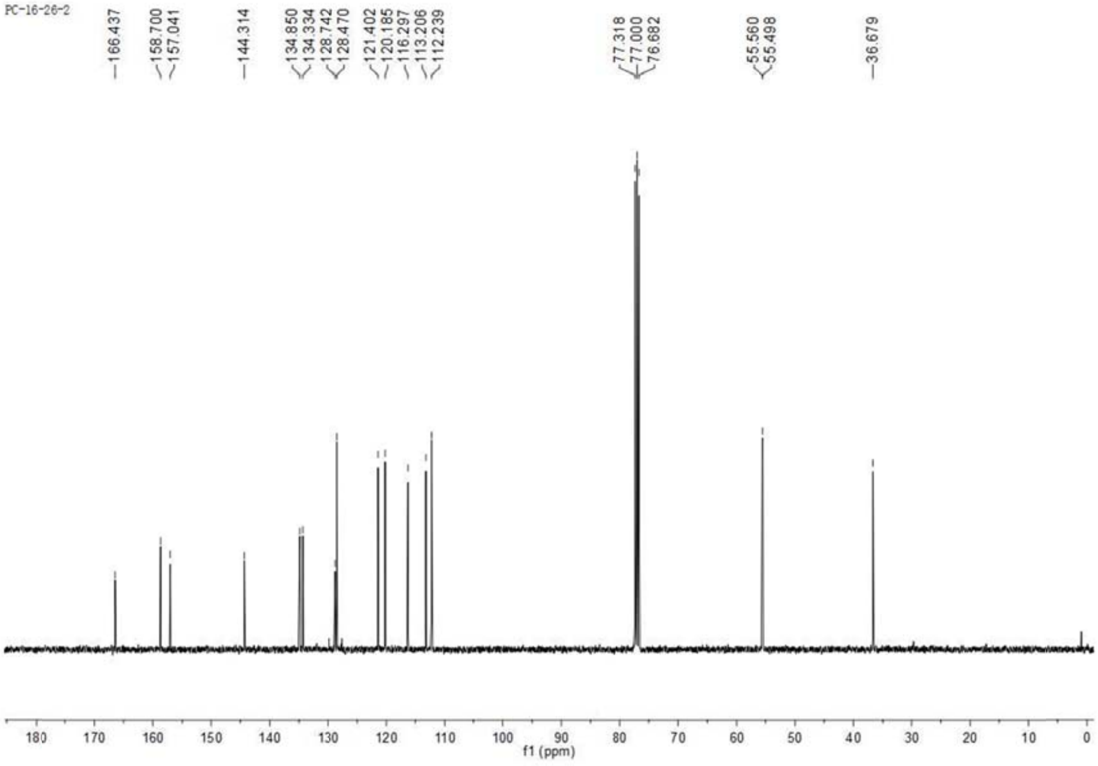

Supplementary Figure 54 ^{1}H NMR and ^{13}C NMR spectra for compound 39

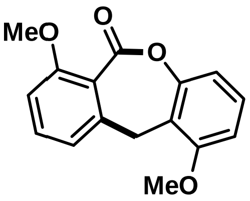

40

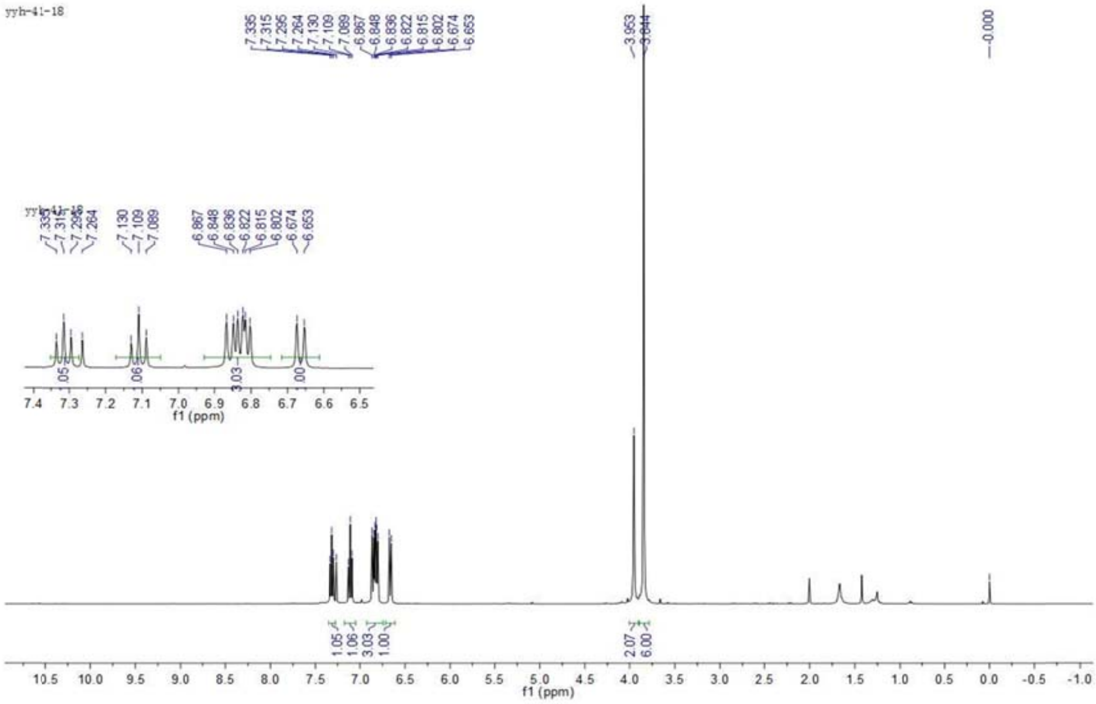

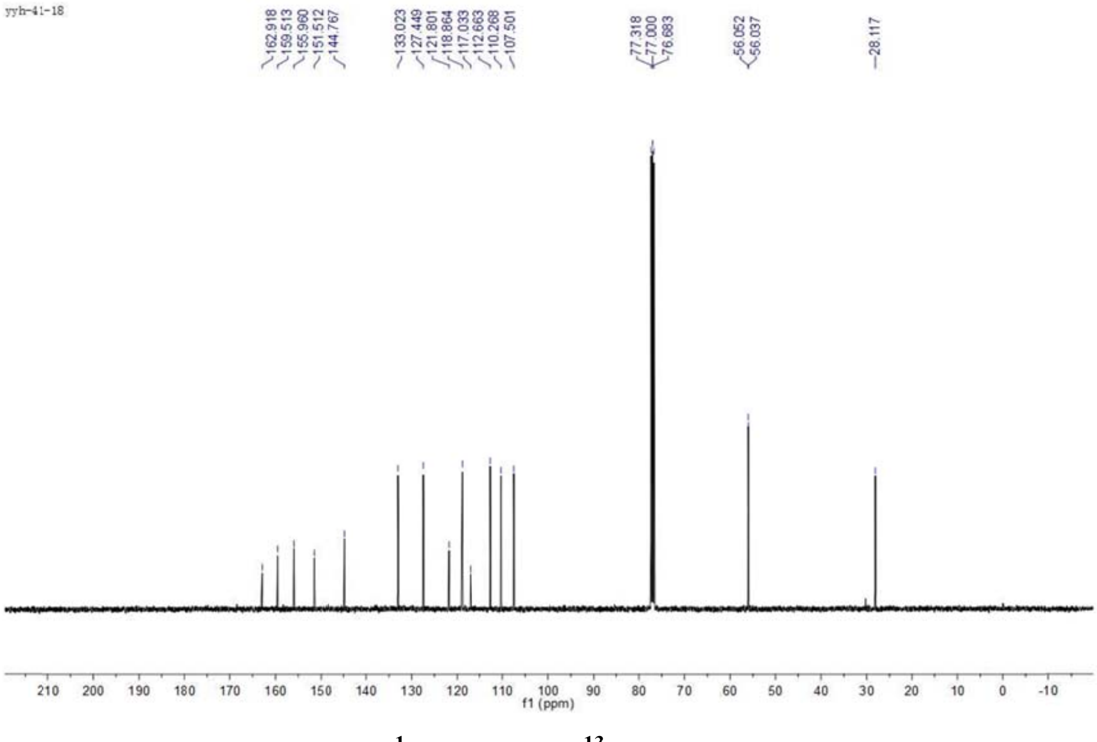

Supplementary Figure 55 ^{1}H NMR and ^{13}C NMR spectra for compound 40

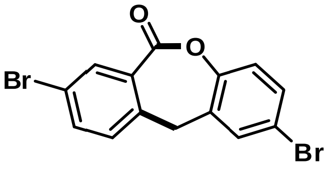

41

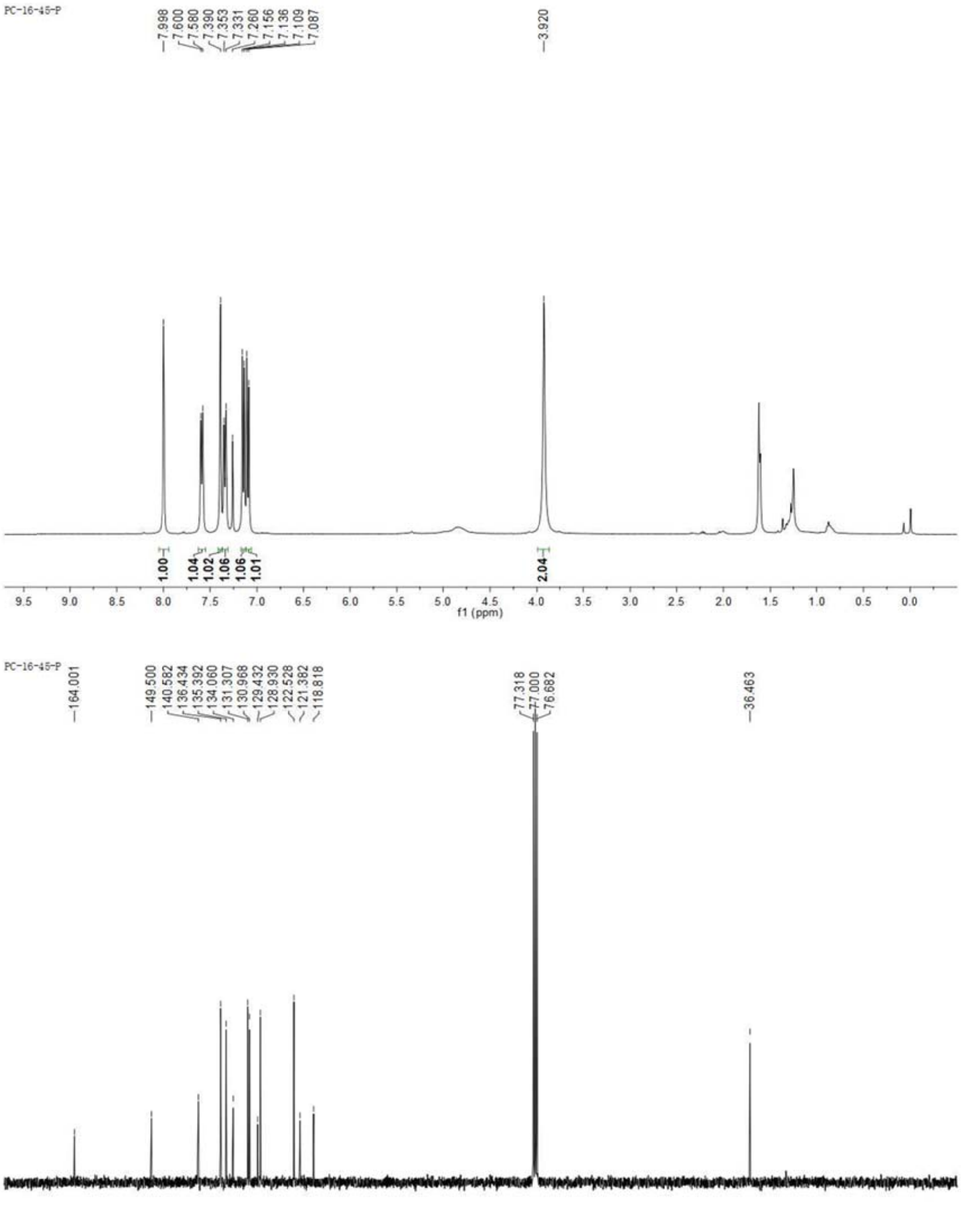

Supplementary Figure 56 ^{1}H NMR and ^{13}C NMR spectra for compound 41

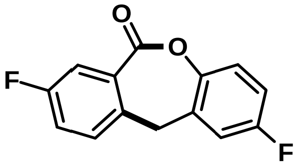
O
O
F
F

42

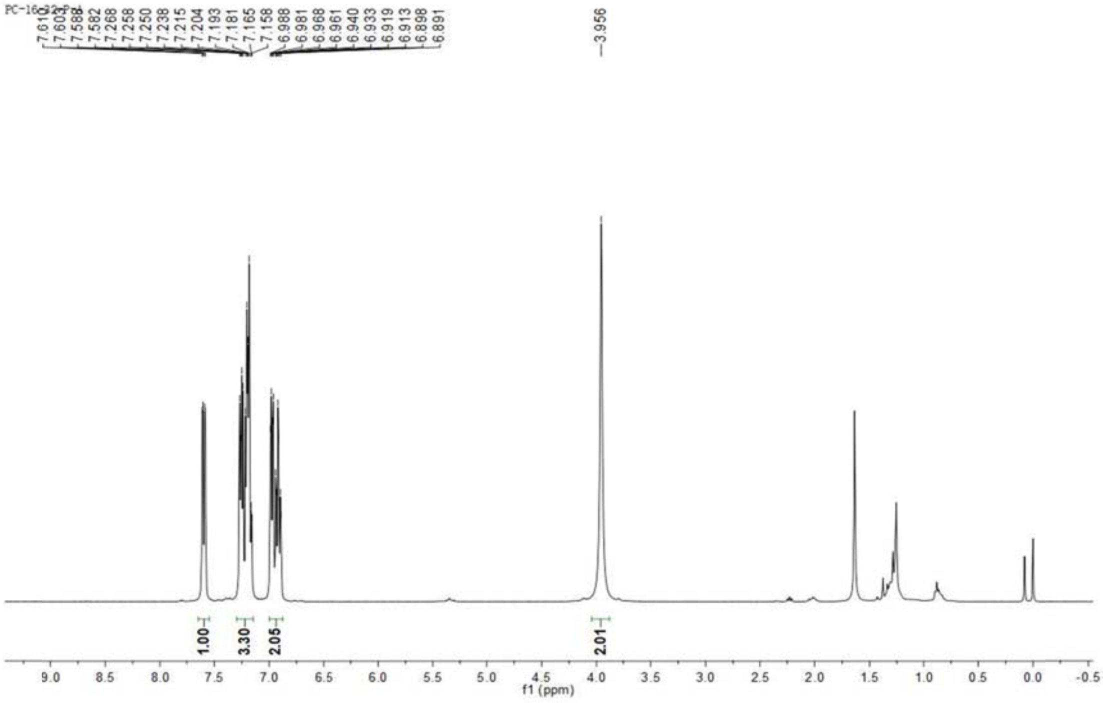
7.603
7.588
7.582
7.268
7.258
7.250
7.238
7.215
7.204
7.193
7.181
7.165
7.158
6.988
6.981
6.968
6.961
6.940
6.933
6.919
6.913
6.898
6.891
3.956
1.00
3.30
2.05
2.01
9.0 8.5 8.0 7.5 7.0 6.5 6.0 5.5 5.0 4.5 4.0 3.5 3.0 2.5 2.0 1.5 1.0 0.5 0.0 -0.5
f1 (ppm)

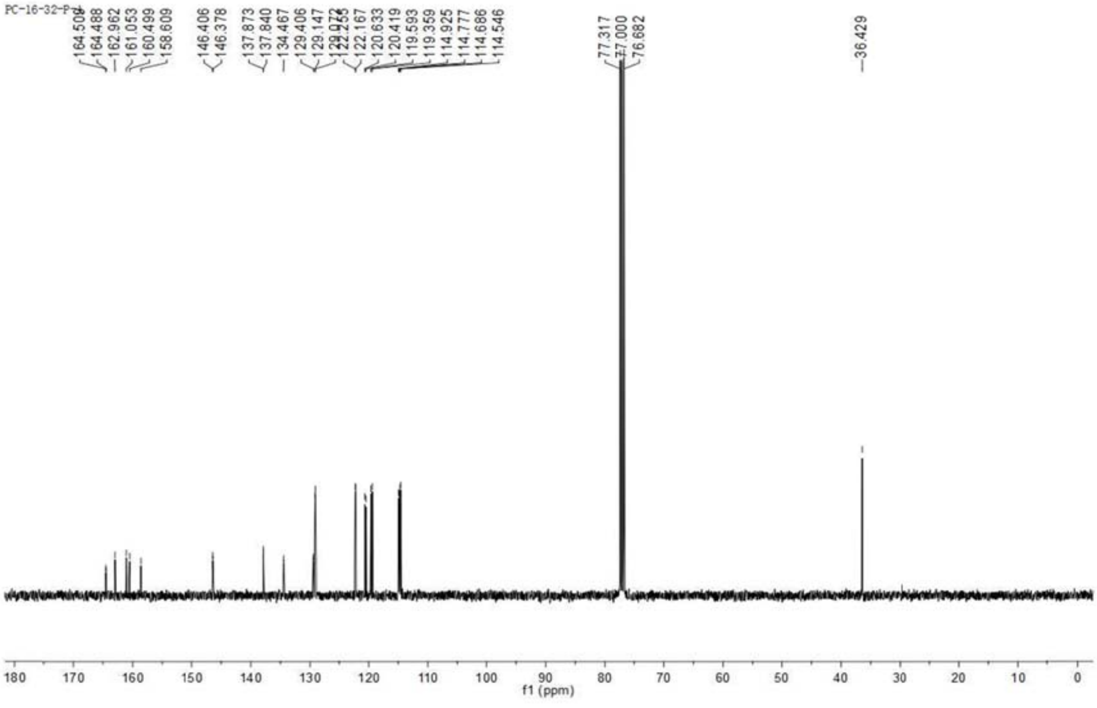
164.488
162.962
161.053
160.499
158.609
146.406
146.378
137.873
137.840
134.467
129.406
129.147
122.167
120.633
120.419
119.593
119.359
114.925
114.777
114.686
114.546
77.317
77.000
76.682
36.429
180 170 160 150 140 130 120 110 100 90 80 70 60 50 40 30 20 10 0
f1 (ppm)

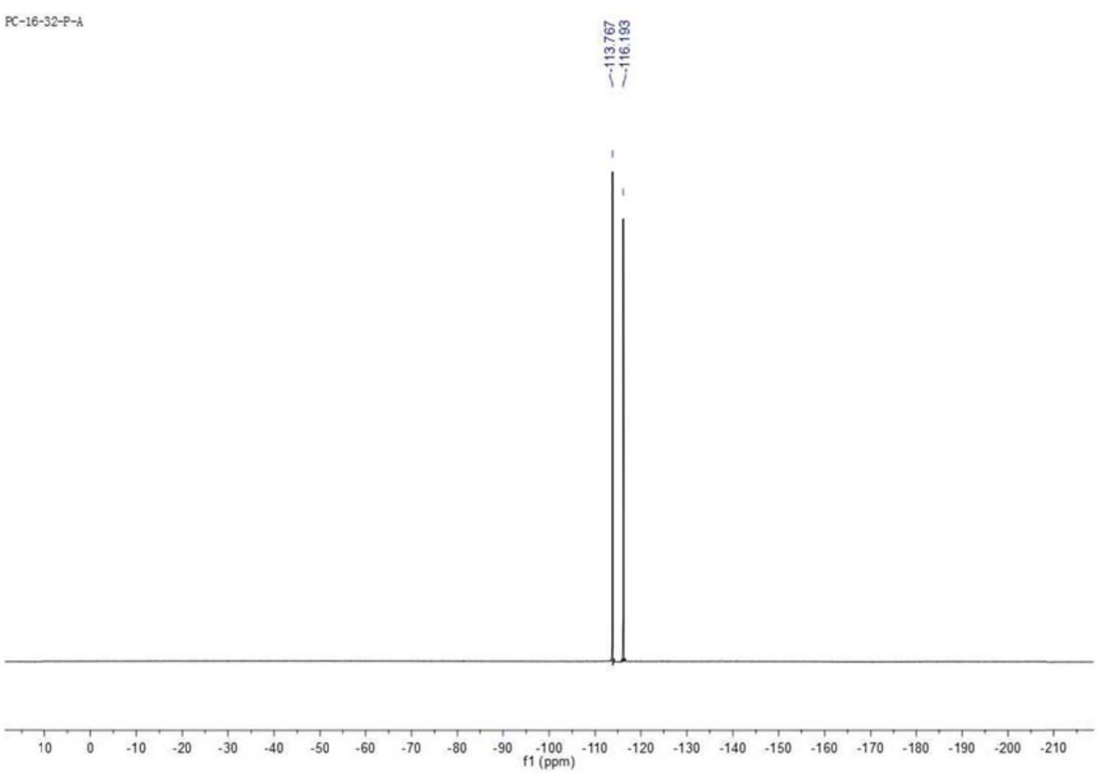

Supplementary Figure 57 ^{1}H NMR, ^{13}C NMR and ^{19}F NMR spectra for compound 42

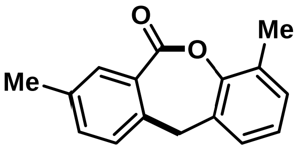

43

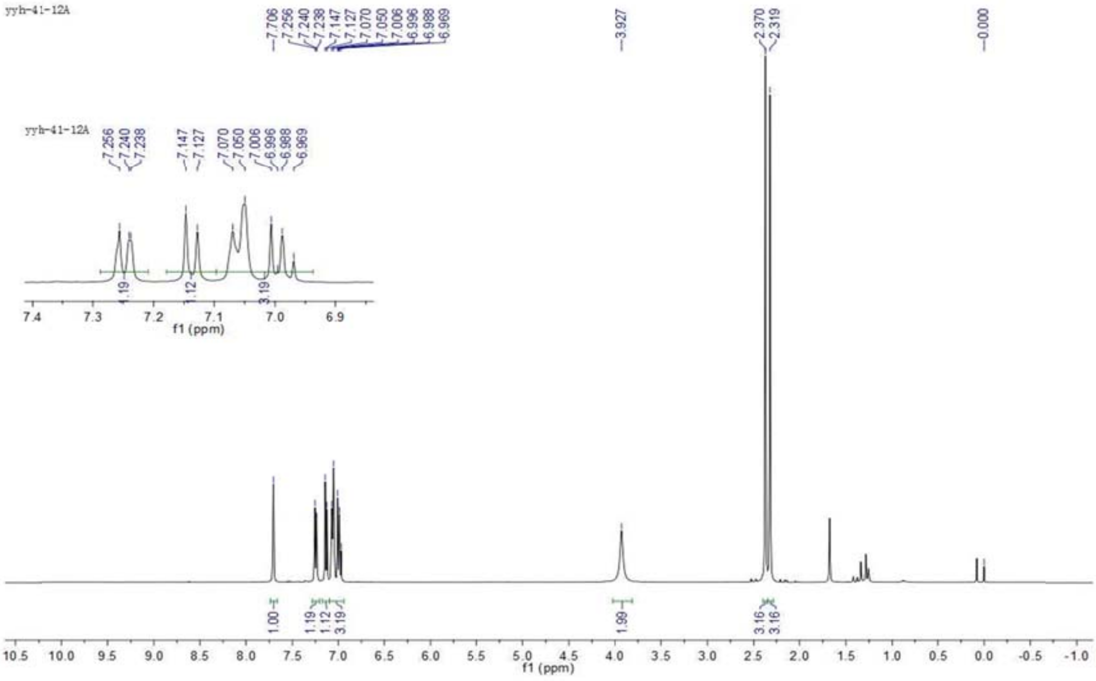

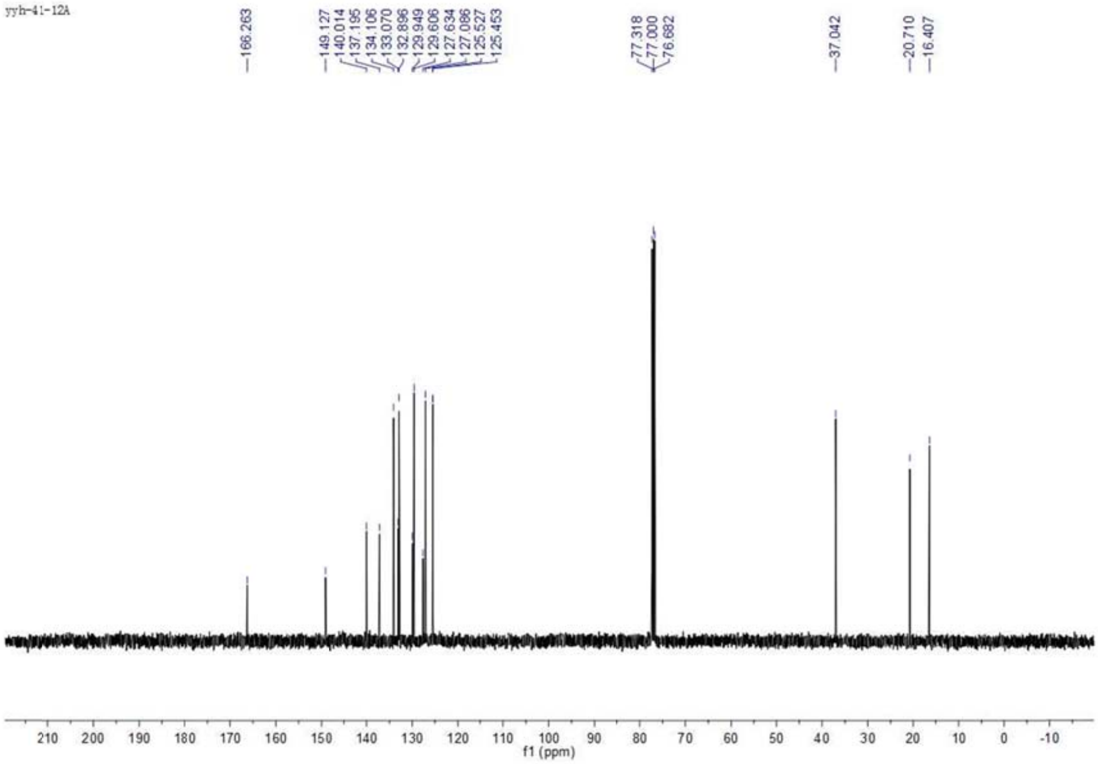

Supplementary Figure 58 ^{1}H NMR and ^{13}C NMR spectra for compound 43

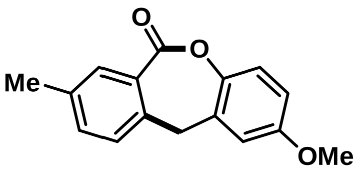

44

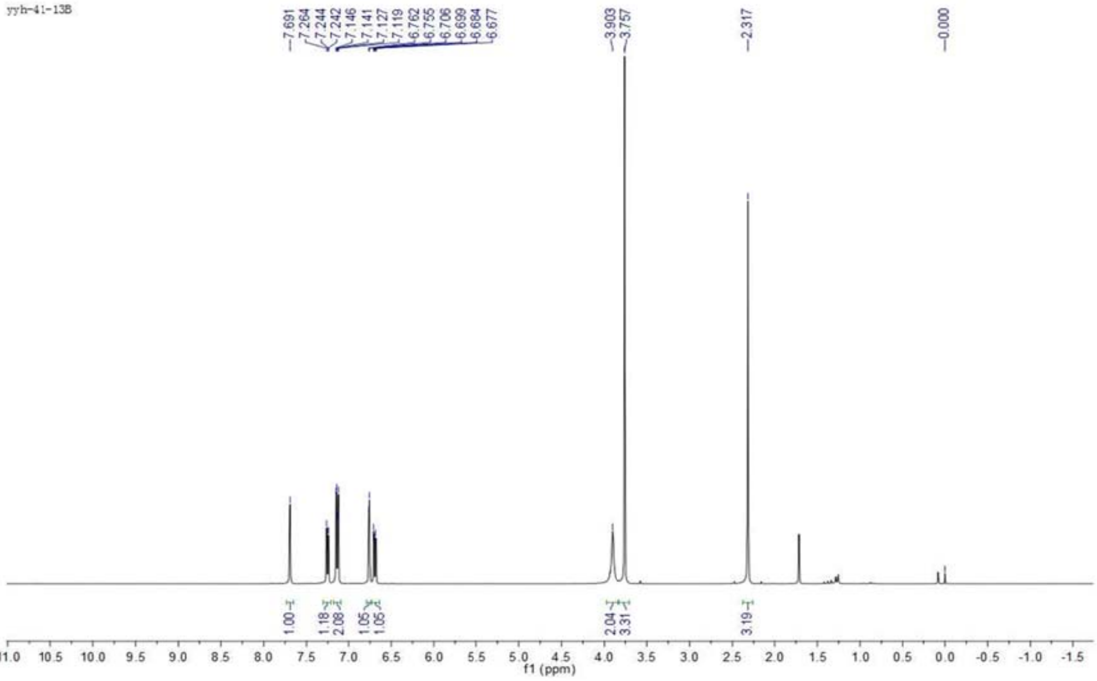

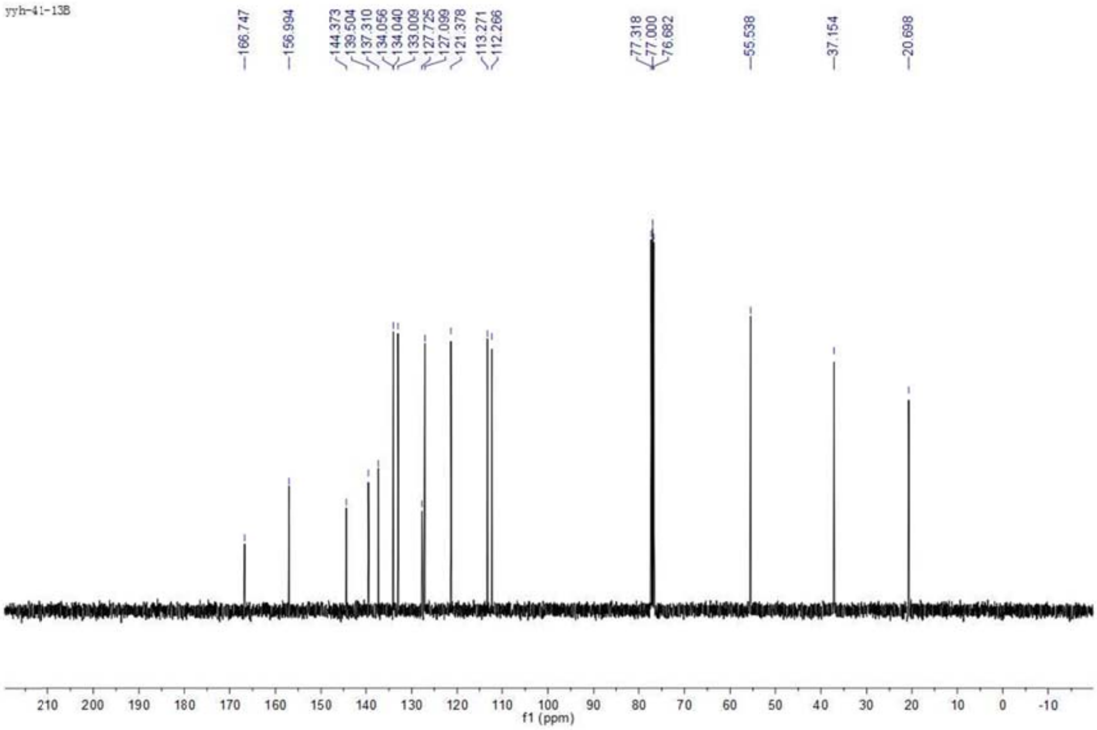

Supplementary Figure 59 ^{1}H NMR and ^{13}C NMR spectra for compound 44

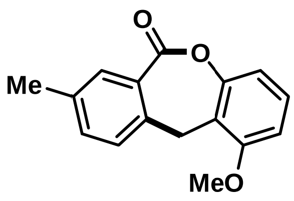

45

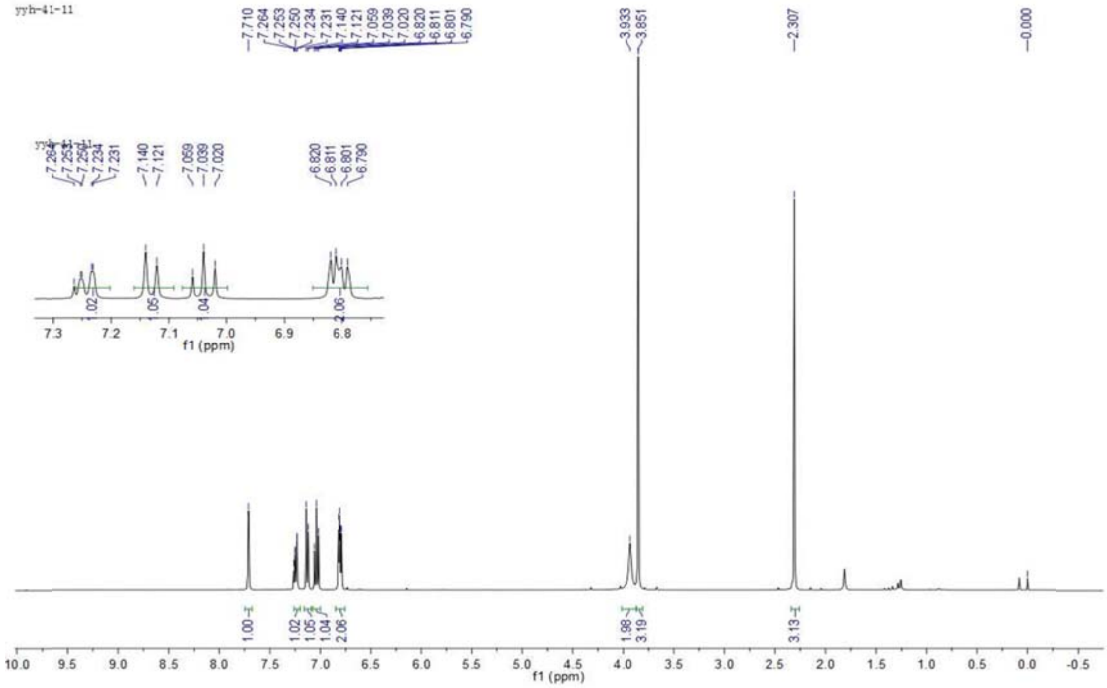

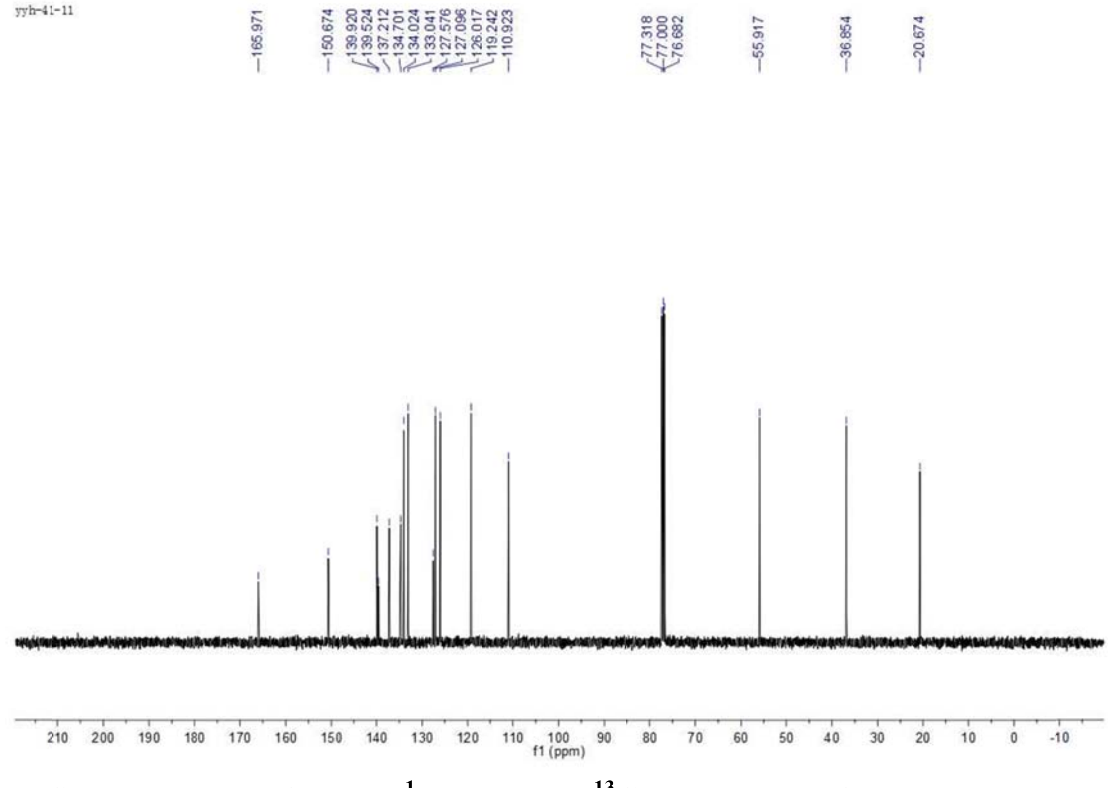

Supplementary Figure 60 ^{1}H NMR and ^{13}C NMR spectra for compound 45

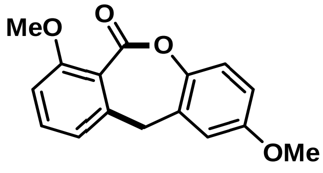

46

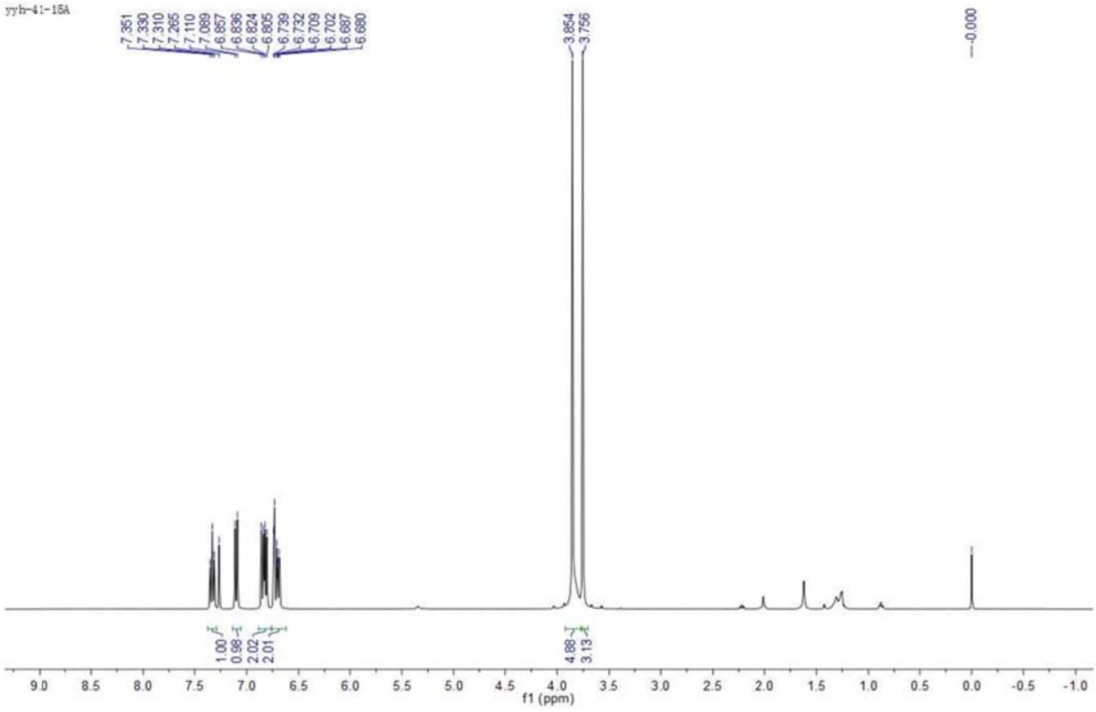

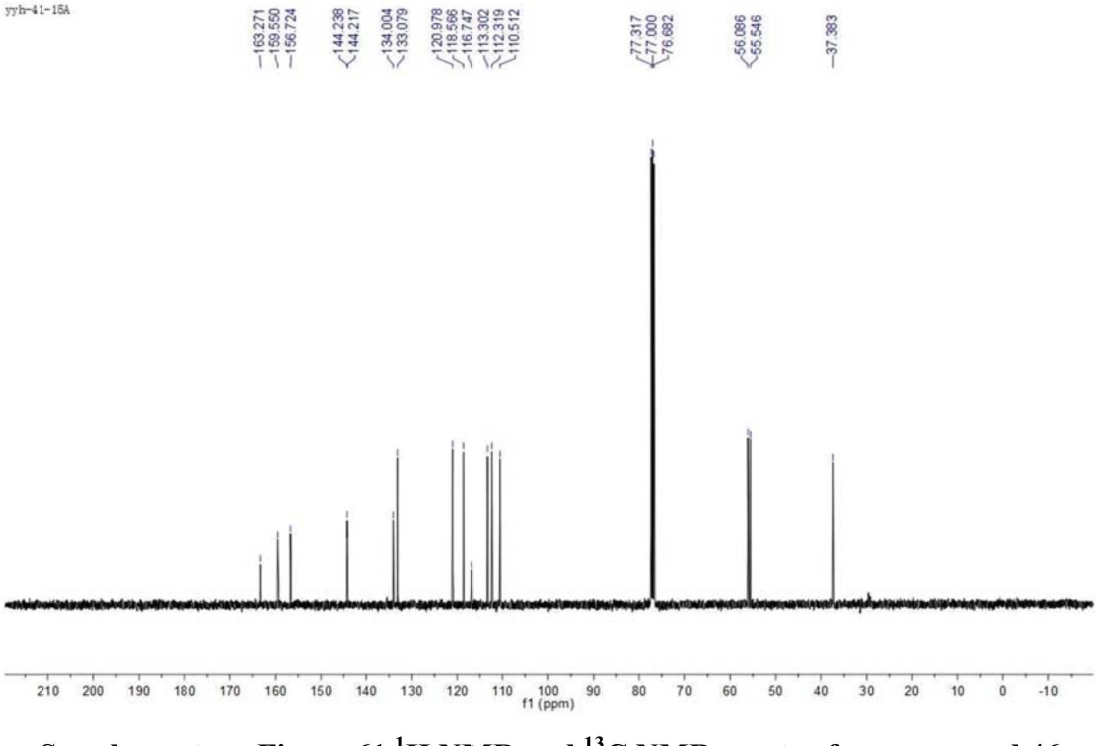

Supplementary Figure 61 ^{1}H NMR and ^{13}C NMR spectra for compound 46

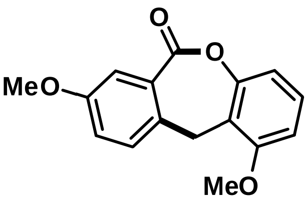

47

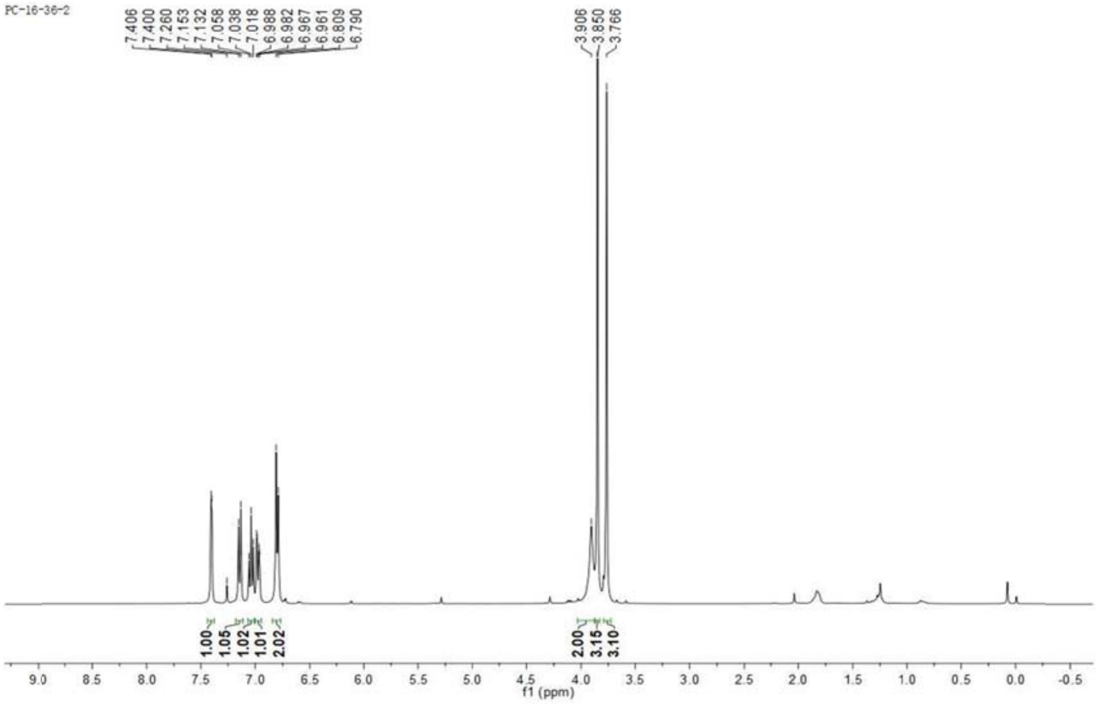

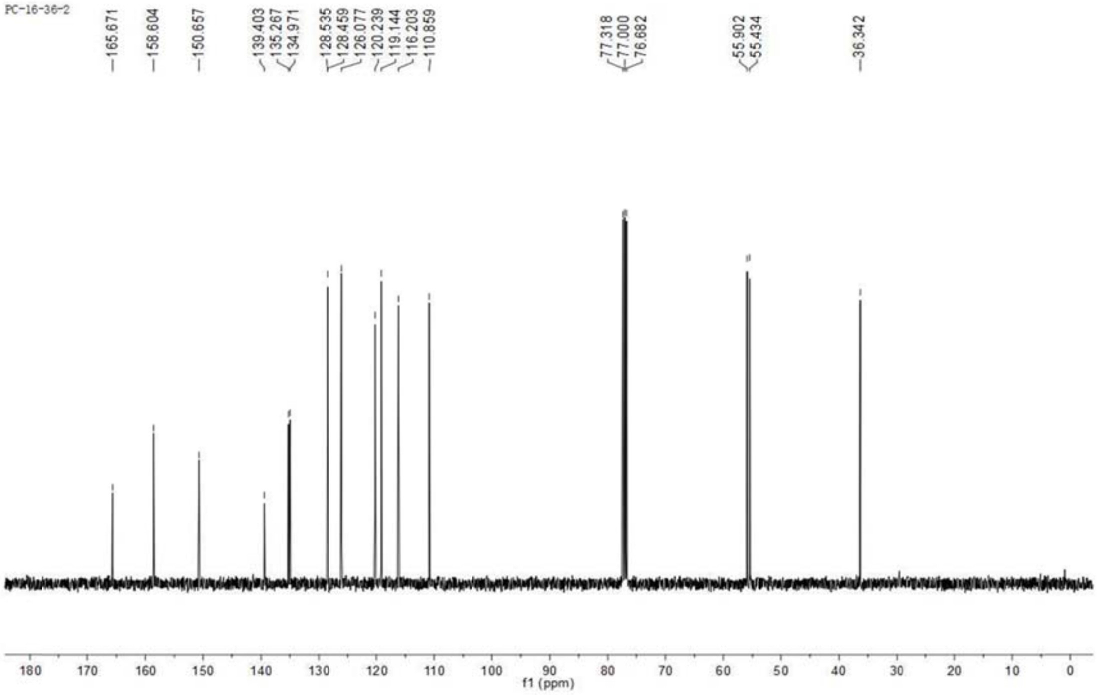

Supplementary Figure 62 ^{1}H NMR and ^{13}C NMR spectra for compound 47

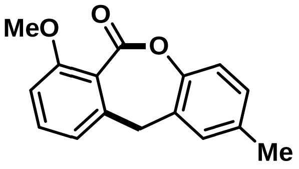

48

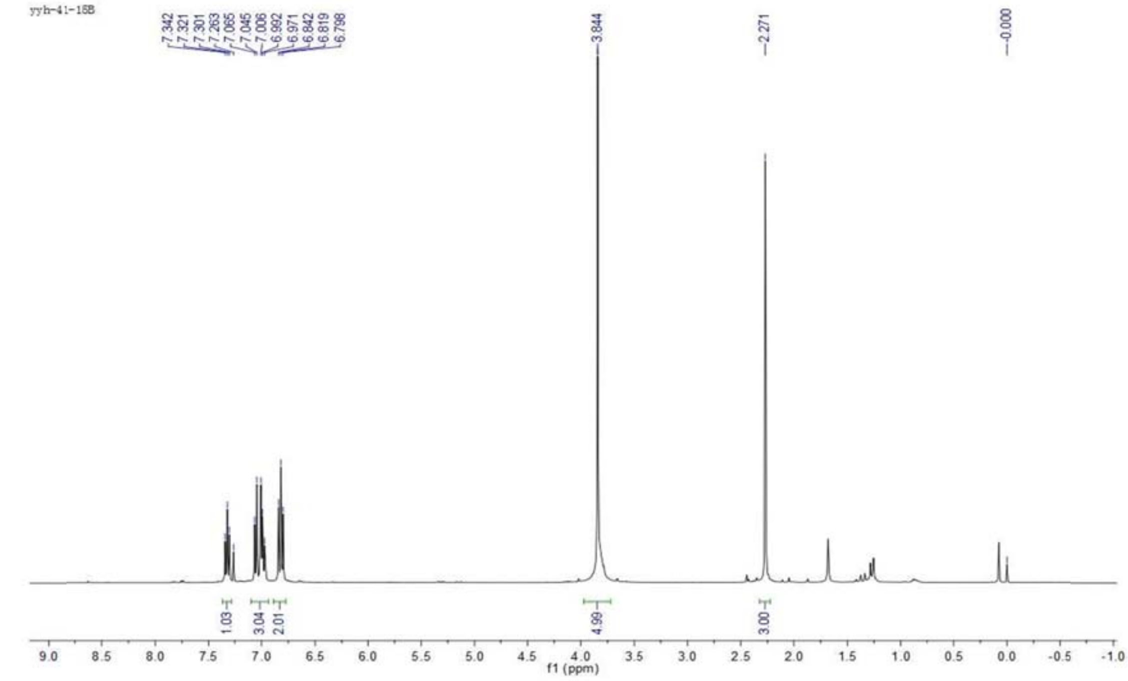

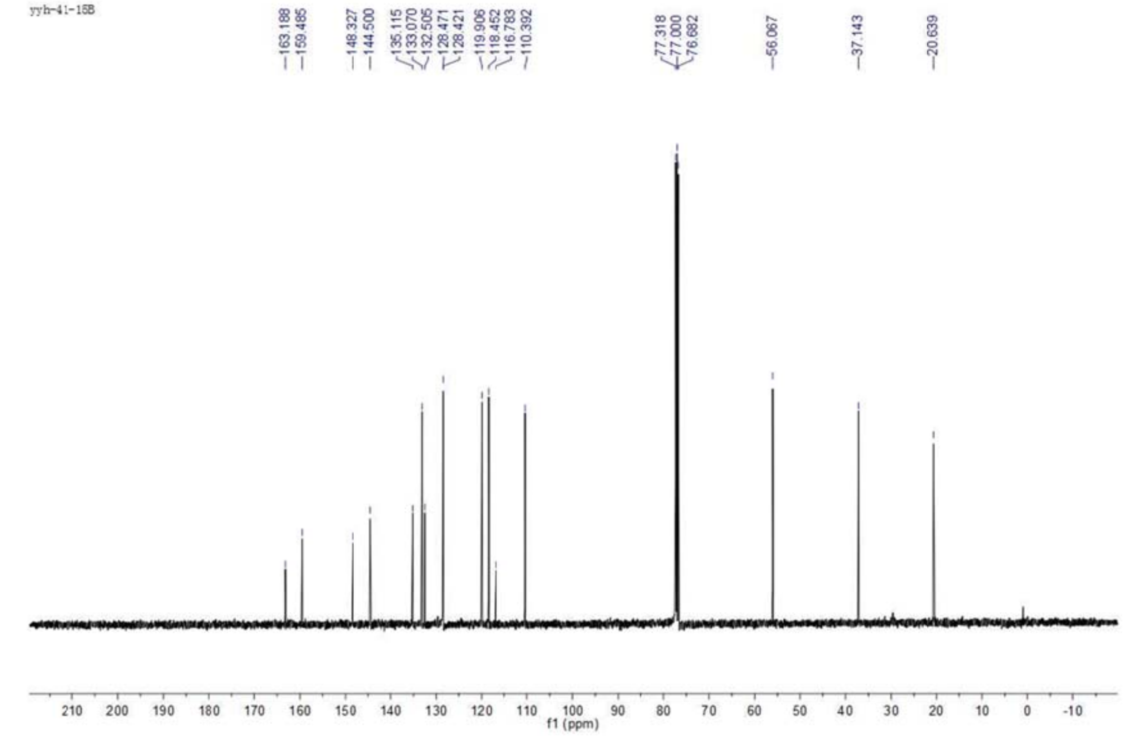

Supplementary Figure 63 ^{1}H NMR and ^{13}C NMR spectra for compound 48

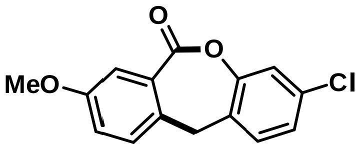

49

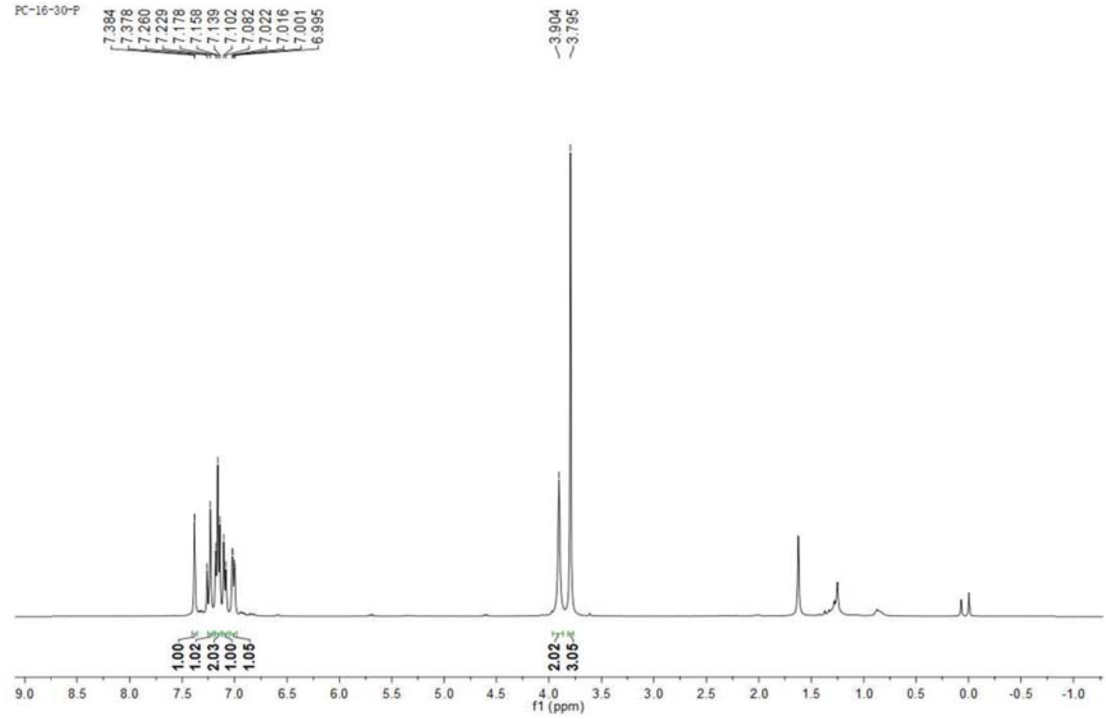

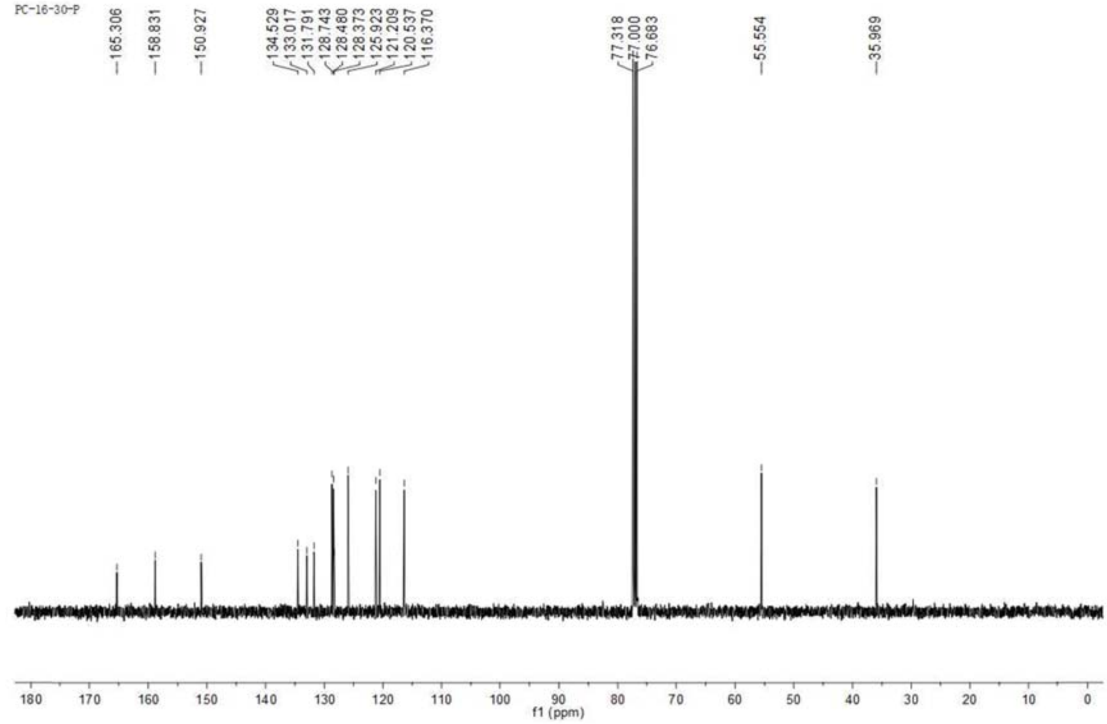

Supplementary Figure 64 ^{1}H NMR and ^{13}C NMR spectra for compound 49

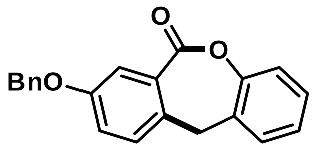

50

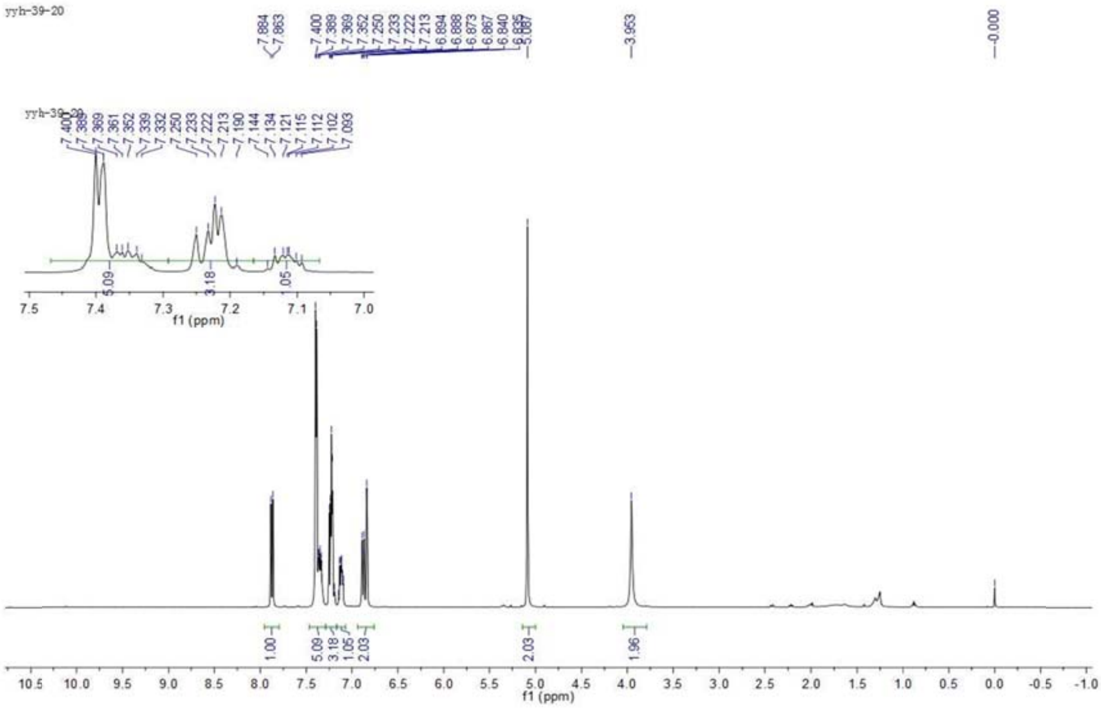

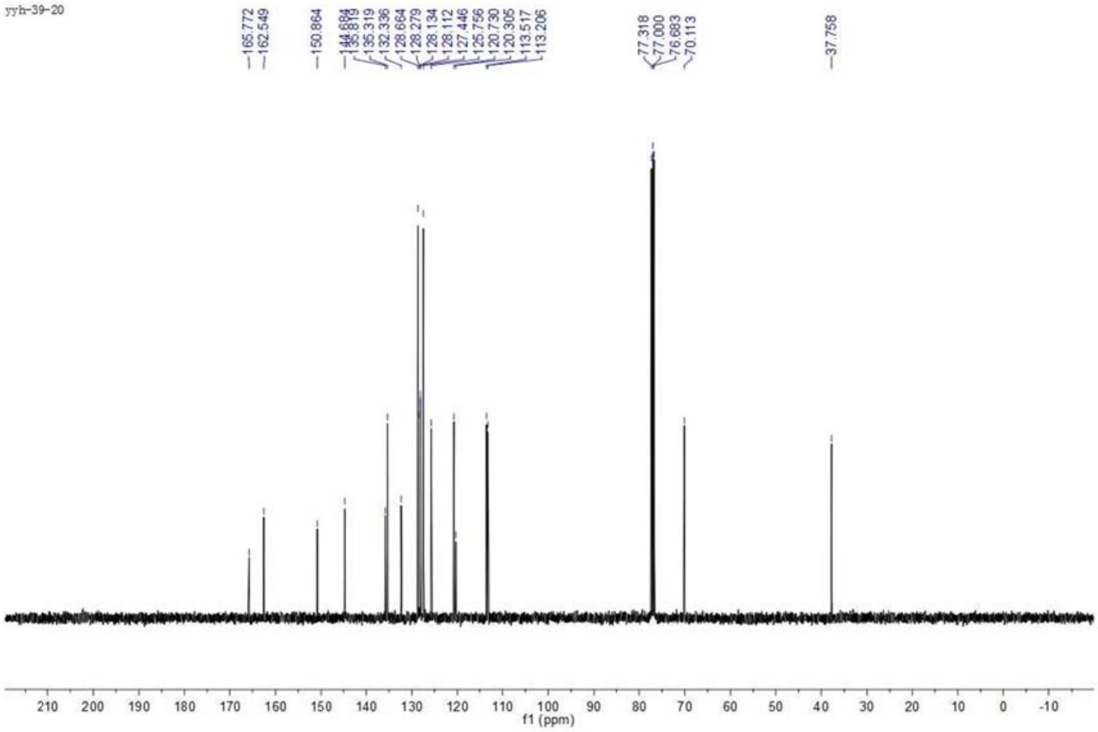

Supplementary Figure 65 ^{1}H NMR and ^{13}C NMR spectra for compound 50

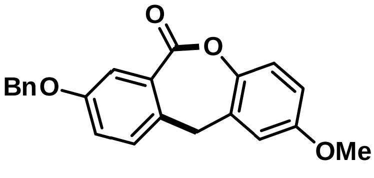

51

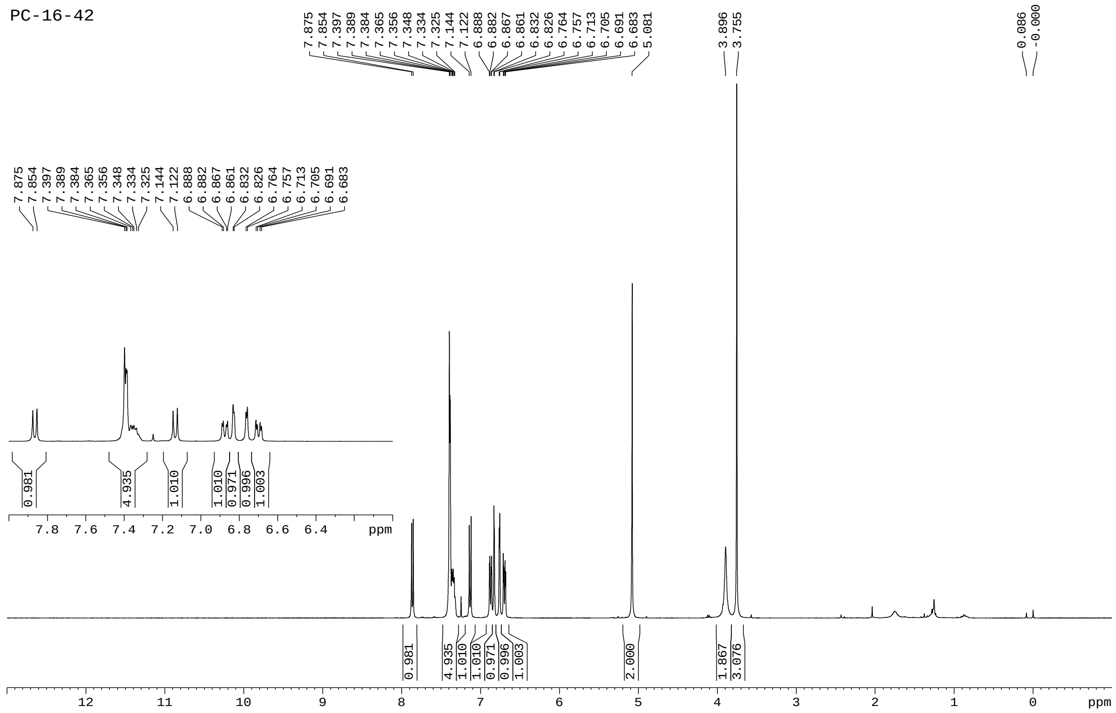

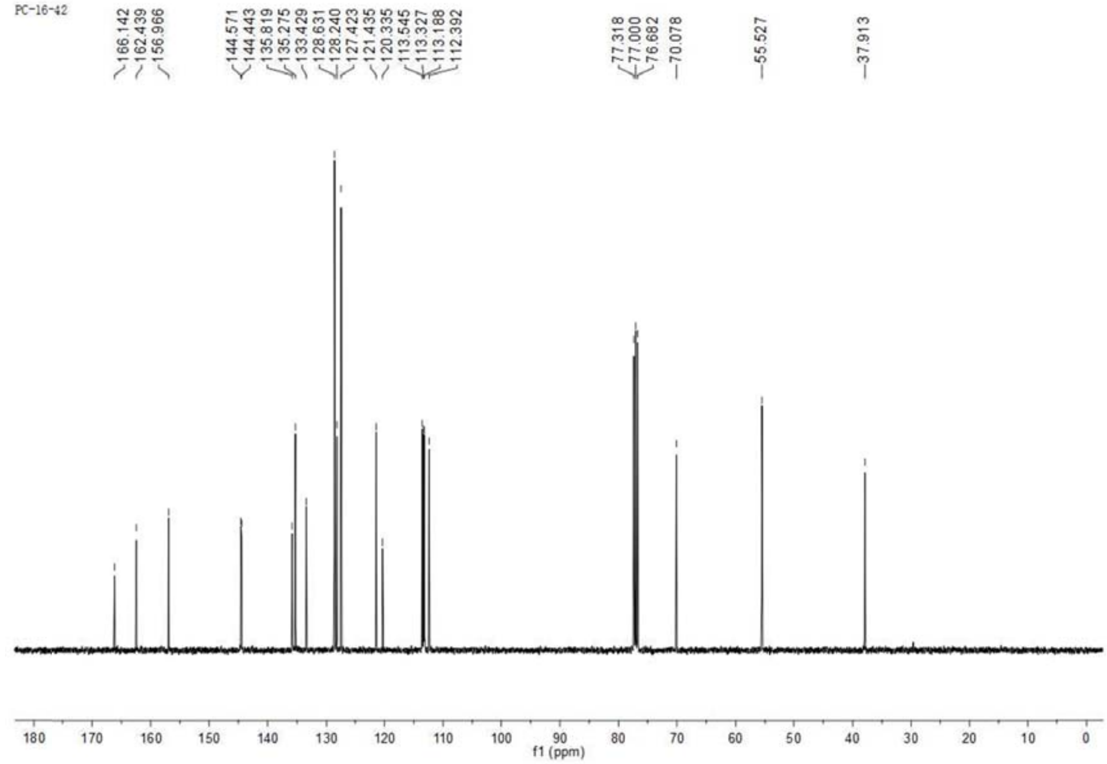

Supplementary Figure 66 ^{1}H NMR and ^{13}C NMR spectra for compound 51

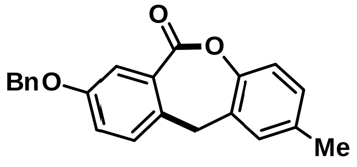

52

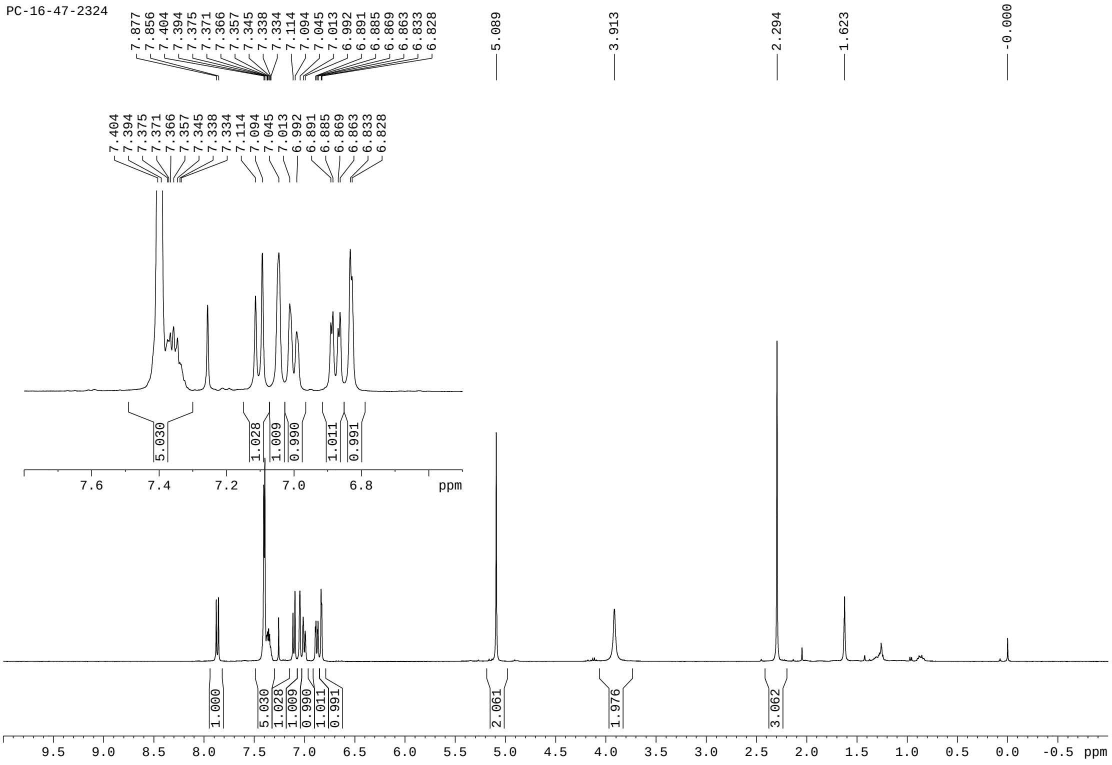

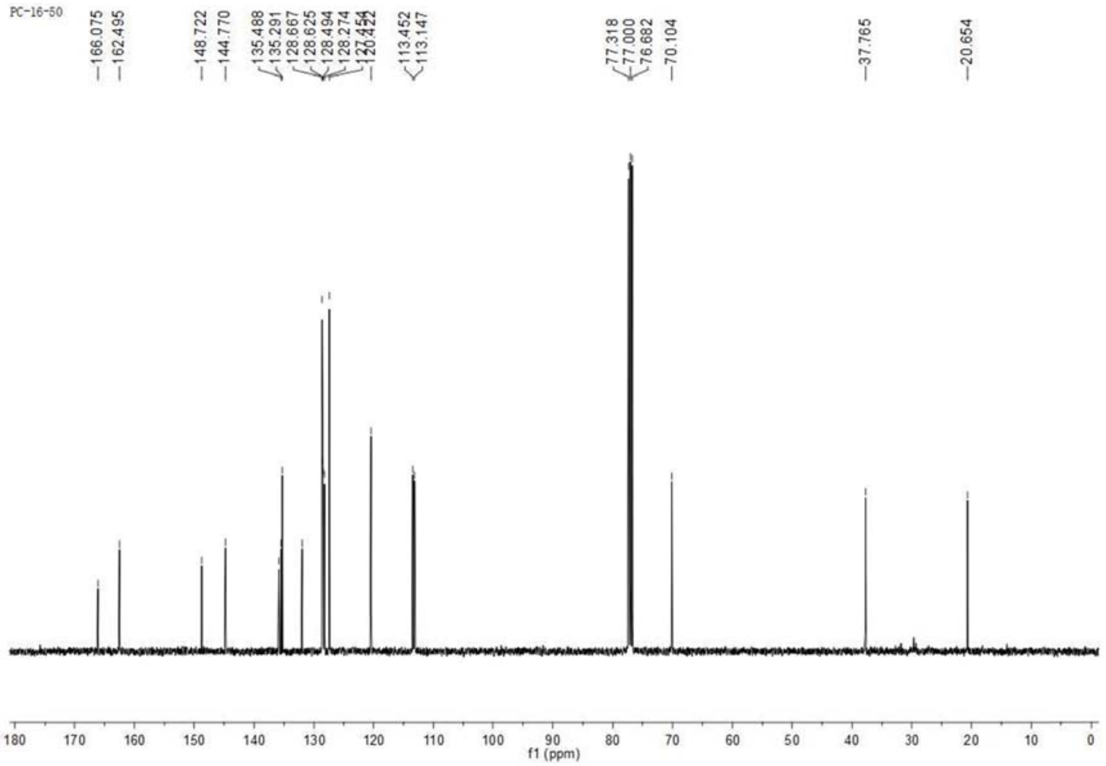

Supplementary Figure 67 ^{1}H NMR and ^{13}C NMR spectra for compound 52

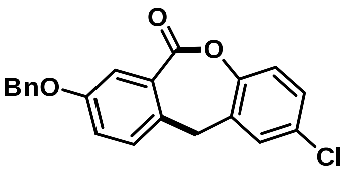

53

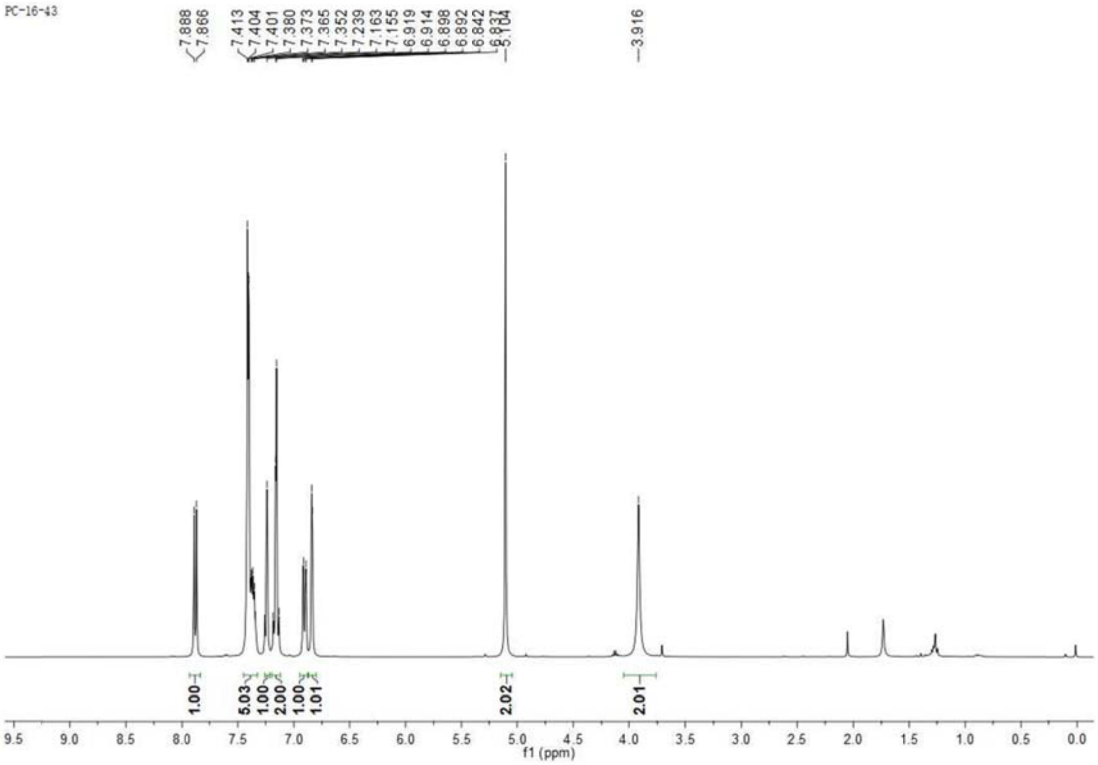

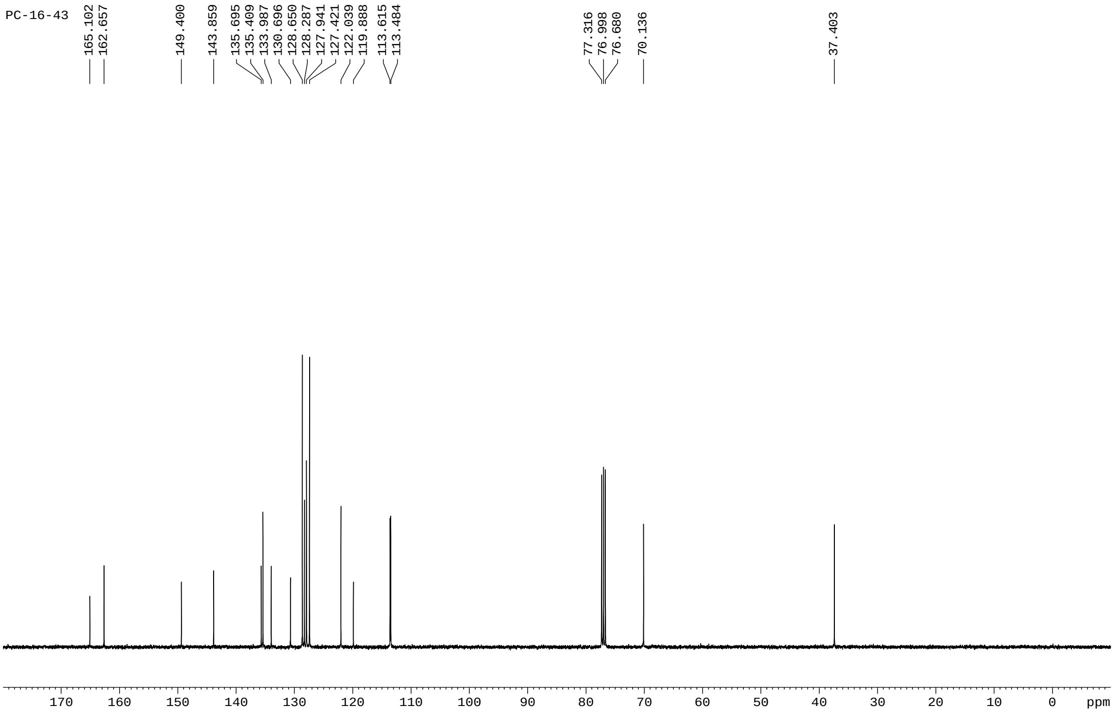

Supplementary Figure 68 ^{1}H NMR and ^{13}C NMR spectra for compound 53

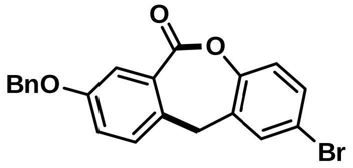

54

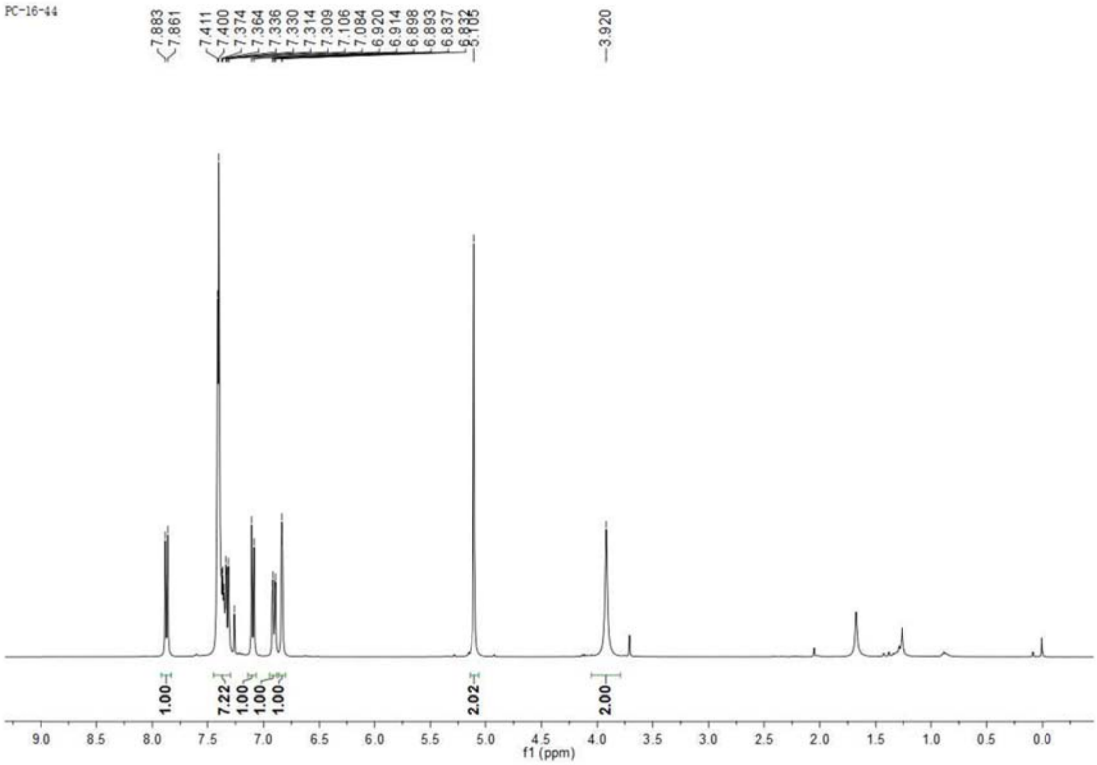

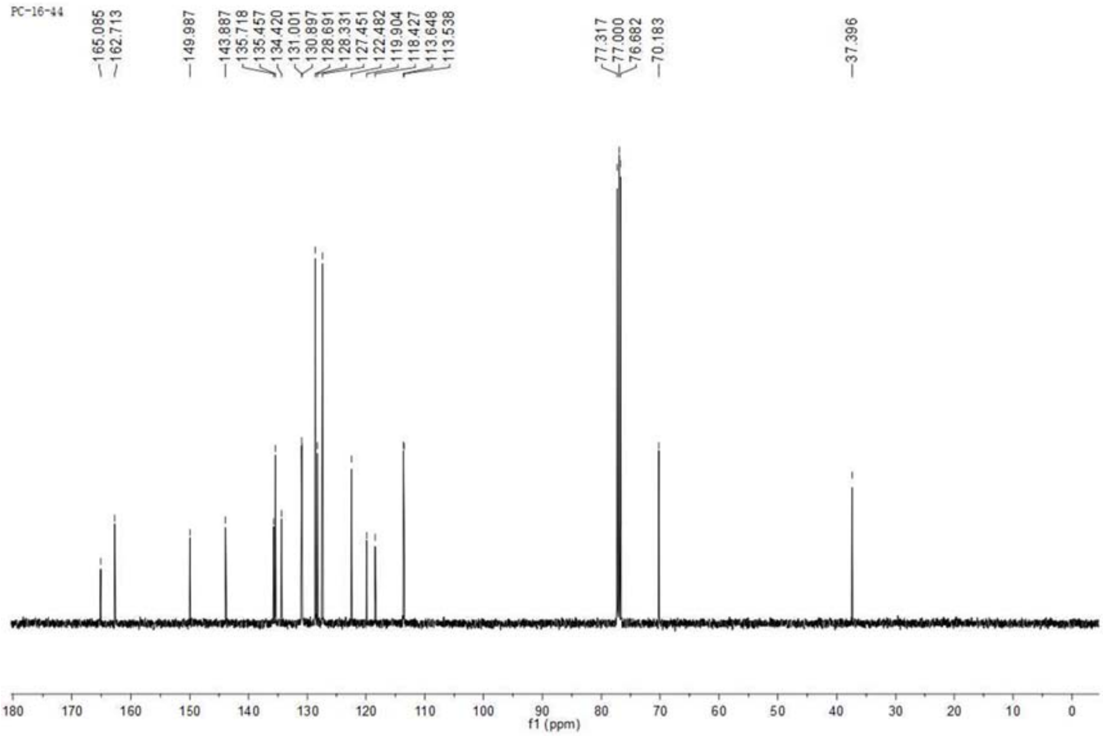

Supplementary Figure 69 ^{1}H NMR and ^{13}C NMR spectra for compound 54

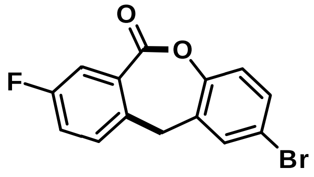
O
O
F
Br

55

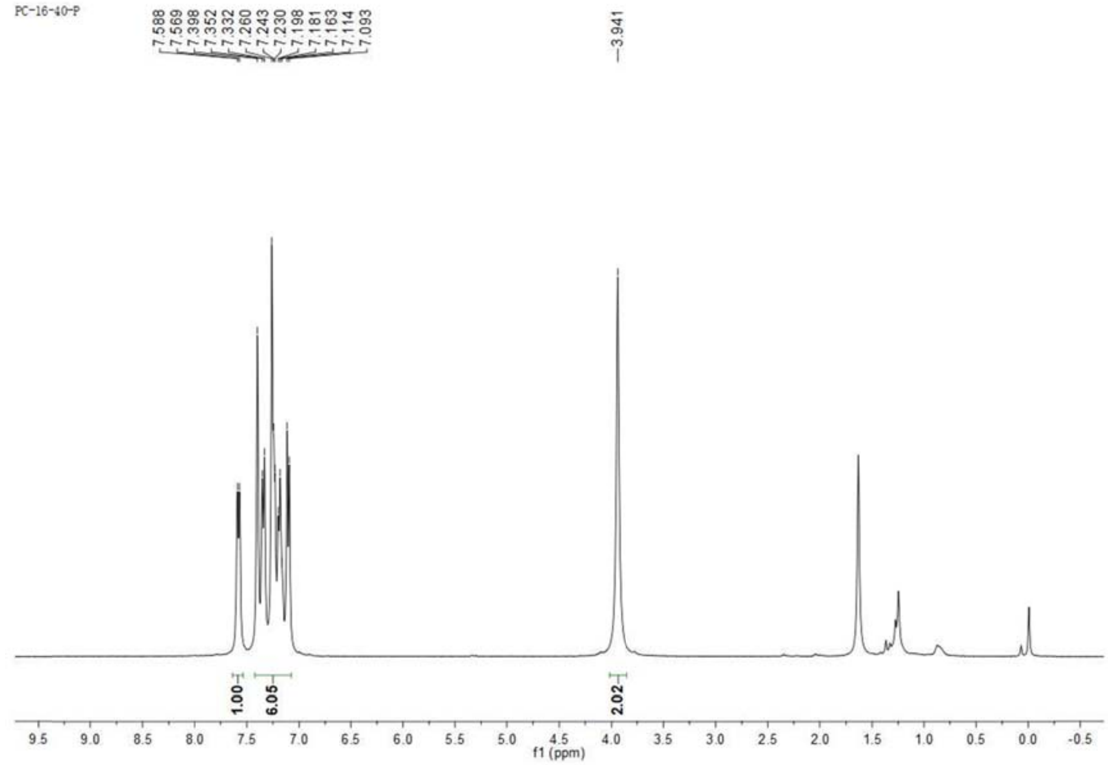
PC-16-40-P
7.588
7.569
7.398
7.352
7.332
7.260
7.243
7.230
7.198
7.181
7.163
7.114
7.093
3.941
1.00
6.05
2.02
9.5 9.0 8.5 8.0 7.5 7.0 6.5 6.0 5.5 5.0 4.5 4.0 3.5 3.0 2.5 2.0 1.5 1.0 0.5 0.0 -0.5
f1 (ppm)

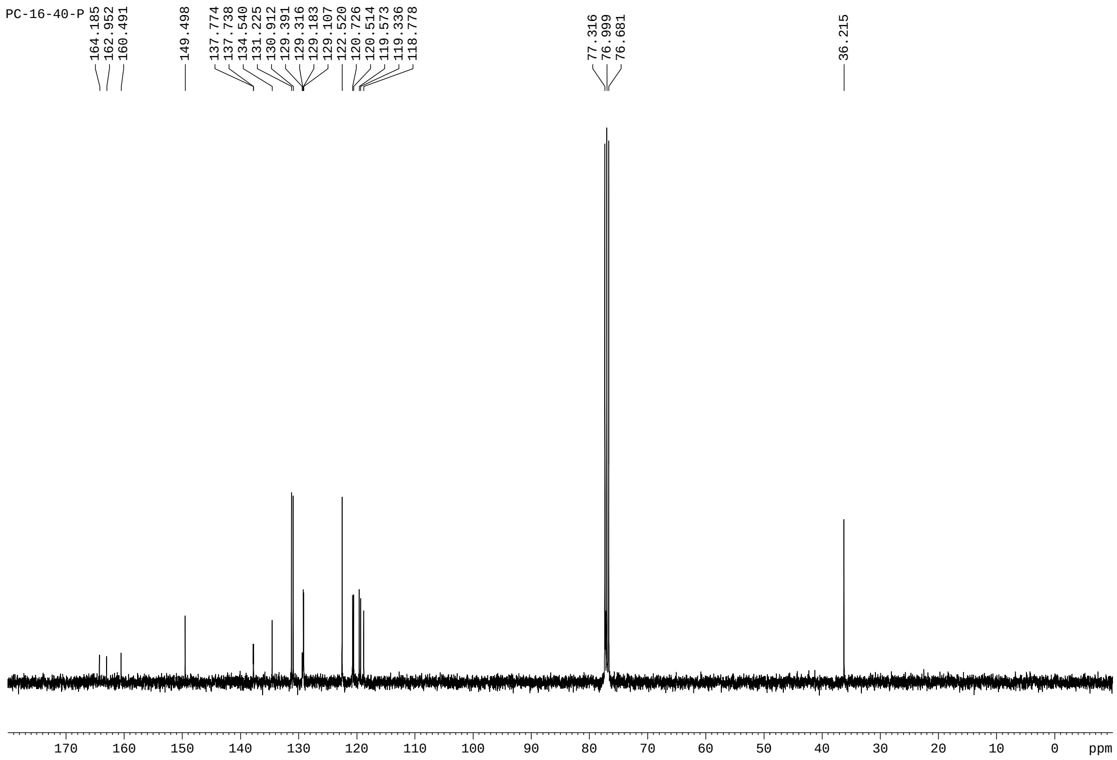
PC-16-40-P
164.185
162.952
160.491
149.498
137.774
137.738
134.540
131.225
130.912
129.391
129.316
129.183
129.107
122.520
120.726
120.514
119.573
119.336
118.778
77.316
76.999
76.681
36.215
170 160 150 140 130 120 110 100 90 80 70 60 50 40 30 20 10 0 ppm

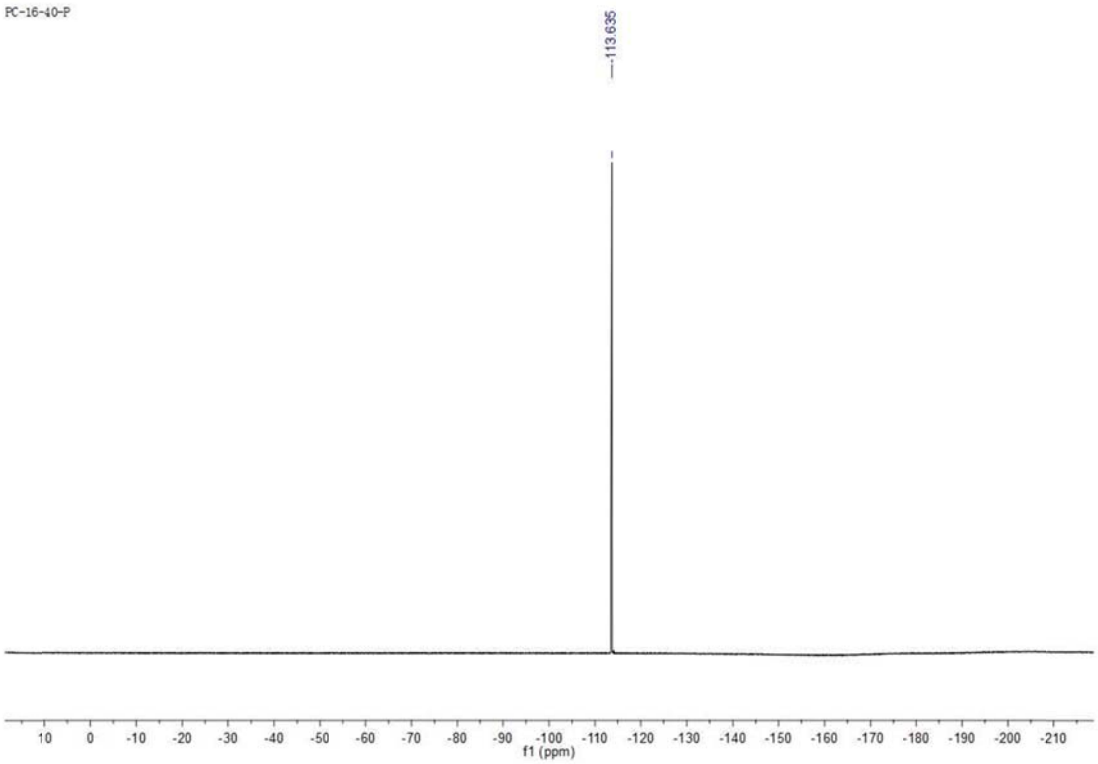

Supplementary Figure 70 ^{1}H NMR, ^{13}C NMR and ^{19}F NMR spectra for compound 55

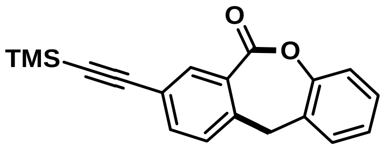

56

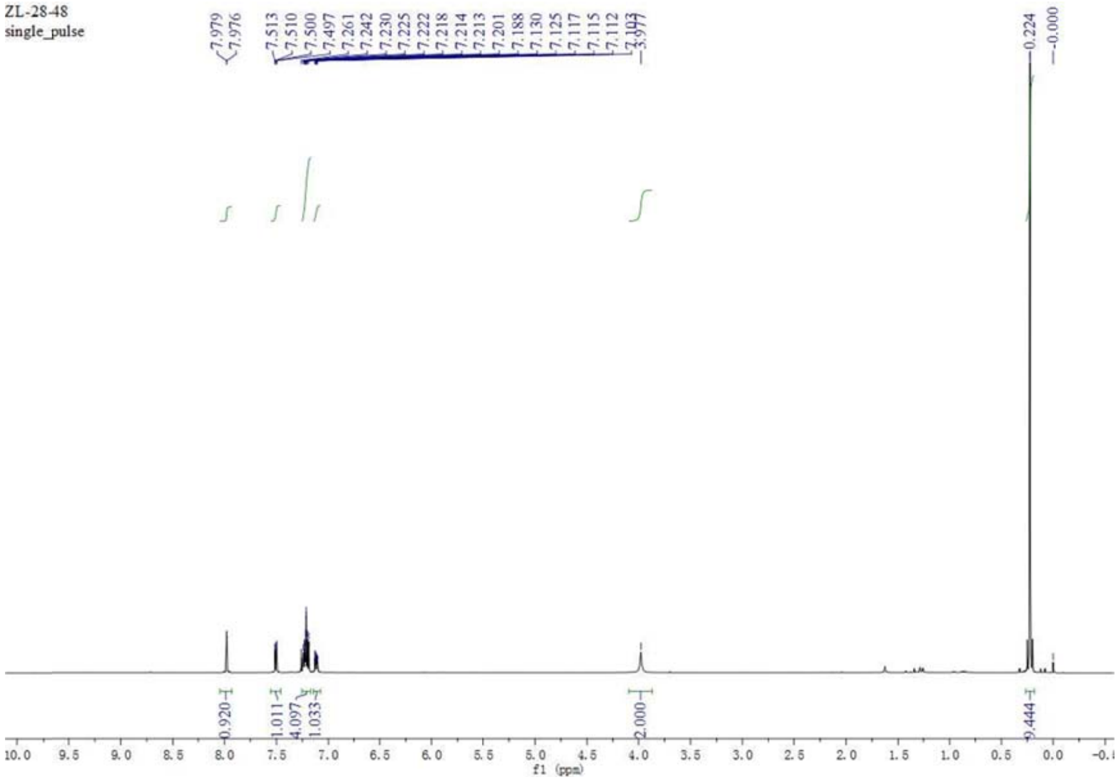

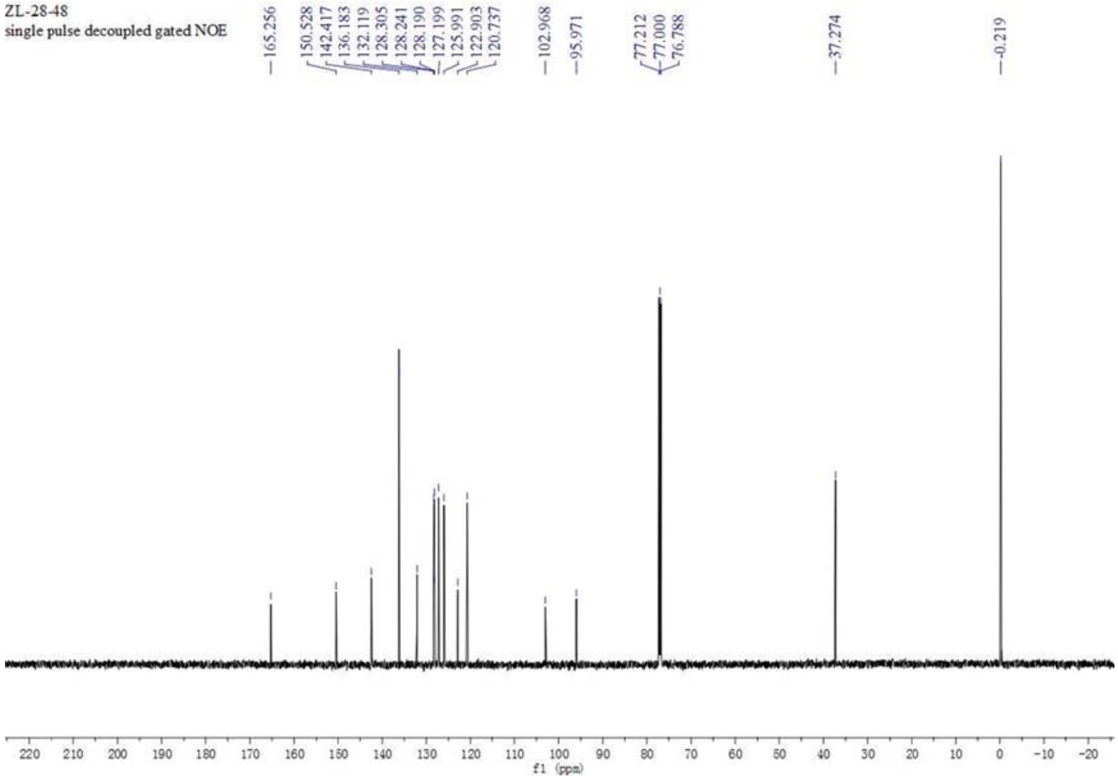

Supplementary Figure 71 ^{1}H NMR and ^{13}C NMR spectra for compound 56

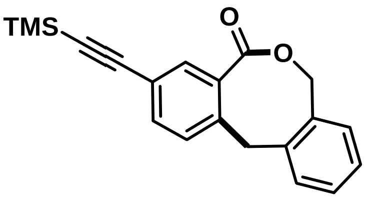

57

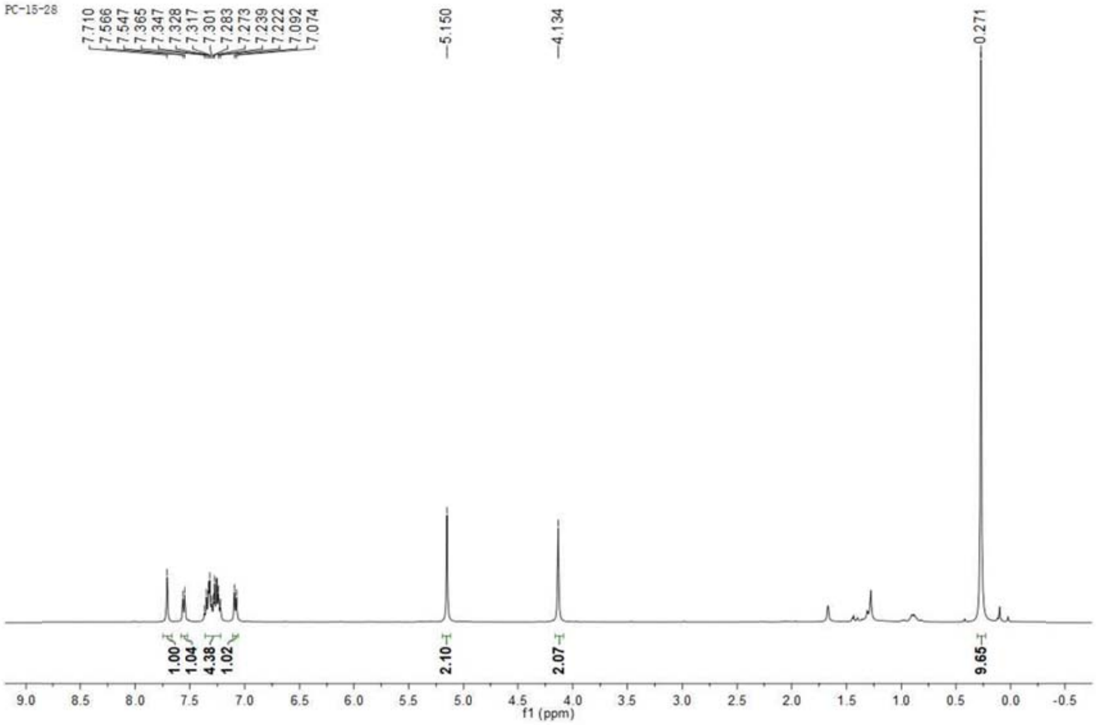

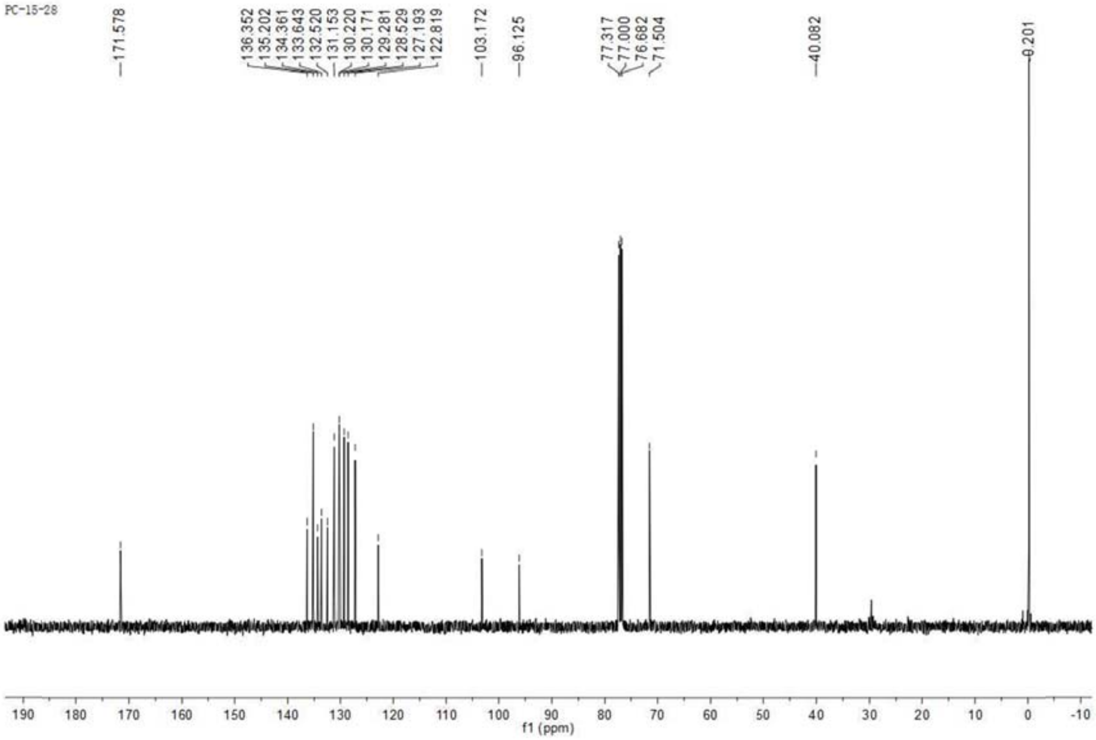

Supplementary Figure 72 ^{1}H NMR and ^{13}C NMR spectra for compound 57

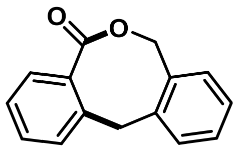

58

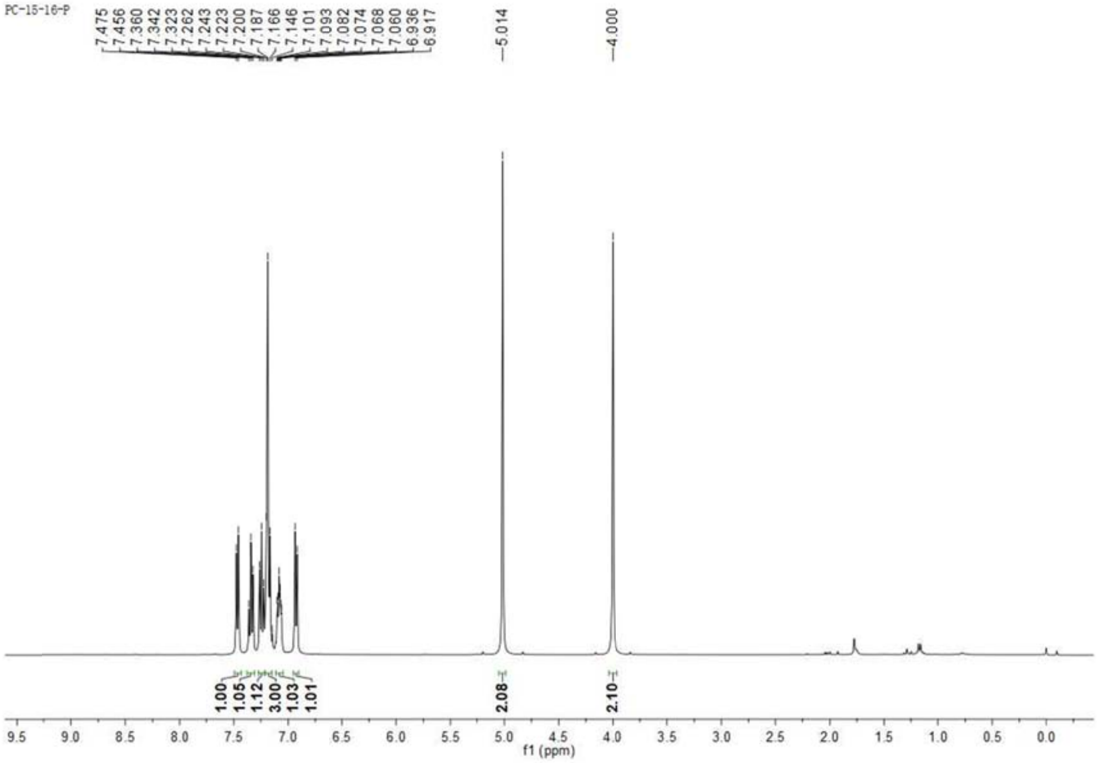

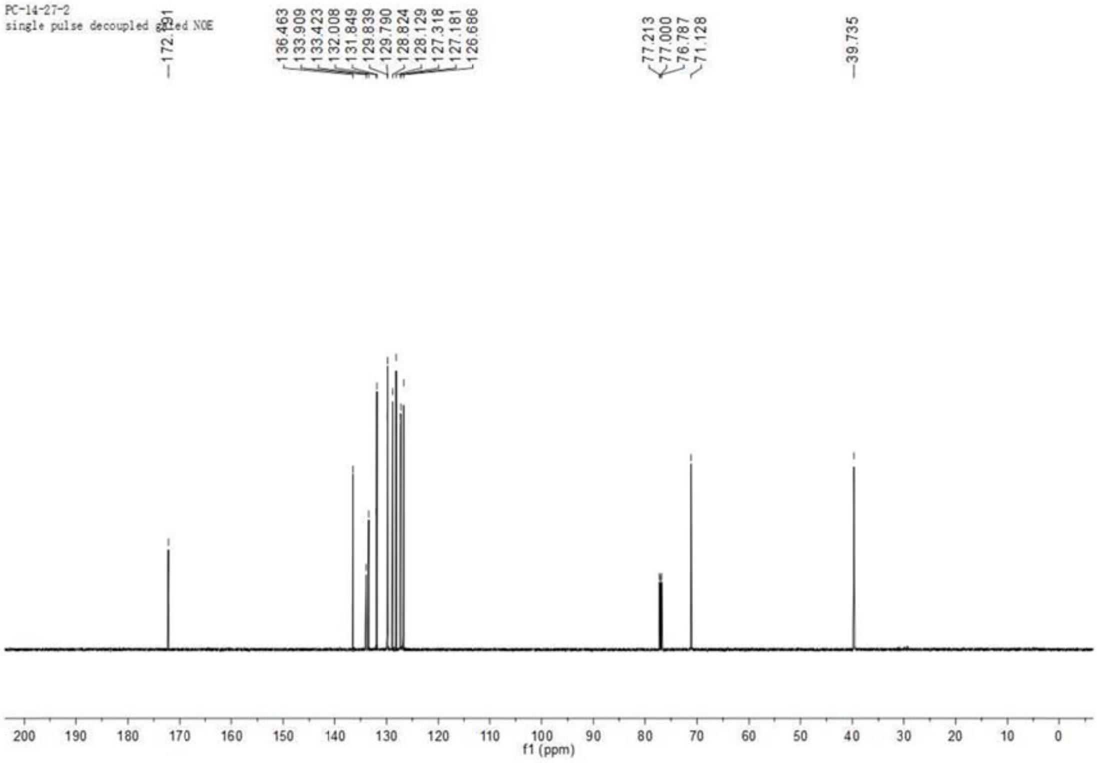

Supplementary Figure 73 ^{1}H NMR and ^{13}C NMR spectra for compound 58

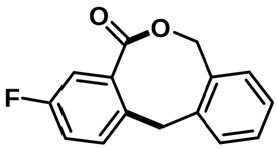

59

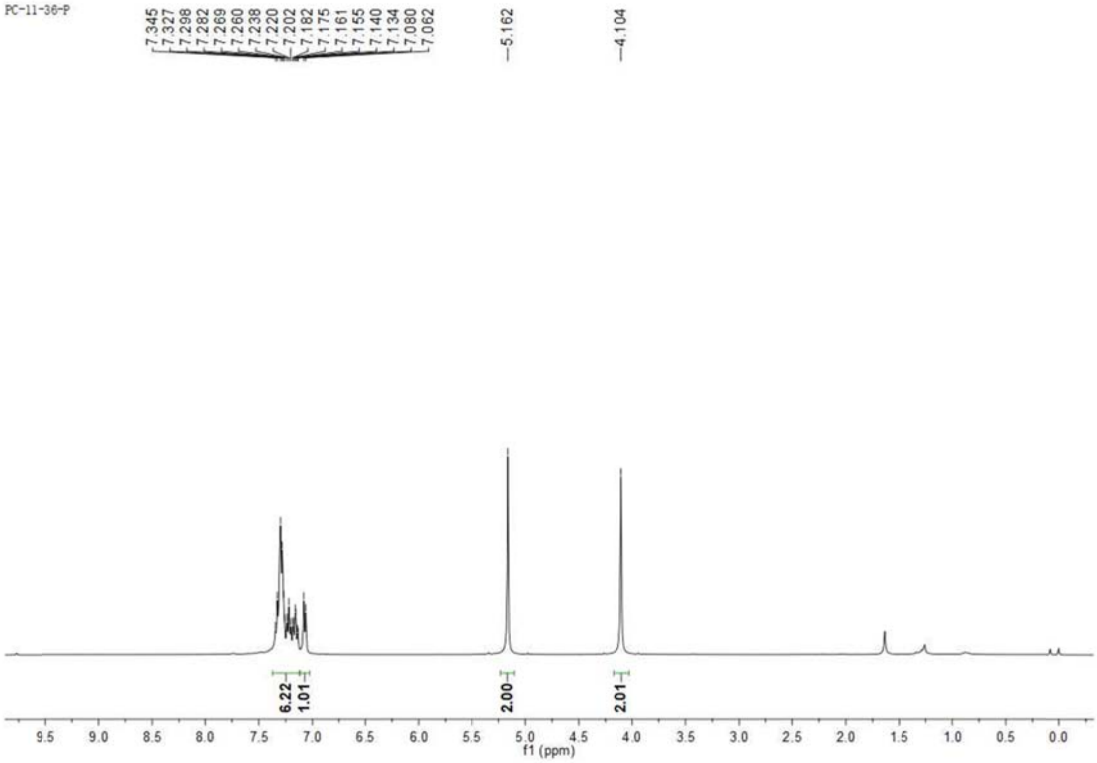
PC-11-36-P
7.345
7.327
7.298
7.282
7.269
7.260
7.238
7.220
7.202
7.182
7.175
7.161
7.155
7.140
7.134
7.080
7.062
5.162
4.104
6.22
1.01
2.00
2.01
9.5 9.0 8.5 8.0 7.5 7.0 6.5 6.0 5.5 5.0 4.5 4.0 3.5 3.0 2.5 2.0 1.5 1.0 0.5 0.0
f1 (ppm)

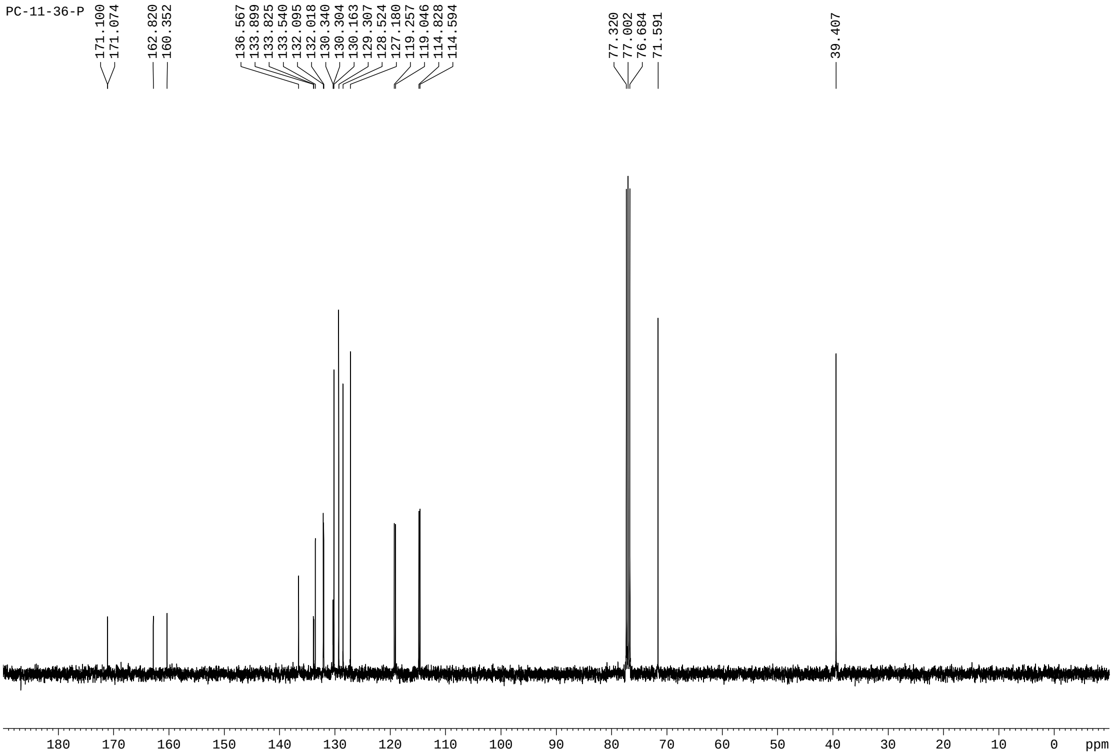
PC-11-36-P
171.100
171.074
162.820
160.352
136.567
133.899
133.825
133.540
132.095
132.018
130.340
130.304
130.163
129.307
128.524
127.180
119.257
119.046
114.828
114.594
77.320
77.002
76.684
71.591
39.407
180 170 160 150 140 130 120 110 100 90 80 70 60 50 40 30 20 10 0 ppm

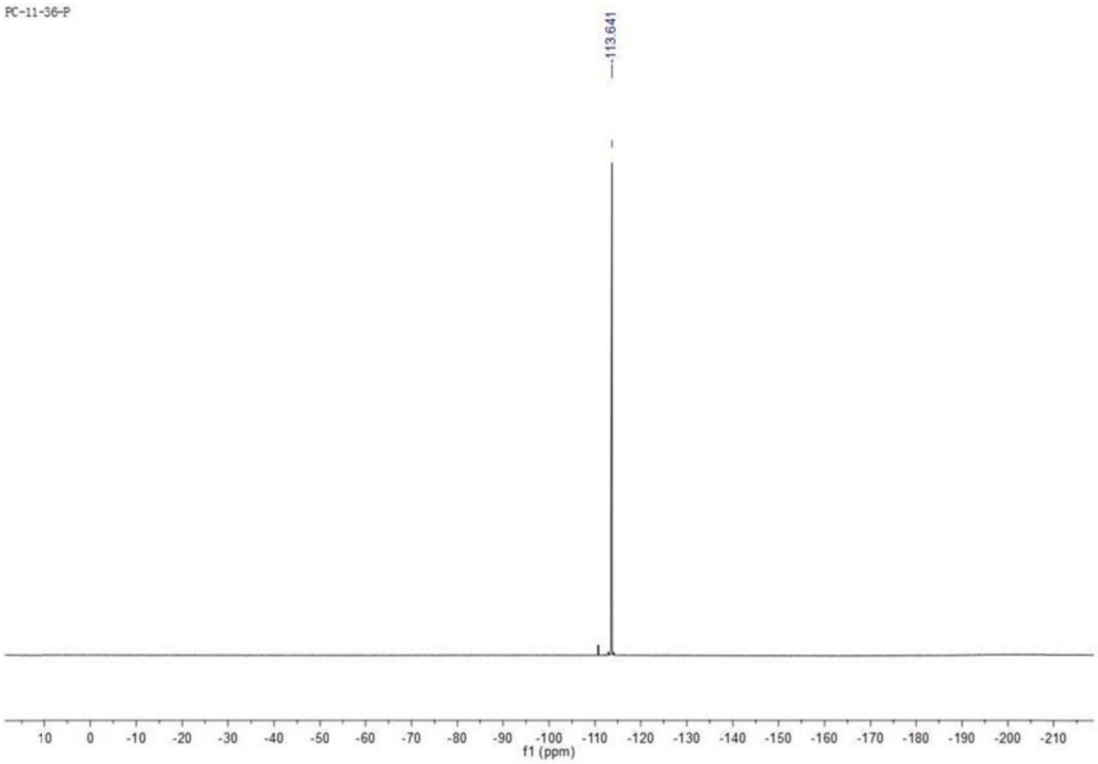

Supplementary Figure 74 ^{1}H NMR, ^{13}C NMR and ^{19}F NMR spectra for compound 59

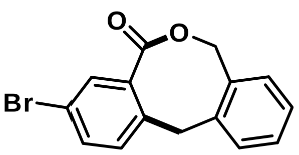

60

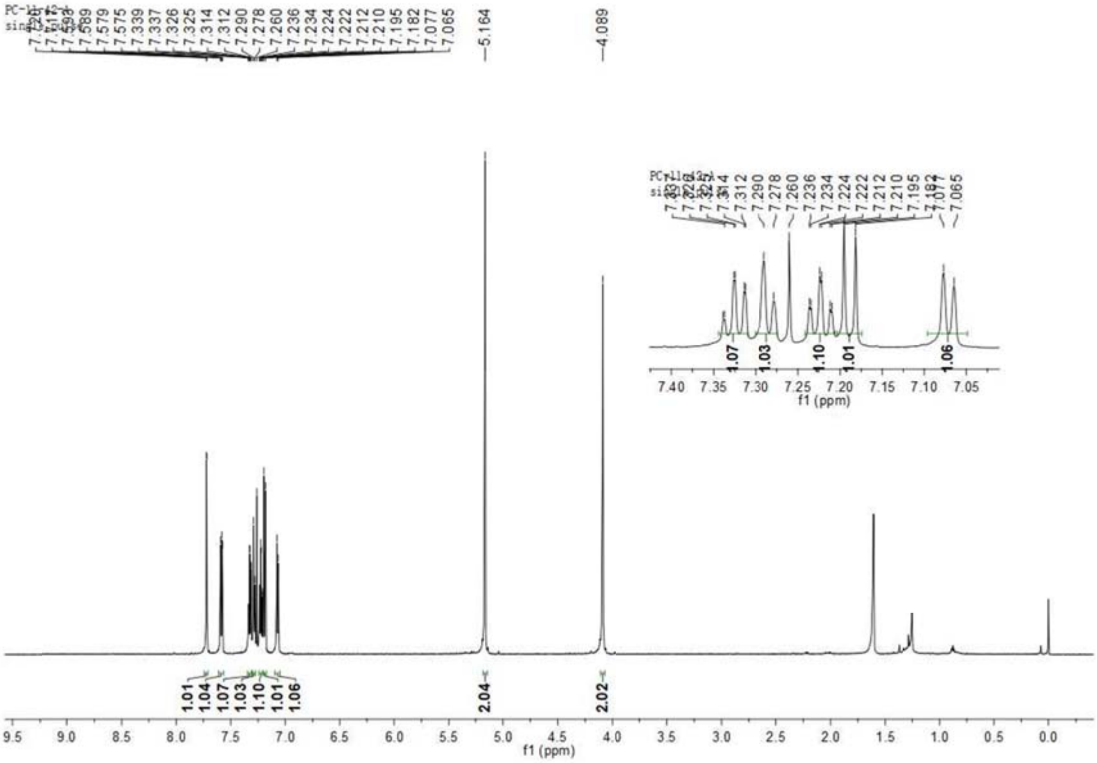

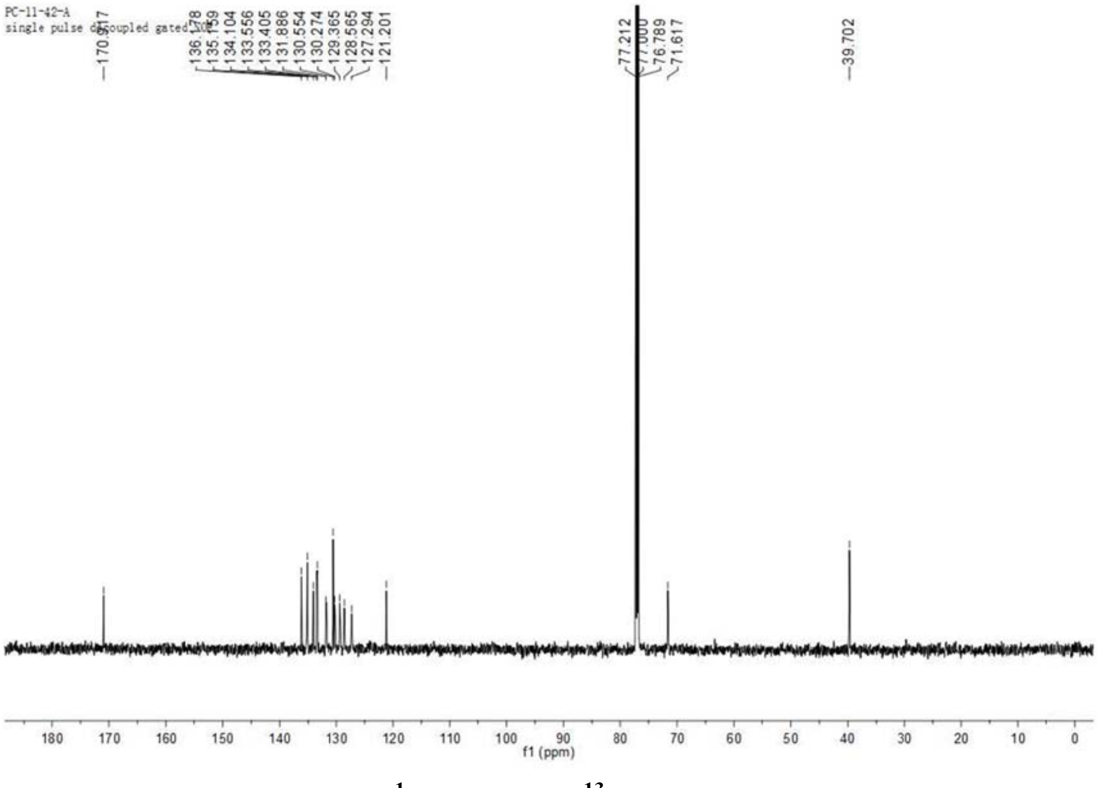

Supplementary Figure 75 ^{1}H NMR and ^{13}C NMR spectra for compound 60

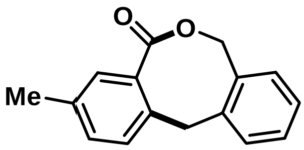

61

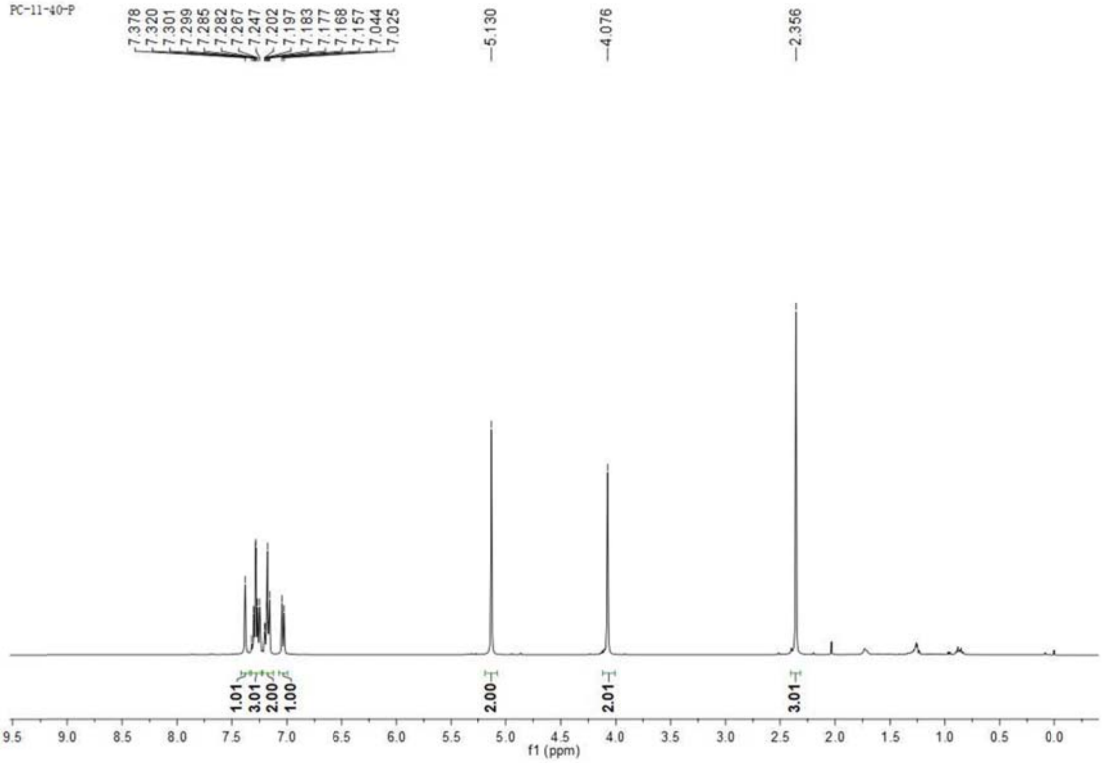

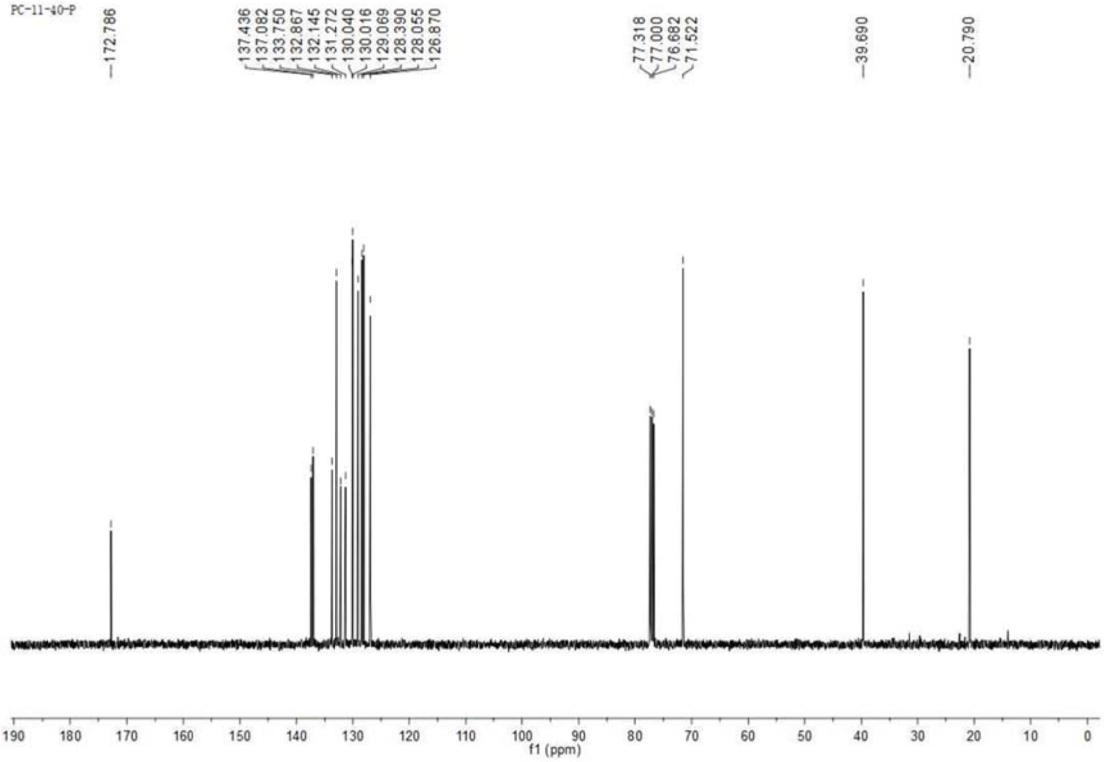

Supplementary Figure 76 ^{1}H NMR and ^{13}C NMR spectra for compound 61

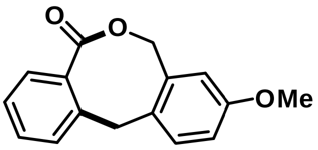

62

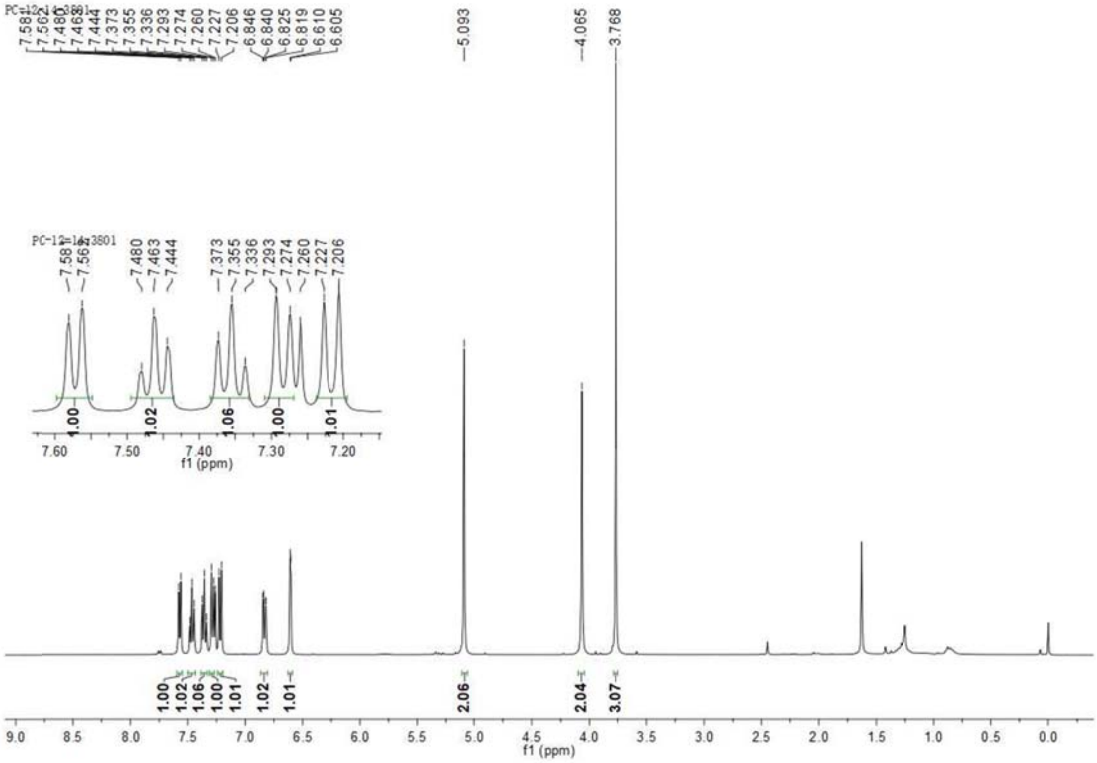

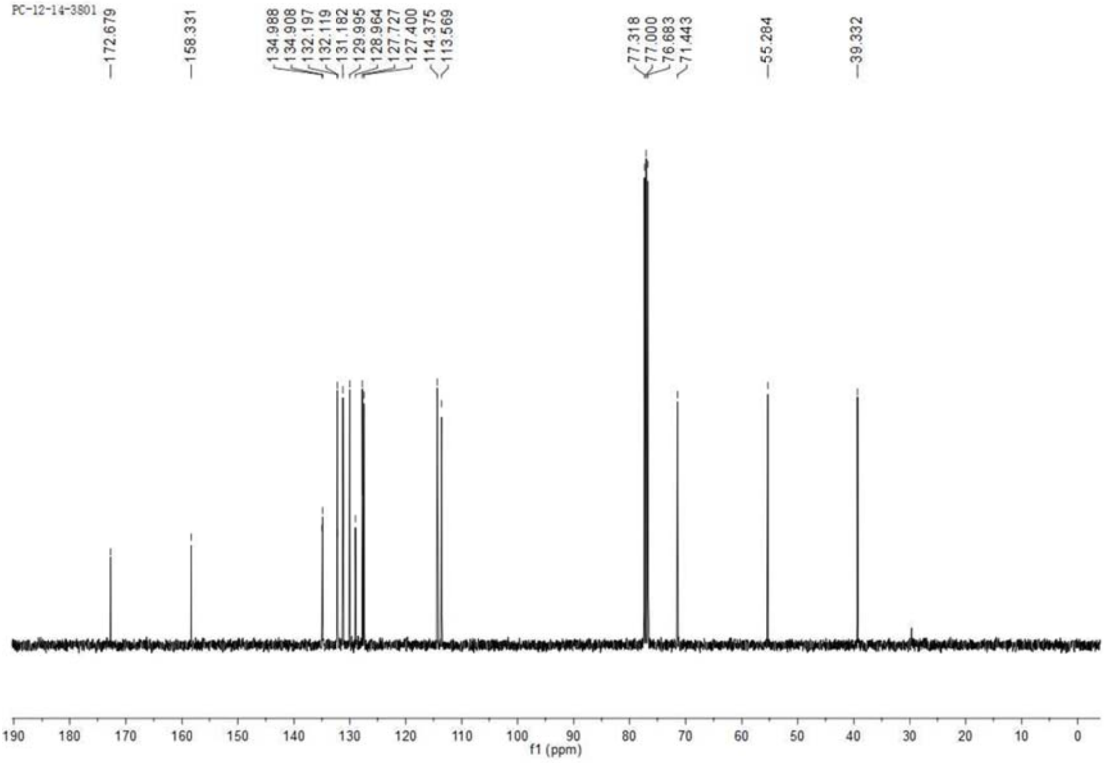

Supplementary Figure 77 ^{1}H NMR and ^{13}C NMR spectra for compound 62

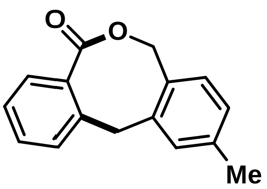

63

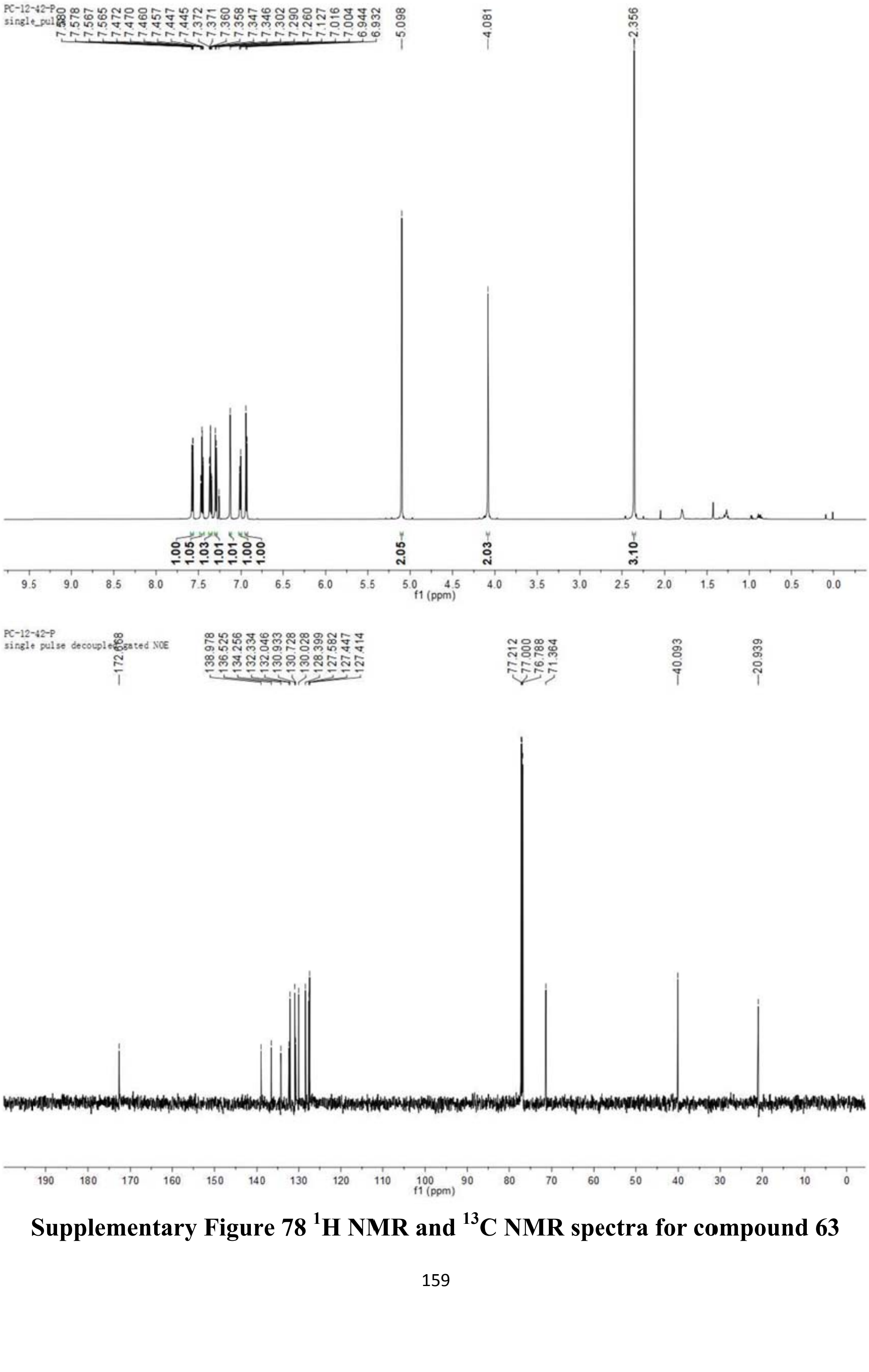

Supplementary Figure 78 ^{1}H NMR and ^{13}C NMR spectra for compound 63

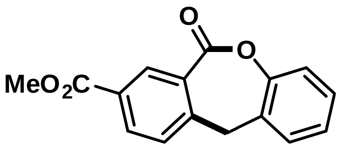

64

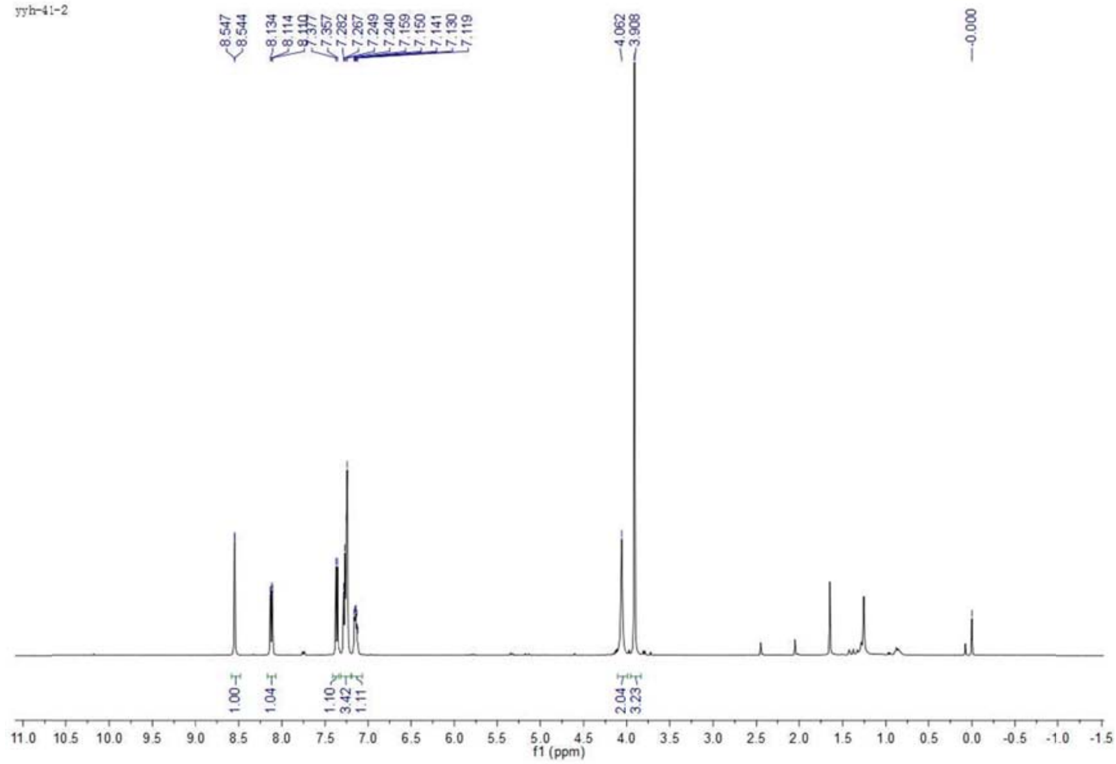

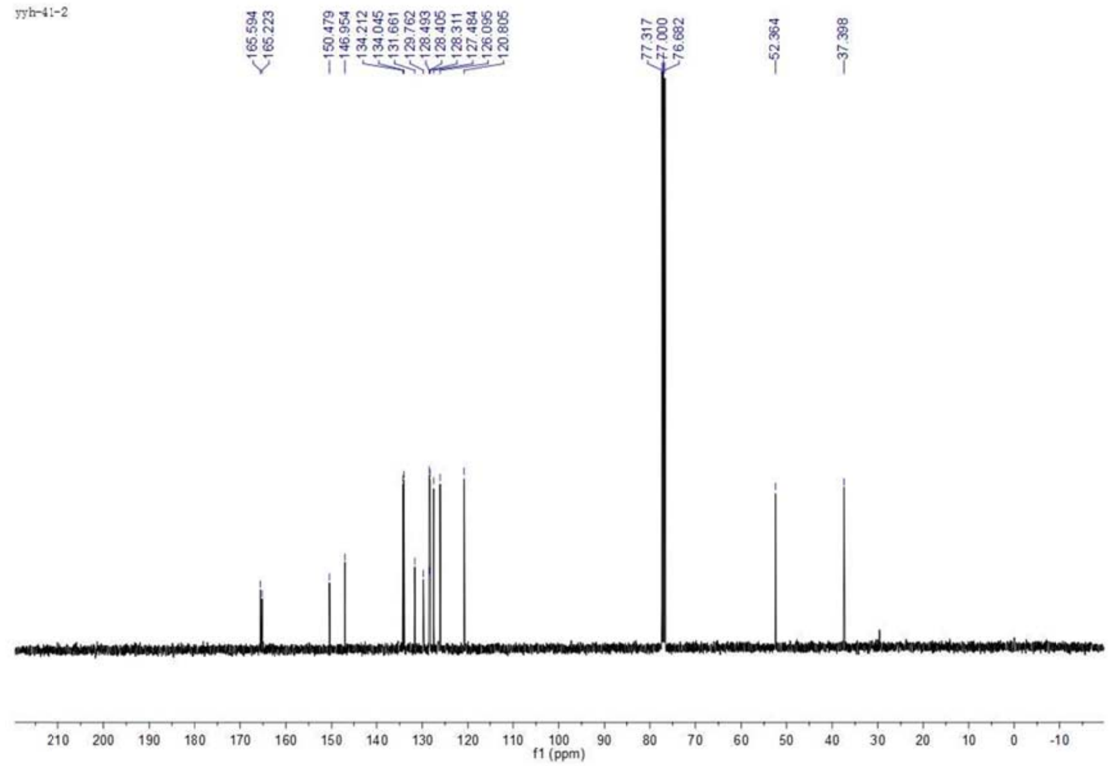

Supplementary Figure 79 ^{1}H NMR and ^{13}C NMR spectra for compound 64

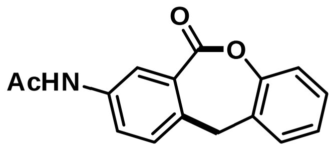

65

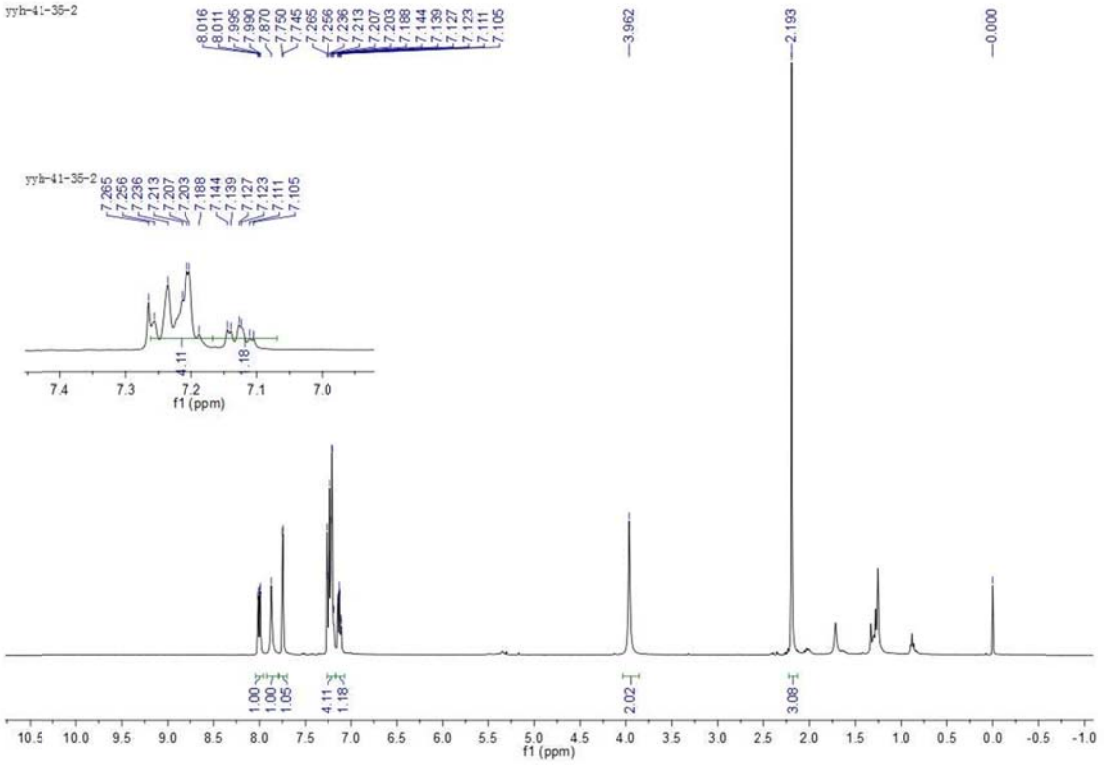

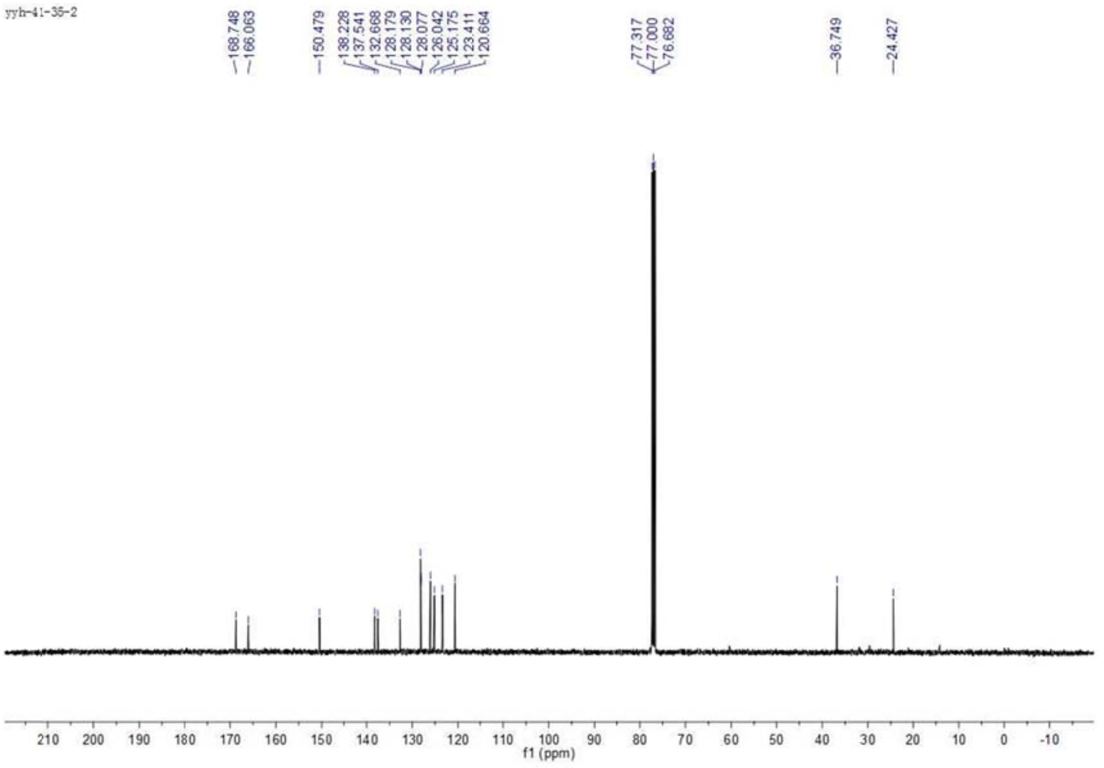

Supplementary Figure 80 ^{1}H NMR and ^{13}C NMR spectra for compound 65

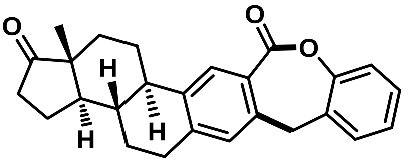

66

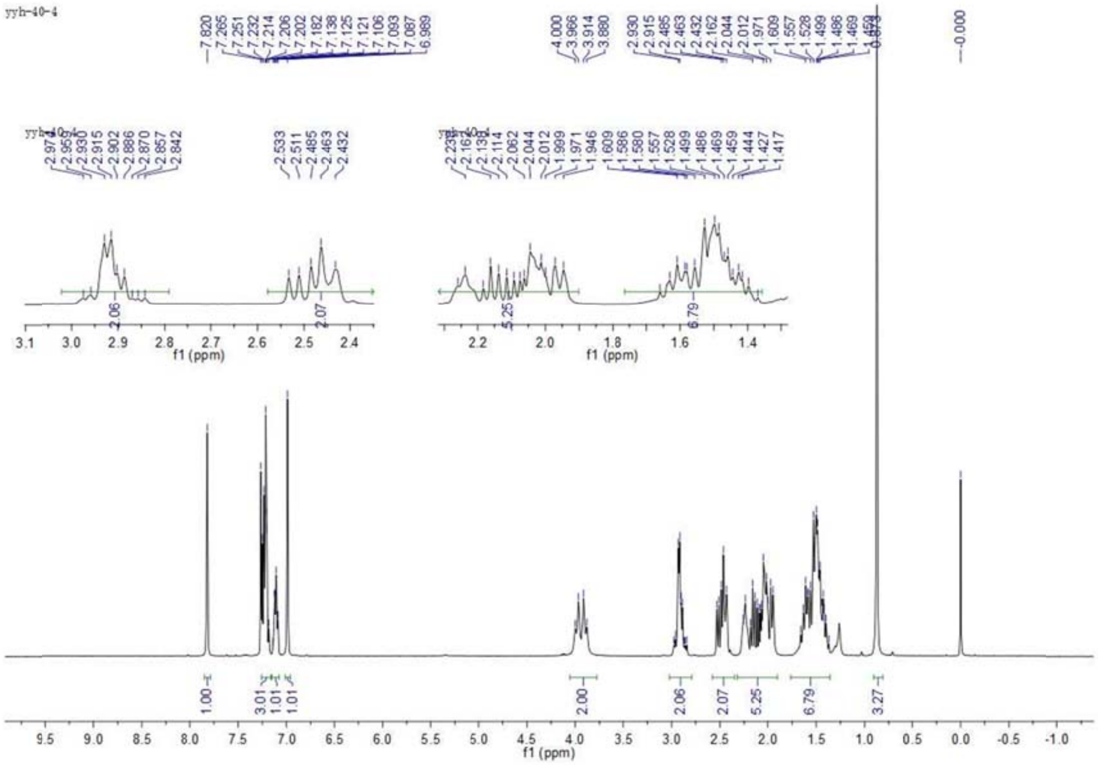

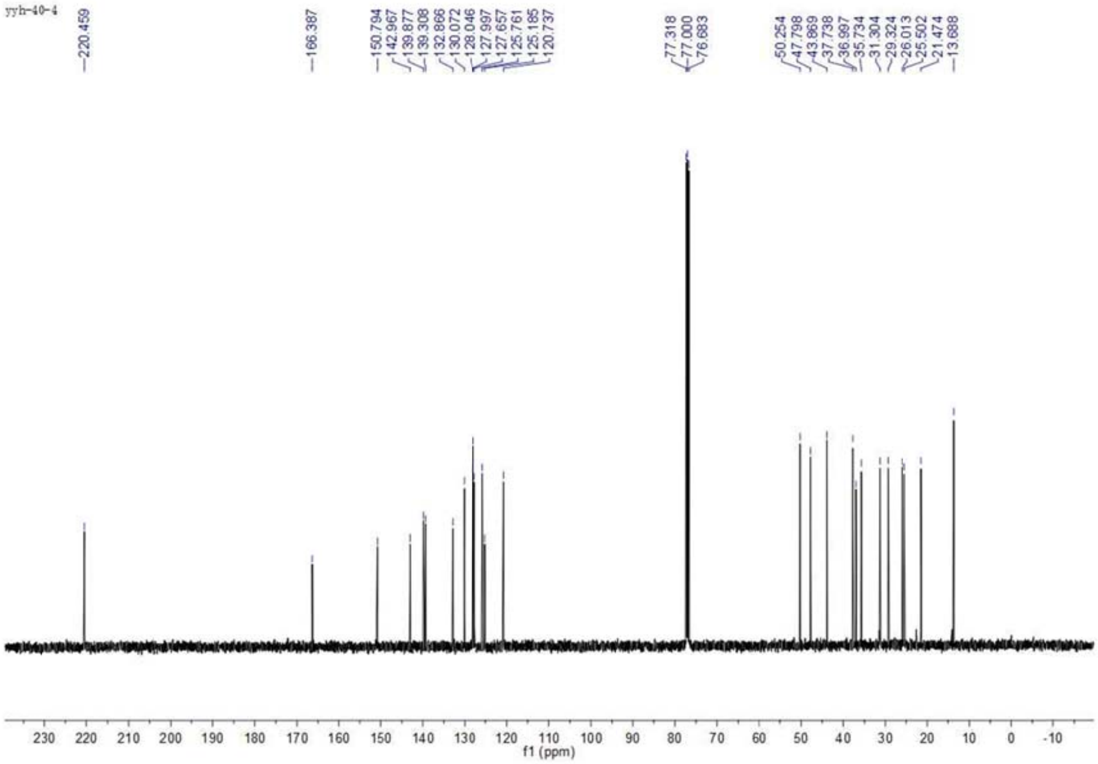

Supplementary Figure 81 ^{1}H NMR and ^{13}C NMR spectra for compound 66

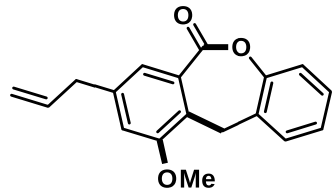

67

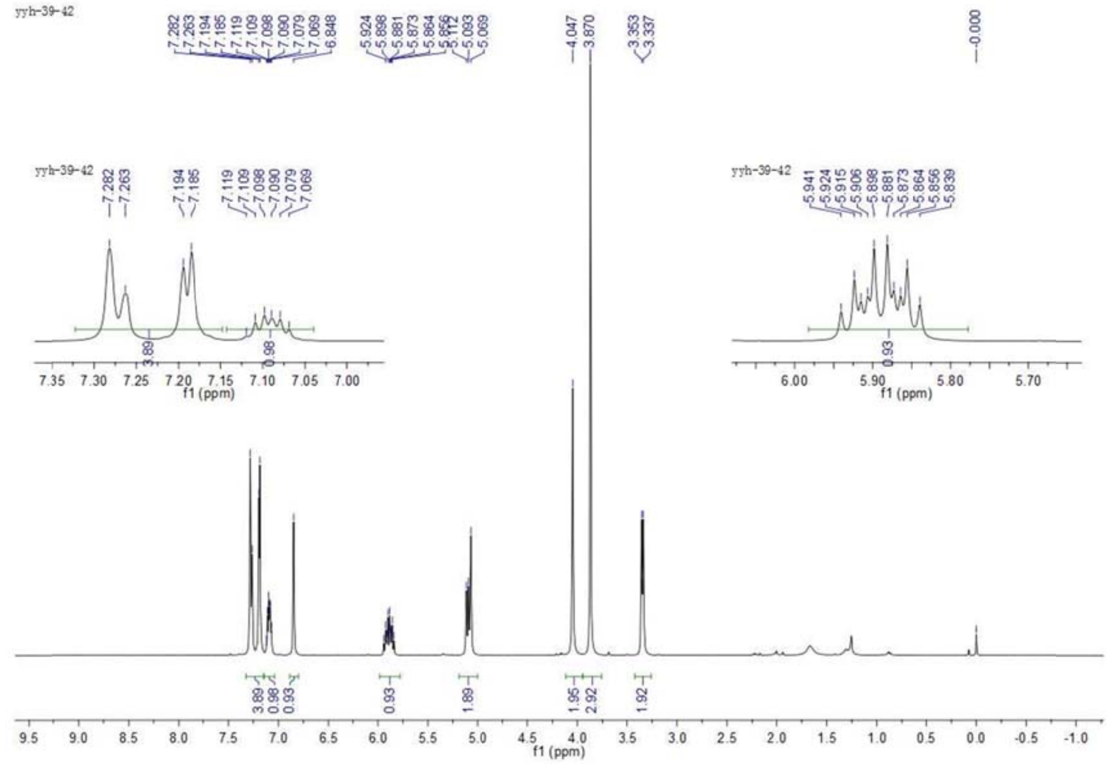

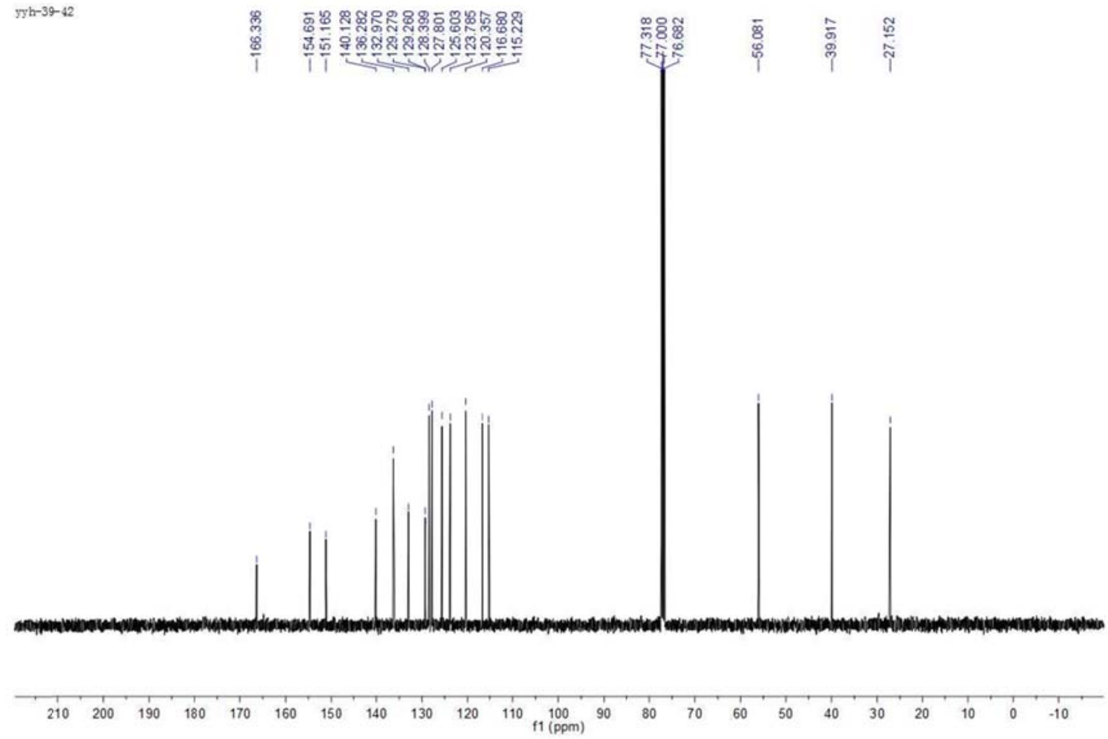

Supplementary Figure 82 ^{1}H NMR and ^{13}C NMR spectra for compound 67

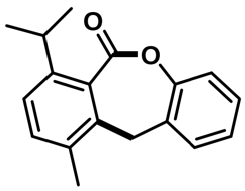

68

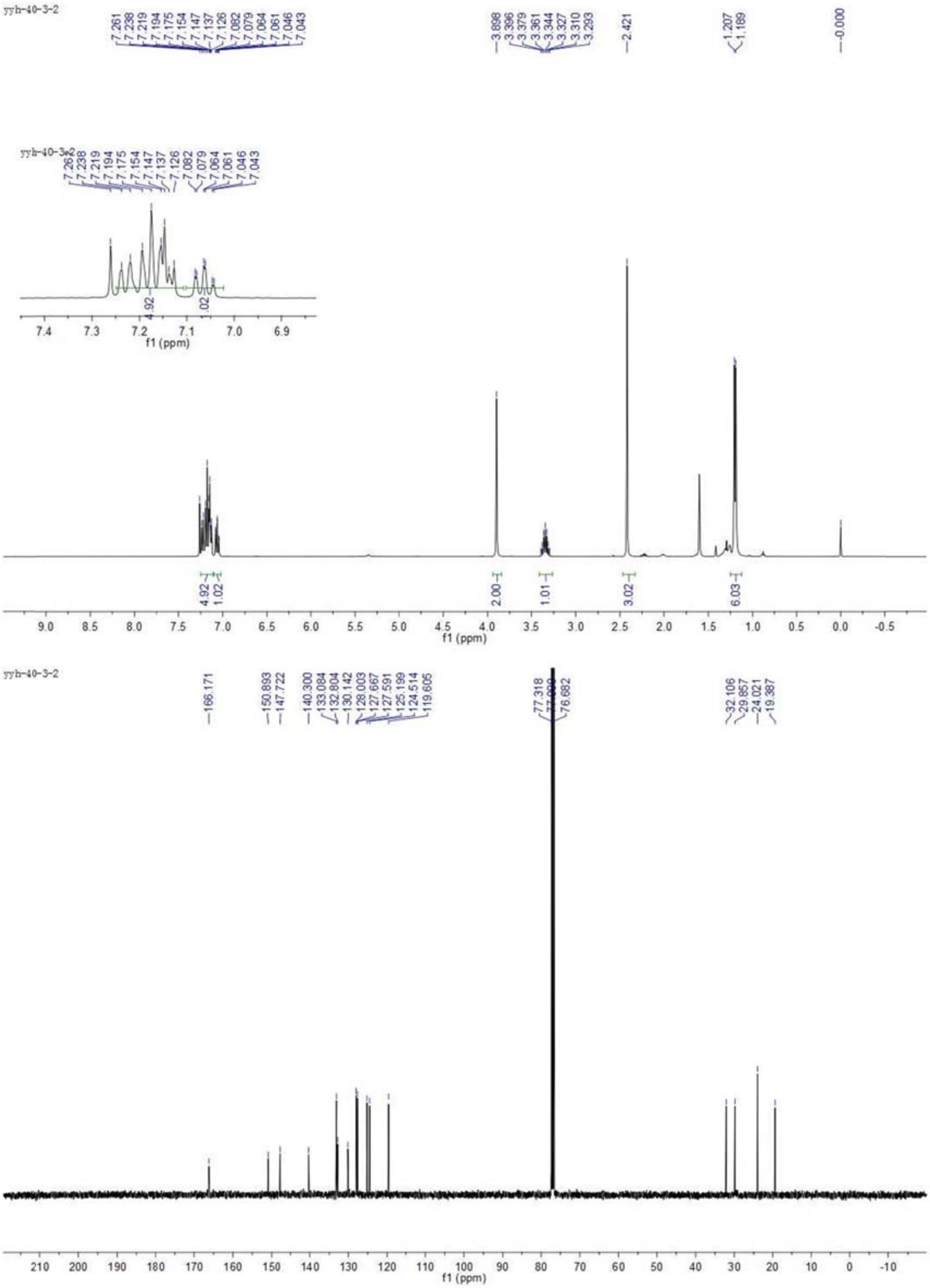

Supplementary Figure 83 ^{1}H NMR and ^{13}C NMR spectra for compound 68

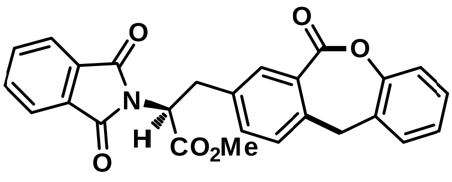

69

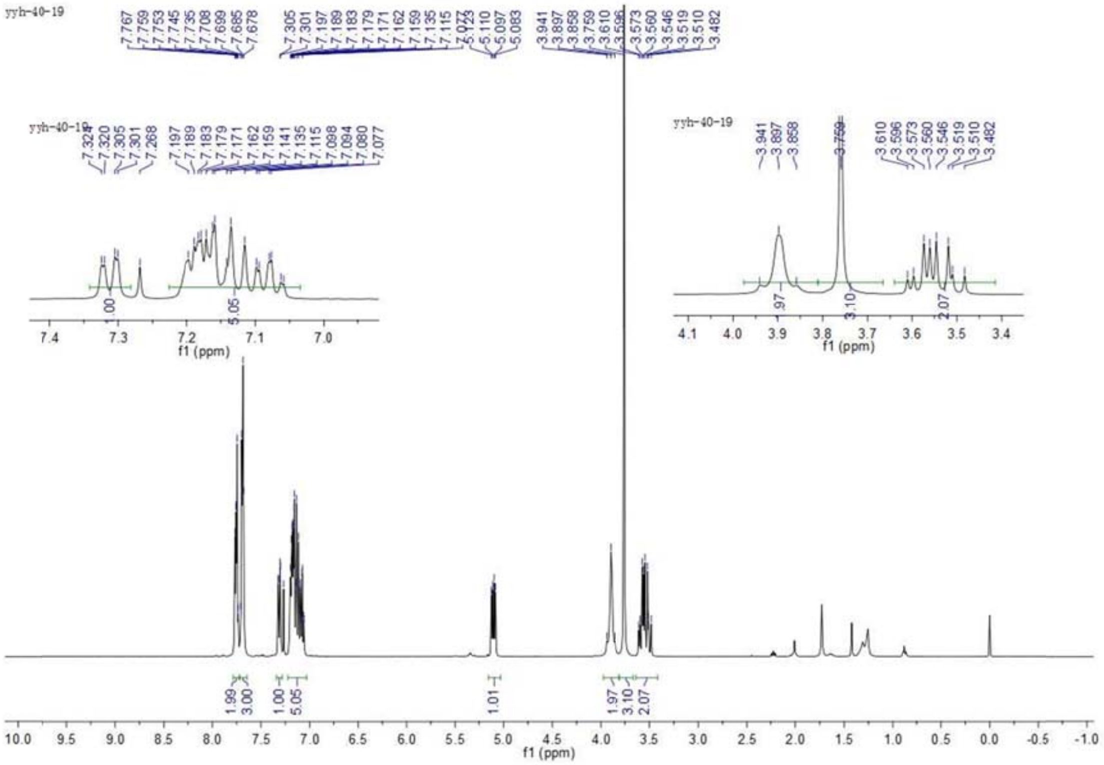

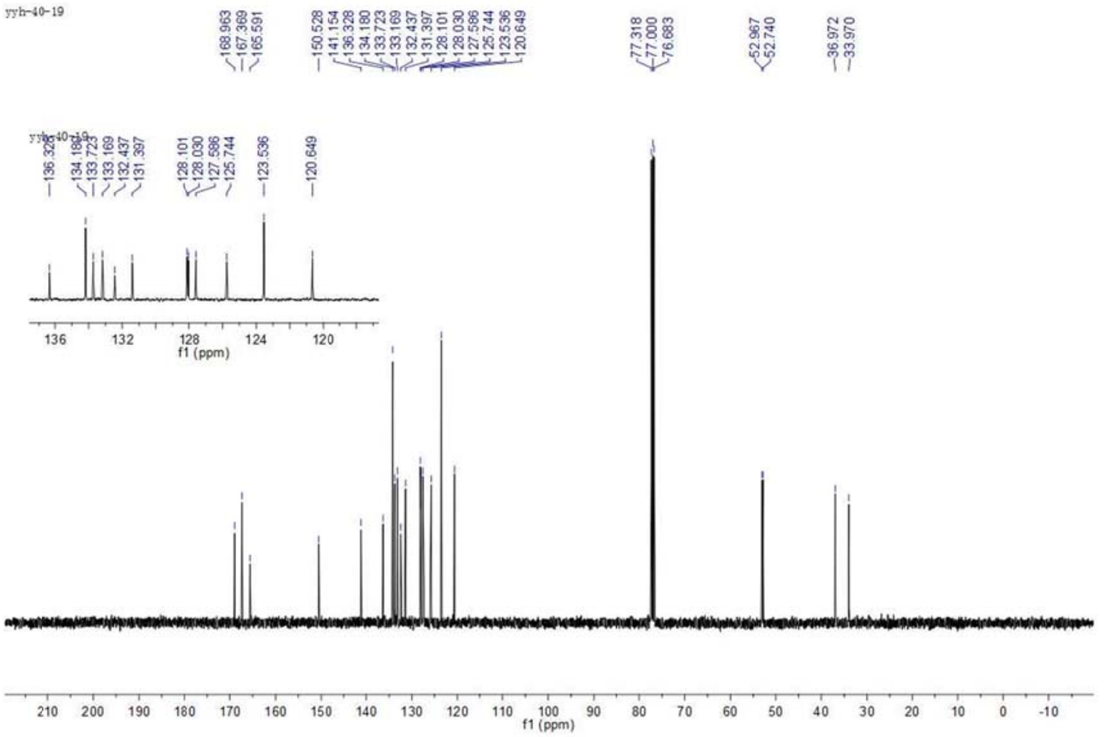

Supplementary Figure 84 ^{1}H NMR and ^{13}C NMR spectra for compound 69

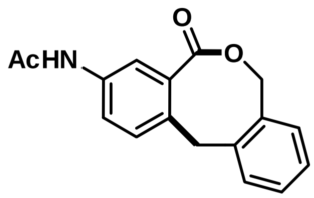

70

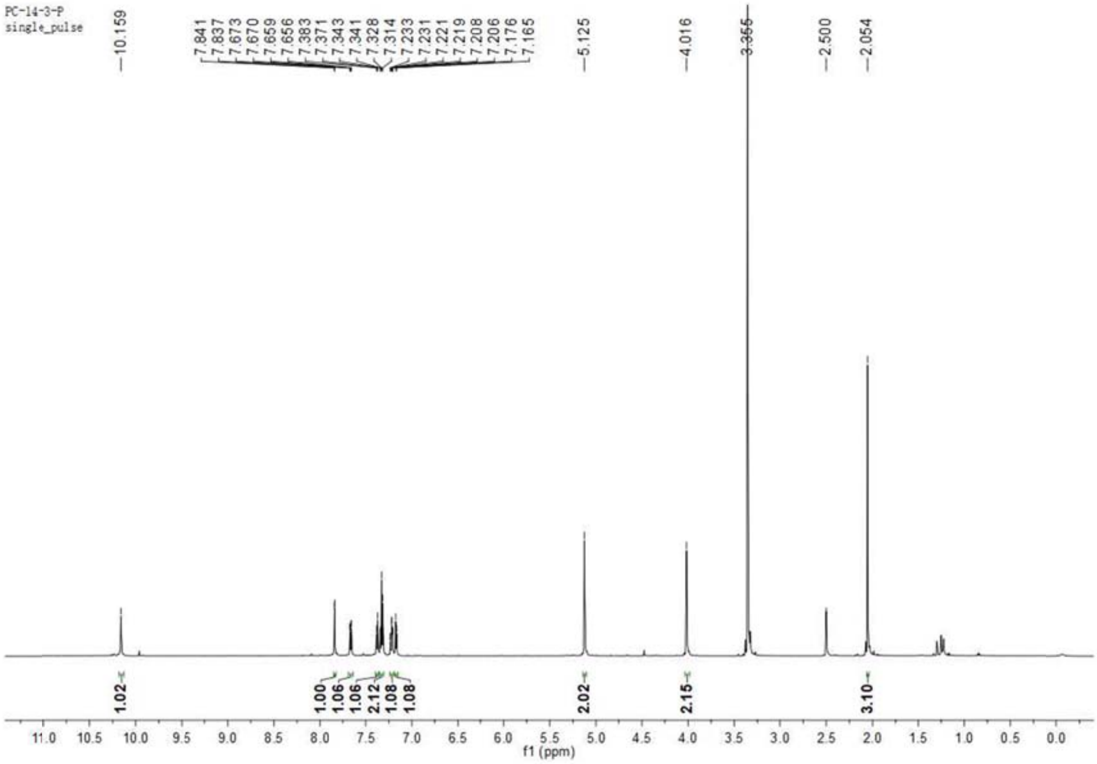

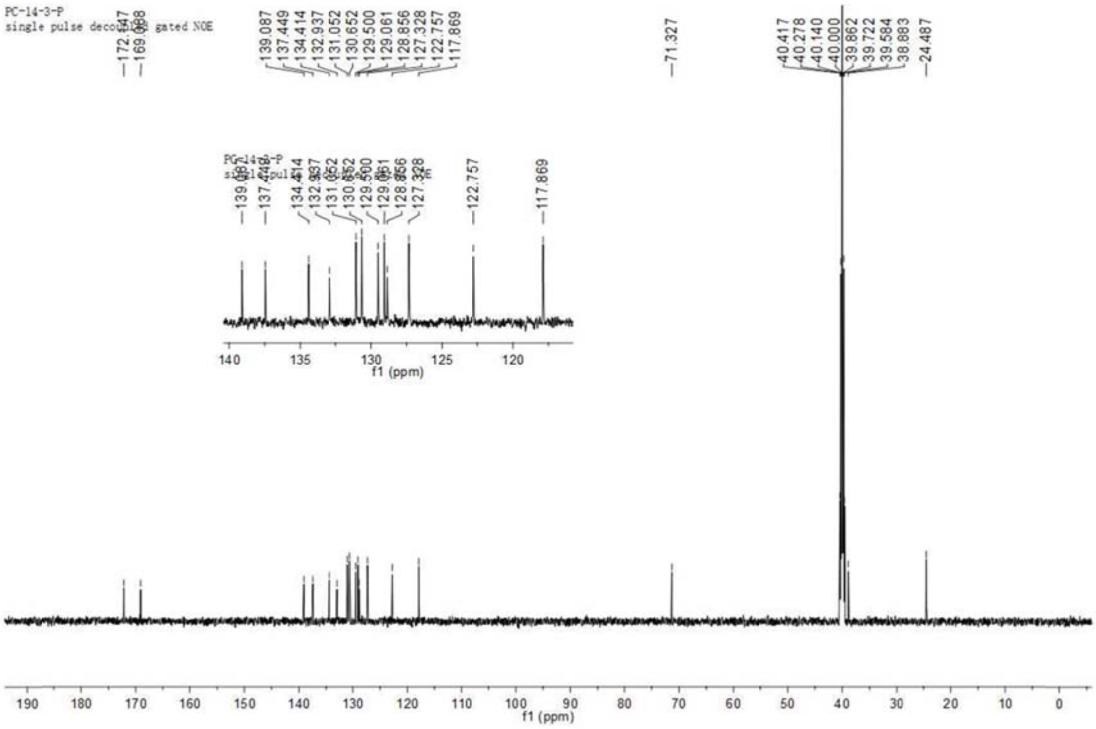

Supplementary Figure 85 ^{1}H NMR and ^{13}C NMR spectra for compound 70

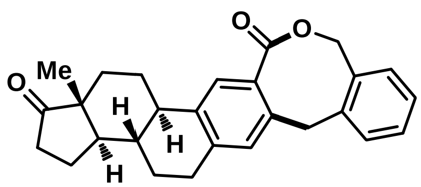

71

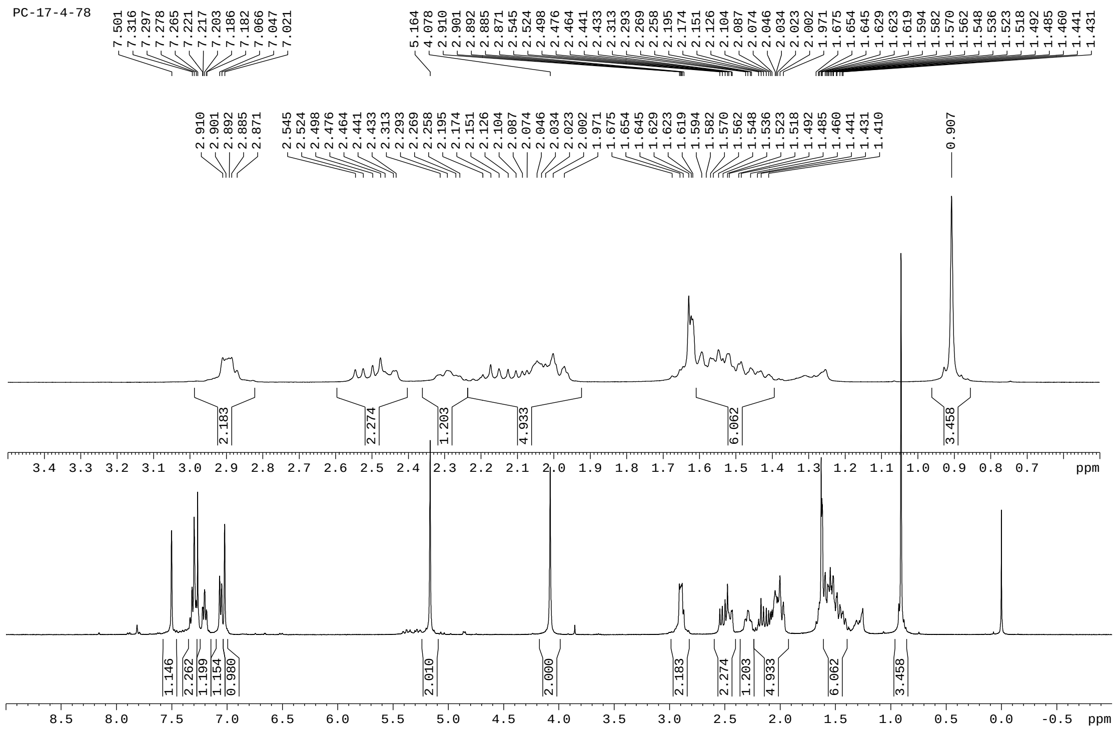

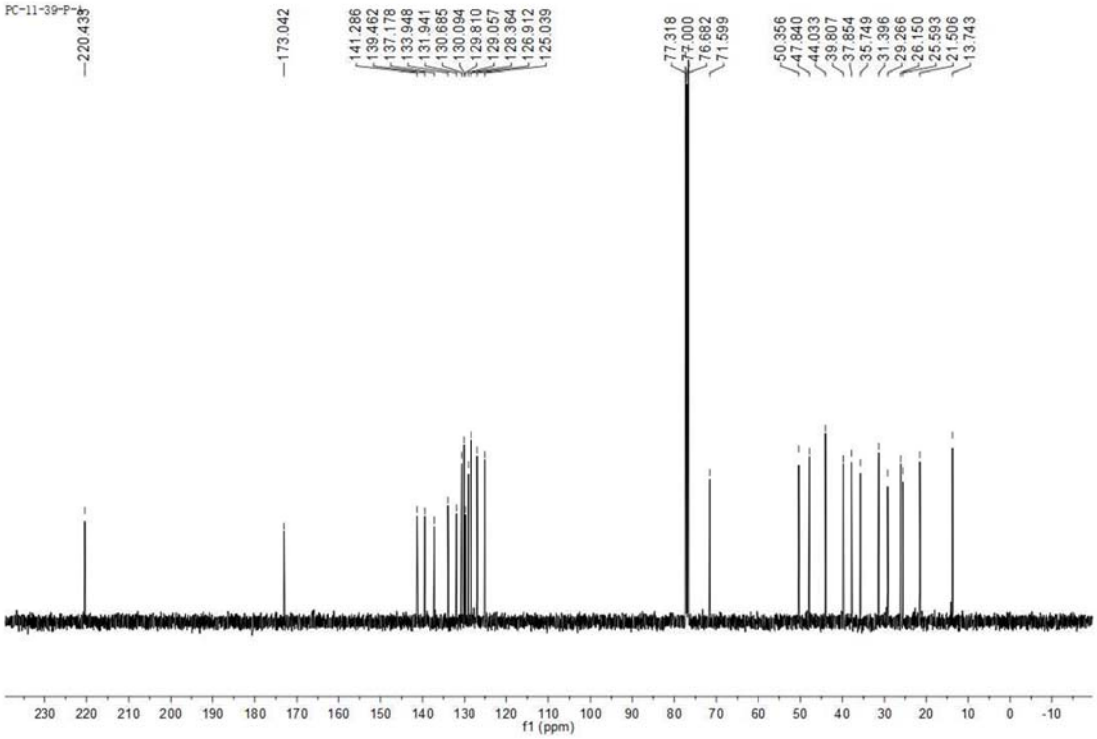

Supplementary Figure 86 ^{1}H NMR and ^{13}C NMR spectra for compound 71

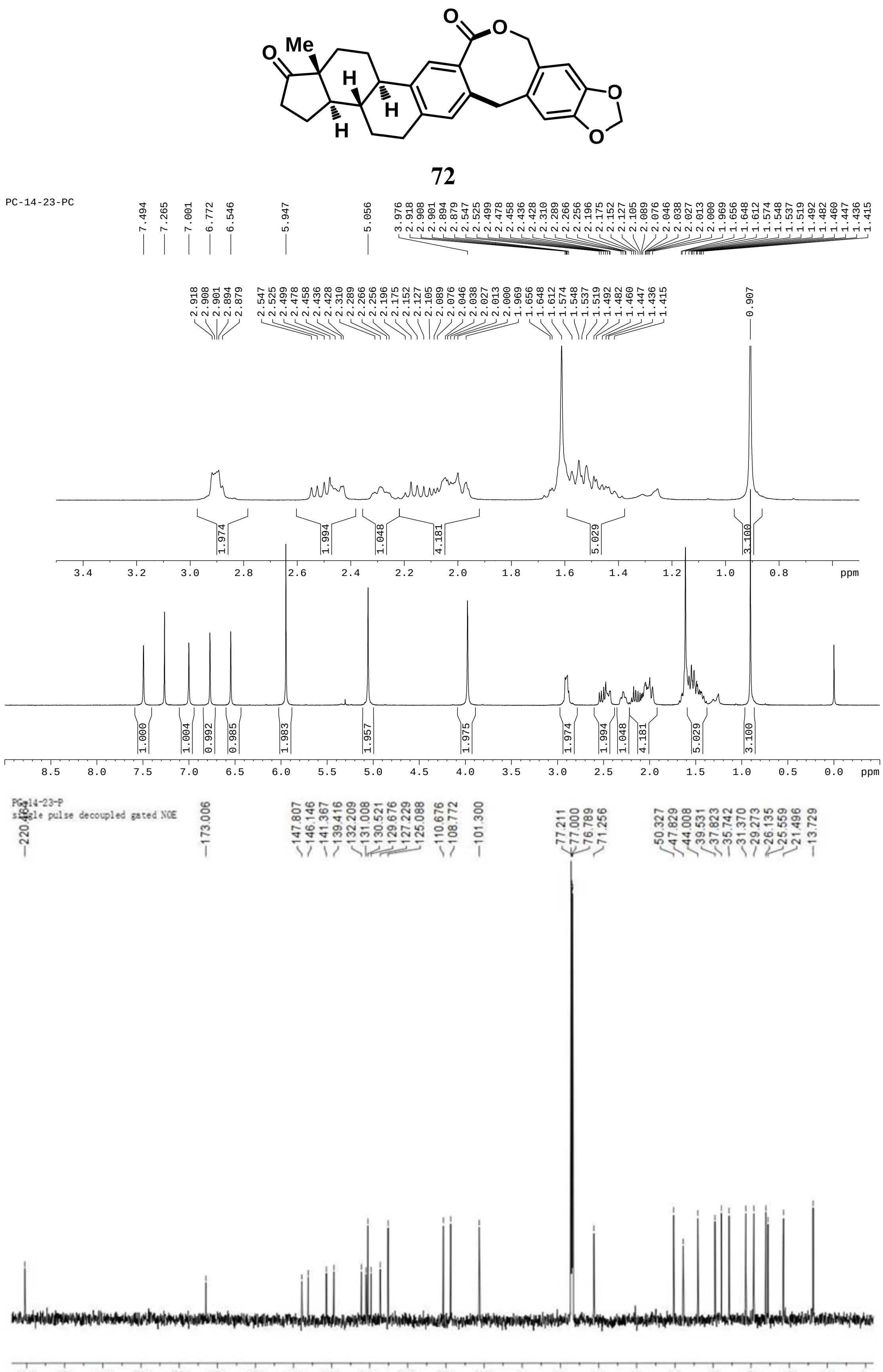

Supplementary Figure 87 ^{1}H NMR and ^{13}C NMR spectra for compound 72

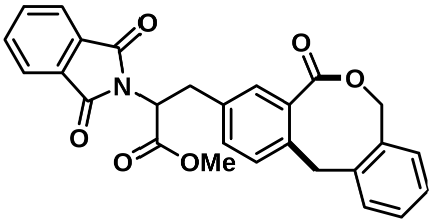

73

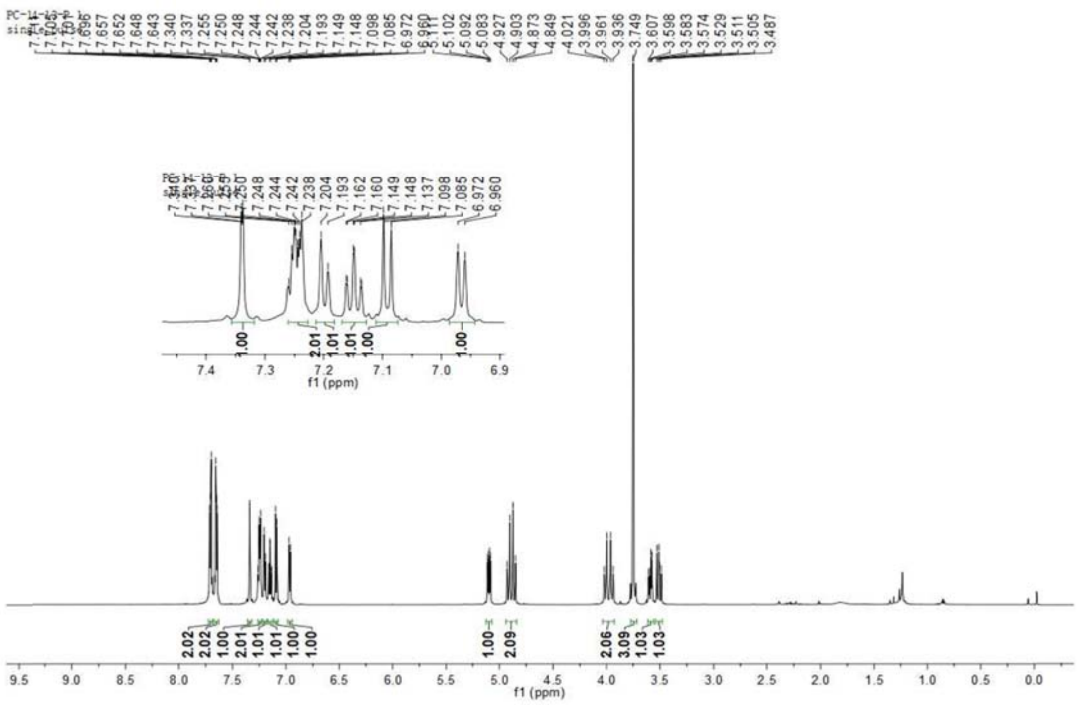

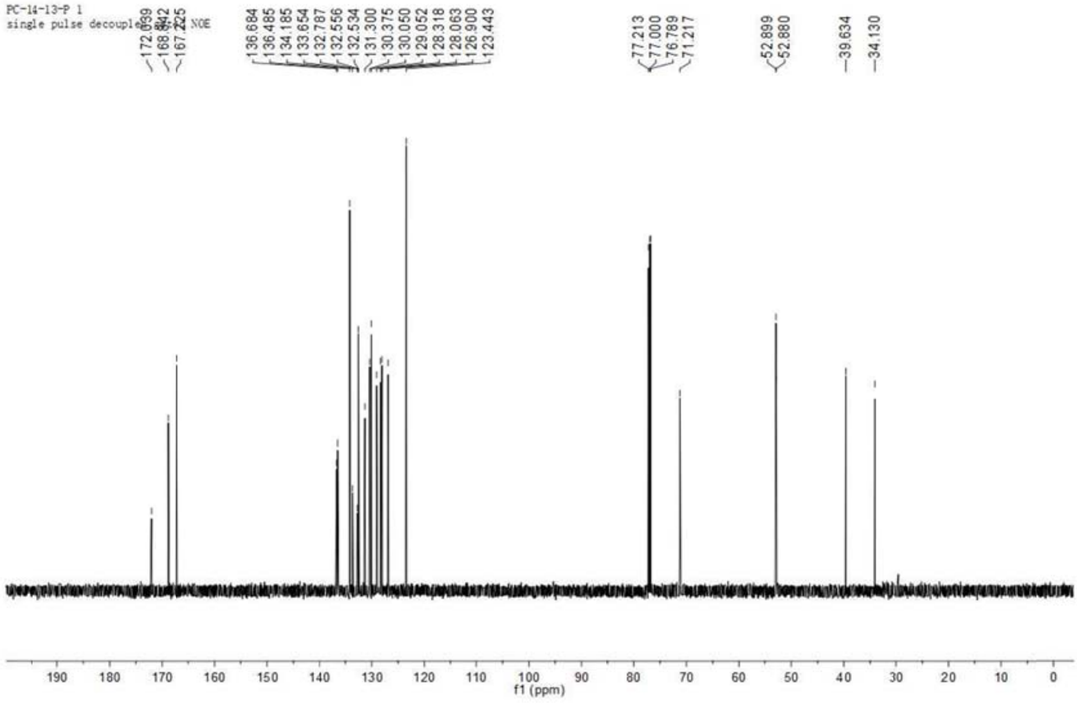

Supplementary Figure 88 ^{1}H NMR and ^{13}C NMR spectra for compound 73

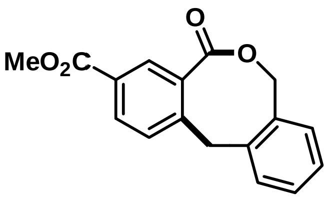

74

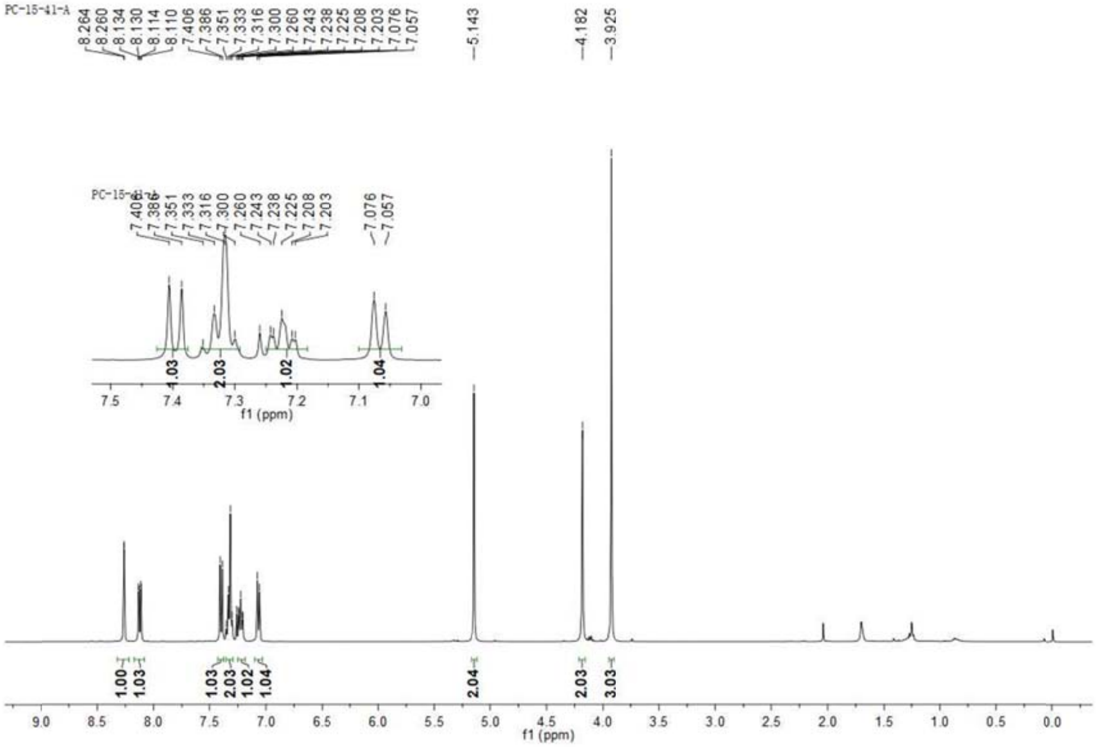

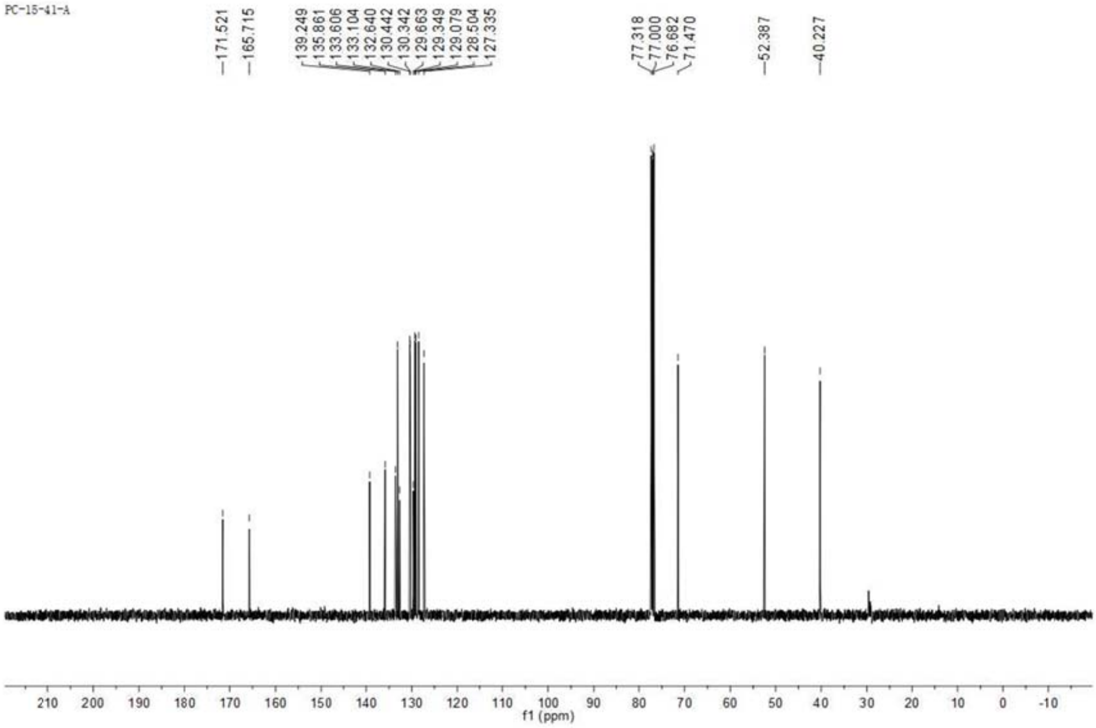

Supplementary Figure 89 ^{1}H NMR and ^{13}C NMR spectra for compound 74

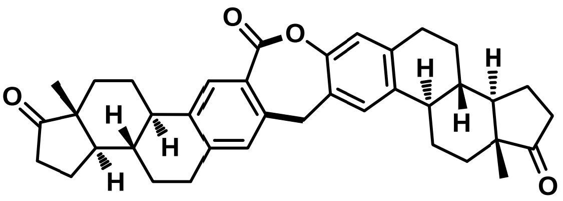

75

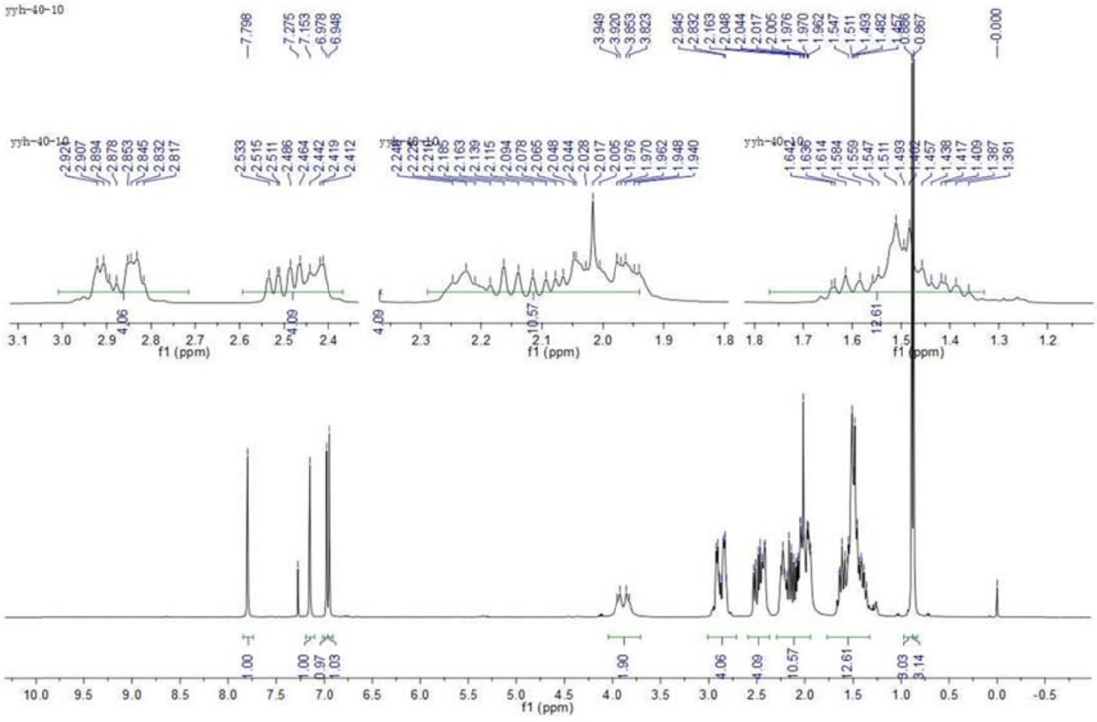

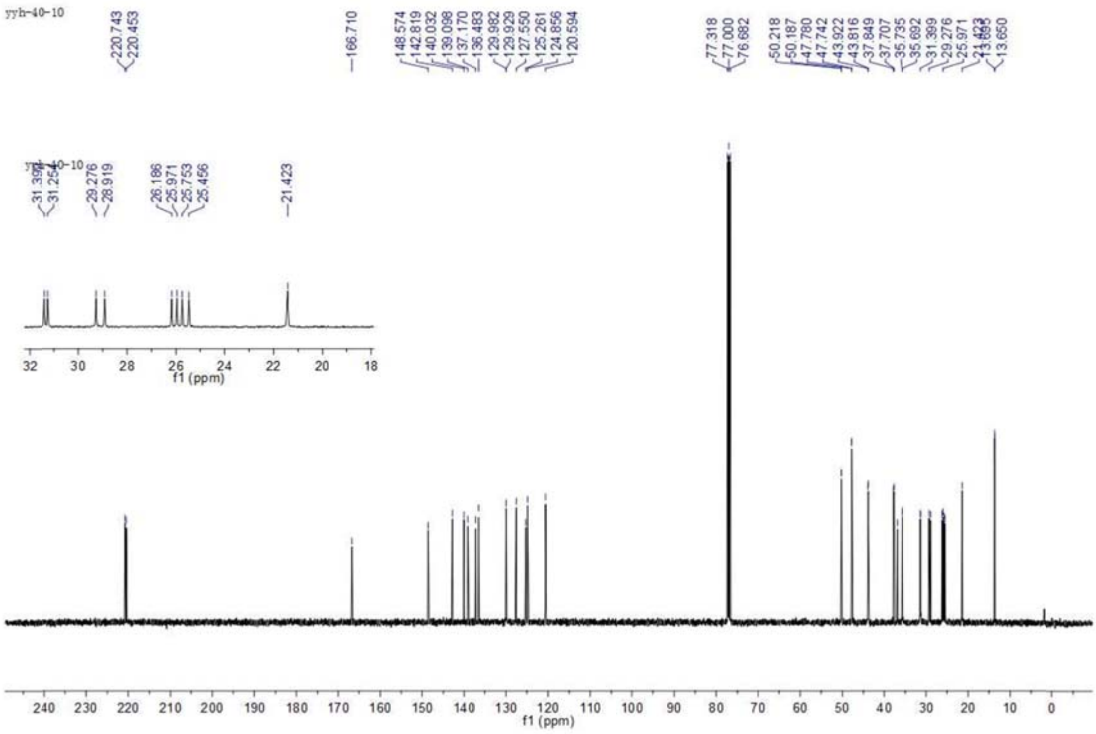

Supplementary Figure 90 ^{1}H NMR and ^{13}C NMR spectra for compound 75

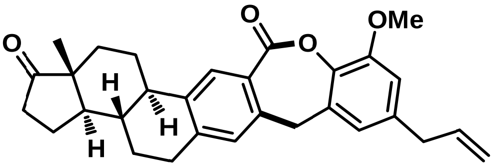

76

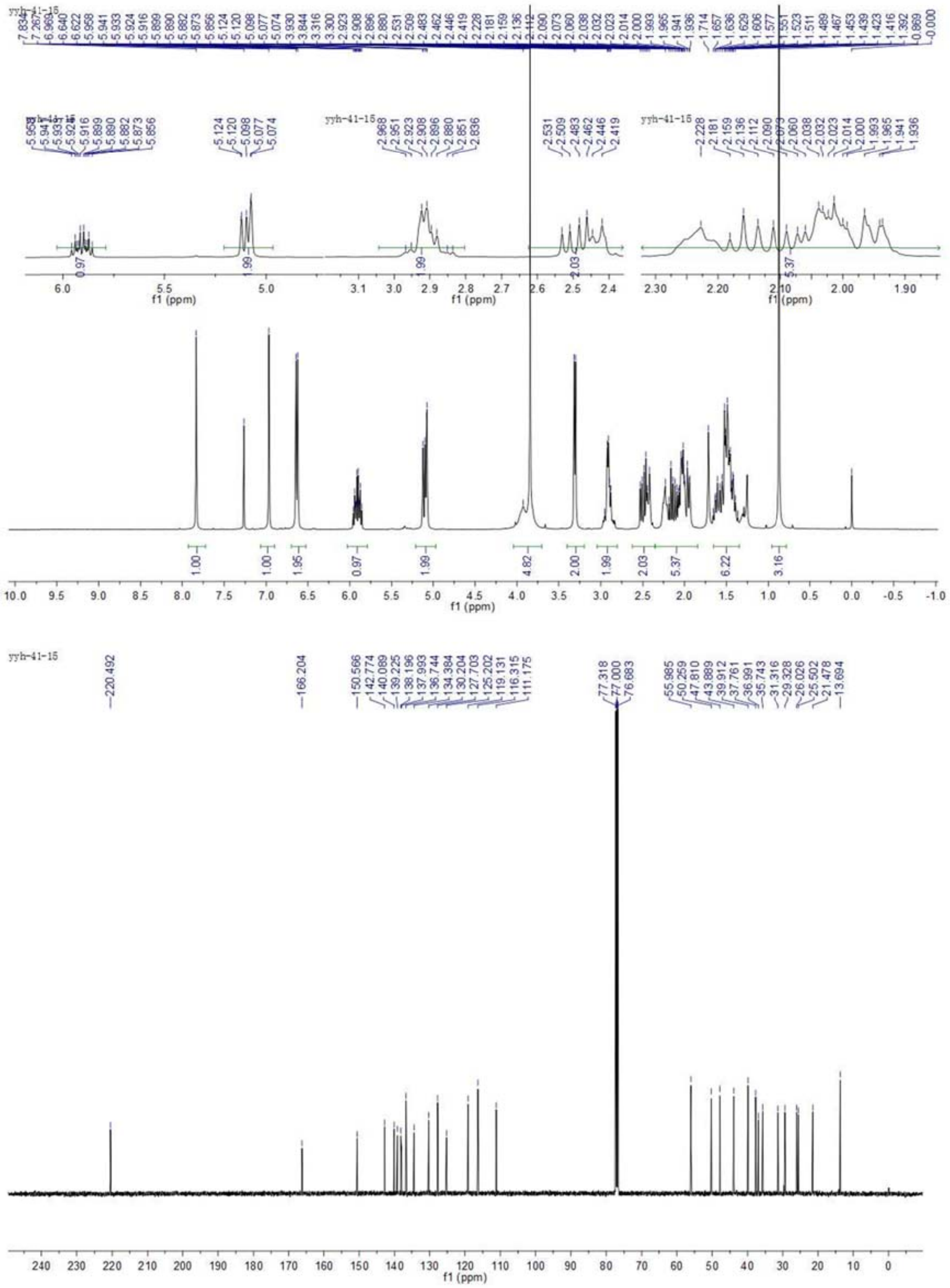

Supplementary Figure 91 ^{1}H NMR and ^{13}C NMR spectra for compound 76

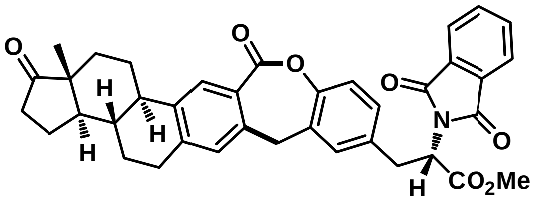

77

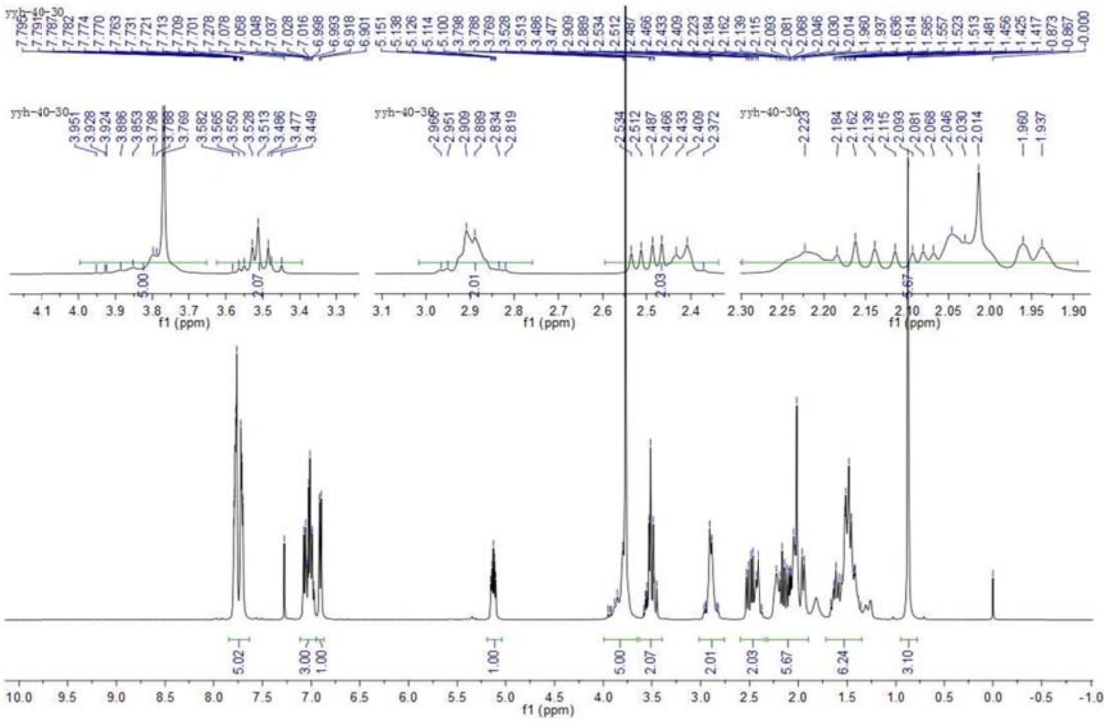

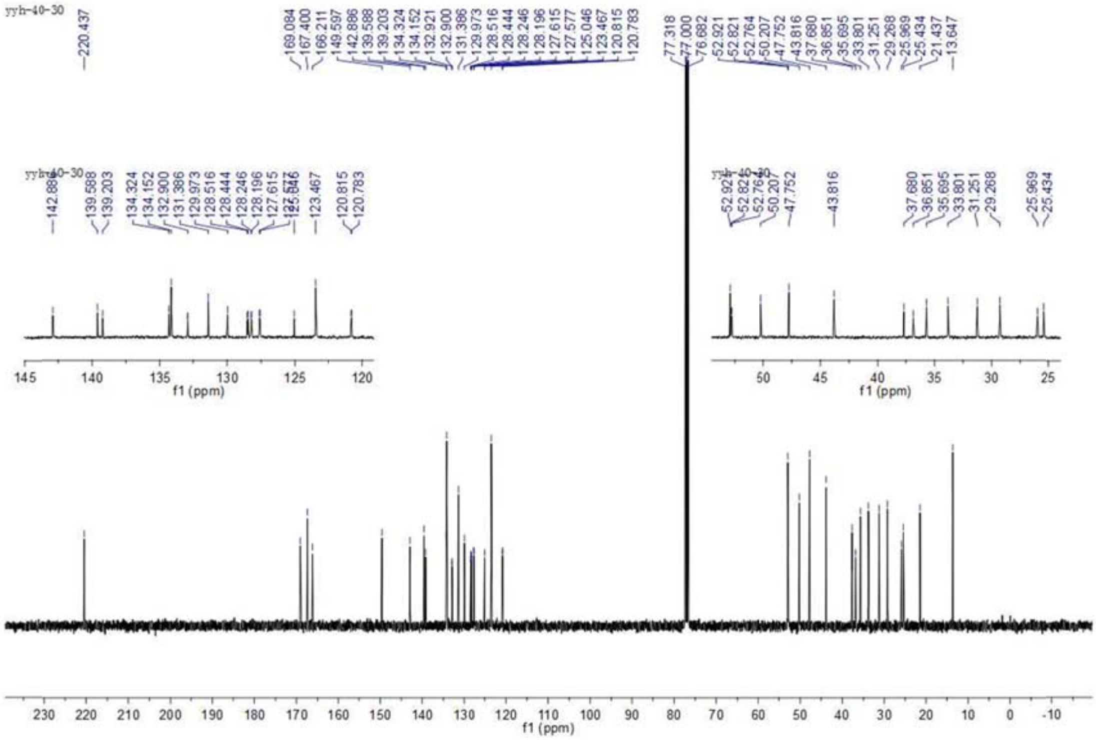

Supplementary Figure 92 ^{1}H NMR and ^{13}C NMR spectra for compound 77

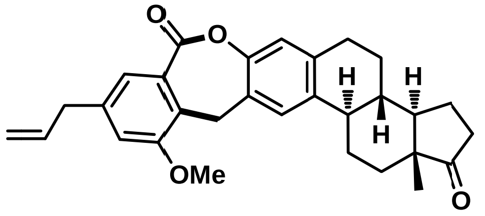

78

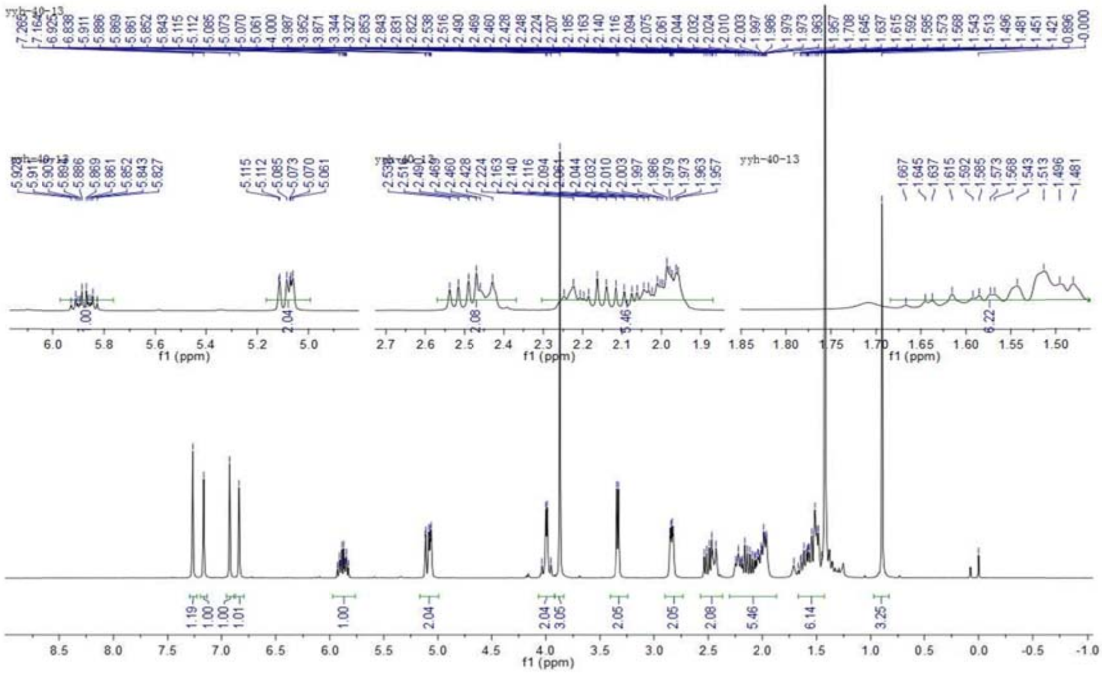

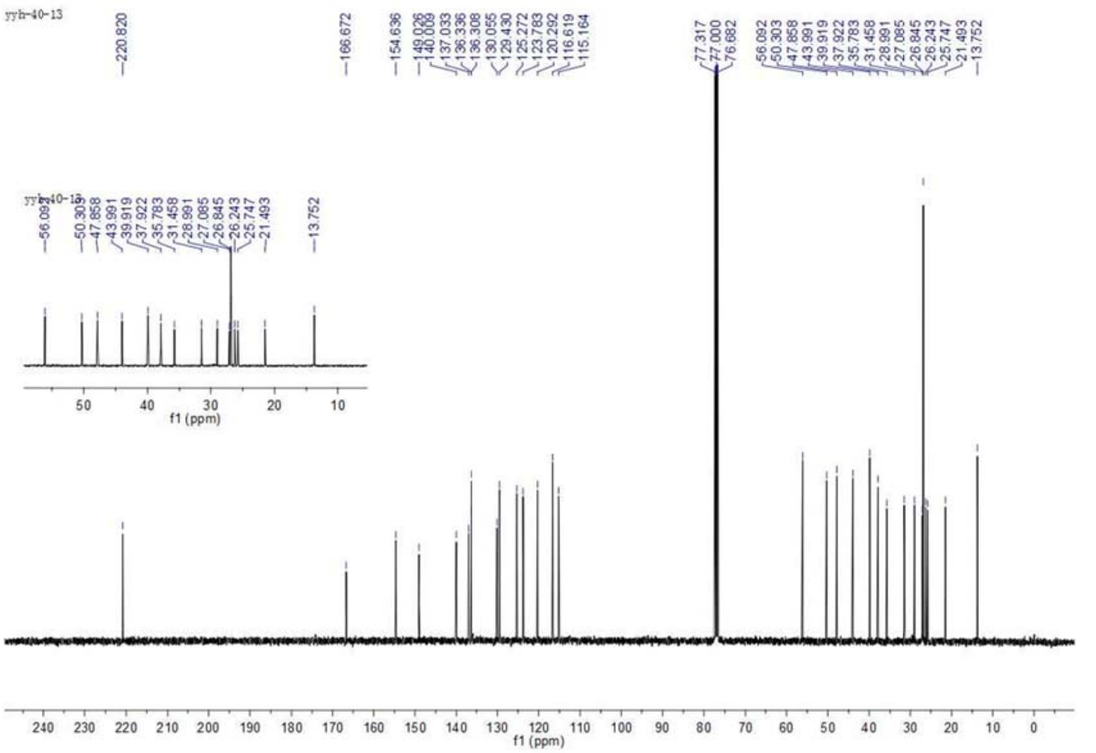

Supplementary Figure 93 ^{1}H NMR and ^{13}C NMR spectra for compound 78

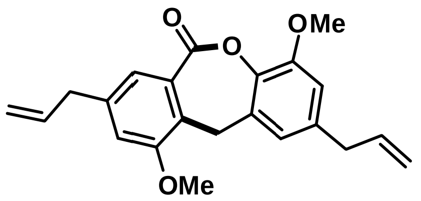

79

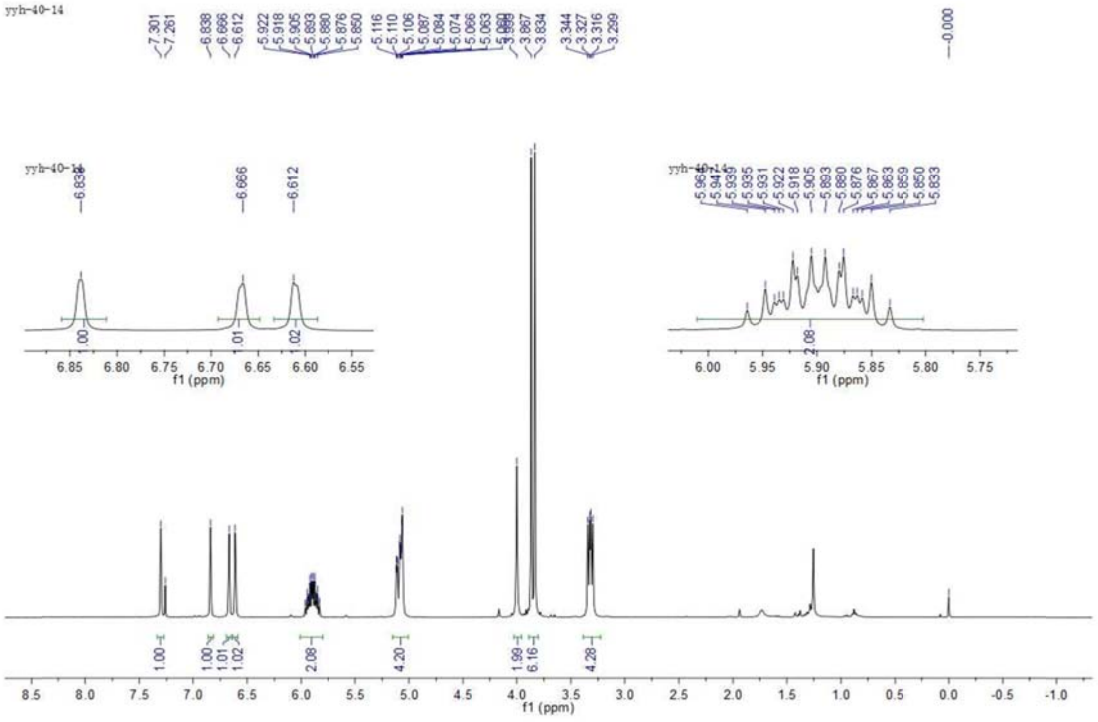

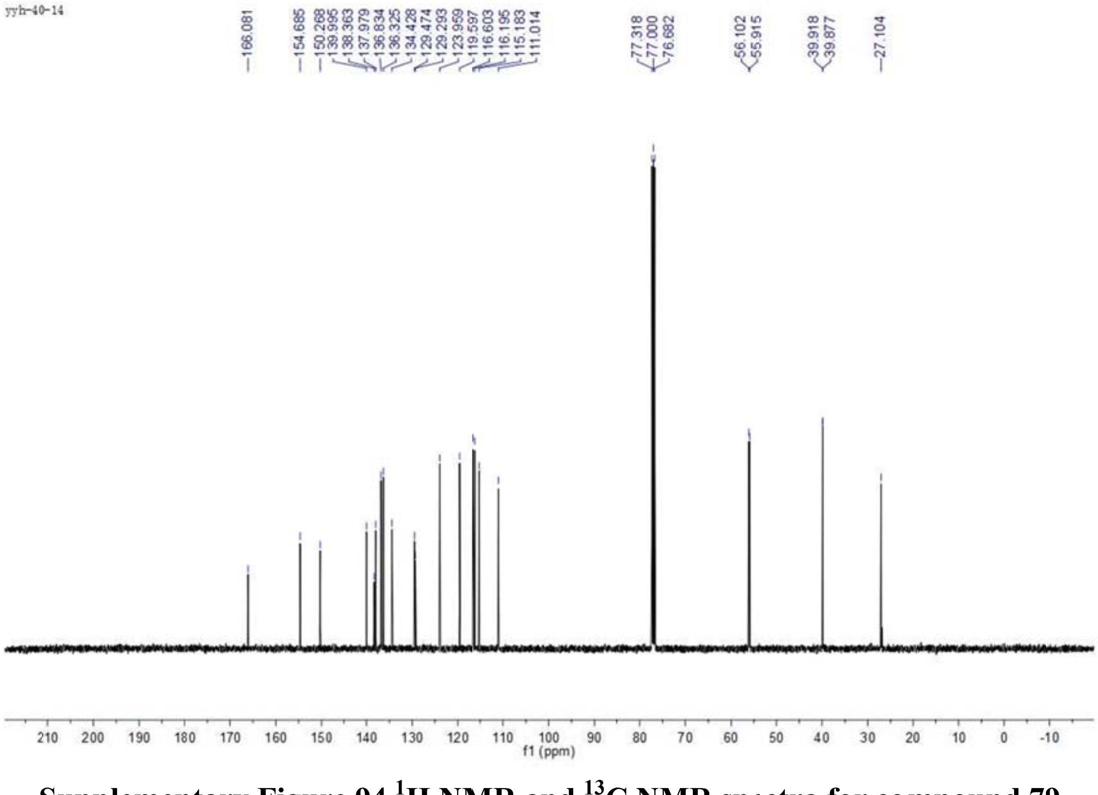

Supplementary Figure 94 ^{1}H NMR and ^{13}C NMR spectra for compound 79

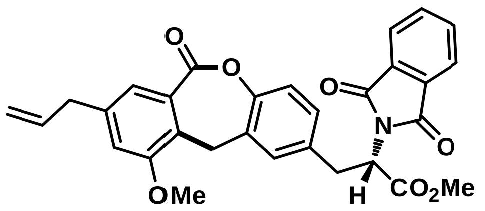

80

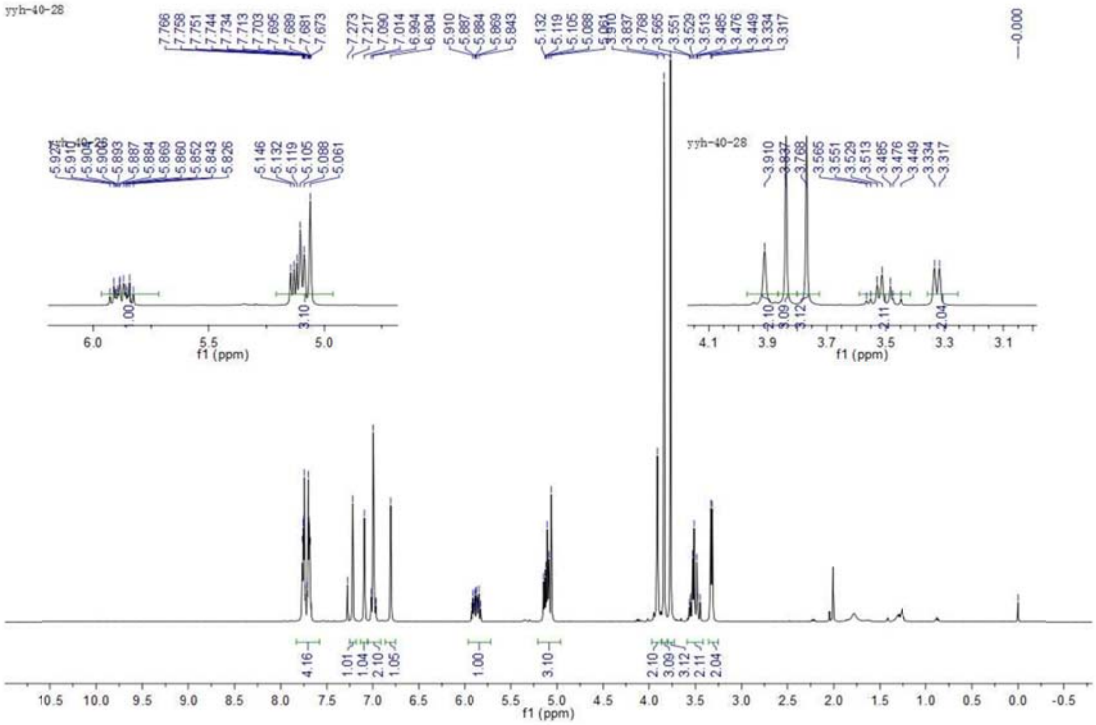

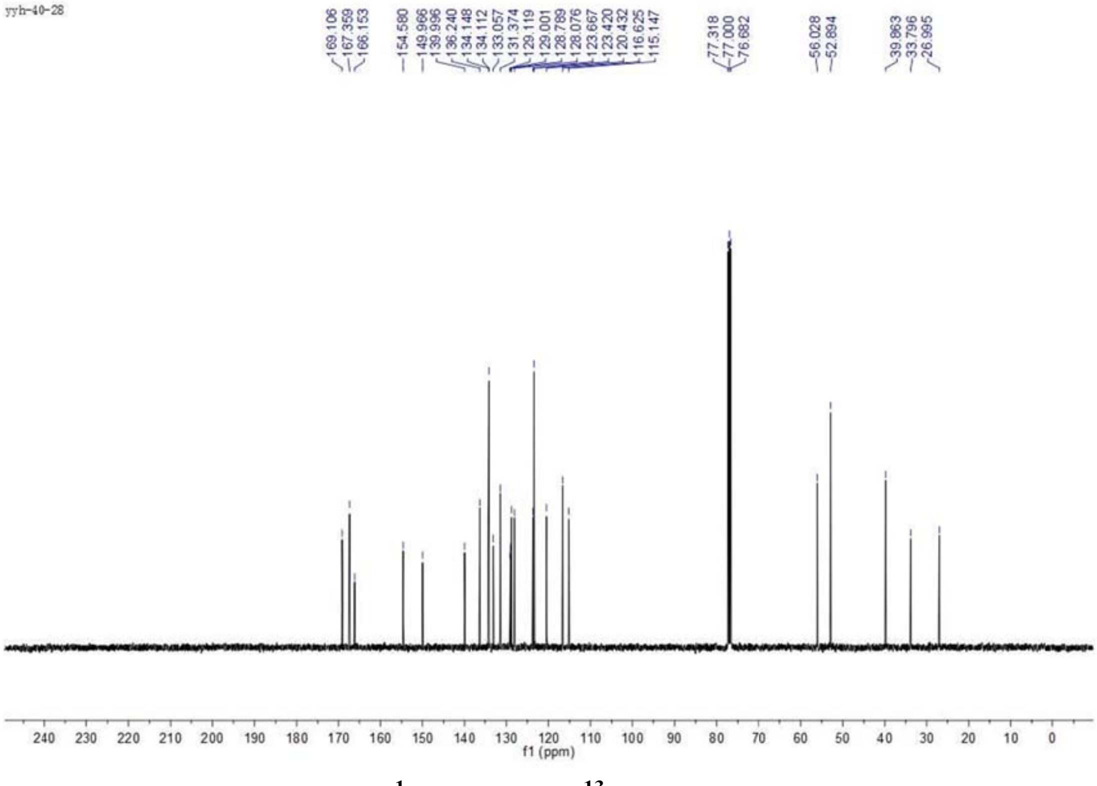

Supplementary Figure 95 ^{1}H NMR and ^{13}C NMR spectra for compound 80

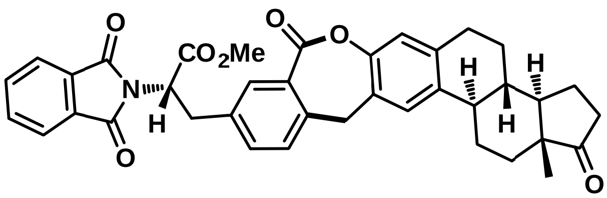

81

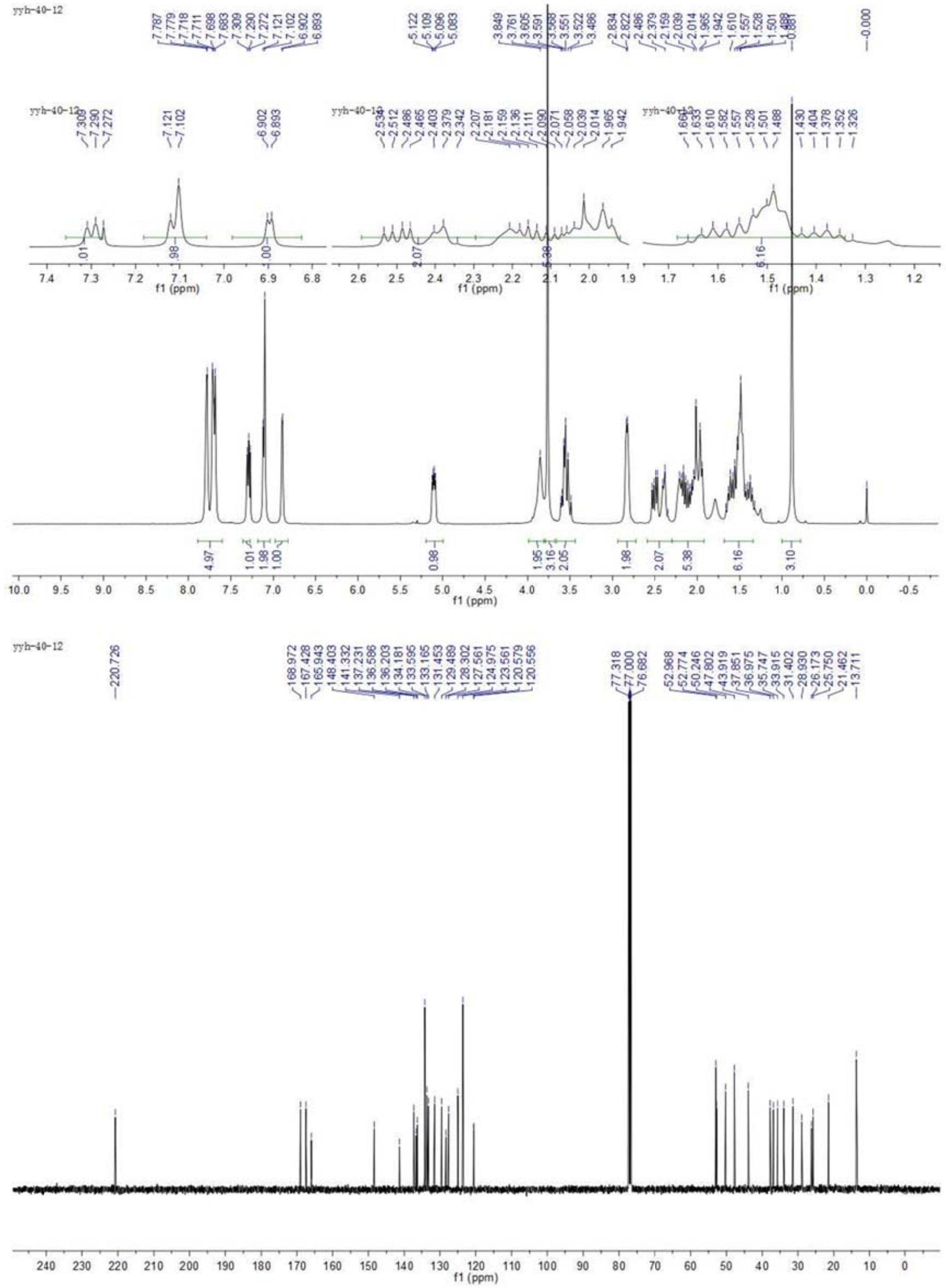

Supplementary Figure 96 ^{1}H NMR and ^{13}C NMR spectra for compound 81

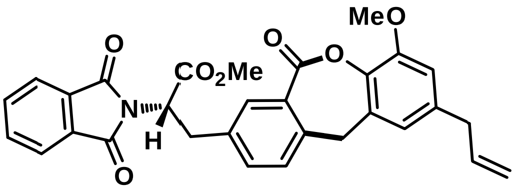

82

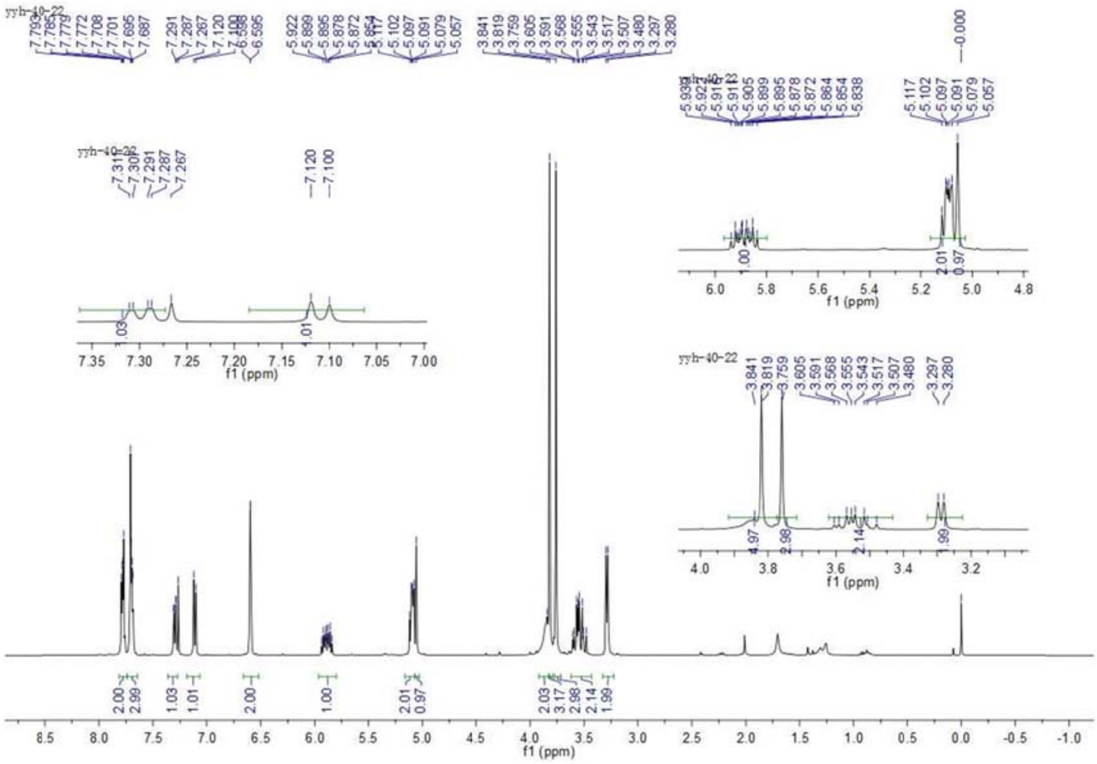

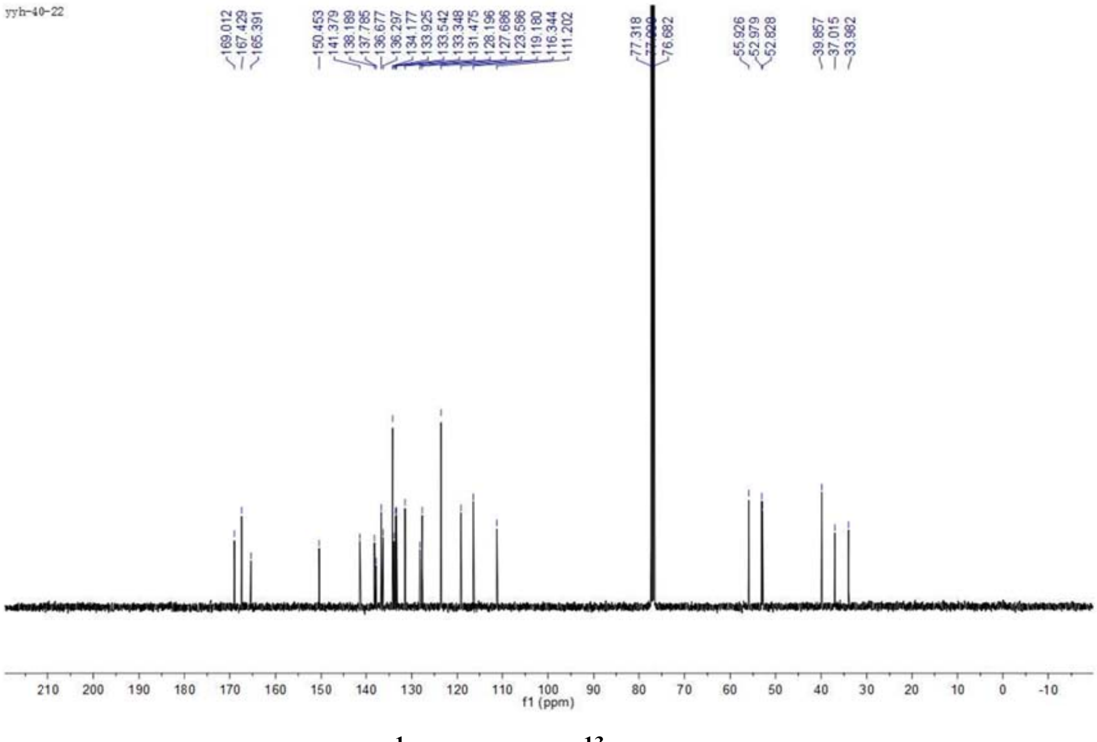

Supplementary Figure 97 ^{1}H NMR and ^{13}C NMR spectra for compound 82

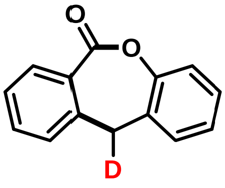

[D]-3

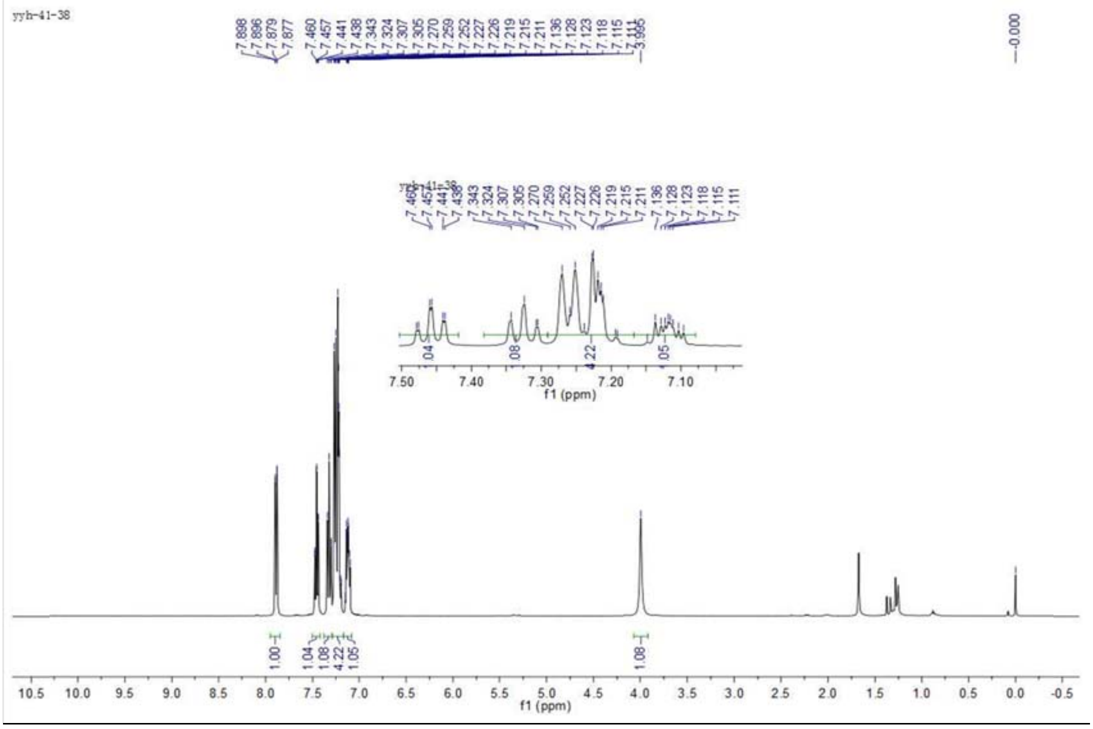

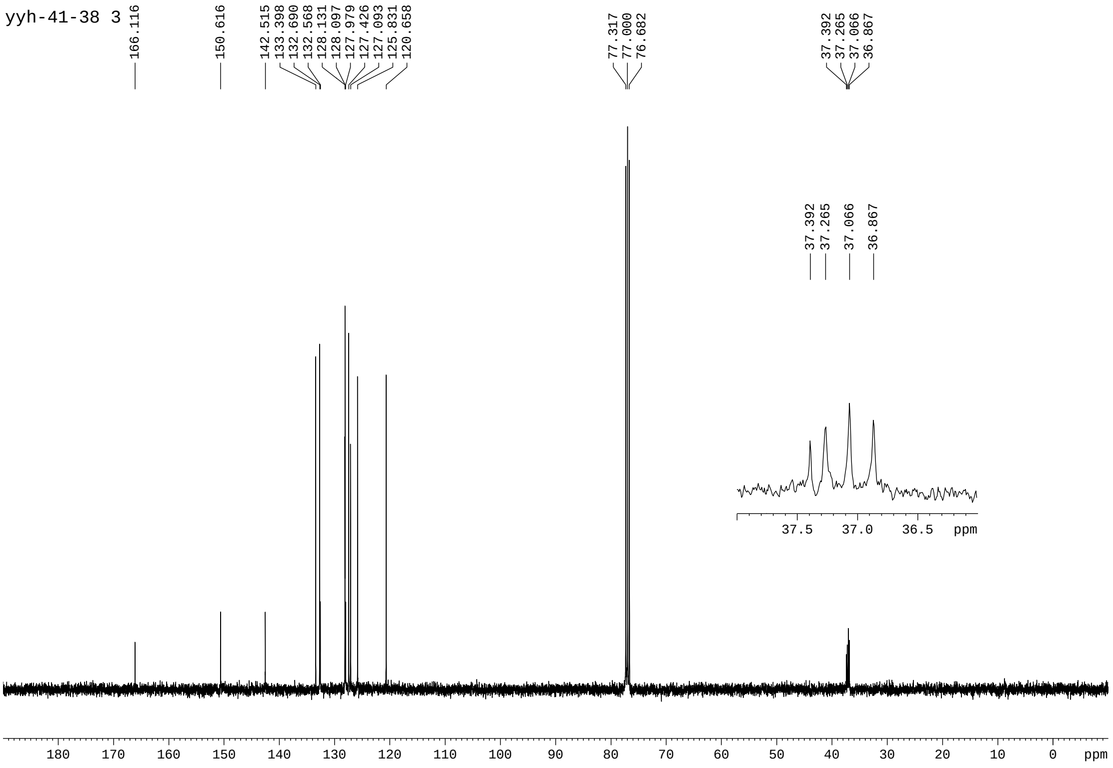

Supplementary Figure 98 ^{1}H NMR and ^{13}C NMR spectra for compound [D]-3

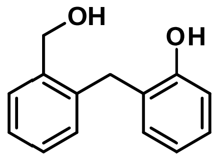

83

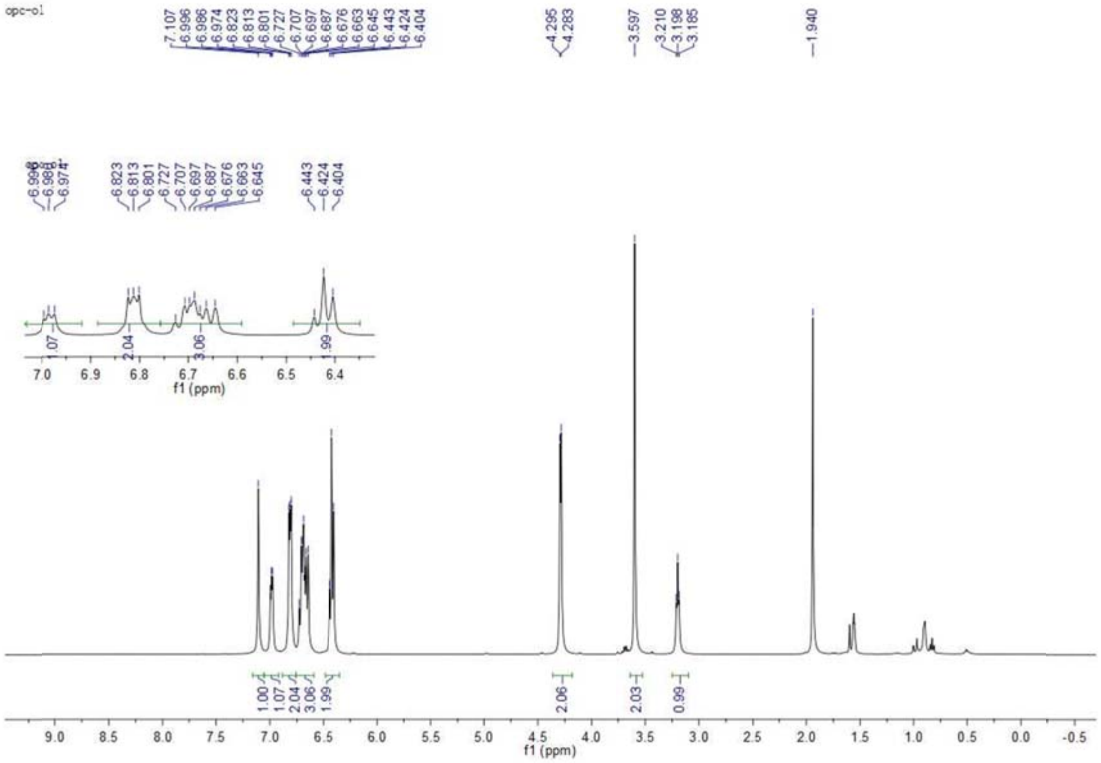

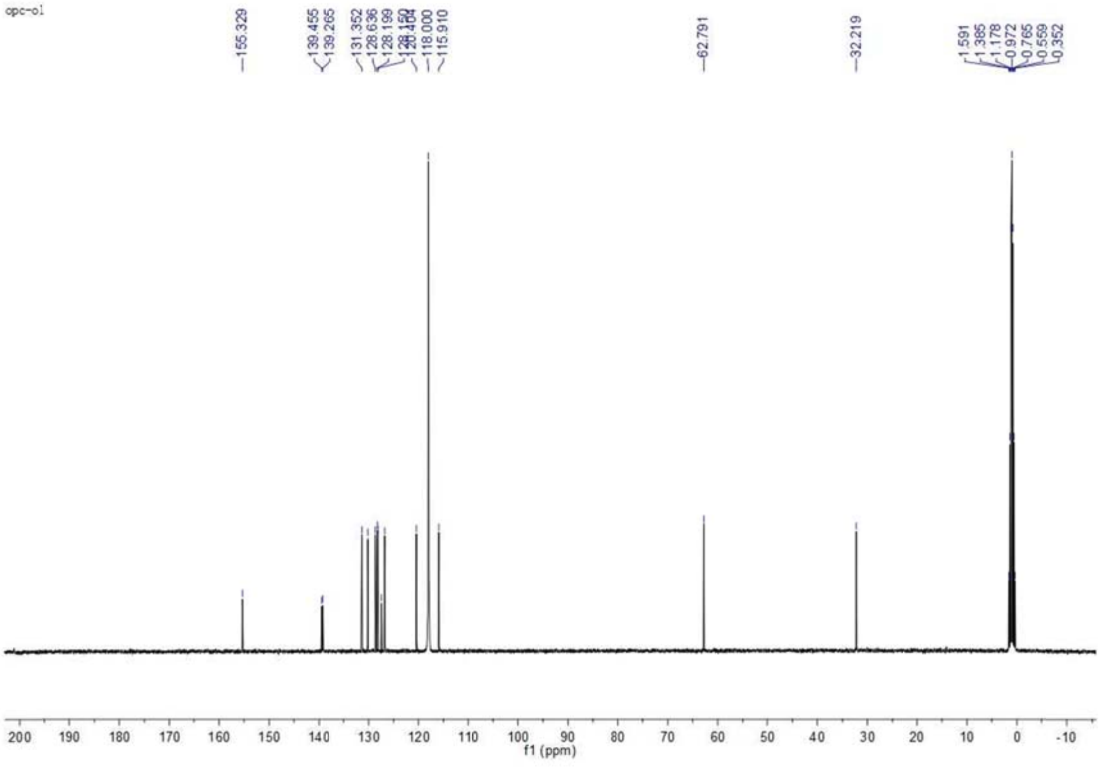

Supplementary Figure 99 ^{1}H NMR and ^{13}C NMR spectra for compound 83

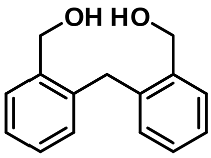

84

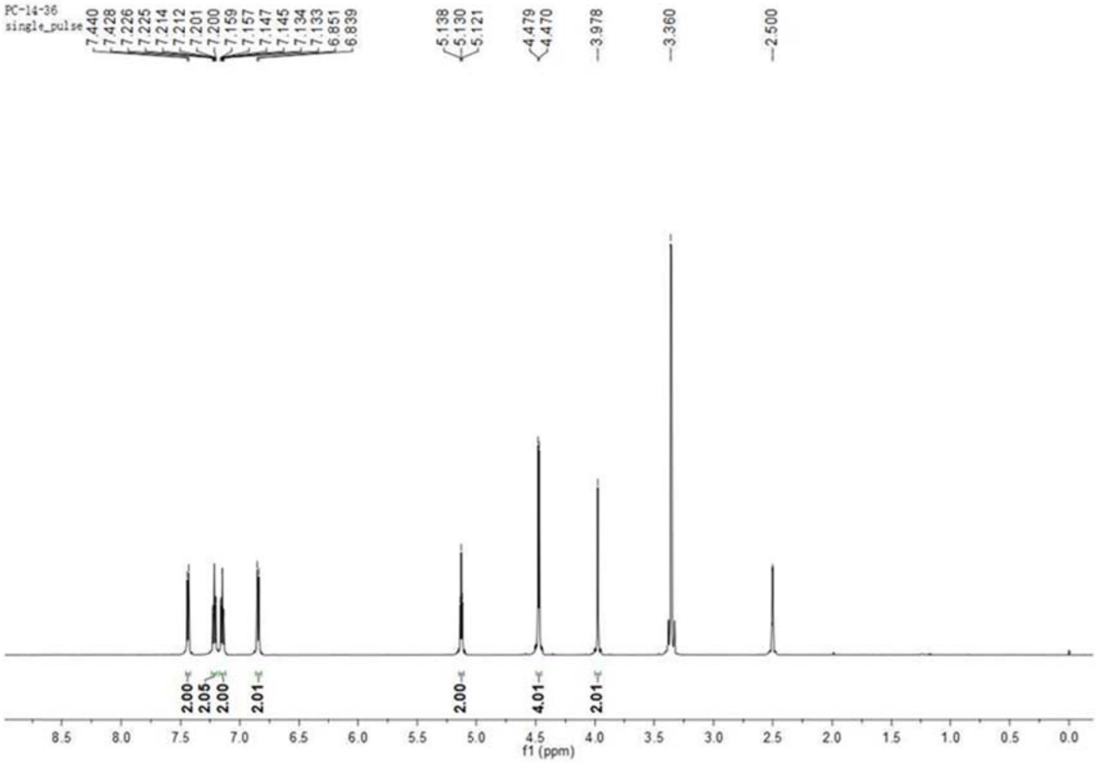

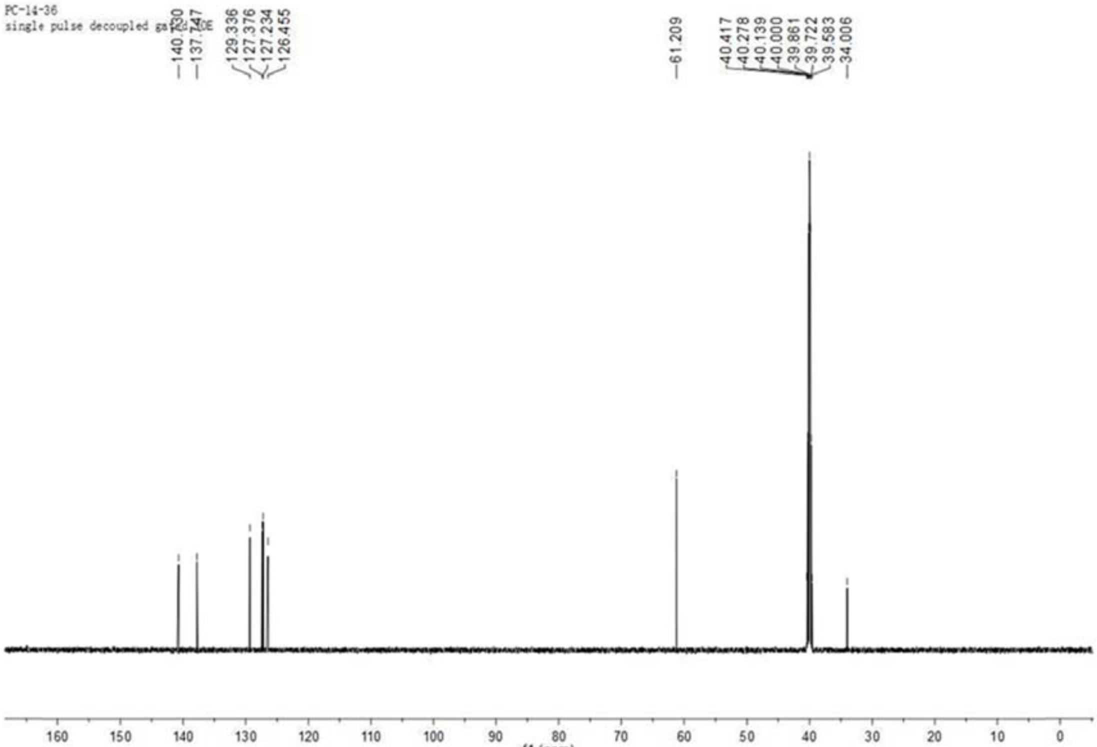

Supplementary Figure 100 ^{1}H NMR and ^{13}C NMR spectra for compound 84

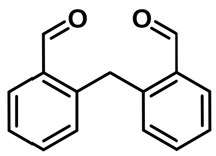

S84

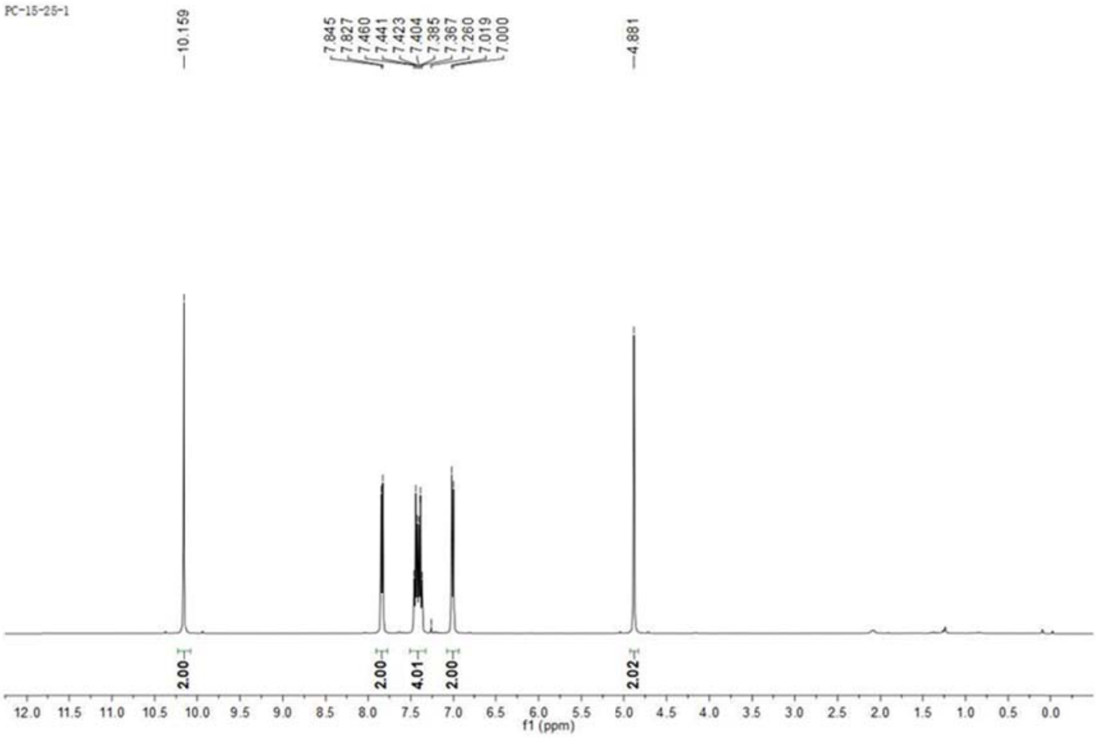

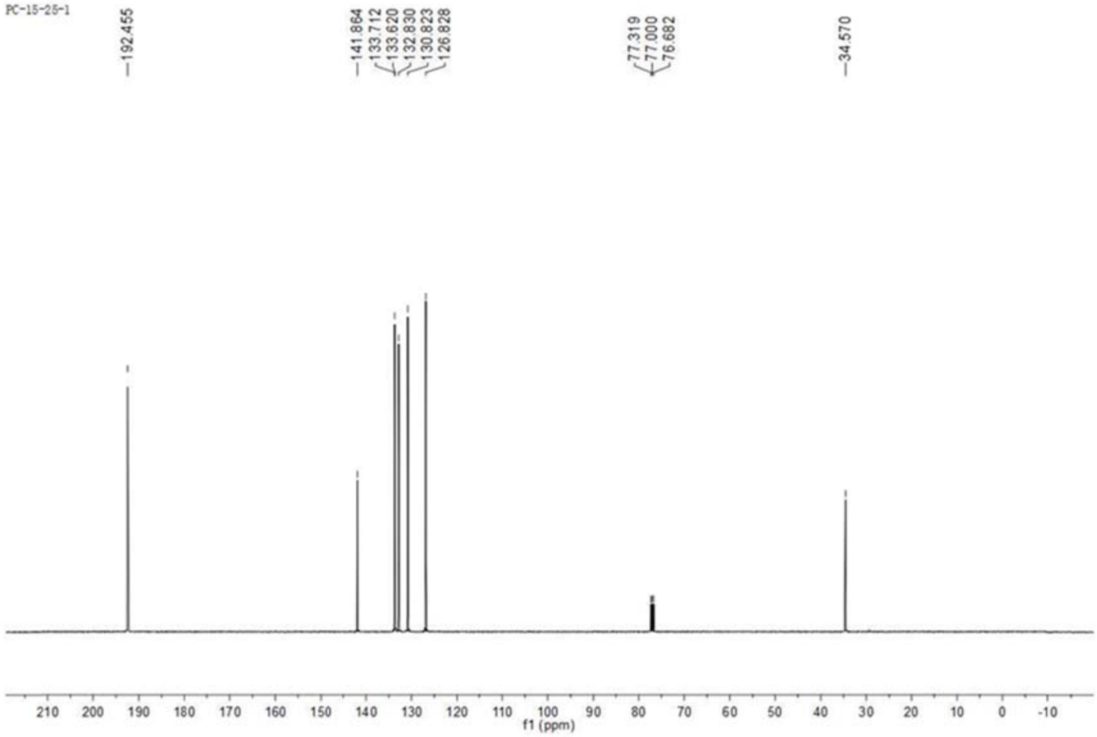

Supplementary Figure 101 ^{1}H NMR and ^{13}C NMR spectra for compound S84

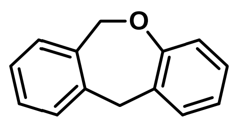

85

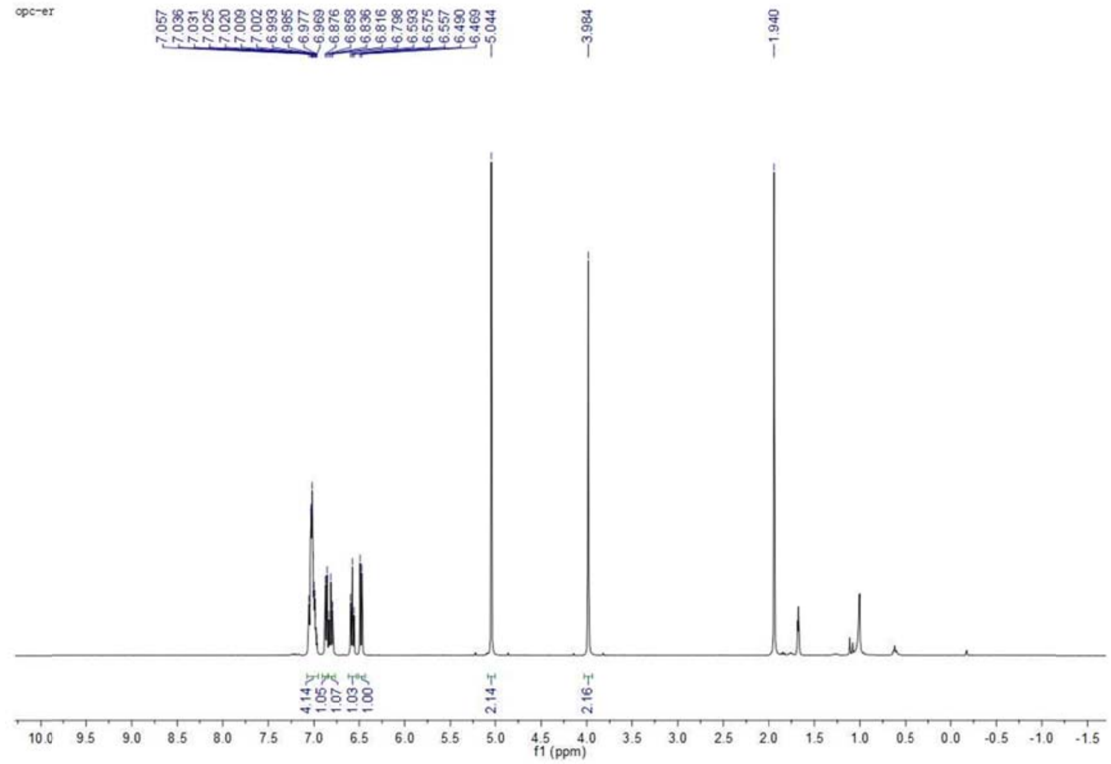

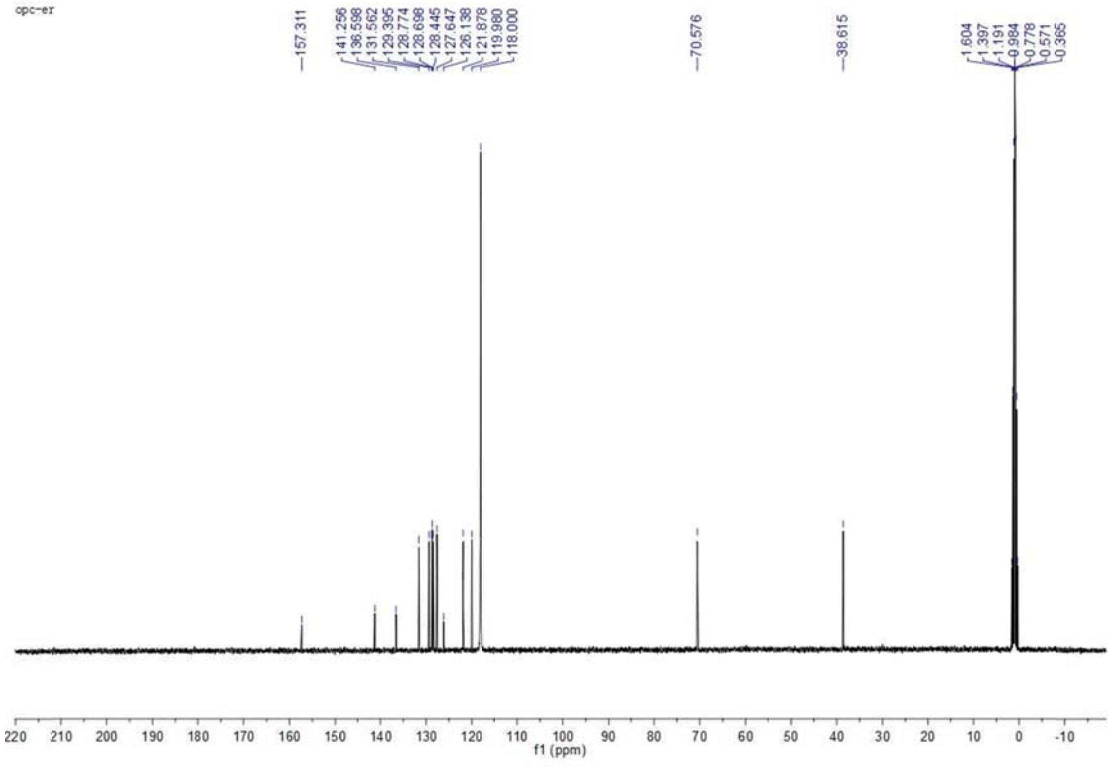

Supplementary Figure 102 ^{1}H NMR and ^{13}C NMR spectra for compound 85

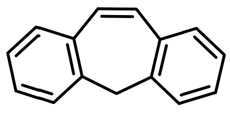

86

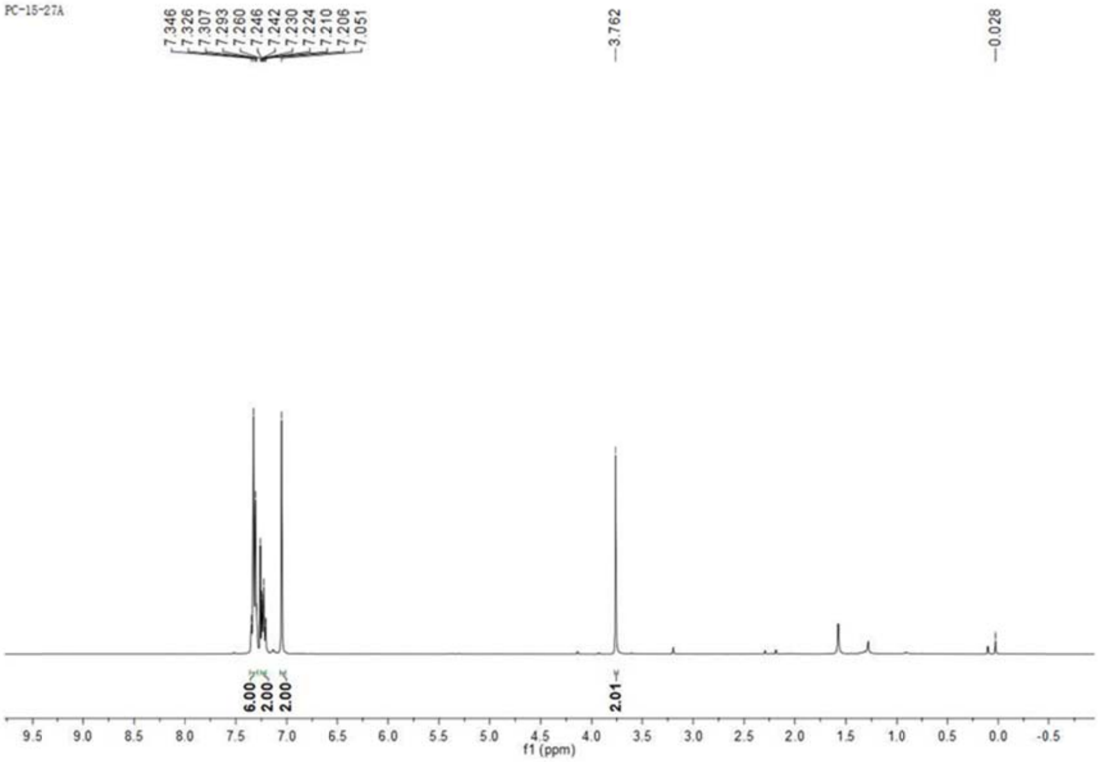

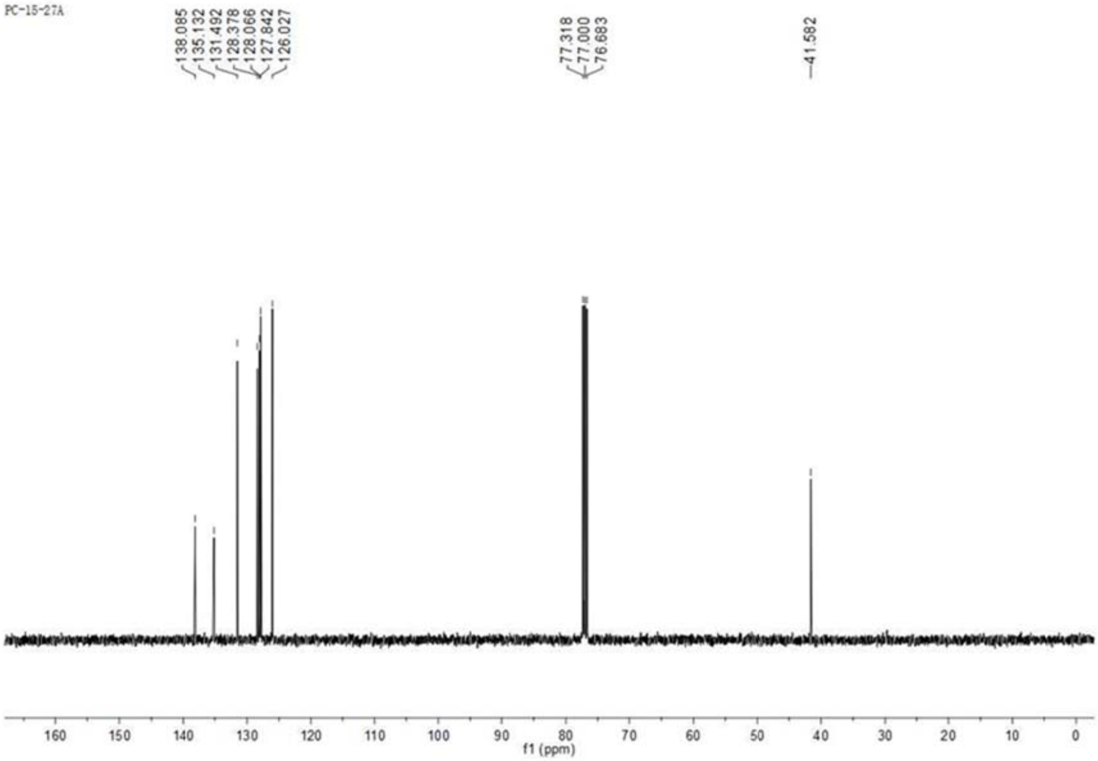

Supplementary Figure 102 ^{1}H NMR and ^{13}C NMR spectra for compound 86

Supplementary References

1. Ma, C., Lo, A., Abdolmaleki, A. & MacLachlan, M. J. Synthesis and metalation of novel fluorescent conjugated macrocycles. *Org. Lett.* **6**, 3841-3844 (2004).

2. Casiraghi, G., Casnati, G., Puglia, G., Sartori, G., Terenghi, G. Selective reactions between phenols and formaldehyde. A novel route to salicylaldehydes. *J. Chem. Soc., Perkin Trans. 1*, 1862-1865 (1980).

3. Hansen, T. V.; Skattebøl, L. Ortho-formylation of phenols; preparation of 3-bromosalicylalehyde. *Org. Synth.* **82**, 64-68 (2005).

4. Hofsløkken, N. U., Skattebøl, L. Convenient method for the ortho-Formylation of phenols. *Acta Chem. Scand.* **53**, 258-262 (1999).

5. Sun, P., Gao, S., Yang, C., Guo, S., Lin, A., Yao, H. Controllable Rh(III)-catalyzed annulation between salicylaldehydes and diazo compounds: divergent synthesis of chromones and benzofurans. *Org. Lett.* **18**, 6464-6467 (2016).

6. Mori, K., Ichikawa, Y., Kobayashi, M., Shibata, Y., Yamanaka, M., Akiyama, T. Enantioselective synthesis of multisubstituted biaryl skeleton by chiral phosphoric acid catalyzed desymmetrization/kinetic resolution sequence. *J. Am. Chem. Soc.* **135**, 3964-3970 (2013).

7. Xia, Y., Xia, Y., Zhang, Y., Wang, J. Palladium-catalyzed coupling of *N*-tosylhydrazones and β-bromostyrene derivatives: new approach to 2*H*-chromenes. *Org. Biomol. Chem.* **12**, 9333-9336 (2014).

8. Sayama, M., Inoue, A., Nakamura, S., Jung, S., Ikubo, M., Otani, Y., Uwamizu, A., Kishi, T., Makide, K., Aoki, J., Hirokawa, T., Ohwada, T. Probing the

hydrophobic binding pocket of G-protein-coupled lysophosphatidylserine receptor GPR34/LPS_1 by docking-aided structure-activity analysis. *J. Med. Chem.* **60**, 6384-6399 (2017).

9. Caldwell, C. G., Rupprecht, K. M., Bondy, S. S. & Davis, A. A. Synthesis of the lipophilic side chain of the cyclic hexadepsipeptide antibiotic L-156,602. *J. Org. Chem.* **55**, 2325-2332 (1990).

10. Dinh, T. N., Chen, A. & Chai, C. L. L. A pattern recognition approach to 14-epi-hydrophenanthrene core of the morphine alkaloids based on shikimic acid *Tetrahedron*, **67**, 3363-3368 (2011).

11. Becke, A. D., Density-functional thermochemistry. III. The role of exact exchange. *J. Chem. Phys.* **1993**, *98*, 5648-5652.

12. Lee, C.; Yang, W.; Parr, R. G., Development of the Colle-Salvetti correlation-energy formula into a functional of the electron density. *Phys. Rev. B* **1988**, *37*, 785-789.

13. Hay, P. J.; Wadt, W. R., Ab initio effective core potentials for molecular calculations. Potentials for the transition metal atoms Sc to Hg. *J. Chem. Phys.* **1985**, *82*, 270-283.

14. Wadt, W. R.; Hay, P. J., Ab initio effective core potentials for molecular calculations. Potentials for main group elements Na to Bi. *J. Chem. Phys.* **1985**, *82*, 284-298.

15. Zhao, Y.; Truhlar, D. G., The M06 suite of density functionals for main group thermochemistry, thermochemical kinetics, noncovalent interactions, excited states,

and transition elements: two new functionals and systematic testing of four M06-class functionals and 12 other functionals. *Theor. Chem. Acc.* **2008**, *120*, 215-241.

16. Weigend, F.; Ahlrichs, R., Balanced basis sets of split valence, triple zeta valence and quadruple zeta valence quality for H to Rn: Design and assessment of accuracy. *Phys. Chem. Chem. Phys.* **2005**, *7*, 3297-3305.

17. Marenich, A. V.; Cramer, C. J.; Truhlar, D. G., Universal Solvation Model Based on Solute Electron Density and on a Continuum Model of the Solvent Defined by the Bulk Dielectric Constant and Atomic Surface Tensions. *J. Phys. Chem. B* **2009**, *113*, 6378-6396.

18. M. J. Frisch; G. W. Trucks; H. B. Schlegel; G. E. Scuseria; M. A. Robb; J. R. Cheeseman; G. Scalmani; V. Barone; G. A. Petersson; H. Nakatsuji; X. Li; M. Caricato; A. Marenich; J. Bloino; B. G. Janesko; R. Gomperts; B. Mennucci; H. P. Hratchian; J. V. Ortiz; A. F. Izmaylov; J. L. Sonnenberg; D. Williams-Young; F. Ding; F. Lipparini; F. Egidi; J. Goings; B. Peng; A. Petrone; T. Henderson; D. Ranasinghe; V. G. Zakrzewski; J. Gao; N. Rega; G. Zheng; W. Liang; M. Hada; M. Ehara; K. Toyota; R. Fukuda; J. Hasegawa; M. Ishida; T. Nakajima; Y. Honda; O. Kitao; H. Nakai; T. Vreven; K. Throssell; J. A. Montgomery; Jr., J. E. P.; F. Ogliaro; M. Bearpark; J. J. Heyd; E. Brothers; K. N. Kudin; V. N. Staroverov; T. Keith; R. Kobayashi; J. Normand; K. Raghavachari; A. Rendell; J. C. Burant; S. S. Iyengar; J. Tomasi; M. Cossi; J. M. Millam; M. Klene; C. Adamo; R. Cammi; J. W. Ochterski; R. L. Martin; K. Morokuma; O. Farkas; J. B. Foresman; Fox, D. J. *Gaussian 09*, Revision E.01; Gaussian, Inc., Wallingford CT, 2016.
