# Supplementary material for: Easy access to medium-sized lactones through metal carbene migratory insertion enabled 1,4-palladium shift
Source: Nat Commun. 2020 Jan 23;11:461. doi: 10.1038/s41467-019-14101-5 (PMC6978448; doi:10.1038/s41467-019-14101-5)
Supplement: Supplementary file 3 — Description of Additional Supplementary Files [file 41467_2019_14101_MOESM3_ESM.pdf]

Description of Additional Supplementary Files

File Name: Supplementary Data 1

Description: Easy access to medium-sized lactones through metal carbene migratory insertion enabled 1,4-palladium shift
