# Supplementary material for: Easy access to medium-sized lactones through metal carbene migratory insertion enabled 1,4-palladium shift
Source: Nat Commun. 2020 Jan 23;11:461. doi: 10.1038/s41467-019-14101-5 (PMC6978448; doi:10.1038/s41467-019-14101-5)
Supplement: Supplementary file 4 — Supplementary Data 1 [file 41467_2019_14101_MOESM4_ESM.pdf]

**Int1**

| | | | |
|---|---|---|---|
| Pd | 0.057257 | -0.559010 | 0.214920 |
| Br | -1.881379 | -3.323028 | 0.134593 |
| P | 2.231981 | 0.244313 | -0.454936 |
| P | -1.353922 | 0.887213 | -1.118057 |
| C | 3.240316 | 1.084033 | 0.845033 |
| C | 3.153961 | 2.468199 | 1.067589 |
| H | 2.538957 | 3.090751 | 0.425295 |
| C | 3.849668 | 3.066361 | 2.121014 |
| H | 3.773395 | 4.140212 | 2.271922 |
| C | 4.640910 | 2.294373 | 2.971958 |
| H | 5.183143 | 2.761246 | 3.789725 |
| C | 4.729656 | 0.915438 | 2.764782 |
| H | 5.344481 | 0.304027 | 3.420342 |
| C | 4.033432 | 0.316181 | 1.715870 |
| H | 4.117925 | -0.756384 | 1.563850 |
| C | 3.421223 | -0.980687 | -1.167205 |
| C | 2.979162 | -2.290108 | -1.405934 |
| H | 1.958493 | -2.560802 | -1.146026 |
| C | 3.835155 | -3.240140 | -1.969928 |
| H | 3.477444 | -4.250820 | -2.147657 |
| C | 5.145172 | -2.890905 | -2.298888 |
| H | 5.813664 | -3.629025 | -2.734145 |
| C | 5.598084 | -1.589064 | -2.064309 |
| H | 6.618838 | -1.313837 | -2.316928 |
| C | 4.742842 | -0.640795 | -1.503861 |
| H | 5.109492 | 0.364760 | -1.314685 |
| C | -1.243253 | 2.647924 | -0.555435 |
| C | -1.732419 | 3.724002 | -1.316553 |
| H | -2.215030 | 3.540472 | -2.272842 |
| C | -1.625488 | 5.033871 | -0.849327 |
| H | -2.009160 | 5.854371 | -1.450124 |
| C | -1.032956 | 5.290254 | 0.390626 |
| H | -0.952039 | 6.311251 | 0.754198 |
| C | -0.551707 | 4.231068 | 1.160806 |
| H | -0.095014 | 4.420579 | 2.128474 |
| C | -0.655927 | 2.918953 | 0.689708 |
| H | -0.277757 | 2.091424 | 1.285493 |
| C | -3.181340 | 0.617743 | -1.236877 |
| C | -3.658373 | -0.490165 | -1.960077 |
| H | -2.962664 | -1.144033 | -2.479581 |

| | | | |
|---|---|---|---|
| C | -5.020730 | -0.783510 | -2.001240 |
| H | -5.368060 | -1.643259 | -2.568197 |
| C | -5.932855 | 0.013524 | -1.304761 |
| H | -6.993624 | -0.220347 | -1.329147 |
| C | -5.469828 | 1.102784 | -0.566794 |
| H | -6.168150 | 1.721531 | -0.009926 |
| C | -4.106322 | 1.403531 | -0.532029 |
| H | -3.766882 | 2.254519 | 0.049185 |
| C | -1.128462 | -1.937354 | 1.433603 |
| C | -2.036803 | -1.330435 | 2.366196 |
| C | -1.533769 | -0.864937 | 3.575416 |
| H | -2.234963 | -0.370770 | 4.241944 |
| C | -0.181365 | -1.026571 | 3.931644 |
| C | 0.683628 | -1.672984 | 3.067855 |
| H | 1.714548 | -1.858728 | 3.359047 |
| C | 0.238595 | -2.155054 | 1.805103 |
| H | 0.830626 | -2.904752 | 1.290805 |
| H | 0.170782 | -0.670970 | 4.895690 |
| C | -3.476733 | -1.126358 | 2.080670 |
| O | -4.253624 | -0.605454 | 2.865566 |
| H | -3.824977 | -1.473427 | 1.091335 |
| C | -0.867098 | 0.970466 | -2.945169 |
| H | -0.826410 | 2.020005 | -3.260651 |
| H | -1.698772 | 0.517573 | -3.494122 |
| C | 0.435335 | 0.238734 | -3.328799 |
| H | 0.354546 | -0.047373 | -4.386424 |
| H | 0.500940 | -0.700847 | -2.766842 |
| C | 1.748258 | 1.031012 | -3.191986 |
| H | 1.699925 | 1.903450 | -3.859604 |
| H | 2.559402 | 0.400816 | -3.577032 |
| C | 2.151379 | 1.548769 | -1.797658 |
| H | 3.122262 | 2.054146 | -1.875780 |
| H | 1.429063 | 2.296542 | -1.450583 |

**TS1**

| | | | |
|---|---|---|---|
| Pd | 0.161410 | 0.486443 | 0.031934 |
| Br | 0.433102 | 3.025779 | -1.154134 |
| P | 2.361845 | -0.396265 | 0.762294 |
| P | -1.704016 | -0.482137 | 1.240441 |
| C | 3.020102 | -1.535822 | -0.537706 |
| C | 2.602028 | -2.875675 | -0.597521 |
| H | 1.939337 | -3.276943 | 0.164283 |
| C | 3.021213 | -3.709315 | -1.635883 |
| H | 2.691564 | -4.744944 | -1.658822 |
| C | 3.859692 | -3.218231 | -2.638281 |
| H | 4.186972 | -3.867785 | -3.445574 |
| C | 4.274163 | -1.885271 | -2.595090 |
| H | 4.928822 | -1.492261 | -3.368734 |
| C | 3.856992 | -1.050687 | -1.557095 |
| H | 4.195419 | -0.018742 | -1.533479 |
| C | 3.794859 | 0.723795 | 1.089523 |
| C | 3.578355 | 2.108516 | 1.115839 |
| H | 2.588615 | 2.498168 | 0.896346 |
| C | 4.626603 | 2.985175 | 1.411622 |
| H | 4.444328 | 4.056392 | 1.425583 |
| C | 5.900381 | 2.486558 | 1.683240 |
| H | 6.715711 | 3.167740 | 1.912414 |
| C | 6.128254 | 1.107015 | 1.656565 |
| H | 7.120221 | 0.714273 | 1.864739 |
| C | 5.083899 | 0.231957 | 1.361762 |
| H | 5.273817 | -0.837793 | 1.333815 |
| C | -2.146007 | -2.189599 | 0.669863 |
| C | -3.016582 | -3.025246 | 1.391608 |
| H | -3.482733 | -2.666455 | 2.305377 |
| C | -3.310123 | -4.311315 | 0.939049 |
| H | -3.985777 | -4.943371 | 1.509571 |
| C | -2.740113 | -4.784365 | -0.246413 |
| H | -2.968976 | -5.786888 | -0.598405 |
| C | -1.881191 | -3.962859 | -0.976375 |
| H | -1.438331 | -4.320021 | -1.902378 |
| C | -1.586242 | -2.674410 | -0.521415 |
| H | -0.915869 | -2.038154 | -1.092434 |
| C | -3.348376 | 0.361667 | 1.337803 |
| C | -3.429874 | 1.587560 | 2.023959 |
| H | -2.550543 | 1.994509 | 2.518746 |
| C | -4.623648 | 2.304890 | 2.067437 |
| H | -4.665724 | 3.248223 | 2.605538 |
| C | -5.756176 | 1.822310 | 1.406969 |
| H | -6.683158 | 2.388352 | 1.427466 |
| C | -5.683454 | 0.618480 | 0.708032 |
| H | -6.555439 | 0.241323 | 0.180469 |
| C | -4.490855 | -0.107576 | 0.672887 |
| H | -4.453701 | -1.041135 | 0.121440 |
| C | -0.673098 | 1.234847 | -1.834019 |
| C | -2.054711 | 1.401216 | -2.086367 |
| C | -2.644133 | 0.605893 | -3.079434 |

| | | | |
|---|---|---|---|
| H | -3.712550 | 0.727353 | -3.234696 |
| C | -1.894279 | -0.290838 | -3.837827 |
| C | -0.512358 | -0.372550 | -3.637704 |
| H | 0.094664 | -1.028726 | -4.256668 |
| C | 0.115437 | 0.421506 | -2.673262 |
| H | 1.195697 | 0.422110 | -2.580171 |
| H | -2.373162 | -0.891056 | -4.605988 |
| C | -2.904113 | 2.379041 | -1.365990 |
| O | -4.073400 | 2.587425 | -1.642117 |
| H | -2.402526 | 2.939791 | -0.557085 |
| C | 2.382521 | -1.484091 | 2.309325 |
| H | 2.868831 | -0.900594 | 3.099576 |
| H | 3.016824 | -2.357955 | 2.122260 |
| C | 0.973712 | -1.903588 | 2.760105 |
| H | 1.055889 | -2.722181 | 3.487658 |
| H | 0.428689 | -2.319182 | 1.904748 |
| C | 0.179315 | -0.733325 | 3.381441 |
| H | 0.589346 | 0.218088 | 3.021002 |
| H | 0.323276 | -0.734596 | 4.470658 |
| C | -1.323717 | -0.739016 | 3.075145 |
| H | -1.793185 | -1.668533 | 3.417686 |
| H | -1.810882 | 0.079081 | 3.615968 |

**Int2**

| | | | |
|---|---|---|---|
| Pd | -0.133360 | -0.576090 | 0.324573 |
| Br | 1.184379 | -2.174607 | 1.892550 |
| P | 1.998701 | 0.346886 | -0.497165 |
| P | -1.485336 | 0.898642 | -0.895483 |
| C | 2.984858 | 1.278943 | 0.751740 |
| C | 3.637309 | 0.569952 | 1.776429 |
| H | 3.566033 | -0.511806 | 1.813802 |
| C | 4.356604 | 1.250403 | 2.758130 |
| H | 4.854830 | 0.685840 | 3.541561 |
| C | 4.432928 | 2.645200 | 2.740868 |
| H | 4.994415 | 3.172027 | 3.507980 |
| C | 3.781791 | 3.358028 | 1.734253 |
| H | 3.834085 | 4.443581 | 1.710485 |
| C | 3.061943 | 2.681197 | 0.746276 |
| H | 2.563283 | 3.260271 | -0.023533 |
| C | 3.170748 | -0.901750 | -1.173053 |
| C | 2.702465 | -2.183575 | -1.496910 |
| H | 1.673956 | -2.455090 | -1.280558 |
| C | 3.562947 | -3.118149 | -2.077854 |
| H | 3.190785 | -4.109872 | -2.319516 |
| C | 4.894112 | -2.786045 | -2.331980 |
| H | 5.563294 | -3.517884 | -2.776997 |
| C | 5.368920 | -1.512745 | -2.006083 |
| H | 6.406800 | -1.251389 | -2.195615 |
| C | 4.512119 | -0.574595 | -1.431025 |
| H | 4.892919 | 0.409122 | -1.169650 |
| C | -1.295593 | 2.634303 | -0.295887 |
| C | -1.804122 | 3.730509 | -1.015357 |
| H | -2.331510 | 3.573509 | -1.952363 |
| C | -1.656520 | 5.028405 | -0.527532 |
| H | -2.053812 | 5.865934 | -1.094675 |
| C | -1.004146 | 5.250809 | 0.688974 |
| H | -0.890186 | 6.263127 | 1.067296 |
| C | -0.502862 | 4.170616 | 1.415390 |
| H | 0.007768 | 4.333643 | 2.360176 |
| C | -0.647912 | 2.869341 | 0.926153 |
| H | -0.249732 | 2.028726 | 1.488902 |
| C | -3.315831 | 0.651003 | -0.894157 |
| C | -3.860188 | -0.406484 | -1.642491 |
| H | -3.207711 | -1.067353 | -2.205994 |
| C | -5.235513 | -0.633589 | -1.652338 |
| H | -5.639970 | -1.453548 | -2.239727 |
| C | -6.088276 | 0.183121 | -0.906297 |
| H | -7.160261 | 0.005774 | -0.914909 |
| C | -5.554692 | 1.221945 | -0.143849 |
| H | -6.208877 | 1.857992 | 0.446491 |
| C | -4.177405 | 1.454115 | -0.134133 |
| H | -3.780112 | 2.266951 | 0.464306 |
| C | -1.784425 | -1.456621 | 1.141060 |
| C | -2.332978 | -2.637917 | 0.591798 |
| C | -3.380672 | -3.311292 | 1.251674 |
| H | -3.773275 | -4.229375 | 0.816557 |
| C | -3.911976 | -2.816331 | 2.435246 |
| C | -3.382062 | -1.638665 | 2.972115 |
| H | -3.782315 | -1.239857 | 3.901930 |
| C | -2.330252 | -0.973246 | 2.334557 |
| H | -1.921022 | -0.078382 | 2.795374 |
| H | -4.721823 | -3.337212 | 2.938326 |
| C | -1.875364 | -3.218496 | -0.676267 |
| O | -1.090305 | -2.721083 | -1.475641 |
| H | -2.333469 | -4.204176 | -0.914487 |
| C | 1.875159 | 1.564373 | -1.910200 |

| | | | |
|---|---|---|---|
| H | 1.171875 | 2.345598 | -1.598842 |
| H | 2.849553 | 2.048263 | -2.047300 |
| C | 1.428593 | 0.948935 | -3.247468 |
| H | 1.365052 | 1.767977 | -3.978217 |
| H | 2.214956 | 0.276309 | -3.609441 |
| C | 0.098387 | 0.179844 | -3.249092 |
| H | -0.101987 | -0.117236 | -4.286865 |
| H | 0.183981 | -0.754093 | -2.684154 |
| C | -1.126325 | 0.968855 | -2.747387 |
| H | -1.058492 | 2.023344 | -3.041070 |
| H | -2.026382 | 0.574004 | -3.225896 |

**Int3**

| | | | |
|---|---|---|---|
| Pd | -3.105990 | -0.900111 | -0.960149 |
| Br | -4.891141 | -2.015729 | -2.318753 |
| P | 5.023076 | -0.632218 | -1.223143 |
| P | -1.156776 | -0.104934 | 0.040960 |
| C | 6.624254 | -1.070718 | -0.391567 |
| C | 6.933926 | -2.436905 | -0.259389 |
| H | 6.226781 | -3.189264 | -0.603071 |
| C | 8.143538 | -2.850148 | 0.296807 |
| H | 8.356700 | -3.911498 | 0.395226 |
| C | 9.083166 | -1.903888 | 0.713730 |
| H | 10.029577 | -2.223787 | 1.141270 |
| C | 8.800251 | -0.546189 | 0.566088 |
| H | 9.527343 | 0.198918 | 0.879222 |
| C | 7.582614 | -0.132210 | 0.019138 |
| H | 7.382155 | 0.929196 | -0.084473 |
| C | 4.919761 | 1.205706 | -0.996979 |
| C | 4.902744 | 1.994337 | -2.158634 |
| H | 4.961152 | 1.510344 | -3.130489 |
| C | 4.807868 | 3.386394 | -2.082722 |
| H | 4.797406 | 3.978410 | -2.994075 |
| C | 4.725947 | 4.012615 | -0.838648 |
| H | 4.650771 | 5.095015 | -0.775686 |
| C | 4.744217 | 3.242625 | 0.327838 |
| H | 4.685602 | 3.725850 | 1.299839 |
| C | 4.841902 | 1.852969 | 0.248538 |
| H | 4.871104 | 1.271155 | 1.165996 |
| C | -0.627756 | -1.084877 | 1.502513 |
| C | 0.195804 | -0.559742 | 2.511041 |
| H | 0.500238 | 0.481438 | 2.478439 |
| C | 0.617377 | -1.369407 | 3.567085 |
| H | 1.252273 | -0.952177 | 4.343957 |
| C | 0.224428 | -2.708189 | 3.627667 |
| H | 0.553224 | -3.334835 | 4.452108 |
| C | -0.598973 | -3.237527 | 2.631544 |
| H | -0.915596 | -4.275703 | 2.677363 |
| C | -1.027819 | -2.429350 | 1.578010 |
| H | -1.680743 | -2.839195 | 0.810570 |
| C | -1.074273 | 1.649868 | 0.565981 |
| C | -0.403203 | 2.621446 | -0.193199 |
| H | 0.139253 | 2.346366 | -1.091364 |
| C | -0.421640 | 3.961785 | 0.196992 |
| H | 0.103926 | 4.700280 | -0.401596 |
| C | -1.110794 | 4.349305 | 1.346012 |
| H | -1.127974 | 5.393343 | 1.645679 |
| C | -1.786445 | 3.390970 | 2.105000 |
| H | -2.333880 | 3.685907 | 2.995652 |
| C | -1.773883 | 2.051859 | 1.718254 |
| H | -2.314386 | 1.320174 | 2.310870 |
| C | -4.243422 | -0.090542 | 0.459967 |
| C | -4.838267 | 1.166260 | 0.274852 |
| C | -5.680154 | 1.675209 | 1.281755 |
| H | -6.129696 | 2.649307 | 1.110692 |
| C | -5.924910 | 0.950988 | 2.440988 |
| C | -5.327346 | -0.303257 | 2.607734 |
| H | -5.517065 | -0.884337 | 3.506963 |
| C | -4.485577 | -0.827660 | 1.620929 |
| H | -4.036735 | -1.804677 | 1.763965 |
| H | -6.580297 | 1.350789 | 3.209683 |
| C | -4.618604 | 1.993692 | -0.940131 |
| O | -5.081343 | 3.108306 | -1.095957 |
| H | -3.987293 | 1.522366 | -1.721732 |
| C | 0.184533 | -0.328383 | -1.237178 |
| H | -0.086945 | 0.349714 | -2.054805 |
| H | 0.039411 | -1.339340 | -1.638632 |
| C | 1.656438 | -0.131458 | -0.800483 |
| H | 2.191189 | 0.366131 | -1.618182 |
| H | 1.719485 | 0.554455 | 0.053195 |
| C | 2.364792 | -1.451438 | -0.460775 |
| H | 2.371532 | -2.089929 | -1.355035 |
| H | 1.783459 | -1.987400 | 0.299920 |
| C | 3.803647 | -1.291724 | 0.058072 |
| H | 3.818126 | -0.685380 | 0.972313 |
| H | 4.186754 | -2.279683 | 0.338845 |

**Int4**

| | | | |
|---|---|---|---|
| P | 4.508372 | -0.423693 | 0.140276 |
| C | 6.225244 | -1.093748 | -0.078819 |
| C | 7.370007 | -0.295670 | -0.223258 |
| H | 7.275073 | 0.783603 | -0.283990 |
| C | 8.640534 | -0.873514 | -0.294245 |
| H | 9.513079 | -0.235236 | -0.409216 |
| C | 8.792224 | -2.258018 | -0.221847 |
| H | 9.780764 | -2.705515 | -0.279572 |
| C | 7.662309 | -3.064623 | -0.064315 |
| H | 7.767310 | -4.144349 | 0.004487 |
| C | 6.396174 | -2.487137 | 0.017108 |
| H | 5.531682 | -3.130984 | 0.166051 |
| C | 4.697302 | 1.367406 | -0.305609 |
| C | 5.112830 | 1.830043 | -1.566558 |
| H | 5.368796 | 1.120154 | -2.348598 |
| C | 5.219477 | 3.196204 | -1.828800 |
| H | 5.544978 | 3.534467 | -2.809281 |
| C | 4.913764 | 4.127710 | -0.832306 |
| H | 5.000665 | 5.191829 | -1.036364 |
| C | 4.502364 | 3.685112 | 0.425340 |
| H | 4.266864 | 4.402749 | 1.207142 |
| C | 4.394458 | 2.315916 | 0.683840 |
| H | 4.063294 | 1.977731 | 1.662324 |
| P | -1.157615 | -0.267570 | 0.051478 |
| C | -0.921906 | -2.071301 | 0.323012 |
| C | -0.918806 | -2.616141 | 1.616597 |
| H | -0.975383 | -1.966239 | 2.483666 |
| C | -0.848433 | -3.997827 | 1.804951 |
| H | -0.850230 | -4.400357 | 2.813964 |
| C | -0.788023 | -4.854693 | 0.706275 |
| H | -0.737443 | -5.929965 | 0.853773 |
| C | -0.810336 | -4.323780 | -0.585336 |
| H | -0.780598 | -4.983914 | -1.447872 |
| C | -0.883535 | -2.944942 | -0.776265 |
| H | -0.925418 | -2.555368 | -1.788365 |
| C | -0.420232 | 0.547657 | 1.534799 |
| C | 0.806964 | 0.139635 | 2.087035 |
| H | 1.352271 | -0.696662 | 1.661786 |
| C | 1.335965 | 0.791101 | 3.202274 |
| H | 2.282379 | 0.455944 | 3.617976 |
| C | 0.646125 | 1.857001 | 3.784652 |
| H | 1.052900 | 2.355788 | 4.660603 |
| C | -0.572919 | 2.269497 | 3.244346 |
| H | -1.123039 | 3.086880 | 3.704744 |
| C | -1.106194 | 1.622318 | 2.126445 |
| H | -2.050875 | 1.951909 | 1.702369 |
| Pd | -3.257107 | 0.552821 | -0.435557 |
| C | -4.031577 | -1.276418 | -0.790601 |
| C | -4.176454 | -1.667638 | -2.131732 |
| C | -4.528560 | -2.138964 | 0.207703 |
| C | -4.788082 | -2.877912 | -2.467676 |
| H | -3.827910 | -1.014054 | -2.928037 |
| C | -5.140405 | -3.359295 | -0.137888 |
| C | -5.269769 | -3.732875 | -1.468214 |
| H | -4.895038 | -3.153402 | -3.515229 |
| H | -5.510266 | -3.988373 | 0.667373 |
| H | -5.746831 | -4.672998 | -1.733208 |
| C | -4.428543 | -1.789226 | 1.641135 |
| O | -4.799774 | -2.495293 | 2.564747 |
| H | -3.974248 | -0.792972 | 1.838038 |
| O | -4.950341 | 1.729907 | -0.868093 |
| C | -4.325087 | 2.847896 | -0.660921 |
| O | -4.827707 | 3.989073 | -0.775760 |
| O | -3.032499 | 2.701185 | -0.302053 |
| K | -2.697331 | 5.210025 | -0.279604 |
| C | -0.059122 | 0.230193 | -1.384159 |
| H | -0.394888 | 1.253828 | -1.589924 |
| H | -0.398062 | -0.365368 | -2.241391 |
| C | 1.476596 | 0.211669 | -1.277776 |
| H | 1.862443 | 0.790849 | -2.129349 |
| H | 1.787229 | 0.762880 | -0.382835 |
| C | 2.130460 | -1.176915 | -1.300211 |
| H | 1.750796 | -1.734405 | -2.168452 |
| H | 1.827584 | -1.754494 | -0.419303 |
| C | 3.666848 | -1.151576 | -1.384748 |
| H | 4.034632 | -2.181260 | -1.461661 |
| H | 3.994940 | -0.635833 | -2.295952 |

**TS2**

| | | | |
|---|---|---|---|
| P | -5.206366 | 0.247755 | 0.163907 |
| C | -7.047584 | 0.438454 | 0.222455 |
| C | -7.811582 | -0.621258 | 0.748781 |

| | | | | | | | |
|---|---|---|---|---|---|---|---|
| H | -7.316837 | -1.549908 | 1.023075 | C | 4.757211 | 1.909642 | -0.422996 |
| C | -9.844395 | 0.680524 | 0.568507 | C | 4.692009 | 2.246670 | -3.186048 |
| H | -10.919596 | 0.773393 | 0.700350 | H | 3.037007 | 0.884263 | -3.168800 |
| C | -9.102288 | 1.744311 | 0.056764 | C | 5.682514 | 2.762536 | -1.057651 |
| H | -9.597809 | 2.673482 | -0.215898 | C | 5.655695 | 2.935034 | -2.434111 |
| C | -7.720127 | 1.623876 | -0.116496 | H | 4.658622 | 2.369860 | -4.267626 |
| H | -7.168868 | 2.466151 | -0.522924 | H | 6.409033 | 3.277898 | -0.434406 |
| C | -4.983836 | -1.234489 | -0.920949 | H | 6.371043 | 3.591469 | -2.924470 |
| C | -5.889674 | -1.633476 | -1.920111 | C | 4.823957 | 1.796013 | 1.051463 |
| H | -6.825159 | -1.095891 | -2.051879 | O | 5.597660 | 2.416934 | 1.766379 |
| C | -5.596687 | -2.721297 | -2.742986 | H | 4.088905 | 1.089208 | 1.490480 |
| H | -6.308374 | -3.023526 | -3.508913 | C | 3.810392 | -1.628633 | -0.301737 |
| C | -4.387156 | -3.410935 | -2.598152 | N | 4.608256 | -1.609643 | -1.751812 |
| H | -4.157398 | -4.246025 | -3.256342 | N | 4.681242 | -1.789238 | -2.854994 |
| C | -3.475957 | -3.020153 | -1.617328 | C | 3.514585 | -3.028470 | -0.033486 |
| H | -2.511300 | -3.499517 | -1.482454 | C | 2.549612 | -3.751859 | -0.766540 |
| C | -3.785351 | -1.950273 | -0.773245 | C | 4.269167 | -3.663007 | 1.064574 |
| H | -3.052263 | -1.690329 | -0.015015 | C | 2.243688 | -5.072305 | -0.466247 |
| P | 0.683742 | 1.577437 | 0.185693 | H | 1.998373 | -3.236056 | -1.550815 |
| C | 0.863231 | 3.425226 | 0.112616 | C | 3.856511 | -5.030163 | 1.368841 |
| C | 0.055357 | 4.287799 | 0.876270 | C | 2.913347 | -5.701352 | 0.616179 |
| H | -0.639311 | 3.876329 | 1.601550 | H | 1.489024 | -5.606432 | -1.035966 |
| C | 0.144782 | 5.672142 | 0.725830 | H | 4.389449 | -5.527108 | 2.177253 |
| H | -0.487366 | 6.320244 | 1.328423 | H | 2.678328 | -6.739431 | 0.855194 |
| C | 1.041410 | 6.222615 | -0.192914 | O | -0.039165 | -1.645684 | -1.586393 |
| H | 1.113996 | 7.301655 | -0.306878 | C | -0.019158 | -1.905497 | -0.355026 |
| C | 1.845088 | 5.378640 | -0.959091 | O | -0.811267 | -2.708820 | 0.265769 |
| H | 2.552160 | 5.793764 | -1.672726 | O | 0.896453 | -1.303079 | 0.448705 |
| C | 1.758049 | 3.992280 | -0.807759 | K | 1.064317 | -3.337231 | 1.938567 |
| H | 2.393609 | 3.349686 | -1.406072 | O | 5.163808 | -3.073926 | 1.717289 |
| C | 0.227037 | 1.279747 | 1.950548 | H | 4.619927 | -1.287609 | 0.347138 |
| C | -0.670077 | 0.262519 | 2.320767 | C | -0.809901 | 1.321279 | -0.899645 |
| H | -1.143286 | -0.357683 | 1.569938 | H | -0.796446 | 0.259296 | -1.178296 |
| C | -0.920848 | -0.001623 | 3.669590 | H | -0.541728 | 1.866221 | -1.816276 |
| H | -1.635866 | -0.778045 | 3.932997 | C | -2.195271 | 1.764492 | -0.411427 |
| C | -0.264820 | 0.719181 | 4.670388 | H | -2.467397 | 1.206578 | 0.490586 |
| H | -0.462727 | 0.508067 | 5.718828 | H | -2.195936 | 2.827024 | -0.131499 |
| C | 0.650221 | 1.710584 | 4.312986 | C | -3.252878 | 1.521079 | -1.504852 |
| H | 1.179708 | 2.271525 | 5.079125 | H | -3.089687 | 0.532276 | -1.948887 |
| C | 0.892603 | 1.987277 | 2.965923 | H | -3.099542 | 2.246482 | -2.316701 |
| H | 1.610486 | 2.760529 | 2.708391 | C | -4.714077 | 1.626241 | -1.029671 |
| Pd | 2.375705 | -0.050635 | -0.376314 | H | -5.385670 | 1.613704 | -1.898039 |
| C | 3.775833 | 1.205068 | -1.160178 | H | -4.867462 | 2.580697 | -0.507298 |
| C | 3.774070 | 1.403058 | -2.558067 | C | -9.190478 | -0.506516 | 0.912503 |

| | | | |
|---|---|---|---|
| H | -9.756485 | -1.344632 | 1.312536 |

**Int5**

| | | | |
|---|---|---|---|
| P | 5.398803 | 0.745956 | 0.460181 |
| C | 7.170488 | 0.202950 | 0.408863 |
| C | 8.150359 | 1.122436 | 0.829589 |
| H | 7.852664 | 2.131758 | 1.104931 |
| C | 9.894804 | -0.532396 | 0.559795 |
| H | 10.942601 | -0.816012 | 0.618530 |
| C | 8.936131 | -1.459563 | 0.153552 |
| H | 9.233465 | -2.472050 | -0.109375 |
| C | 7.588200 | -1.095184 | 0.075688 |
| H | 6.864340 | -1.835571 | -0.249282 |
| C | 5.385487 | 2.081224 | -0.830308 |
| C | 6.207504 | 2.081494 | -1.969717 |
| H | 6.927026 | 1.280058 | -2.114685 |
| C | 6.117120 | 3.104989 | -2.913479 |
| H | 6.762110 | 3.090884 | -3.789057 |
| C | 5.197489 | 4.142949 | -2.738648 |
| H | 5.125573 | 4.938022 | -3.476718 |
| C | 4.373631 | 4.154987 | -1.612706 |
| H | 3.655056 | 4.957628 | -1.468326 |
| C | 4.473176 | 3.135215 | -0.662762 |
| H | 3.834837 | 3.156500 | 0.217050 |
| P | -0.563459 | -1.399317 | 0.388529 |
| C | 0.088657 | -2.736275 | -0.728017 |
| C | 1.163511 | -3.575917 | -0.384675 |
| H | 1.603852 | -3.509302 | 0.605262 |
| C | 1.662550 | -4.509182 | -1.294851 |
| H | 2.492577 | -5.151072 | -1.007622 |
| C | 1.096788 | -4.622160 | -2.567288 |
| H | 1.482845 | -5.352795 | -3.274459 |
| C | 0.027762 | -3.797938 | -2.919934 |
| H | -0.429706 | -3.883735 | -3.902410 |
| C | -0.473665 | -2.865545 | -2.008179 |
| H | -1.313936 | -2.239599 | -2.289946 |
| C | -0.241931 | -2.074879 | 2.070907 |
| C | 0.184774 | -1.241533 | 3.117029 |
| H | 0.367248 | -0.190826 | 2.931290 |
| C | 0.334725 | -1.747864 | 4.408950 |
| H | 0.662859 | -1.085019 | 5.205556 |
| C | 0.046176 | -3.085494 | 4.683328 |
| H | 0.158040 | -3.474225 | 5.692923 |
| C | -0.402502 | -3.917548 | 3.655826 |
| H | -0.646615 | -4.957666 | 3.858759 |
| C | -0.548077 | -3.415186 | 2.362060 |
| H | -0.903792 | -4.074400 | 1.574870 |
| Pd | -2.755242 | -0.306408 | 0.249380 |
| C | -3.567508 | -1.210339 | -1.402805 |
| C | -3.429537 | -0.626964 | -2.680820 |
| C | -4.244590 | -2.453704 | -1.354330 |
| C | -3.906805 | -1.247705 | -3.836377 |
| H | -2.936708 | 0.337327 | -2.777735 |
| C | -4.726892 | -3.080330 | -2.520451 |
| C | -4.558676 | -2.486985 | -3.763227 |
| H | -3.774777 | -0.761209 | -4.802216 |
| H | -5.233332 | -4.036161 | -2.410432 |
| H | -4.932510 | -2.968528 | -4.664289 |
| C | -4.467133 | -3.142106 | -0.063404 |
| O | -4.998292 | -4.237360 | 0.064210 |
| H | -4.107606 | -2.578681 | 0.823431 |
| C | -4.391843 | 0.829179 | 0.361627 |
| C | -4.625240 | 2.086406 | -0.168804 |
| C | -3.669051 | 2.713624 | -1.051387 |
| C | -5.865964 | 2.840731 | 0.207631 |
| C | -3.839133 | 3.984356 | -1.534875 |
| H | -2.771208 | 2.147814 | -1.275383 |
| C | -5.950970 | 4.215463 | -0.312946 |
| C | -5.002779 | 4.735526 | -1.148975 |
| H | -3.105765 | 4.430480 | -2.201866 |
| H | -6.835556 | 4.782132 | -0.031292 |
| H | -5.126162 | 5.746870 | -1.538430 |
| O | -1.267657 | 2.413000 | 0.974444 |
| C | -1.726198 | 1.847611 | 2.043766 |
| O | -1.990398 | 2.509546 | 3.103492 |
| O | -1.966849 | 0.539870 | 2.036408 |
| K | -2.937238 | 4.259501 | 1.554617 |
| O | -6.763420 | 2.378488 | 0.926350 |
| H | -5.198832 | 0.477076 | 1.011252 |
| C | 0.662984 | -0.022749 | 0.107646 |
| H | 0.220818 | 0.851669 | 0.602859 |
| H | 0.559546 | 0.190771 | -0.964963 |
| C | 2.151211 | -0.242556 | 0.467111 |
| H | 2.493503 | 0.599697 | 1.081729 |
| H | 2.282987 | -1.131629 | 1.097161 |
| C | 3.050730 | -0.367601 | -0.772789 |

| | | | |
|---|---|---|---|
| H | 2.960116 | 0.542084 | -1.381141 |
| H | 2.679904 | -1.190614 | -1.397223 |
| C | 4.536263 | -0.640896 | -0.473724 |
| H | 5.072303 | -0.842543 | -1.410639 |
| H | 4.618451 | -1.540316 | 0.151562 |
| C | 9.495103 | 0.764631 | 0.896074 |
| H | 10.232412 | 1.496449 | 1.217483 |

**TS3**

| | | | |
|---|---|---|---|
| P | 5.458579 | -0.384762 | 1.691540 |
| C | 7.087332 | -1.284251 | 1.642769 |
| C | 8.052949 | -1.146931 | 0.634477 |
| H | 7.859964 | -0.496401 | -0.212773 |
| C | 9.263911 | -1.841161 | 0.703193 |
| H | 9.997201 | -1.720218 | -0.090920 |
| C | 9.531941 | -2.689496 | 1.777960 |
| H | 10.473463 | -3.230695 | 1.828393 |
| C | 8.582387 | -2.832317 | 2.792918 |
| H | 8.781877 | -3.484539 | 3.639814 |
| C | 7.380415 | -2.128333 | 2.729433 |
| H | 6.659610 | -2.231362 | 3.538328 |
| C | 5.571840 | 0.708109 | 0.197625 |
| C | 5.363734 | 0.293851 | -1.128369 |
| H | 5.118578 | -0.741336 | -1.344792 |
| C | 5.458848 | 1.200286 | -2.186350 |
| H | 5.280722 | 0.860939 | -3.203488 |
| C | 5.776411 | 2.538104 | -1.939010 |
| H | 5.848473 | 3.243093 | -2.763305 |
| C | 5.992017 | 2.965221 | -0.627356 |
| H | 6.235234 | 4.005242 | -0.424204 |
| C | 5.881513 | 2.058743 | 0.428988 |
| H | 6.026608 | 2.402594 | 1.450285 |
| P | -0.366772 | 0.636338 | -0.452510 |
| C | 0.296132 | 2.342337 | -0.153475 |
| C | 1.569542 | 2.764415 | -0.577738 |
| H | 2.197699 | 2.095849 | -1.158320 |
| C | 2.036749 | 4.043315 | -0.272527 |
| H | 3.027068 | 4.344505 | -0.605275 |
| C | 1.239667 | 4.925494 | 0.461872 |
| H | 1.603793 | 5.922812 | 0.698411 |
| C | -0.026336 | 4.521283 | 0.885877 |
| H | -0.659297 | 5.200953 | 1.450767 |
| C | -0.495021 | 3.240888 | 0.579084 |
| H | -1.484060 | 2.937528 | 0.906309 |
| C | 0.208302 | 0.254103 | -2.167370 |
| C | 0.473702 | -1.076356 | -2.537651 |
| H | 0.422405 | -1.868134 | -1.797183 |
| C | 0.765448 | -1.400065 | -3.864886 |
| H | 0.965335 | -2.437432 | -4.122973 |
| C | 0.786149 | -0.407671 | -4.849604 |
| H | 1.017711 | -0.661698 | -5.882144 |
| C | 0.510558 | 0.915353 | -4.494709 |
| H | 0.522259 | 1.698709 | -5.249660 |
| C | 0.222314 | 1.240990 | -3.167088 |
| H | 0.006906 | 2.274244 | -2.909899 |
| Pd | -2.729932 | -0.001862 | -0.107207 |
| C | -3.535559 | 1.226381 | 1.310745 |
| C | -3.332981 | 0.934057 | 2.681063 |
| C | -4.138574 | 2.481331 | 1.022990 |
| C | -3.695174 | 1.822042 | 3.688877 |
| H | -2.903980 | -0.026155 | 2.955495 |
| C | -4.514435 | 3.373692 | 2.046489 |
| C | -4.292328 | 3.054802 | 3.377095 |
| H | -3.520315 | 1.553645 | 4.729573 |
| H | -4.972529 | 4.315720 | 1.755687 |
| H | -4.581652 | 3.742590 | 4.168446 |
| C | -4.374596 | 2.897103 | -0.374992 |
| O | -4.829179 | 3.979596 | -0.722334 |
| H | -4.090768 | 2.132999 | -1.129866 |
| C | -4.626793 | -0.358965 | 0.374947 |
| C | -5.122754 | -1.454250 | 1.057643 |
| C | -4.260615 | -2.475060 | 1.605868 |
| C | -6.606038 | -1.588347 | 1.213230 |
| C | -4.763280 | -3.541719 | 2.294318 |
| H | -3.193187 | -2.400271 | 1.403366 |
| C | -7.058684 | -2.751504 | 1.979792 |
| C | -6.181919 | -3.663867 | 2.486757 |
| H | -4.101099 | -4.313239 | 2.678113 |
| H | -8.133038 | -2.849221 | 2.117218 |
| H | -6.560892 | -4.519514 | 3.046967 |
| O | -1.176691 | -2.805066 | -0.106775 |
| C | -1.790901 | -2.713238 | -1.206826 |
| O | -1.857487 | -3.639372 | -2.097289 |
| O | -2.401214 | -1.547164 | -1.543795 |
| K | -3.200046 | -2.326564 | -3.705773 |
| C | 4.318915 | -1.796280 | 1.190831 |

| | | | |
|---|---|---|---|
| H | 4.637334 | -2.631155 | 1.828397 |
| H | 4.506076 | -2.118087 | 0.158309 |
| C | 2.818400 | -1.536885 | 1.417635 |
| H | 2.288558 | -2.488912 | 1.279725 |
| H | 2.655168 | -1.242918 | 2.463855 |
| C | 2.184235 | -0.490387 | 0.487067 |
| H | 2.427090 | -0.744775 | -0.553004 |
| H | 2.625555 | 0.496126 | 0.677641 |
| C | 0.662423 | -0.442673 | 0.665887 |
| H | 0.195575 | -1.432004 | 0.540140 |
| H | 0.405718 | -0.106058 | 1.679958 |
| O | -7.421183 | -0.780284 | 0.733668 |
| H | -5.417571 | 0.278756 | -0.024328 |

**Int6**

| | | | |
|---|---|---|---|
| P | -5.381079 | -0.326175 | -0.491967 |
| C | -7.033075 | -1.003379 | -1.012116 |
| C | -8.193378 | -0.915363 | -0.226055 |
| H | -8.133686 | -0.514359 | 0.780735 |
| C | -9.429552 | -1.344690 | -0.715949 |
| H | -10.311125 | -1.269710 | -0.083275 |
| C | -9.535985 | -1.870455 | -2.003583 |
| H | -10.497407 | -2.207778 | -2.382598 |
| C | -8.394116 | -1.950467 | -2.804599 |
| H | -8.462031 | -2.348628 | -3.814235 |
| C | -7.163087 | -1.512981 | -2.317735 |
| H | -6.292927 | -1.562974 | -2.968336 |
| C | -5.571697 | -0.187137 | 1.349529 |
| C | -5.901292 | -1.263230 | 2.191926 |
| H | -6.092513 | -2.244547 | 1.765799 |
| C | -5.995157 | -1.088968 | 3.572701 |
| H | -6.248289 | -1.934026 | 4.208481 |
| C | -5.758112 | 0.167189 | 4.138743 |
| H | -5.824441 | 0.300800 | 5.215667 |
| C | -5.431921 | 1.245845 | 3.316697 |
| H | -5.238163 | 2.224455 | 3.747880 |
| C | -5.342093 | 1.067824 | 1.933343 |
| H | -5.075579 | 1.908666 | 1.298371 |
| P | 0.179825 | 0.602225 | 0.147312 |
| C | -0.544817 | 1.011206 | -1.499958 |
| C | -1.515078 | 1.998299 | -1.723117 |
| H | -1.891027 | 2.590614 | -0.894908 |
| C | -2.009964 | 2.224868 | -3.010492 |
| H | -2.764260 | 2.992159 | -3.168883 |
| C | -1.545924 | 1.463661 | -4.084294 |
| H | -1.933639 | 1.639718 | -5.085333 |
| C | -0.582206 | 0.474521 | -3.866554 |
| H | -0.217547 | -0.124374 | -4.697671 |
| C | -0.074287 | 0.248861 | -2.586772 |
| H | 0.665173 | -0.530645 | -2.405984 |
| C | -0.470014 | 1.879435 | 1.309843 |
| C | -1.143931 | 1.535209 | 2.494605 |
| H | -1.357445 | 0.496100 | 2.719591 |
| C | -1.545055 | 2.517145 | 3.403116 |
| H | -2.067062 | 2.223743 | 4.310918 |
| C | -1.278765 | 3.862950 | 3.147920 |
| H | -1.588817 | 4.627399 | 3.856586 |
| C | -0.592423 | 4.217510 | 1.984434 |
| H | -0.350506 | 5.258201 | 1.785522 |
| C | -0.186269 | 3.237825 | 1.078773 |
| H | 0.387063 | 3.533857 | 0.207689 |
| Pd | 2.568369 | 0.341635 | 0.011701 |
| C | 4.446254 | 1.123879 | -1.094707 |
| C | 4.800765 | 0.748982 | -2.438569 |
| C | 3.468685 | 2.207636 | -0.943296 |
| C | 4.198401 | 1.317245 | -3.527905 |
| H | 5.593251 | 0.022491 | -2.572093 |
| C | 2.851189 | 2.746770 | -2.125006 |
| C | 3.180112 | 2.304577 | -3.375374 |
| H | 4.511878 | 1.023686 | -4.527382 |
| H | 2.145402 | 3.558709 | -1.982224 |
| H | 2.702192 | 2.729940 | -4.253822 |
| C | 3.408155 | 3.057367 | 0.267987 |
| O | 2.698005 | 4.053277 | 0.377142 |
| H | 4.086888 | 2.779146 | 1.094169 |
| C | 5.733799 | -0.750429 | 0.167251 |
| C | 5.771525 | -1.803971 | -0.770468 |
| C | 6.509304 | -0.856633 | 1.421338 |
| C | 6.577004 | -2.929181 | -0.598947 |
| H | 5.095727 | -1.775887 | -1.620675 |
| C | 7.354814 | -2.032550 | 1.527145 |
| C | 7.388105 | -3.017741 | 0.554129 |
| H | 6.564666 | -3.729176 | -1.333892 |
| H | 7.964198 | -2.109200 | 2.425864 |
| H | 8.036434 | -3.884425 | 0.688436 |
| O | 1.574691 | -2.172738 | -1.198093 |

| | | | |
|---|---|---|---|
| C | 2.113957 | -2.538196 | -0.113636 |
| O | 2.397699 | -3.729351 | 0.232883 |
| O | 2.437482 | -1.586225 | 0.816836 |
| K | 4.331865 | -3.007988 | 1.750348 |
| C | -0.676340 | -0.975616 | 0.628466 |
| H | -0.274833 | -1.229384 | 1.615284 |
| H | -0.231480 | -1.705222 | -0.058637 |
| C | -2.220701 | -1.039260 | 0.604079 |
| H | -2.664508 | -0.057901 | 0.812133 |
| H | -2.549393 | -1.690821 | 1.427354 |
| C | -2.795019 | -1.594631 | -0.708819 |
| H | -2.286277 | -2.540113 | -0.941025 |
| H | -2.562975 | -0.912788 | -1.533823 |
| C | -4.309498 | -1.868957 | -0.679548 |
| H | -4.555887 | -2.599074 | 0.102545 |
| H | -4.606326 | -2.322358 | -1.632888 |
| C | 4.901239 | 0.431909 | 0.068368 |
| H | 4.907285 | 0.974477 | 1.012470 |
| O | 6.417757 | -0.011995 | 2.353708 |

**TS4**

| | | | |
|---|---|---|---|
| Pd | -2.888546 | 0.646019 | 0.778414 |
| P | -0.554696 | -0.090312 | 0.581075 |
| P | 5.115418 | -0.315688 | -0.126640 |
| C | 6.783876 | -0.891477 | -0.710982 |
| C | 7.931449 | -0.082038 | -0.677683 |
| H | 7.846495 | 0.961674 | -0.392504 |
| C | 9.186205 | -0.596030 | -1.013885 |
| H | 10.056585 | 0.055556 | -0.984491 |
| C | 9.325751 | -1.932526 | -1.388552 |
| H | 10.301883 | -2.330978 | -1.653350 |
| C | 8.197411 | -2.755762 | -1.411082 |
| H | 8.290464 | -3.802302 | -1.691486 |
| C | 6.946913 | -2.243332 | -1.067412 |
| H | 6.088683 | -2.911303 | -1.070048 |
| C | 5.252479 | 1.528759 | -0.273803 |
| C | 5.635548 | 2.194849 | -1.451194 |
| H | 5.904668 | 1.621227 | -2.334513 |
| C | 5.685130 | 3.587844 | -1.500677 |
| H | 5.981052 | 4.086217 | -2.420726 |
| C | 5.347103 | 4.341690 | -0.372887 |
| H | 5.377204 | 5.427619 | -0.414226 |
| C | 4.963296 | 3.695299 | 0.801907 |
| H | 4.687805 | 4.273745 | 1.679744 |
| C | 4.919341 | 2.299213 | 0.850608 |
| H | 4.598778 | 1.803160 | 1.762774 |
| C | -0.328368 | -1.926674 | 0.618872 |
| C | -0.343870 | -2.602080 | 1.852539 |
| H | -0.470511 | -2.043162 | 2.772414 |
| C | -0.264317 | -3.996448 | 1.899741 |
| H | -0.295508 | -4.495972 | 2.864074 |
| C | -0.177254 | -4.740641 | 0.720426 |
| H | -0.105235 | -5.825855 | 0.759883 |
| C | -0.199929 | -4.077682 | -0.513266 |
| H | -0.154180 | -4.645208 | -1.440662 |
| C | -0.287254 | -2.682688 | -0.569643 |
| H | -0.398339 | -2.192269 | -1.534958 |
| C | 0.431217 | 0.509544 | 2.023484 |
| C | 1.661726 | -0.062659 | 2.387355 |
| H | 2.044169 | -0.924033 | 1.848563 |
| C | 2.399286 | 0.450026 | 3.456572 |
| H | 3.343897 | -0.015377 | 3.728659 |
| C | 1.918344 | 1.547723 | 4.174593 |
| H | 2.488804 | 1.944836 | 5.011519 |
| C | 0.696309 | 2.121687 | 3.821235 |
| H | 0.305344 | 2.966635 | 4.382280 |
| C | -0.048552 | 1.604986 | 2.758475 |
| H | -1.008897 | 2.047434 | 2.513845 |
| C | -3.876181 | 2.230162 | -1.535404 |
| C | -4.513242 | 2.604321 | -2.727191 |
| C | -3.695614 | 3.207485 | -0.542687 |
| C | -4.935056 | 3.921944 | -2.919814 |
| H | -4.664265 | 1.858154 | -3.502423 |
| C | -4.084777 | 4.535231 | -0.746384 |
| C | -4.712813 | 4.894787 | -1.937758 |
| H | -5.436380 | 4.194067 | -3.846664 |
| H | -3.916484 | 5.255978 | 0.049234 |
| H | -5.043853 | 5.918603 | -2.097331 |
| C | -3.131554 | 2.736249 | 0.762812 |
| O | -2.733423 | 3.494539 | 1.630607 |
| H | -4.157005 | 1.614664 | 1.161330 |
| C | -3.842564 | -0.351495 | -1.836845 |
| C | -5.197260 | -0.668430 | -1.655079 |
| C | -2.950285 | -1.304929 | -2.501887 |
| C | -5.746505 | -1.881260 | -2.075947 |
| H | -5.828577 | 0.052576 | -1.139061 |

| | | | |
|---|---|---|---|
| C | -3.555387 | -2.552638 | -2.900273 |
| C | -4.907243 | -2.821309 | -2.702258 |
| H | -6.801449 | -2.093629 | -1.920908 |
| H | -2.915361 | -3.263701 | -3.421333 |
| H | -5.323840 | -3.766641 | -3.055037 |
| O | -2.622847 | -0.424385 | 2.805578 |
| C | -3.261226 | -1.481807 | 2.367924 |
| O | -3.275664 | -2.599189 | 2.951345 |
| O | -3.838425 | -1.328323 | 1.190369 |
| K | -3.433112 | -3.525666 | 0.232715 |
| C | 0.372988 | 0.570967 | -0.904654 |
| H | 0.049178 | 1.620176 | -0.920345 |
| H | -0.105341 | 0.097830 | -1.771650 |
| C | 1.909187 | 0.543524 | -0.993322 |
| H | 2.340357 | 0.946217 | -0.069486 |
| H | 2.196280 | 1.248995 | -1.787776 |
| C | 2.537124 | -0.818551 | -1.317347 |
| H | 2.033859 | -1.239531 | -2.198531 |
| H | 2.355387 | -1.525534 | -0.499749 |
| C | 4.047171 | -0.766829 | -1.616262 |
| H | 4.253141 | -0.084622 | -2.451044 |
| H | 4.379861 | -1.761633 | -1.935231 |
| C | -3.237632 | 0.887932 | -1.331152 |
| H | -2.214309 | 0.941475 | -1.709505 |
| O | -1.716982 | -1.057133 | -2.718363 |

**Int7**

| | | | |
|---|---|---|---|
| P | -5.531729 | -0.455510 | 0.406707 |
| C | -7.274499 | -0.776026 | 0.967129 |
| C | -8.350643 | 0.062451 | 0.631457 |
| H | -8.163553 | 0.995663 | 0.109747 |
| C | -9.662859 | -0.279893 | 0.966448 |
| H | -10.475733 | 0.391473 | 0.699148 |
| C | -9.932759 | -1.470227 | 1.642234 |
| H | -10.953792 | -1.734292 | 1.905876 |
| C | -8.876189 | -2.323565 | 1.968812 |
| H | -9.070485 | -3.259757 | 2.486896 |
| C | -7.567537 | -1.984175 | 1.626784 |
| H | -6.765847 | -2.676972 | 1.871329 |
| C | -5.526257 | 1.377088 | 0.127880 |
| C | -5.947946 | 2.315631 | 1.086160 |
| H | -6.341308 | 1.974039 | 2.040281 |
| C | -5.875709 | 3.683632 | 0.825479 |
| H | -6.203295 | 4.395971 | 1.578912 |
| C | -5.371851 | 4.138667 | -0.396880 |
| H | -5.303580 | 5.205503 | -0.594226 |
| C | -4.946480 | 3.220124 | -1.355980 |
| H | -4.533371 | 3.561787 | -2.300597 |
| C | -5.028823 | 1.849600 | -1.095641 |
| H | -4.681158 | 1.141502 | -1.843153 |
| P | -0.002562 | -0.575204 | -0.479182 |
| C | -0.792399 | -2.224999 | -0.793666 |
| C | -2.122944 | -2.401403 | -1.214795 |
| H | -2.747912 | -1.538720 | -1.424190 |
| C | -2.662506 | -3.679541 | -1.369046 |
| H | -3.695169 | -3.790070 | -1.691659 |
| C | -1.882129 | -4.807814 | -1.103726 |
| H | -2.302271 | -5.803798 | -1.224970 |
| C | -0.559753 | -4.648103 | -0.688945 |
| H | 0.059915 | -5.519142 | -0.489764 |
| C | -0.018420 | -3.368733 | -0.540685 |
| H | 1.017093 | -3.252495 | -0.241446 |
| C | -0.728641 | 0.532929 | -1.760186 |
| C | -0.797534 | 1.917135 | -1.518787 |
| H | -0.464233 | 2.323505 | -0.569995 |
| C | -1.238954 | 2.791032 | -2.512877 |
| H | -1.268141 | 3.857147 | -2.303630 |
| C | -1.614953 | 2.306683 | -3.767525 |
| H | -1.953630 | 2.990852 | -4.542651 |
| C | -1.526512 | 0.937104 | -4.027409 |
| H | -1.797557 | 0.546959 | -5.006070 |
| C | -1.080442 | 0.060367 | -3.037319 |
| H | -1.003787 | -0.998611 | -3.262932 |
| Pd | 2.623345 | -0.302226 | -0.090697 |
| C | 5.337588 | -1.457539 | 0.135430 |
| C | 6.589817 | -1.870077 | 0.616312 |
| C | 4.681341 | -2.282767 | -0.789411 |
| C | 7.160985 | -3.060428 | 0.162852 |
| H | 7.107455 | -1.247282 | 1.339520 |
| C | 5.262710 | -3.461621 | -1.270298 |
| C | 6.507272 | -3.858057 | -0.786194 |
| H | 8.129852 | -3.369696 | 0.550598 |
| H | 4.713413 | -4.059835 | -1.992326 |
| H | 6.961105 | -4.785967 | -1.127234 |
| C | 3.314390 | -1.843625 | -1.185680 |
| O | 2.662903 | -2.375562 | -2.061091 |

| | | | |
|---|---|---|---|
| H | 2.513650 | -1.445346 | 0.988652 |
| C | 5.018378 | 0.509743 | 1.745570 |
| C | 4.838345 | -0.159745 | 2.970148 |
| C | 5.571102 | 1.856291 | 1.728340 |
| C | 5.175747 | 0.416746 | 4.188380 |
| H | 4.404089 | -1.158802 | 2.947678 |
| C | 5.906636 | 2.411453 | 3.009092 |
| C | 5.712784 | 1.718585 | 4.190925 |
| H | 5.013725 | -0.119833 | 5.120364 |
| H | 6.324134 | 3.416925 | 3.012651 |
| H | 5.977784 | 2.189710 | 5.138085 |
| O | 1.725270 | 1.655333 | 0.677680 |
| C | 2.172127 | 2.286233 | -0.366580 |
| O | 2.088466 | 3.529948 | -0.542851 |
| O | 2.786171 | 1.517466 | -1.272388 |
| K | 4.561400 | 3.466113 | -1.265063 |
| C | -0.773241 | 0.000565 | 1.127394 |
| H | -0.195399 | 0.904713 | 1.343965 |
| H | -0.450398 | -0.738222 | 1.872860 |
| C | -2.292699 | 0.256609 | 1.227237 |
| H | -2.700470 | 0.498693 | 0.238866 |
| H | -2.454358 | 1.158423 | 1.835181 |
| C | -3.087169 | -0.896074 | 1.859423 |
| H | -2.663903 | -1.108251 | 2.852279 |
| H | -2.956175 | -1.809010 | 1.267864 |
| C | -4.591169 | -0.618037 | 2.037474 |
| H | -4.749552 | 0.277180 | 2.652821 |
| H | -5.049151 | -1.456054 | 2.576162 |
| C | 4.724152 | -0.126041 | 0.456358 |
| H | 4.971607 | 0.577293 | -0.342814 |
| O | 5.783459 | 2.506652 | 0.639794 |

**TS5**

| | | | |
|---|---|---|---|
| P | 5.489200 | 0.508797 | 0.435346 |
| C | 7.217330 | 0.879786 | 1.009955 |
| C | 8.318947 | 0.069415 | 0.688261 |
| H | 8.162335 | -0.870367 | 0.168414 |
| C | 9.618007 | 0.448220 | 1.035045 |
| H | 10.451313 | -0.202021 | 0.778747 |
| C | 9.848862 | 1.647951 | 1.708652 |
| H | 10.859666 | 1.940331 | 1.981621 |
| C | 8.766569 | 2.473957 | 2.020858 |
| H | 8.930445 | 3.417057 | 2.536904 |
| C | 7.471049 | 2.098167 | 1.667085 |
| H | 6.648667 | 2.770187 | 1.900566 |
| C | 5.536594 | -1.324920 | 0.167824 |
| C | 5.977030 | -2.244838 | 1.135750 |
| H | 6.352367 | -1.886123 | 2.090873 |
| C | 5.947103 | -3.615888 | 0.883172 |
| H | 6.288600 | -4.313759 | 1.643915 |
| C | 5.468135 | -4.092839 | -0.340863 |
| H | 5.433195 | -5.162353 | -0.532243 |
| C | 5.024694 | -3.192980 | -1.309436 |
| H | 4.630802 | -3.552353 | -2.255653 |
| C | 5.063962 | -1.819033 | -1.056966 |
| H | 4.702140 | -1.125987 | -1.811854 |
| P | -0.023706 | 0.462205 | -0.495541 |
| C | 0.689399 | 2.144577 | -0.803370 |
| C | 2.011707 | 2.373781 | -1.223588 |
| H | 2.668550 | 1.536384 | -1.437496 |
| C | 2.501230 | 3.673028 | -1.368177 |
| H | 3.528413 | 3.826048 | -1.690625 |
| C | 1.678255 | 4.768174 | -1.093475 |
| H | 2.059504 | 5.780366 | -1.207949 |
| C | 0.364049 | 4.555203 | -0.676175 |
| H | -0.287349 | 5.400215 | -0.466902 |
| C | -0.126745 | 3.254870 | -0.537916 |
| H | -1.153998 | 3.093945 | -0.227519 |
| C | 0.752208 | -0.624273 | -1.762394 |
| C | 0.852779 | -2.004559 | -1.509348 |
| H | 0.517111 | -2.410775 | -0.560954 |
| C | 1.331951 | -2.872418 | -2.490640 |
| H | 1.388446 | -3.936087 | -2.274608 |
| C | 1.711329 | -2.385424 | -3.743547 |
| H | 2.077995 | -3.065408 | -4.509439 |
| C | 1.589008 | -1.020998 | -4.014834 |
| H | 1.860031 | -0.631006 | -4.993430 |
| C | 1.106392 | -0.149439 | -3.037116 |
| H | 0.998077 | 0.904531 | -3.272166 |
| Pd | -2.492662 | 0.108841 | -0.256073 |
| C | -5.170168 | 1.569201 | 0.014029 |
| C | -6.372915 | 2.157221 | 0.434790 |
| C | -4.477638 | 2.162299 | -1.056158 |
| C | -6.870814 | 3.287704 | -0.213742 |
| H | -6.912295 | 1.712213 | 1.265028 |
| C | -4.991633 | 3.281760 | -1.723719 |

| | | | |
|---|---|---|---|
| C | -6.188321 | 3.852898 | -1.299178 |
| H | -7.804298 | 3.731306 | 0.127344 |
| H | -4.423696 | 3.695098 | -2.552643 |
| H | -6.583556 | 4.736237 | -1.795869 |
| C | -3.156822 | 1.574812 | -1.443029 |
| O | -2.538560 | 1.944417 | -2.423030 |
| H | -3.061986 | 0.976510 | 0.952025 |
| C | -5.081889 | -0.195457 | 1.899248 |
| C | -4.957011 | 0.564859 | 3.072373 |
| C | -5.715085 | -1.502253 | 1.940191 |
| C | -5.440101 | 0.121368 | 4.299852 |
| H | -4.455547 | 1.531514 | 3.010286 |
| C | -6.196581 | -1.922077 | 3.224536 |
| C | -6.062490 | -1.138701 | 4.358795 |
| H | -5.323213 | 0.728043 | 5.194748 |
| H | -6.678016 | -2.897358 | 3.273928 |
| H | -6.441077 | -1.507983 | 5.312463 |
| O | -1.709521 | -1.769788 | 0.654156 |
| C | -2.161069 | -2.537581 | -0.310239 |
| O | -2.141615 | -3.796215 | -0.248770 |
| O | -2.705384 | -1.905424 | -1.335722 |
| K | -4.677232 | -3.578506 | -0.805749 |
| C | 0.738998 | -0.078227 | 1.124589 |
| H | 0.182622 | -0.996407 | 1.341149 |
| H | 0.389074 | 0.658029 | 1.860122 |
| C | 2.263900 | -0.291942 | 1.239997 |
| H | 2.686633 | -0.535302 | 0.258303 |
| H | 2.443666 | -1.181553 | 1.860566 |
| C | 3.021146 | 0.889714 | 1.864570 |
| H | 2.583799 | 1.101583 | 2.851336 |
| H | 2.870353 | 1.792057 | 1.261382 |
| C | 4.530646 | 0.655220 | 2.057275 |
| H | 4.708687 | -0.230976 | 2.680206 |
| H | 4.959991 | 1.509454 | 2.593944 |
| C | -4.638252 | 0.289780 | 0.579457 |
| H | -4.827579 | -0.516237 | -0.136961 |
| O | -5.860530 | -2.232976 | 0.890817 |

**Int8**

| | | | |
|---|---|---|---|
| P | 5.167396 | 0.211383 | 0.051478 |
| C | 6.943166 | 0.150703 | 0.598594 |
| C | 7.859337 | -0.808539 | 0.137205 |
| H | 7.516547 | -1.618675 | -0.498426 |
| C | 9.209471 | -0.743315 | 0.490506 |
| H | 9.895985 | -1.502724 | 0.123176 |
| C | 9.676676 | 0.282996 | 1.311725 |
| H | 10.726630 | 0.330899 | 1.589315 |
| C | 8.781359 | 1.253908 | 1.767231 |
| H | 9.131751 | 2.065477 | 2.400612 |
| C | 7.435818 | 1.192510 | 1.406566 |
| H | 6.762829 | 1.972881 | 1.754648 |
| C | 4.871771 | -1.512641 | -0.560941 |
| C | 5.078032 | -2.669814 | 0.210179 |
| H | 5.470848 | -2.585174 | 1.220156 |
| C | 4.788722 | -3.932677 | -0.305772 |
| H | 4.947550 | -4.816480 | 0.307166 |
| C | 4.282408 | -4.061800 | -1.602409 |
| H | 4.043659 | -5.045686 | -1.997880 |
| C | 4.073249 | -2.923634 | -2.380468 |
| H | 3.663895 | -3.011381 | -3.382928 |
| C | 4.369860 | -1.660232 | -1.862688 |
| H | 4.190156 | -0.776166 | -2.468911 |
| P | -0.392060 | 1.038092 | -0.377094 |
| C | 0.561995 | 2.619848 | -0.449394 |
| C | 1.800852 | 2.754051 | -1.097663 |
| H | 2.229283 | 1.912511 | -1.632771 |
| C | 2.492932 | 3.966201 | -1.064406 |
| H | 3.450666 | 4.051112 | -1.571912 |
| C | 1.959650 | 5.060004 | -0.378584 |
| H | 2.498436 | 6.004452 | -0.355227 |
| C | 0.731533 | 4.935874 | 0.273457 |
| H | 0.307269 | 5.782098 | 0.807885 |
| C | 0.034137 | 3.726696 | 0.235959 |
| H | -0.923876 | 3.630621 | 0.741097 |
| C | 0.015961 | 0.147285 | -1.934730 |
| C | 0.074705 | -1.258669 | -1.946003 |
| H | -0.104745 | -1.834229 | -1.039484 |
| C | 0.298032 | -1.937394 | -3.146796 |
| H | 0.337001 | -3.023830 | -3.139491 |
| C | 0.454274 | -1.235955 | -4.343527 |
| H | 0.623605 | -1.771142 | -5.275318 |
| C | 0.367634 | 0.157965 | -4.341363 |
| H | 0.462884 | 0.714335 | -5.270722 |
| C | 0.143636 | 0.844926 | -3.148106 |
| H | 0.056664 | 1.926342 | -3.163662 |
| Pd | -2.604734 | 1.442874 | 0.206885 |

| | | | |
|---|---|---|---|
| C | -4.455274 | -1.138248 | -1.345117 |
| C | -5.564440 | -1.945108 | -1.654163 |
| C | -4.465271 | 0.198251 | -1.805554 |
| C | -6.671056 | -1.452069 | -2.341318 |
| H | -5.546104 | -2.987532 | -1.344703 |
| C | -5.575968 | 0.680730 | -2.521233 |
| C | -6.682140 | -0.123575 | -2.772396 |
| H | -7.516804 | -2.104956 | -2.546899 |
| H | -5.545470 | 1.705365 | -2.878460 |
| H | -7.538276 | 0.275887 | -3.311545 |
| C | -3.334588 | 1.206363 | -1.631422 |
| O | -3.050038 | 1.938445 | -2.564149 |
| H | -2.449780 | -1.046047 | -0.595523 |
| C | -3.441733 | -2.378606 | 0.729865 |
| C | -4.652559 | -2.475154 | 1.410215 |
| C | -2.216005 | -2.908007 | 1.320941 |
| C | -4.761599 | -3.100079 | 2.670399 |
| H | -5.545760 | -2.049658 | 0.958573 |
| C | -2.368634 | -3.535007 | 2.609914 |
| C | -3.606344 | -3.648286 | 3.246293 |
| H | -5.728174 | -3.180899 | 3.161438 |
| H | -1.472462 | -3.965268 | 3.054428 |
| H | -3.671263 | -4.165120 | 4.204952 |
| O | -4.387509 | 1.940417 | 1.239342 |
| C | -3.806021 | 1.866997 | 2.416995 |
| O | -4.425482 | 1.784708 | 3.498106 |
| O | -2.476289 | 1.742154 | 2.364446 |
| K | -3.129942 | -0.592176 | 3.423077 |
| C | 0.392043 | 0.034948 | 0.982601 |
| H | -0.199585 | -0.892199 | 1.008901 |
| H | 0.154682 | 0.589330 | 1.900087 |
| C | 1.893751 | -0.316740 | 0.911686 |
| H | 2.211719 | -0.407142 | -0.133668 |
| H | 2.019111 | -1.321688 | 1.338092 |
| C | 2.816740 | 0.653636 | 1.663635 |
| H | 2.454012 | 0.750890 | 2.697688 |
| H | 2.754681 | 1.654093 | 1.221257 |
| C | 4.291616 | 0.213499 | 1.724297 |
| H | 4.380289 | -0.771687 | 2.200049 |
| H | 4.849044 | 0.917732 | 2.353503 |
| C | -3.263391 | -1.771014 | -0.653888 |
| H | -2.864875 | -2.564914 | -1.305973 |
| O | -1.083363 | -2.801897 | 0.739454 |

**TS6**

| | | | |
|---|---|---|---|
| P | -5.608827 | -0.072864 | -0.405973 |
| C | -5.296857 | 1.663021 | 0.169992 |
| C | -4.548712 | 1.830541 | 1.346359 |
| H | -4.207485 | 0.958642 | 1.899130 |
| C | -4.224572 | 3.107860 | 1.811801 |
| H | -3.631516 | 3.214590 | 2.715993 |
| C | -4.657283 | 4.235804 | 1.114172 |
| H | -4.406335 | 5.230113 | 1.475171 |
| C | -5.411091 | 4.083421 | -0.053387 |
| H | -5.749036 | 4.959813 | -0.601724 |
| C | -5.724466 | 2.808103 | -0.523464 |
| H | -6.309871 | 2.700319 | -1.432802 |
| C | -7.303050 | 0.073904 | -1.143627 |
| C | -8.351994 | 0.564697 | -0.342803 |
| H | -8.134348 | 0.931697 | 0.657299 |
| C | -9.663415 | 0.600083 | -0.812607 |
| H | -10.452534 | 0.993330 | -0.175888 |
| C | -9.965603 | 0.130077 | -2.094044 |
| H | -10.988917 | 0.153396 | -2.460319 |
| C | -8.941077 | -0.371817 | -2.895875 |
| H | -9.161559 | -0.741474 | -3.894702 |
| C | -7.624466 | -0.398763 | -2.426654 |
| H | -6.846361 | -0.791875 | -3.073769 |
| P | 0.176214 | -1.350017 | 0.742581 |
| C | -0.735354 | -0.991987 | 2.326149 |
| C | -2.000046 | -1.515400 | 2.651553 |
| H | -2.475333 | -2.236271 | 1.992515 |
| C | -2.652530 | -1.135985 | 3.827345 |
| H | -3.627622 | -1.557304 | 4.063704 |
| C | -2.049401 | -0.222547 | 4.696979 |
| H | -2.555718 | 0.072850 | 5.613995 |
| C | -0.790902 | 0.296439 | 4.388141 |
| H | -0.305977 | 0.997988 | 5.062606 |
| C | -0.129688 | -0.084929 | 3.215792 |
| H | 0.861623 | 0.321137 | 2.997447 |
| C | -0.198749 | -3.131168 | 0.389964 |
| C | -0.145537 | -3.591699 | -0.939898 |
| H | 0.070547 | -2.891979 | -1.741295 |
| C | -0.313766 | -4.944835 | -1.235324 |
| H | -0.269268 | -5.275548 | -2.270983 |
| C | -0.527782 | -5.873281 | -0.213249 |

| | | | |
|---|---|---|---|
| H | -0.658874 | -6.927772 | -0.445878 |
| C | -0.558241 | -5.433390 | 1.111315 |
| H | -0.711250 | -6.145434 | 1.919819 |
| C | -0.393104 | -4.079133 | 1.409246 |
| H | -0.414535 | -3.756864 | 2.446170 |
| Pd | 2.297463 | -0.645919 | 0.869380 |
| C | 4.944717 | 0.029298 | -2.629420 |
| C | 6.289844 | -0.361864 | -2.728703 |
| C | 4.070384 | -0.723183 | -1.811871 |
| C | 6.767305 | -1.489140 | -2.056265 |
| H | 6.963067 | 0.221688 | -3.355566 |
| C | 4.552195 | -1.867572 | -1.163643 |
| C | 5.887806 | -2.247208 | -1.274673 |
| H | 7.812209 | -1.777589 | -2.150390 |
| H | 3.860973 | -2.417264 | -0.530440 |
| H | 6.243433 | -3.127533 | -0.745539 |
| C | 2.587099 | -0.418194 | -1.742975 |
| O | 1.777602 | -1.189251 | -2.254609 |
| H | 3.588431 | 1.110306 | -3.925140 |
| C | 4.129719 | 2.299902 | -2.212237 |
| C | 4.912102 | 3.418959 | -1.906826 |
| C | 3.049230 | 1.978764 | -1.374382 |
| C | 4.639595 | 4.184740 | -0.765288 |
| H | 5.738943 | 3.686923 | -2.564055 |
| C | 2.782572 | 2.719476 | -0.223025 |
| C | 3.593693 | 3.814968 | 0.086115 |
| H | 5.257593 | 5.048319 | -0.530351 |
| H | 2.014395 | 2.384961 | 0.465359 |
| H | 3.423799 | 4.350399 | 1.014051 |
| O | 4.173182 | 0.194693 | 1.361521 |
| C | 4.005336 | 1.186298 | 2.279601 |
| O | 5.044716 | 1.907236 | 2.510888 |
| O | 2.863696 | 1.364002 | 2.793036 |
| K | 6.124162 | 1.271209 | 0.328292 |
| C | -0.867882 | -0.435189 | -0.522093 |
| H | -0.312111 | -0.526598 | -1.462119 |
| H | -0.769994 | 0.615799 | -0.220432 |
| C | -2.347035 | -0.793957 | -0.704081 |
| H | -2.863667 | -0.743860 | 0.261194 |
| H | -2.446504 | -1.830145 | -1.054778 |
| C | -3.040207 | 0.154540 | -1.695223 |
| H | -2.517254 | 0.110135 | -2.660998 |
| H | -2.932410 | 1.187315 | -1.340268 |
| C | -4.530534 | -0.136730 | -1.951870 |
| H | -4.639550 | -1.149036 | -2.364208 |
| H | -4.920439 | 0.565766 | -2.700430 |
| C | 4.470723 | 1.308115 | -3.303435 |
| H | 5.256807 | 1.704030 | -3.956346 |
| O | 2.207003 | 0.952487 | -1.744544 |

**P**

| | | | |
|---|---|---|---|
| C | -2.385584 | -1.052342 | -1.364139 |
| C | -1.043345 | -1.161533 | -1.912897 |
| C | -3.132628 | 0.121508 | -1.800530 |
| C | -0.545179 | -0.281304 | -2.856843 |
| H | -0.478812 | -2.058703 | -1.669166 |
| C | -2.596222 | 0.969843 | -2.799572 |
| C | -1.334337 | 0.800913 | -3.333775 |
| H | 0.440526 | -0.465758 | -3.282279 |
| H | -3.222846 | 1.800279 | -3.113392 |
| H | -0.949997 | 1.473940 | -4.095812 |
| C | -4.391166 | 0.564924 | -1.228663 |
| O | -5.113042 | 1.455452 | -1.645381 |
| C | -4.186805 | -2.361029 | -0.140222 |
| C | -4.651249 | -3.676468 | 0.128111 |
| C | -5.136880 | -1.325903 | 0.047300 |
| C | -5.965519 | -3.939725 | 0.497081 |
| H | -3.944156 | -4.497848 | 0.022564 |
| C | -6.462561 | -1.585850 | 0.381839 |
| C | -6.893331 | -2.896393 | 0.612084 |
| H | -6.273689 | -4.966611 | 0.688171 |
| H | -7.131670 | -0.738081 | 0.502009 |
| H | -7.924107 | -3.094465 | 0.896269 |
| C | -2.848934 | -2.117692 | -0.589225 |
| H | -2.159236 | -2.953285 | -0.473988 |
| O | -4.722668 | 0.002445 | 0.034620 |
| Pd | 0.720857 | 0.034126 | -0.391499 |
| P | 2.319498 | -1.421959 | 0.438962 |
| P | 0.505993 | 2.064117 | 0.726915 |
| C | 2.006433 | -3.252167 | 0.518632 |
| C | 1.149955 | -3.789367 | 1.496264 |
| H | 0.732643 | -3.146815 | 2.266320 |
| C | 0.806259 | -5.142121 | 1.488447 |
| H | 0.139823 | -5.529751 | 2.255273 |
| C | 1.305610 | -5.991194 | 0.499239 |
| H | 1.034177 | -7.043845 | 0.490429 |

| | | | |
|---|---|---|---|
| C | 2.146073 | -5.469927 | -0.487164 |
| H | 2.536858 | -6.117759 | -1.268769 |
| C | 2.487838 | -4.117475 | -0.480049 |
| H | 3.140613 | -3.730641 | -1.257728 |
| C | 4.074909 | -1.385108 | -0.181632 |
| C | 4.386856 | -0.514723 | -1.235917 |
| H | 3.591296 | 0.090231 | -1.665600 |
| C | 5.692460 | -0.421237 | -1.726954 |
| H | 5.913601 | 0.258708 | -2.546150 |
| C | 6.706777 | -1.199455 | -1.167992 |
| H | 7.723090 | -1.129407 | -1.548763 |
| C | 6.410660 | -2.072827 | -0.117109 |
| H | 7.196593 | -2.684014 | 0.321150 |
| C | 5.106391 | -2.163935 | 0.370327 |
| H | 4.885303 | -2.856228 | 1.178824 |
| C | -1.068374 | 2.712501 | 1.468582 |
| C | -1.144580 | 3.955150 | 2.120811 |
| H | -0.265151 | 4.592248 | 2.182601 |
| C | -2.347986 | 4.394192 | 2.671477 |
| H | -2.394490 | 5.360561 | 3.169847 |
| C | -3.495360 | 3.600403 | 2.569334 |
| H | -4.437003 | 3.950558 | 2.986471 |
| C | -3.434650 | 2.368669 | 1.917896 |
| H | -4.318842 | 1.750781 | 1.796311 |
| C | -2.224617 | 1.926207 | 1.373232 |
| H | -2.190421 | 0.972473 | 0.854694 |
| C | 1.119560 | 3.561209 | -0.186605 |
| C | 2.100111 | 4.446985 | 0.285133 |
| H | 2.553731 | 4.293518 | 1.259743 |
| C | 2.513211 | 5.538208 | -0.485987 |
| H | 3.278552 | 6.209723 | -0.102242 |
| C | 1.945754 | 5.766981 | -1.739150 |
| H | 2.265262 | 6.616521 | -2.338402 |
| C | 0.968950 | 4.890667 | -2.222360 |
| H | 0.523563 | 5.056077 | -3.200551 |
| C | 0.569102 | 3.793753 | -1.460798 |
| H | -0.170937 | 3.100455 | -1.855994 |
| C | 1.593417 | 2.033473 | 2.260802 |
| H | 1.070613 | 1.359841 | 2.950292 |
| H | 1.604323 | 3.013095 | 2.755685 |
| C | 3.031731 | 1.531724 | 1.979683 |
| H | 3.751079 | 2.279619 | 2.340703 |
| H | 3.196382 | 1.455630 | 0.898443 |
| C | 3.415621 | 0.187778 | 2.629603 |
| H | 3.381166 | 0.301725 | 3.723942 |
| H | 4.465168 | -0.014947 | 2.379428 |
| C | 2.580459 | -1.060776 | 2.276854 |
| H | 3.038635 | -1.930215 | 2.767313 |
| H | 1.569289 | -0.962160 | 2.688366 |
